# Consideration of sex and gender in Cochrane reviews of interventions for preventing healthcare-associated infections: a methodology study

**DOI:** 10.1186/s12913-019-4001-9

**Published:** 2019-03-15

**Authors:** Jesús López-Alcalde, Elena Stallings, Sheila Cabir Nunes, Abelardo Fernández Chávez, Mathilde Daheron, Xavier Bonfill Cosp, Javier Zamora

**Affiliations:** 1 Department of Paediatrics, Obstetrics & Gynaecology and Preventative Medicine, Universitat Autònoma de Barcelona, Barcelona, Spain; 2 Faculty of Health Sciences, Universidad Francisco de Vitoria (UFV)-Madrid, Madrid, Spain; 3 Clinical Biostatistics Unit, Hospital Universitario Ramón y Cajal (IRYCIS), Madrid, Spain; 4 Cochrane Associate Centre of Madrid, Madrid, Spain; 5 Independent Researcher, Madrid, Spain; 6 Preventive medicine Unit, Hospital Universitario Ramón y Cajal, Madrid, Spain; 7 Iberoamerican Cochrane Centre, IIB Sant Pau, Universitat Autònoma de Barcelona, Madrid, Spain; 8 CIBER Epidemiology and Public Health (CIBERESP), Madrid, Spain

**Keywords:** Systematic reviews, Data extraction, Sex, Gender, Sex/gender, Equity, Cochrane, Gender bias, Healthcare-associated infection

## Abstract

**Background:**

Healthcare-associated infections (HAIs) are common and increase morbidity, mortality, and healthcare costs. Their control continues to be an unresolved issue worldwide. HAIs epidemiology shows sex/gender differences. Thus the lack of consideration of sex/gender in Cochrane reviews will limit their applicability and capacity to support informed decisions. This study aims to describe the extent to which Cochrane reviews of interventions for preventing HAIs consider sex and gender.

**Methods:**

Methodology study appraising Cochrane reviews of interventions to prevent HAIs. Search methods: *Cochrane Database of Systematic Reviews* from 1995 (launch of the journal) to 31 December 2016. Two authors independently extracted data with *EPPI-Reviewer 4* software, and independently appraised the sex/gender content of the reviews with the *Sex and Gender Appraisal Tool for Systematic Reviews (SGAT-SR)*.

**Results:**

This study included 113 reviews assessing the effects of interventions for preventing HAIs. 100 reviews (88%) used at least one sex or gender-related term. The terminology used was heterogeneous, being “sex” the term used in more reviews (51%). No review defined neither sex nor gender. Thus we could not assess the definitions provided. Consideration of sex and gender was practically absent in the included reviews; in fact, no review met all the applicable items of the SGAT-SR, and 51 reviews (50%) fulfilled no item. No review provided a complete description of the sex and the gender of the samples of the included studies. Only ten reviews (10%) planned to perform sex- and gender-based analysis and only three (3%) could complete the analysis. The method chosen was always the subgroup analysis based on sex (one review) or gender (two reviews). Three reviews (3%) considered sex or gender-related findings in the conclusions.

**Conclusion:**

Consideration of sex and gender in Cochrane reviews of interventions for preventing HAIs was practically absent. This lack of attention to sex and gender reduces the quality of Cochrane reviews, and their applicability for all people: women and men, boys and girls, and people of diverse gender identities. Cochrane should attempt to address the shortfalls detected.

**Electronic supplementary material:**

The online version of this article (10.1186/s12913-019-4001-9) contains supplementary material, which is available to authorized users.

## Background

### Health inequality and health inequity

‘Health inequality’ and ‘health inequity’ are commonly confused terms, although they have different meanings. Health inequalities refer to the differences in health status or in the distribution of health determinants between different populations (e.g., racial, ethnic, sex, gender, sexual orientation, or socioeconomic groups) [1]. On the other hand, ‘health inequities’, also known as ‘health disparities’ [2], are avoidable and unfair differences in health across socioeconomic, demographic and geographic factors [1–6]. According to the World Health Organization (WHO) *Commission on Social Determinants of Health*, health inequity is caused by the following interacting factors: a) the socioeconomic and political context, b) the social position, b) the material circumstances, and d) the health system [7].

To reduce health inequities both within and between countries remains a priority on the agenda of international organisations, such as the WHO, and local, regional and national governments [8, 9]. The design and implementation of health care interventions and health programmes should apply an “equity lens” to ensure that benefits reach the most hard-to-reach segments of the population and to avoid intervention-generated inequalities [10, 11]. See Additional file 1 for definitions of key terms.

### The relevance of sex and gender in health

Sex, gender, or sexual orientation are characteristics that may contribute to health inequalities and health inequities [5, 10, 12, 13]. The concepts of sex and gender are distinct but interrelated [14]. According to the *Canadian Institutes of Health Research,* every cell is sexed, and every person is gendered [15]. Sex, usually defined as female or male, refers to a number of biological characteristics in humans and animals [16]. Sex is linked with physical and physiological features, such as chromosomes, gene expression, hormone function and reproductive/sexual anatomy [16, 17].

On the other hand, gender refers to the social roles, behaviours, expressions and identities of girls, women, boys, men, and gender diverse people [16, 17]. Consequently, gender influences how people perceive themselves and each other, how they behave and interact, and how power and resources distribute in society [16, 17].

Sex and gender are usually conceptualised as binary factors. Thus, analyses often consider male/female for sex, as well as masculine/feminine for gender [16, 17]. However, this may not reflect the reality, as the attributes of gender are multidimensional, dynamic, and interactive [18]. The term ‘sex/gender’ highlights this ‘entanglement’ of the biological and the social [17, 19, 20].

Biological and gender-based differences result in differential health risks, disease incidence, and health service needs [10]. Consequently, sex and gender interactions can influence health and well-being in a variety of ways [16]. First, pharmacokinetics and pharmacodynamics of drugs differ between sexes, resulting in differential adverse event profiles and further affecting treatment outcomes [21–23]. Secondly, sex and gender both affect environmental and occupational risks, risk-taking behaviours, access to health care, health care-seeking behaviour, health care utilisation, and perceived experience with health care, and thus, disease prevalence and treatment outcomes [16, 24].

### Consideration of sex and gender in research

The consideration of sex and gender in research is relevant for many reasons, such as for warranting scientific rigour, for reducing and enhancing the effectiveness of healthcare interventions, for promoting an informed-decision making, and for addressing inequities in health [17, 25–27]. The absence of consideration of sex and gender in research limits the external validity of research findings and their applicability for women, but also for men [16].

Various stakeholders (e.g., journal editors, research funders, policymakers) agree that sex and gender matter to health outcomes [16]. As an example, the National Institutes of Health (NIH) Revitalization Act of 1993 in the United States of America (USA) required NIH-funded clinical trials to include women and minorities as participants and to assess outcomes by sex and race or ethnicity [28]. Also, other relevant stakeholders are asking systematic reviews (SRs) to determine the evidence of differential effects across age, sex and socioeconomic status [29]; this is the case of NICE (*National Institute for Health and Care Excellence*) [30], or the *PRISMA* statement [31].

However, research design, reporting, and implementation, and general science communication often neglect sex and gender differences [14, 16, 17, 25, 26, 32–35] and policies attempting to solve this problem, such as the NIH policies cited above, have not resulted in significant increases in reporting results by sex, race, or ethnicity [36].

### Methods to consider sex and gender in systematic reviews

A SR is a review that departs from a clear question and follows rigorous and explicit methods in all its stages, that is, from the identification of the studies to the analysis of the data [37].

SRs are essential tools to transfer research knowledge into evidence-informed policy and practice [29, 38, 39]. Also, SRs are crucial in the promotion of health equity as they help to determine the effects of interventions across studies conducted in a variety of settings and populations, which allows for the exploration of both prognostic factors and treatment-covariate interactions [4, 29, 40–42]. Decision-makers are interested in health equity as one of the considerations for decision-making, as they need to know the effects of interventions in the overall population and across population groups [5, 43]. Considering sex and gender in SRs is a significant step forward in determining to whom the evidence applies, which is critical to make sound clinical and policy decisions [33].

Sex- and gender-based analysis (SGBA) is the analytical approach that incoporates the sex and gender perspective into health research, policies, and programmes, and in health planning and decision-making [16]. SGBA systemically inquiries about biological (sex-based) and socio-cultural (gender-based) differences between women and men, and boys and girls, without presuming that there are disparities [44]. In the context of SRs, SGBA is any analytical framework aiming to promote the consideration of sex and gender properly within SRs, so they have the potential to expand their findings for all people: women and men, boys, girls, and people of diverse gender identities.

There are several methodological approaches for addressing factors related to equity (sex and gender among them) in SRs, such as performing subgroup analysis or performing targeted analyses of sex/gender populations [4].

### Consideration of sex and gender in Cochrane reviews

*Cochrane* is an international organisation that prepares SRs to support people in making well-informed decisions about health care (36). Although the extent to which current Cochrane reviews consider sex and gender is not well known, some studies suggest that there is much room for improvement. To our knowledge, only the study by Doull et al. [14] has evaluated the consideration of sex and gender in Cochrane reviews. This study concluded that SGBA was generally absent in a random sample of 38 Cochrane reviews published before April 2007 in cardiovascular health.

Moreover, Cochrane reviews seem to rarely assess whether interventions have intended or unintended effects on health equity [29]; according to another study, only 1% of a random sample of Cochrane reviews assessed differences in the effectiveness of interventions across socioeconomic or demographic factors [45]. This shortfall is also present in non-Cochrane SRs. In fact, the analysis or report of equity issues, [38, 46–49], and sex/gender in particular [17, 25, 26, 33, 35], is infrequent in SRs.

There are several obstacles to consider sex/gender in any type of SRs, such as how the included studies defined sex and gender, the methodological difficulties in measuring and analysing sex and gender, the availability of data to perform sex and gender analysis, and also the quality of this data [33]. As an example, the studies included in SRs usually do not report about the inclusion of specific populations or, if they do, they may not assess variation in effects across critical characteristics, such as sex, age, ethnicity or socioeconomic status [5]. Moreover, sex and gender are highly interrelated, and it is sometimes difficult to attribute particular male-female differences to either sex or gender alone [24]. Consequently, SRs do not clearly identify to whom the research results apply and do not present adequate data and analyses about health equity factors, including sex and gender [34, 40, 50–52]. A proper SGBA framework can help in determining external validity—to whom a particular body of evidence applies and to what degree there is sufficient evidence to generalise results [14].

*The Sex/Gender Methods Group*, a subgroup of the *Campbell and Cochrane Equity Methods Group* [53], was established in December 2005. One of its aims is to develop methods and tools to integrate sex and gender in the development and reporting of research synthesis [54]. Cochrane Madrid is making this a priority, and this article describes our first step to promote the application of an “equity lens” to Cochrane reviews.

### Healthcare-associated infections: a public health problem

Healthcare-associated infections (HAIs) are infections acquired as a result of the delivery of health care [55]. HAIs represent a public health problem worldwide, as about 6 and 4% of the hospitalised patients in Europe and the U. S, respectively, have at least one HAI [56, 57]. Rates of HAIs seem to be even higher in low or middle-income countries [58]. HAIs increase morbidity (prolonged hospital stay and worse prognosis) [59], mortality [59, 60], and healthcare costs [59, 61, 62]. Finally, there can be limited options for treating HAIs caused by certain drug-resistant organisms, such as methicillin-resistant *Staphylococcus aureus* (MRSA) [56, 57, 63].

### Role of sex and gender in infectious diseases transmission and outcomes

Traditionally, little attention has been paid to sex and gender differences in infectious diseases [24]. However, both sex and gender can affect infectious diseases incidence, duration, severity, and mortality through several pathways [24].

There are biological differences between sexes that affect infectious diseases. For example, pregnant and lactating women represent a high-risk group for many infectious diseases [24], or females have an increased risk of catheter-associated urinary tract infection (CAUTI) due to anatomy that facilitates the bacterial contamination of the catheter. On the other hand, the vulnerability to infections differs between sexes due to differences in their immune systems; in this line, pre-menopausal females seem to have a natural advantage under septic conditions [64–69], which may be explained by the role of sex steroids, that change the host immune function, alter genes and modify behaviours that influence susceptibility and resistance to infection [69]. However, a recent SR concluded that the impact of sex on sepsis outcomes remains equivocal [70]. Finally, males may present higher overall HAIs prevalence, thirty-day mortality, and one-year mortality [71].

Gender differences in behaviours, activities, exposures, and access to resources and decision-making affect transmission and outcomes for different HAIs [24]. For example, women are less likely to receive antibiotics within 3 hours of the diagnostic of sepsis, as compared to men [72].

### Why this study is important

Sex and gender are necessary to understand the transmission of infectious diseases. The integration of a sex/gender perspective into SRs of interventions to control the transmission of HAIs is a new and challenging area but critical to defining successful infection control programmes [24].

Although we need more research to understand better how sex and gender interact with HAIs, there is enough knowledge available to justify the inclusion of a sex/gender perspective in research and programmes for HAIs. However, infection control strategies in the healthcare setting do not often consider sex and gender, and thus, they are generally the same for males or females [24, 73].

To consider sex and gender in Cochrane reviews of interventions to prevent HAIs is important. First, it will allow for the identification of the most effective and safest interventions for women and men. Second, it will contribute to the reduction of health inequities between men and women, and thereby promote human rights [24]. Third, the consideration of sex and gender in Cochrane reviews will help an informed-decision making for women and men. Fourth, the findings of our study will contribute to promoting the incorporation of a sex/gender perspective into Cochrane reviews of any topic.

We chose to focus our study on Cochrane reviews for several reasons. First, we foresaw that a high number of Cochrane reviews had evaluated infection control interventions [74]. Second, a wide range of Cochrane Review Groups publishes Cochrane reviews of infection control interventions, which gives an overview of the general approach of Cochrane as an organisation towards sex and gender. Third, Cochrane reviews are recognised as a reliable source of evidence worldwide and have a high impact on decision making, for example, through the consideration of Cochrane reviews in clinical guidelines [75–77], or in the medical policy documents of private health insurers [78]. As an example, the percentage of Cochrane reviews used in WHO guidelines have been steadily rising, and so far for 2016, Cochrane reviews have been included in 90% of the WHO guidelines [77]. However, if Cochrane reviews do not consider sex and gender, they will not be able to generate evidence that applies to all the people that can benefit from the research findings. Thus, Cochrane reviews may not be useful for policy-makers [29] who seek information on the distribution of effects in the population [48, 79], or may even lead to the implementation of policies and programs which inadvertently increase health inequities [80, 81].

### Study aims

The general aim of this study was to describe the extent to which Cochrane reviews of interventions for preventing HAIs consider sex and gender. The specific objectives were the following.

- Objective 1. To describe and assess the terminology and definitions used for sex and gender.
- Objective 2. To determine the content of the reviews about sex and gender.
- Objective 3. To describe the SGBA of the reviews.
- Objective 4. To assess whether the review conclusions considered the sex- and gender-related findings.

## Methods

### Study design

Methodology study, that is, a study that assesses the methods used in randomised trials, other healthcare evaluations or systematic reviews [82].

We did not register this study in PROSPERO database because it did not meet the inclusion criteria, mainly because this is not a systematic review [83]. Our research does not adhere to any reporting statement as to our knowledge there is no guidance for reporting methodology studies.

### Criteria for considering reviews for this study

#### Types of reviews that were eligible

Cochrane reviews published from 1995 (launch of the journal) until 31st December 2016 that evaluated the effects of interventions for preventing HAIs. The review had to be defined as a ‘published review’ (not at protocol nor title stage), an ‘active review’ (not withdrawn), and as an ‘intervention review’, that is, a review assessing the effects (benefits, harms or both) of health care or health policy interventions [84]. Thus, we excluded the remaining types of Cochrane reviews: methodology reviews, diagnostic reviews, overviews of reviews, prognosis reviews, and qualitative reviews.

Participants: the review must have considered any healthcare consumer in risk of healthcare-associated colonisation or HAI, except for reviews focusing on neonates, pre-terms, low birth weight or immunocompromised patients, due to the epidemiological peculiarities of these participants. We also excluded reviews focusing on the prevention of infections in healthcare professionals. All healthcare settings were eligible.

Interventions: any strategy, pharmacological or not, aimed at preventing any healthcare-associated colonisation or infection. Additional file 2 details the eligible interventions. The review could consider any comparator, that is, an inactive comparator (such as doing nothing, use of placebo, or use of a sham intervention), or an active one (such as a pharmacological intervention, or a non-pharmacological intervention, for example, an educational or organisational one).

Outcomes: the review must have planned to assess at least one of the following outcomes: (a) occurrence of HAIs; (b) occurrence of colonisations; (c) mortality due to HAI; (d) total mortality; (e) resistance to antimicrobials; or (f) any surrogate measure of HAI, such as fever, positive culture, or antibiotic use. The review had to consider at least one of the previous outcomes in the “Types of outcome measures” section (as a primary or secondary outcome) or had to present results about any of these outcomes (“Results” section).

### Search methods for identification of reviews

We searched the *Cochrane Database of Systematic Reviews (CDSR)* looking for all the Cochrane interventions reviews active and published from 1995 (launch of the journal) until 31st December 2016. Additional file 3 details the full search strategy. We used *EPPI-Reviewer 4* software [85] to create the database of reviews.

### Selection of reviews

Two authors (JLA and SCN or ES) screened each title and abstract independently to select potentially eligible reviews. If there was any uncertainty based on this information, we obtained the full-text review for further assessment. Two authors (JLA and SCN or ES or AFC) independently assessed the eligibility of the retrieved full texts and resolved disagreements by discussion. If there was no consensus, we consulted a third author (JZ). We used *EPPI-Reviewer 4* software [85] to implement the selection process. We piloted the selection process with 100 records. We created a PRISMA flowchart [86] describing the results of the selection process.

### Data extraction

We designed a data extraction template and piloted the form on ten reviews. Two of the piloted reviews were not eligible for this study, but we used them because they had been defined by the *Canadian Institutes of Health Research* [87] as exemplar Cochrane reviews regarding the consideration of sex and gender. The data extraction template is available upon request.

We extracted data with the *EPPI-Reviewer 4* software [85]. At least two authors (JLA, ES, AF or SCN) extracted the data for each item of the form. For critical items, two authors extracted data independently. For other items, one author extracted the data, and another author cross-checked the information extracted. We resolved discrepancies by consensus. In the case of no consensus, a third author intervened. We did not contact the review authors to obtain missing information or clarification.

Next, we detail the methods used to complete each specific objective.

### Analysis methods

#### Objective 1. To describe and assess the terminology and definitions used for sex and gender

We described the terms used for sex and gender and the sections in which these terms appeared. Moreover, we assessed the appropriateness of the terms and definitions used by comparing them with the proposals of the *SAGER* (*Reporting of Sex and Gender Equity in Research)* guidelines [16]. We followed the classifications detailed in Tables 1 and 2. See also Additional file 1 for sex and gender definitions.

**Table 1:** Classification of the sex and gender terms

Classification	
Correct	Any of the following. 1. Male or female for sex and an adequate definition of sex (biological). 2. Men or women for gender and an adequate definition of gender (cultural and socially determined roles).
Incorrect	Any of the following. 1. Male or female for gender. 2. Men or women for sex.
Unclear	Any of the following. 1. Terms for sex or gender used without defining sex or gender. For example, if a review stated that the sex of the participants was male (56%) and female (64%), but there was no definition for sex, we judged the terminology as unclear because we could not know that the review was referring to sex or gender. 2. Terms inconsistently used in the review. For example, the review used the terms ‘male’ and ‘men’ for the same concept. 3. Abbreviations used without the full term provided.
Not applicable	No mention to sex or gender

**Table 2:** Classification of the sex and gender definitions

Classification	
Correct	Definitions like SAGER guidelines
Incorrect	Definitions different to SAGER guidelines
Unclear	Terms for sex or gender used without a definition for sex or gender
Not applicable	There was no mention of sex or gender in the review

#### Objective 2. To determine the content of the reviews about sex and gender

We determined the sex and gender content of each review according to the domains proposed by the *Sex and Gender Appraisal Tool for Systematic Reviews (SGAT-SR)* [14]. This 21-item tool assesses how a Cochrane review has considered sex and gender. It appraises seven review sections: (a) Background, (b) Objectives, (c) Criteria for inclusion/exclusion, (d) Methods, (e) Results and analysis, (f) Discussion and conclusions, and (g) Table of included studies. Each question of the tool has four answers: (a) “Yes, review met criteria”; (b) “No, the review did not meet criteria”; (c) “Item was not applicable to review”; or (d) “Unable to determine”. The tool allows adding free text comments to each response in case of need. We made minor wording changes to the SGAT-SR. Additional file 4 shows the domains of the tool and the guidance on which we based our judgements.

We tabulated the responses to the tool by simple counts, and summarised the results numerically to provide an indication of overall responses (as done by Doull et al. [14] in a methodology study that used the *SGAT-SR* to examine the consideration of sex and gender in a sample of Cochrane reviews in the area of cardiovascular health). We calculated the percentage of reviews meeting each item only when this was applicable (Number of reviews meeting item × 100/Total number of included reviews in which the item was applicable); thus, when an item was not applicable for a review, that review was considered neither in the numerator nor in the denominator for that item. This omission applied to reviews focusing on females only, that is, those reviews addressing pregnancy and delivery. Breast cancer was not the case, as it can also affect males.

Two authors (SCN, JLA, ES, or AFC) independently answered each item of the tool not masked to the review details. We resolved disagreements through discussion and by consulting a third author if there was no consensus. We tabulated the judgements of the SGAT-SR and used *Powerpoint 2016* [88] and *Review Manager 5.3* [89] to summarise our judgements graphically.

#### Objective 3. To describe the SGBA of the reviews

##### Objective 3a. To describe the reporting of the sex and the gender characteristics of the study participants

We described if the review had attempted to report the sex and gender characteristics of the participants recruited for each included study. For a review to describe the study samples accurately, we agreed that it should have attempted to report at least the following information for each included study (based on Clayton et al. [90]). We will report the following key sex and gender characteristics.

• Sex measurement (ascertained by genotyping of blood sample)

• Number of female and male participants

• Gender measurement (ascertained by self-report)

• Number of women and men participants

We focused on the information that the review reported in the table of included studies. We classified the review reporting of the sex and gender characteristics of the study participants according to the following categories.

• *Correct with a complete description:* the review attempted to describe all the key sex and gender characteristics, and this information was available for all the included studies.

• *Correct with an incomplete description:* the review attempted to describe all the key sex and gender characteristics, but this information was not available for all the included studies, which was highlighted by the review authors.

• *Incorrect:* the review did not attempt to describe all the key sex and gender characteristics for all the included studies.

##### Objective 3b. To describe the SGBA in the reviews

First, we identified the SRs that had planned or used any SGBA. Second, we described the SGBA methods planned and finally used. We considered Welch et al. [4] to describe the SGBA methods used:

• Subgroup analysis-pooled results: SRs that assessed impacts of health interventions on the outcome using subgroup analysis with pooling.

• Subgroup analysis-descriptive: SRs that described within-study differences without pooling.

• Targeted analyses of sex or gender populations

• Other methods used for SGBA

### Objective 4. To assess whether the review conclusions considered the sex- and gender-related findings

We calculated the percentage of reviews considering the sex- and gender-related findings in the conclusions, in particular:

- % of reviews considering sex or gender findings in the “Implications for clinical practice” section
- % of reviews considering sex or gender findings in the “Implications for research” section
- % of reviews performing SGBA that considered sex or gender findings in the “Implications for clinical practice” section
- % of reviews performing SGBA that considered sex or gender findings in the “Implications for research” section

## Results

### Results of the search

**Fig. 1:**
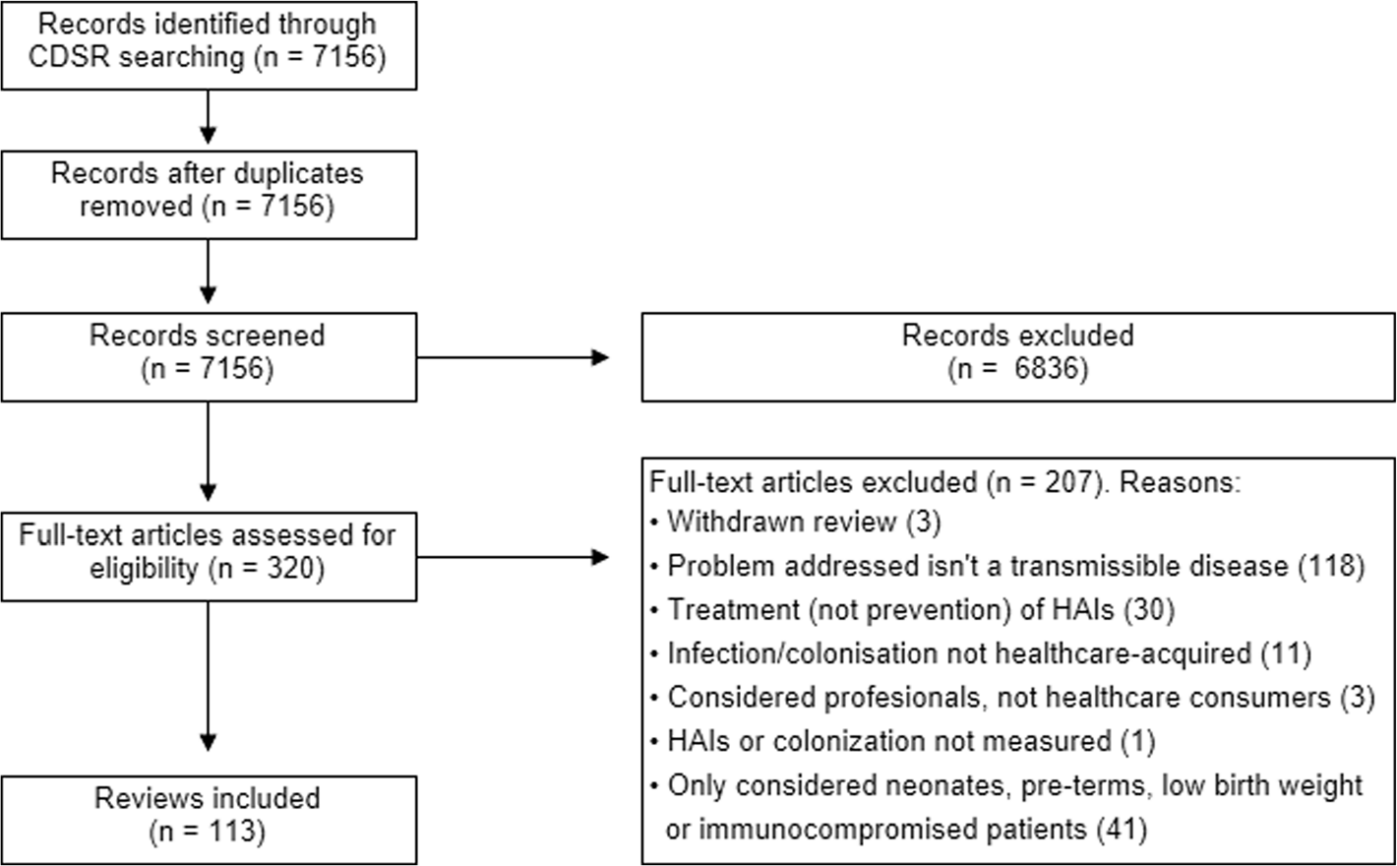
Flow diagram of the selection process

The search strategy in *CDSR* generated 7156 records. First, we screened their titles and abstracts, and we excluded 6836 records because they were not eligible. Second, we subsequently retrieved 320 full texts for further examination. We excluded 207 full texts (see reasons in Fig. 1), and we finally included 113 reviews.

### Description of the included reviews

This study included 113 reviews (see Additional file 5, also available in RIS format upon demand). The reviews were published between 2003 and 2016 within 23 different Cochrane Review Groups. The Cochrane Wounds Group was the review group with the most reviews included in this study (35/113 reviews [31%]), followed by the Anaesthesia, Critical, and Emergency Care Group (13/113 reviews [12%]), and the Incontinence Group (10/113 reviews [9%]). Each of the remaining Cochrane Review Groups published less than ten of the included reviews.

All the reviews evaluated the effects of interventions for preventing HAIs. The interventions most frequently evaluated were those aiming to prevent HAIs associated to surgery (50/113 [44%] reviews), followed by interventions to prevent infections associated to vascular accesses (21/113 [19%] reviews), and interventions based on patient and healthcare personnel hygiene (14/113 [12%] reviews). Other interventions evaluated were, for example, those to prevent urinary catheter-associated infection, education and training to prevent HAIs, or interventions to prevent infection associated with dental procedures.

All the reviews planned to assess at least one of our study outcomes. HAI was defined as eligible in 105/113 (93%) reviews, followed by total mortality (66/113 reviews [58%]), surrogate measures of HAI (31/113 reviews [27%]), colonisation (19/113 reviews [17%]), mortality due to HAI (15/113 reviews [13%]), and resistance to antimicrobials (14/113 reviews [12%]).

The most common study design was the randomised controlled trial (RCT), which was eligible in all the included reviews (100%). The RCT was the only study design eligible in 57 reviews (50%), but another 55 reviews (49%) admitted the inclusion of at least one type of non-randomised study (NRS) as well. For one review (1%), it was unclear if NRS were eligible.

### Objective 1. To describe and assess the terminology and definitions used for sex and gender

100/113 reviews (88%) used at least one sex or gender-related term. The terms used varied, and no review made it explicit that sex and gender were different concepts. ‘Sex’ was the term used in the most reviews (58/113 reviews, 51%), followed by ‘male’ (54/113 reviews, 48%), ‘woman’ or ‘women’ (53/113 reviews, 47%), ‘female’ (50/113 reviews, 44%), ‘gender’ (42/113 reviews, 37%), ‘men’ or ‘man’ (28/113 reviews, 25%), or other terms, in particular ‘boys’ and ‘girls’ (3/113 reviews, 3%). 13/113 reviews (12%) used no term related to sex nor gender.

The terms appeared mainly in the ‘Characteristics of studies’ section (84/113 reviews, 74%). On the other hand, only 10/113 (9%) reviews used these terms in the conclusions section. Table 3 details the review sections that used these terms.

**Table 3:** Percentage of reviews using sex and gender terms in each review section

Review section	Sex	Gender	Male^a^	Female^b^	Men^c^	Women^d^	Other
Abstract	2%	–	–	1%	2%	8%	–
Background	3%	1%	4%	5%	3%	15%	–
Plain language summary		2%	1%	2%	–	8%	–
Eligibility criteria	4%	8%	1%	1%	4%	13%	–
Search methods	–	–	–	–	–	1%	–
Data collection and analysis	12%	12%	4%	4%	5%	9%	–
Results	9%	10%	12%	12%	7%	18%	1%
Discussion	4%	2%	2%	1%	4%	15%	–
Authors’ conclusions	1%	–	–	1%	1%	7%	–
Characteristics of studies	31%	22%	42%	35%	13%	35%	2%
Other sections	5%	3%	–	–	–	3%	–
Not reported	49%	63%	52%	56%	75%	53%	97%

^a^‘Male’, ‘males’ or ‘m’

^b^‘Female’, ‘females’ or ‘f

^c^‘Men’ or ‘man’ or ‘m’

^d^‘Women’ or woman

**Fig. 2:**
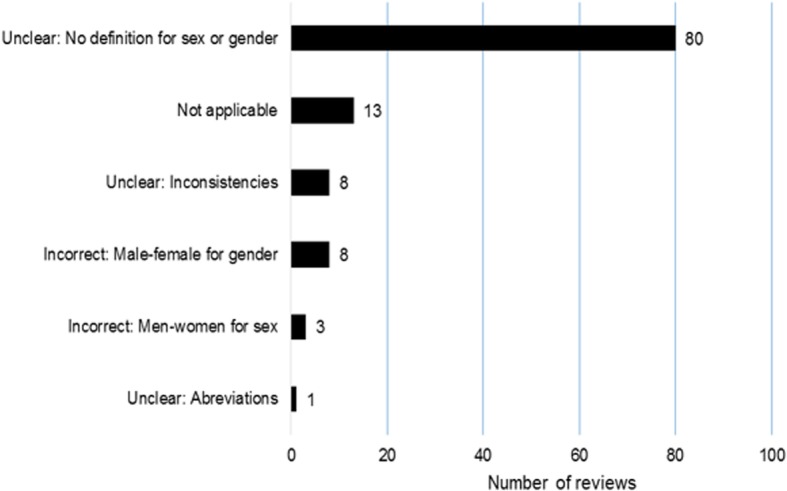
Appropriateness of the sex and gender terminology

The reviews defined neither sex nor gender. Thus we could not assess the definitions provided. Moreover, the absence of definitions hindered our attempt to assess the appropriateness of the terms used. For thirteen reviews it was not applicable to assess the terminology because they did not use sex or gender terms. Another 11/100 (11%) reviews used incorrect terms: ‘male’ or ‘female’ for gender (8 reviews), and ‘men’ or ‘women’ for sex (3 reviews). Also, 89/100 reviews (89%) used unclear terminology because there was no definition for sex or gender (80 reviews), because both sex and gender terms were used inconsistently apparently for the same concepts (eight reviews), or because the abbreviation “M” was used without detailing the full term (one review). Reasons to judge the terms as unclear or incorrect are shown in Fig. 2.

### Objective 2. To determine the content of the reviews about sex and gender

**Fig. 3:**
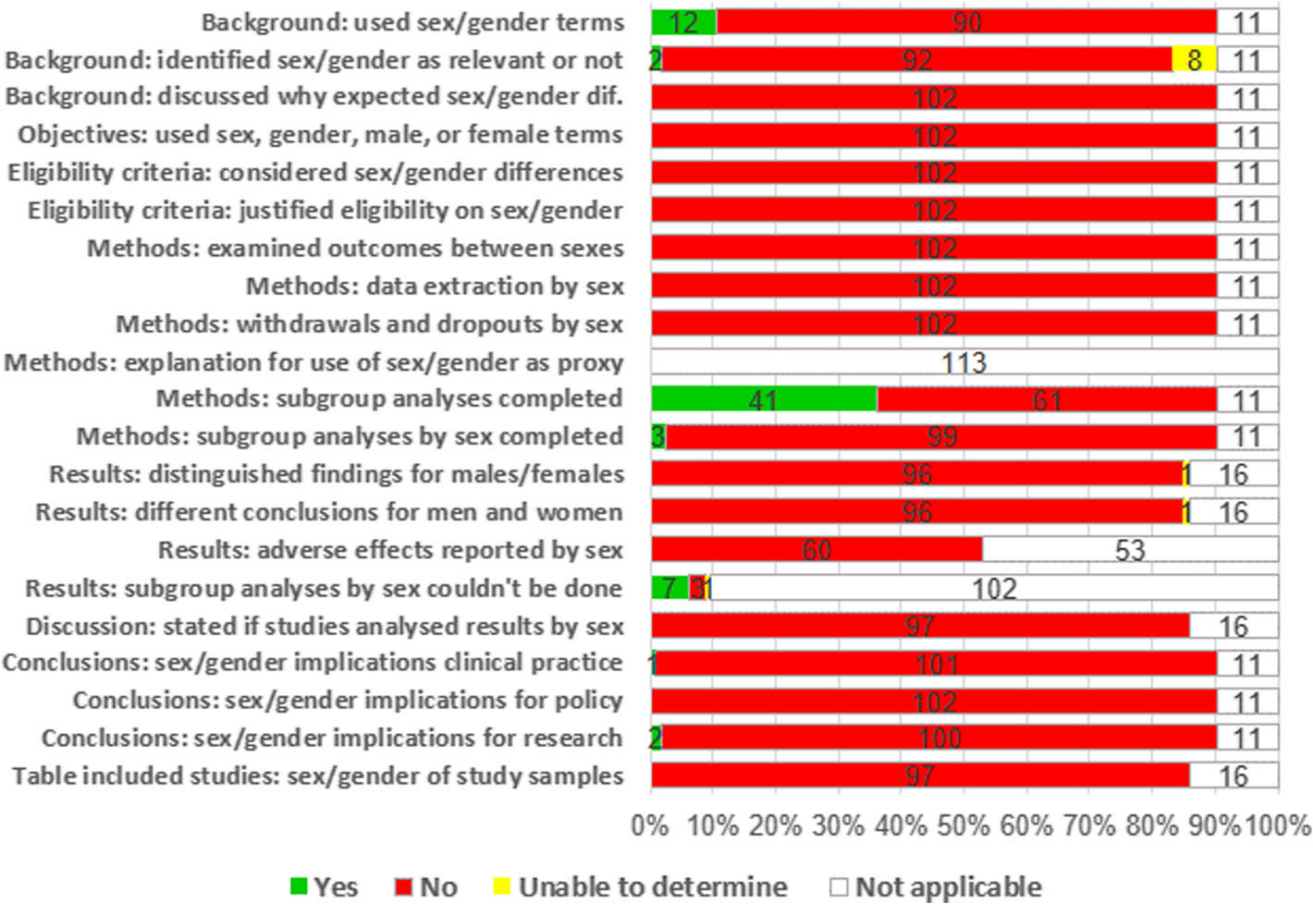
Sex and gender appraisal graph. Judgements across reviews for each item of the tool

We did not determine the sex and gender content for eleven reviews [91–101] as they included females only due to the topics addressed, that is, interventions to prevent HAIs in caesarean section, operative vaginal delivery, abortion, amniotomy, or prelabour rupture of membranes. Table 4 details the overall responses to the SGAT-SR questions, and Additional files 6 and 7 detail our responses to the tool for each review, which can be seen graphically in Fig. 3.

**Table 4:** Responses to the Sex and Gender Appraisal Tool for Systematic Reviews (SGAT-SR)

Reviews meeting the criteria (n)	Tool question	Reviews meeting the criteria (n)			
		Yes	No	Unable to determine	Not applicable
1.Background	1.1. Are the terms sex and gender used in the background?	12	90	0	11
	1.2. Are sex/gender identified as relevant or not to review question?	2	92	8	11
	1.3. Does background discuss why sex/gender differences may be expected?	0	102	0	11
2. Objectives	2.1. Are the terms sex, gender, male, or female used in objectives?	0	102	0	11
3.Criteria for inclusion-exclusion	3.1. Do the review’s inclusion-exclusion criteria consider sex-gender differences?	0	102	0	11
	3.2. Was there justification or explanation for the exclusion of some groups?	0	102	0	11
4. Methods	4.1. Does the review examine whether outcome measures are different for males and females?	0	102	0	11
	4.2. Did the review extract data by sex?	0	102	0	11
	4.3. Did the review extract data on sex of withdrawals and dropouts?	0	102	0	11
	4.4. In cases where sex/gender is used as a proxy, is there an explanation?	0	0	0	113
	4.5. Were any subgroup analyses completed?	41	61	0	11
	4.6. Were subgroup analyses by sex completed?	3	99	0	11
5.Results and analysis	5.1. Do results distinguish between findings for males/females?	0	96	1	16
	5.2. Does the review report conclusions that are different for men and women?	0	96	1	16
	5.3. If adverse effects are reported, is information sex-disaggregated?	0	60	0	53
	5.4. Does review note that subgroup analyses by sex could not be done?	7	3	1	102
6. Discussion and conclusion	6.1. Does the review report that primary studies analysed or failed to analyse results by sex?	0	97	0	16
	6.2. Does the review address sex/gender implications for clinical practice?	1	101	0	11
	6.3. Does the review address sex/gender implications for policy and regulation?	0	102	0	11
	6.4. Does the review address sex/gender implications for research?	2	100	0	11
7. Table of included studies	7.1. Detailed information on sex/gender of the study samples?	0	97	0	16

No review met all the applicable items of the SGAT-SR. In fact, 51/102 reviews (50%) fulfilled none of the applicable items. The remaining reviews fulfilled one (38/102 reviews [37%]), two (9/102 [9%]), three (3/102 [3%)], or four (1/102 [1%]) of the applicable items.

### Review section: background

12/102 reviews (12%) used sex or gender-related terms in the background. However, only 2/102 reviews (2%) defined the relevance of sex or gender to the review question, and this was unclear for 8/102 reviews (8%). No review (0/102) discussed in its background why sex or gender differences might be expected.

### Review section: objectives

No review (0/102 reviews) used the terms sex, gender, male, or female in the objectives.

### Review section: criteria for inclusion/exclusion

No review used sex or gender as criteria for deciding on study eligibility (0/102 reviews) or explained why to consider sex or gender differences for study eligibility (0/102 reviews).

### Review section: methods

No review (0/102 reviews) planned to examine or finally examined whether outcome measures were different for males and females. No review planned to extract or extracted data by sex (0/102 reviews). No review extracted withdrawals and dropouts by sex (0/102 reviews). No review used sex/gender as a proxy for other measures (0/102 reviews).

86/102 reviews (84%) planned to perform subgroup analysis, but only 41/102 reviews (40%) could complete at least one subgroup analysis. 10/102 reviews (10%) chose sex as the factor to analyse, but only three (3/102 [3%]) could complete the analysis.

### Review section: results and analysis

No review in which it was applicable distinguished between findings for males and females (0/97), or reported conclusions (of effectiveness, efficacy, safety) that were different for men and women (0/97 reviews). For one review [102] we judged that these two items of the tool were unclear because the results and conclusions were reported separately only for some review outcomes. For sixteen reviews it was not applicable to assess these items because they focused on one sex only (11 reviews), or they included no study (6 reviews).

No review (0/60 reviews) reported adverse effects disaggregated by sex. For the remaining 53 reviews, it was not applicable to assess this item of the tool due to the following reasons: the reviews focused on one sex only (11 reviews), they included no study (6 reviews), they did not plan to assess adverse effects (33 reviews), or although they planned to assess safety the included studies did not report adverse effects (9 reviews).

Seven out of the ten reviews (70%) that planned but could not perform subgroup analysis by sex explained why this analysis could not be completed.

### Review section: discussion and conclusions

No review in which it was applicable (0/97 reviews) reported if the included studies had analysed or failed to analyse results by sex. This aspect was not applicable in 16 reviews because they had no included studies (6 reviews) or because they focused on one sex only (11 reviews).

A total of 3/102 reviews (3%) considered sex/gender implications in their conclusions: 1/102 review (1%) addressed the implications for clinical practice, and the other 2/102 reviews (2%) considered the implications for research. However, no review addressed the implications of sex/gender for policy and regulation.

### Review section: table of included studies

No review (0/97 reviews [0%]) provided detailed information on the sex and the gender of the samples of all the included studies. For sixteen reviews, this was not applicable as they did not include any study (six reviews) or they focused on one sex only (eleven reviews).

Regarding the sex of the participants recruited, 78/97 reviews (80%) provided no information at all. Another 19/97 reviews (20%) provided unclear information. Regarding the gender, 87/97 reviews (90%) provided no information, and in the other 10/97 reviews (10%) the information was unclear. We considered that the information about the sex or gender of the recruited samples was unclear for several reasons: there was no definition for sex or gender, the authors used sex or gender terms but they did not state if they referred to sex or gender, or the authors misused the terms (men or women for sex, or male or female for gender).

### Objective 3. To describe the SGBA of the included reviews

#### Objective 3a. To describe the reporting of the sex and the gender characteristics of the study participants

The review reporting of the sex and gender characteristics of the study participants was always incorrect, as no review attempted to report the sex or the gender of the participants of all the included studies. The method to ascertain the sex or gender of the recruited participants was never reported in the reviews.

#### Objective 3b. Description of the SGBA in the included reviews

Ten reviews (10/102 [10%]) planned to perform SGBA, but only three (3/102 [3%]) could complete the analysis. The method chosen for SGBA was always the use of subgroup analysis based on sex (one review) or gender (two reviews). Two reviews performed subgroup analysis by pooling results of studies to assess the effect of sex or gender on the outcome, and the other review performed a descriptive subgroup analysis, that is, described within-study differences by gender without pooling.

### Objective 4. To assess whether the review conclusions considered the sex- and gender-related findings

Only 3/102 reviews (3%) [102–104] considered the sex or gender-related findings in the conclusions. One of them mentioned the sex/gender implications for clinical practice by stating that “Siliconised catheters may be less likely to cause urethral side effects in men” [102]. The other two reviews considered the implications of sex/gender for research by stating that “sub-group analysis would give valuable data as to whether certain policies are more effective in sub-groups such as females” [103], or “Future trials comparing suprapubic and intermittent urethral catheterisation for short-term use in hospitalised men should be conducted [...]” [104]. However, no review addressed the implications of sex/gender for policy and regulation. Table 5 details how the reviews considered sex or gender findings in their conclusions.

**Table 5:** Reviews considering sex or gender findings in the conclusions

	% reviews (n/total number of reviews)
Reviews considering sex or gender findings in the “Implications for clinical practice” section	1% (1/102)
Reviews considering sex or gender findings in the “Implications for research” section	2% (2/102)
Reviews performing SGBA that considered sex or gender findings in the “Implications for clinical practice” section	33% (1/3)
Reviews performing SGBA that considered sex or gender findings in the “Implications for research” section	33% (1/3)

*SGBA* sex- and gender-based analysis

## Discussion

### Summary of main results

One hundred thirteen Cochrane reviews assessed the effects of interventions to prevent HAIs. Consideration of sex and gender in these reviews was practically absent. Several reasons may explain this inattention.

First, SRs may replicate limitations or gaps in primary studies regarding their research question, their data analysis, and the interpretation of their results [14]. Primary studies of hospital infection control interventions usually ignore sex, gender or having a gender identity that does not match one’s biological sex. Several reasons can explain this inattention. For example, researchers may consider that making conclusions about these factors is challenging because hospital-based studies are often based on small sample sizes. Moreover, it is probably unfeasible for retrospective large primary studies, or those based on electronic records, to capture gender and sex differences because they are based on data sources that have not collected vital information, such as the assessment of biological sex by genotyping or the gender measurement by self-report. As a consequence, the included studies may not have reported the sex and gender distributions of the recruited samples. Alternatively, the authors may have just detailed that the study groups had equal numbers of males and females at baseline as a proxy for the randomisation success. Thus, sex was “controlled for” in the primary studies rather than considered to assess how the study outcomes vary across sex or gender groups [5]. Our study did not assess the data provided by the primary studies, so we cannot confirm this. Nevertheless, other studies have highlighted that RCTs do not usually provide sex or gender disaggregated data [14, 36, 105–109], and that sex/gender policies have not resulted in significant increases in reporting results by sex [36]. On the other hand, the SRs included in our study did not even plan to perform any SGBA, so we think the lack of data in the included studies cannot entirely explain their lack of attention to sex and gender.

Second, Cochrane launched the SGSR-AT [110] in 2011, that is, the Cochrane guidance to integrate sex and gender in SRs is quite recent. Thus, the guidance was not available at the time of writing the protocols of the included reviews. In fact, 31 included reviews (27%) were published in 2011 or before, and the 43 SRs published from 2012 to 2014 probably did not consider this guidance at their protocol stage.

Third, the authors of the included SRs may have thought that sex/gender was not a relevant factor to consider when evaluating the effects of interventions to prevent HAIs. Again, this is possible, but we cannot confirm that this was the case as the review authors did not make this assumption explicit in the reviews.

Fourth, the review authors did not even consider the role of sex/gender while planning the review. This situation is, in our opinion, the most plausible explanation and the most worrying one, as it denotes a knowledge gap about the potential relevance of sex and gender in these reviews.

### Overall completeness and applicability of evidence

This study aimed to describe the extent to which Cochrane reviews of interventions for preventing HAIs consider sex and gender. We are quite confident that we have achieved our goal. Firstly, we have identified all the Cochrane reviews of interventions to prevent HAIs (we did not rely on a sample of Cochrane reviews), and secondly, we have demonstrated that SGBA is practically absent in these reviews.

Our study findings apply only to Cochrane reviews of interventions to prevent HAIs. Thus, we cannot infer that our study findings can be applied to Cochrane reviews of other health topics, or to non-Cochrane reviews. First, other Cochrane reviews may have incorporated the guidance of the Sex/Gender Methods Group [111]. Second, many interventions to prevent HAIs are non-pharmacological, for example, the use of gloves or hand washing. The methods used to evaluate non-pharmacological interventions may differ from those applied to evaluate drugs, and this may imply a different approach to consider sex and gender. Third, there are other health areas where the consideration of the relevance of sex and gender may be more common than in infection control research.

The findings of this study are relevant and confirm that the reviews did not consider the sex and the gender of the body of the evidence synthesised. Thus, these reviews do not provide critical information to judge the applicability of the results to the target population [112]. Also, the sex and gender characteristics should have been considered to judge the “indirectness” of the evidence with the GRADE (*Grading of Recommendations Assessment, Development and Evaluation*) system, and therefore, to rate the quality of that evidence [113]; however, we have demonstrated that this was not the case.

The tables of characteristics of the included studies are essential to describe the sex and the gender of the subjects considered within each study. In our opinion, the relevant information is the sex and gender characteristics of the sample from which the study results were obtained (and not the inclusion criteria of the study). However, specific guidance to collect and report this information in Cochrane reviews is still lacking.

### Strengths and limitations of this study

#### Strengths

We made efforts to identify all the Cochrane reviews assessing the effects of interventions to prevent HAIs. We obtained all the Cochrane reviews published in *CDSR* until December 31st, 2016 and screened all these records. To minimise bias, at least two authors independently participated in the selection process, and, in case of disagreement, a third author was consulted. We also defined and applied explicit exclusion criteria which made the process even more rigorous.

We determined the sex/gender content of each review with the SGAT-SR. Again, to minimise bias, at least two reviewers (JLA and ES or SCN) independently participated in the assessment. In case of disagreement, a third reviewer (JZ) was consulted. Furthermore, to improve the consistency we piloted the tool with several reviews, and we prepared a user guide.

The SGAT-SR helped us to assess how the different Cochrane review sections had considered sex and gender. We chose this tool because it is recommended by the Sex/Gender Cochrane Working Group [54]. Moreover, we performed an extensive search up to April 2017 that revealed a lack of additional sex- and gender-specific appraisal tools for SRs. As the tool had already been used to assess SRs on cardiovascular diseases, we were able to better understand the tool’s content by comparing our judgments with those presented in the published article [14]. Independent subject experts had also reviewed the tool to ensure consistency with common understandings of the concepts of sex and gender and to ensure compatibility with Cochrane review format and style [14].

#### Limitations

This study aimed to assess how the reviews had been conducted, and not how they had been reported. However, we did not write to the review authors to obtain any missing, incomplete, or unclear information. Thus, we made assumptions for information that was not clear by reading the review: we generally considered that the lack of reporting of a particular aspect meant that this was not done. However, this may not represent what the reviewers did.

During the selection and data extraction processes, we were not masked to the review team or institution. Moreover, JLA and SCN were the authors of this methodology study and three of the included reviews [114–116]. However, we prevented that this fact influenced the decisions, as the selection and extraction processes were done by at least two authors independently, and, in case of disagreement, we consulted a third author.

We did not measure the reliability of the SGAT-SR judgements (for example, by obtaining the kappa statistic), so we cannot confirm that our decisions were reliable.

We encountered some challenges with the use of the SGAT-SR. First, the tool needs to be more operative and manageable, that is, it should provide specific guidance with examples taken from other reviews. Second, it is not clear if some items of the tool refer to the planning of the review or to what the review finally did. Third, the tool should suggest when an item is not applicable; for example, for reviews with no included studies, it is not clear if the items related to the results must be answered as “not meeting the criteria” or as “not applicable”. Fourth, the tool does not allow explicit assessment on the dimension of sex and the dimension of gender separately. It gathers both dimensions in the same questions. We think that each of these two domains warrants a focused appraisal. Fifth, the tool does not assess relevant sections of a Cochrane review, such as the abstract, the plain language summary, the discussion, or the summary of findings tables.

As stated in the methods section, we considered that it was not applicable to use the SGAT-ST for reviews of topics focused on one sex, such as pregnancy or delivery. We decided this because we felt that the SGAT-SR was not developed to assess both features, sex and gender, separately. However, although it did not apply to the assessment of the sex content for these reviews, it was still relevant to assess how the gender was addressed.

Finally, it is noteworthy that although sex and gender are important factors in clinical research [90], more work is needed to standardise the way sex and gender are measured and reported, and the methods to determine how these factors influence health and health care [90]). There is no consensus on how to disaggregate demographic and outcome data by sex, gender, or both, or on how to report this information. Therefore, the report of crucial information on sex, gender, or both, is incomplete in primary studies and systematic reviews. In the end, this lack of consensus moves away from a personalised medicine approach.

### Agreements and disagreements with other studies or reviews

Our results are consistent with another study describing the consideration of SGBA in Cochrane reviews in the area of cardiovascular health [14], which also concluded that SGBA was practically absent in this sample of Cochrane reviews. In this line, our study supports the idea that current Cochrane reviews do not consider sex and gender, and that there is much room for improvement in this aspect. It is also noteworthy that, to our knowledge, only one study has appraised the SGBA done in Cochrane reviews [14].

This gap does not only affect Cochrane reviews, as it is also present in non-Cochrane ones. In fact, SRs do not often analyse or report equity issues in general [38, 46–49], and sex/gender in particular [17, 25, 26, 33, 35].

## Conclusions

### Main conclusions of this study

Consideration of sex and gender in Cochrane reviews of interventions for preventing HAIs was practically absent. This lack of attention to sex and gender reduces the quality of Cochrane reviews, and the applicability of their results for all people: women and men, boys and girls, and people of diverse gender identities.

### Recommendations derived from this study

Cochrane should map the consideration of sex and gender in all Cochrane reviews and, if necessary, plan how to address the shortfalls detected efficiently.

Cochrane should continue encouraging review authors to consider sex and gender in their reviews.

The SGAT-SR helps to assess how sex and gender have been considered in a Cochrane review, although this tool has some room for improvement.

Cochrane should provide review authors with more operative guidance to consider sex and gender in Cochrane reviews.

Cochrane guidance to consider sex and gender in Cochrane reviews should be updated, validated, and required to meet the *Methodological Expectations of Cochrane Intervention Reviews (MECIR) standards.*

Primary studies should consider sex and gender differences in their research questions, data, analyses, interpretation and reporting of the study results. This approach will facilitate the consideration of sex and gender in systematic reviews and meta-analyses.

## Additional files

Additional file 1: Glossary of terms. (DOCX 28 kb)

Additional file 2: Interventions defined as eligible. (DOCX 13 kb)

Additional file 3: Search strategy. (DOCX 15 kb)

Additional file 4: SGAT-SR template and guidance. (DOCX 30 kb)

Additional file 5: List of included reviews. (DOCX 21 kb)

Additional file 6: Responses to the Sex and Gender Appraisal Tool for Systematic Reviews (SGAT-SR). (DOCX 96 kb)

Additional file 7: Sex and gender appraisal summary. (PDF 643 kb)

## Abbreviations

CAUTI: Catheter-associated urinary tract infection
CDSR: *Cochrane Database of Systematic Reviews*
GRADE: Grading of Recommendations Assessment, Development and Evaluation
HAI: Healthcare-associated infection
MECIR: Methodological Expectations of Cochrane Intervention Reviews
MRSA: Meticillin- (or methicillin-) resistant *Staphylococcus aureus*
NICE: National Institute for Health and Care Excellence
NIH: National Institutes of Health (NIH)
NRS: Non-randomised study
RCT: Randomised controlled trial
RevMan: Review Manager
SAGER: Sex and Gender Equity in Research
SGAT-SR: Sex and gender appraisal tool for systematic reviews
SGBA: Sex- and gender-based analysis
SR: Systematic review
UCL: University College London
USA: United States of America
WHO: World Health Organization
