# Supplementary material for: Consideration of sex and gender in Cochrane reviews of interventions for preventing healthcare-associated infections: a methodology study
Source: BMC Health Serv Res. 2019 Mar 15;19:169. doi: 10.1186/s12913-019-4001-9 (PMC6419810; doi:10.1186/s12913-019-4001-9)
Supplement: Supplementary file 1 — Glossary of terms. (DOCX 28 kb) [file 12913_2019_4001_MOESM1_ESM.docx]

| **Additional file 1 - Glossary of terms** | |
| --- | --- |
| **Controlled clinical trial (CCT)** | Type of non-randomised study in which the investigator allocates prospectively each participant to an intervention or to a control group (or more) using a process that is clearly not random (for example, allocation by judgement of the clinician, or by preference of the participant) [1]. |
| **Experimental study** | A study in which an intervention is deliberately introduced by the researcher to observe its effects [2]. Therefore, it is a study in which the investigator actively intervenes to test a hypothesis [3]. It is also called experiment. |
| **Gender** | The socially constructed roles, behaviours and identities of female, male and gender diverse people [4]. It influences how people perceive themselves and each other, how they behave and interact and the distribution of power and resources in society. Gender is usually incorrectly conceptualized as a binary factor (female/male). However, In reality, there is a spectrum of gender identities and expressions defining how individuals identify themselves and express their gender. Definition taken from SAGER guidelines [5]. |
| **Gender-based analysis** | An analytical tool that systematically integrates a gender perspective into the development of policies, programmes and legislation, as well as planning and decision-making processes. It helps to identify and clarify the differences between women and men and boys and girls and demonstrates how these differences affect health status, access to, and interaction with, the health care system [6]. Definition taken from SAGER guidelines [5]. |
| ***GRADE*** | *GRADE (Grading of Recommendations Assessment, Development and Evaluation*) is a common, sensible and transparent approach to grading quality (or certainty) of evidence and strength of recommendations [7] |
| **Healthcare-associated infection (HAI)** | Infection acquired as a result of the delivery of healthcare [8]. |
| **Health inequality** | Differences in health status or in the distribution  of health determinants between different population  groups (e.g., racial, ethnic, sex, gender, sexual orientation, or socioeconomic groups) [9]. |
| **Health inequity** | Avoidable and unfair health inequalities (differences) across socioeconomic, demographic and geographic factors [10, 11]. |
| **Non-randomised study (NRS)** | Any quantitative study estimating the effectiveness of an intervention (harm or benefit) that does not use randomisation to allocate units to comparison groups (including studies where ‘allocation’ occurs in the course of usual treatment decisions or peoples’ choices, i.e. studies usually called ‘observational’). To avoid ambiguity, the term should be substantiated using a description of the type of question being addressed [3]. |
| **Non-randomised intervention study** | A comparative study of an experimental intervention against some control intervention (or no intervention) that is not a randomised controlled trial. There are many possible types of non-randomised intervention studies, including cohort studies, case-control studies, controlled before-and-after studies, interrupted-time-series studies and controlled trials that do not use appropriate randomisation strategies (sometimes called quasi-randomised studies) [3]. |
| **Observational study** | A study in which the investigators do not seek to intervene, and simply observe the course of events. Changes or differences in one characteristic (e.g. whether or not people received the intervention of interest) are studied in relation to changes or differences in other characteristic(s) (e.g. whether or not they died), without action by the investigator [3]. |
| **PROSPERO** | International database of prospectively registered systematic reviews in health and social care, welfare, public health, education, crime, justice, and international development, where there is a health related outcome |
| **Quasi- randomised controlled trial (Quasi-RCT)** | Type of non-randomised study in which the investigator allocates prospectively each participant to an intervention or to a control arm (or more) using a process that attempts but does not achieve true randomisation [12]. Therefore, it uses methods of allocating people to a trial that are not random, but were intended to produce similar groups when used to allocate participants [3]. Quasi-random methods include: allocation by the person's date of birth, by the day of the week or month of the year, by a person's medical record number, or just allocating every alternate person [3]. |
| **Randomised controlled trial (RCT)** | An experiment in which two or more interventions, possibly including a control intervention or no intervention, are compared by being randomly allocated to participants [3]. Examples of processes of random allocation are the use of software for random number generation or coin flips [13]. |
| **Sex** | A set of biological attributes in humans and animals that are associated with physical and physiological features including chromosomes, gene expression, hormone function and reproductive/sexual anatomy [4]. Sex is usually categorized as female or male, although there is variation in the biological attributes that constitute sex and how those attributes are expressed. Definition taken from SAGER guidelines [5]. |
| **Sex/gender** | A “domain of complex phenomena that are simultaneously biological and social […]” [14]. This term has been used “to highlight this socio-biological intertwinement […]”[15] or “to acknowledge the interrelationships among […]” sex and gender [16]. |
| **Sex/Gender based analysis (SGBA)** | Analytical approach that integrates a sex and gender perspective into the development of health research, policies and programmes, as well as health planning and decision-making processes. It helps to identify and clarify the differences between women and men and boys and girls, and demonstrates how these differences affect health status, access to, and interaction with, the health care system [5, 17]. |
| **Sex-disaggregated data** | Data that are collected and presented separately on men and women [18]. Definition taken from SAGER guidelines [5]. |
| **Systematic review** | Review of a clearly formulated question that uses systematic and explicit methods to identify, select, and critically appraise relevant research, and to collect and analyse data from the included studies. Systematic reviews are important tools to transfer research knowledge into policy, program, regulatory, and clinical practice (17, 37). |

**Reference list**

1. Reeves BC, Deeks JJ, Higgins JPT, Wells GA: **Chapter 13: Including non-randomised studies. In: Higgins JPT, Green S (editors). Cochrane Handbook for Systematic Reviews of Interventions. Version 5.1.0 [updated March 2011]. The Cochrane Collaboration, 2011**. In: *Available from wwwcochrane-handbookorg.* 2011.

2. Shadish WR, Cook TD, Campbell DT: **Experimental and quasi-experimental designs for generalized causal inference**. Boston (MA): Houghton Mifflin; 2002.

3. **Glossary | Cochrane Community** [<http://community.cochrane.org/glossary#letter-O>]

4. Cohen S, Banister E, for the CIHR Institute of Gender and Health: **What a difference sex and gender make: a gender, sex and health research casebook**. In*.*; 2012.

5. Heidari S, Babor TF, Castro PD, Tort S, Curno M: **Sex and Gender Equity in Research: rationale for the SAGER guidelines and recommended use**. *Research Integrity and Peer Review* 2016, **1**(1):2.

6. Raphael D, Bryant T, Rioux MH: **Staying alive : critical perspectives on health, illness, and health care**. Toronto: Canadian Scholars' Press; 2006.

7. **GRADE Working Group** [<http://www.gradeworkinggroup.org/>]

8. Pratt RJ, Pellowe C, Loveday HP, Robinson N, Smith GW, Barrett S, et al: **The epic project: developing national evidence-based guidelines for preventing healthcare associated infections. Phase I: Guidelines for preventing hospital-acquired infections. Department of Health (England)**. *The Journal of Hospital Infection* 2001, **47 Suppl**:S3-82.

9. **Health Impact Assessment (HIA) - Glossary of terms used** [<http://www.who.int/hia/about/glos/en/index1.html>]

10. Whitehead M: **The concepts and principles of equity and health**. *Int J Health Serv* 1992, **22**(3):429-445.

11. **Inequity and inequality in health** [<http://www.globalhealtheurope.org/index.php/resources/glossary/values/179-inequity-and-inequality-in-health.html>]

12. Higgins JPT, Altman DG, Sterne JAC: **Chapter 8: Assessing risk of bias in included studies**. In: *In: Higgins JPT, Green S (editors) Cochrane Handbook for Systematic Reviews of Interventions Version 510 (updated March 2011) The Cochrane Collaboration, 2011 Available from wwwcochrane-handbookorg.* 2011.

13. **Cochrane Handbook for Systematic Reviews of Interventions  Version 5.1.0 [updated March 2011]** [[www.handbook.cochrane.org](http://www.handbook.cochrane.org)]

14. Springer KW, Mager Stellman J, Jordan-Young RM: **Beyond a catalogue of differences: a theoretical frame and good practice guidelines for researching sex/gender in human health**. *Soc Sci Med* 2012, **74**(11):1817-1824.

15. Kaiser A, Haller S, Schmitz S, Nitsch C: **On sex/gender related similarities and differences in fMRI language research**. *Brain Res Rev* 2009, **61**(2):49-59.

16. **Challenging "dis-ease": sex, gender and systematic reviews in health** [<http://www.cihr-irsc.gc.ca/e/44734.html#a04>]

17. **Health Canada** [<https://www.canada.ca/en/health-canada.html> ]]

18. UNESCO: **UNESCO’S Gender Mainstreaming Implementation Framework (GMIF) for 2002-2007. Accessed at:**[**http://unesdoc.unesco.org/images/0013/001318/131854e.pdf**](http://unesdoc.unesco.org/images/0013/001318/131854e.pdf). In*.*; 2003.
