# Supplementary material for: Consideration of sex and gender in Cochrane reviews of interventions for preventing healthcare-associated infections: a methodology study
Source: BMC Health Serv Res. 2019 Mar 15;19:169. doi: 10.1186/s12913-019-4001-9 (PMC6419810; doi:10.1186/s12913-019-4001-9)
Supplement: Supplementary file 2 — Interventions defined as eligible. (DOCX 13 kb) [file 12913_2019_4001_MOESM2_ESM.docx]

**Additional file 2 -** Interventions defined as eligible

| Eligible interventions |
| --- |
| 1. Regulatory interventions  2. Organizational measures  3. Education & training  4. Antimicrobial stewardship  5. HAIs surveillance  6. Monitoring, audit and feedback  7. Decontamination of surfaces, medical equipment, environmental infection control  8. Hand hygiene  9. Patient and healthcare personnel hygiene (not hand hygiene)  10. Physical barriers to reduce the transmission of microorganisms  11. Decolonisation  12. Prevention of infections associated to vascular accesses  13. Interventions to prevent infections associated to surgery  14. Interventions to prevent HAIs associated to non-surgical procedures  15. Interventions to prevent HAIs associated to dental procedures  16. Interventions to prevent respiratory HAIs (due to mechanical ventilation or not)  17. Interventions to prevent urinary catheter-associated infection  18. Interventions to prevent infection in non-surgical wounds  19. Interventions to prevent HAIs in the context of a medical condition  20. Interventions to prevent HAIs by physiotherapy and rehabilitation techniques  21. Other interventions not included in the previous sections |
