# Supplementary material for: Consideration of sex and gender in Cochrane reviews of interventions for preventing healthcare-associated infections: a methodology study
Source: BMC Health Serv Res. 2019 Mar 15;19:169. doi: 10.1186/s12913-019-4001-9 (PMC6419810; doi:10.1186/s12913-019-4001-9)
Supplement: Supplementary file 3 — Search strategy. (DOCX 15 kb) [file 12913_2019_4001_MOESM3_ESM.docx]

**Additional file 3** – Search strategy

**Database:** Cochrane Database of Systematic Reviews (CDSR), accessed via The Cochrane Library (issue 4, 2017) (http://www.cochranelibrary.com/)

**Date accessed:** 15/04/2017

**Search terms:** search all text with *

**Search limits:**

- Database: Cochrane reviews (only reviews)
- Dates of publication: between 1992 and 2016

**Procedure:** all the *CDSR* records were exported to the reference management software *Endnote* X7.7.1 [1] where Cochrane reviews defined as ‘published’, ‘active’, and ‘intervention review’ were identified and exported to *EPPI-Reviewer 4* software [2].

**Reference List**

1. Clarivate A: **EndNote version X7.7.1, software for reference management.** New York: Thomsom Reuters; 2016.

2. Thomas J BJ, Graziosi S,: **EPPI-Reviewer 4.0: software for research synthesis. Version 4.0.** London: Institute of Education; 2010.
