# Supplementary material for: Consideration of sex and gender in Cochrane reviews of interventions for preventing healthcare-associated infections: a methodology study
Source: BMC Health Serv Res. 2019 Mar 15;19:169. doi: 10.1186/s12913-019-4001-9 (PMC6419810; doi:10.1186/s12913-019-4001-9)
Supplement: Supplementary file 4 — SGAT-SR template and guidance. (DOCX 30 kb) [file 12913_2019_4001_MOESM4_ESM.docx]

**Additional file 4: SGAT-SR template and guidance**

This file details the domains of the Sex & Gender Appraisal Tool for Systematic reviews (SGAT-SR)^[[1]](#footnote-1)^ and the guidance agreed by our team to guide the assessments.

**0. Definition if the review was focused on one sex**

**Not sex focused**

- Not sex focused
- Not sex focused-Typically male
- Not sex focused-Typically female
  - *Example: breast cancer*

**Sex MALE ONLY**

*Examples: prostate; testicular cancer;*

**Sex FEMALE only**

*Examples: Pregnancy; Gynaecological problems*

**1. Review section: Background**

**1.1. Are the terms sex and gender used in the background?**

*Terms related with sex and gender: sex, gender, sex/gender, male/s, or female/s, man, men, woman, women, boy/s, girl/s.*

- - Yes, review met criteria
  - No, review did not meet criteria
  - Item was not applicable to the review
    - Sex focused review
    - Sex female only or sex male only
  - Unable to determine

**1.2. Are sex/gender identified as relevant or not to review question?**

*This item assesses if the relevance/irrelevance of sex/gender was mentioned.*

- - Yes, review met criteria

*The relevance or not relevance of sex or gender or both was mentioned.*

*Sex/gender were identified as relevant for males/men and females/women*

*Example: "It is one of the malignant tumours that causes the most deaths internationally, being the second most common cancer in men, after prostate cancer, and in women, after breast cancer."*

- - No, review did not meet criteria

*If there is no mention to the relevance or not relevance of sex or gender (none of them was mentioned) and no evidence was provided.*

- - Item was not applicable to the review
    - Sex focused review
  - Unable to determine

*The review provides data about sex/gender differences, but it does not explicitly states that sex/gender is important. Example: “The actual incidence of acute appendicitis varies, the overall lifetime risk for acute appendicitis is 6-20% (Blewett 1995; Addiss 1990). Addiss reported 8.6% for males and 6.7% for females, compared to an overall life time risk for appendectomy of 12% (males) and 23.1% (females) in US. (Andersen 2005; DOI: 0.1002/14651858.CD001439.pub2).”*

- - - Sex/gender identified as relevant or not for women/females but no reference to relevance in men/males

*Example: Breast cancer remains the second most common cancer in women with a reported mortality reaching 460,000 deaths worldwide in 2008 (WHO 2011).*

- - - Sex/gender identified as relevant or not for men/males but no reference to relevance in women/females
    - Data about sex/gender differences are provided but without explicitly stating that sex/gender is important

*“The actual incidence of acute appendicitis varies, the overall lifetime risk for acute appendicitis is 6-20% (Blewett 1995; Addiss 1990). Addiss reported 8.6% for males and 6.7% for females, compared to an overall life time risk for appendectomy of 12% (males) and 23.1% (females) in US. (Andersen 2005; DOI: 10.1002/14651858.CD001439.pub2).”*

**1.3. Does background discuss why sex/gender differences may be expected?**

*Is there evidence in the literature to suggest sex/gender differences in the subject area?*

*Is it likely that the intervention will affect men and women differently?*

*Is the intervention widely applied across broad population groups?*

*If so, have differences by sex or gender been examined?*

***Context:***

*Did the review consider sociocultural understandings of sex/gender as part of context? This may include developing an analytic framework/logic model to define assumptions about sex/gender across intervention, comparator and outcomes.*

*Example: how gender roles, norms, identity, and gender relations can influence symptoms, implementation and health.*

***Population:***

*Did the review consider ways in which other inclusion criteria at the review or primary study level, such as age or ethnicity, may interact with sex/gender.*

*Example: age of onset of disease or complications related to a condition may differ for men and women – thus age-based inclusion criteria may favour one sex.*

***Intervention/Comparator:***

*Did the review consider whether sex/gender interacts with aspects of the intervention (e.g. treatment delivery; likelihood of offering treatment; acceptability), and whether this is important for the review question?*

*Example: Meader et al (2010) conducted sex-disaggregated analyses and found that trials of psychosocial interventions that enrolled mainly females were more effective than trials that included both men and women in each group.*

***Outcomes:***

*If women and men are included, is it also necessary to consider different outcomes?*

*Consider whether the outcomes are relevant for both women and men.*

*Does the condition or its complications manifest differently in men and women – and do the selected outcomes for the review capture both of these scenarios?*

*Example: At the same viral load, women have a higher risk of progression to AIDS as compared to men consider this if using thresholds or cut off points for viral load measures (Farzadegan et al., 1998).*

- - Yes, review met criteria

*At least differences of sex or gender or both in key factors were mentioned.*

*Example: differences in incidence.*

- - No, review did not meet criteria

*There is no mention to sex or gender at all.*

*The review states that there may be differences but no justification was provided.*

- - Item was not applicable to the review
    - Sex focused review
  - Unable to determine

*The review justified why sex differences may/may NOT be expected, but justification was unclear.*

*Example: categories of gender used for sex.*

*Example: the term sex was used but no definition was provided so it is not clear if the review discussed about sex or gender.*

**2. Review section: Objectives**

**2.1. Are the terms sex, gender, male, or female used in objectives?**

*Terms related: sex, gender, male/s, m, female/s, f, man, men, woman, women, boy/s, girl/s used in objectives*

- - Yes, review met criteria
  - No, review did not meet criteria
  - Item was not applicable to the review
    - Sex focused review
  - Unable to determine

**3. Review section: Criteria for inclusion/exclusion**

**3.1. Do the review’s inclusion/exclusion criteria consider sex/gender differences?**

*We interpreted this question as follows: Were sex/gender used as criterion for deciding on study eligibility?*

- - Yes, review met criteria

*Example: Breast cancer screening reviewers stated that “Only women were eligible”.*

- - No, review did not meet criteria

*Example: “No restrictions to gender"*

- - Item was not applicable to the review
    - Sex focused review
  - Unable to determine

**3.2. Was there justification or explanation for the exclusion of some groups?**

*Did the review justify or explain why some population groups are excluded from the review based on sex/gender?*

*We interpreted that this item refers only to the exclusion of some groups based on sex, gender or both (it is not clear in the original question).*

*Example: If women or men as a group were excluded, a rationale should be provided.*

*Example: If subgroups of men or women were excluded (e.g., older men) a rationale should be provided*

- - Yes, review met criteria
  - No, review did not meet criteria

*When there is no justification about exclusion/inclusion of groups based on sex-gender, independently of the inclusion-exclusion of these groups. For example, a review did not exclude any group based on sex but it did not provide any justification for this. Add as comment: Comment: The review did not exclude groups based on sex/gender. Comment: The review did not justify this.*

- - - The review didn’t exclude based on sex/gender but didn’t provide an explanation on sex or gender
    - The review EXCLUDED based on sex/gender but didn’t provide an explanation on the exclusion
  - Item was not applicable to the review
    - Sex focused review
  - Unable to determine

**4. Review section: Methods**

*For this section assumed that the tool refers to what was planned to be done (“Methods”) or what was finally done (“Results”). Therefore, we assessed the “methods section” or the “results section”. For this section we assessed sex only (it is not clear if the tool refers to sex, gender or both).*

*If the review did not report the “data extraction form” and no relevant information was reported in the text about the data extraction, we answered “No, review did not meet criteria”.*

**4.1. Does the review examine whether outcome measures are different for males and females?**

- - Yes, review met criteria
  - No, review did not meet criteria
  - Item was not applicable to the review
    - Sex focused review
  - Unable to determine

**4.2. Did the review extract data by sex?**

*We assessed the “Data collection and analysis” and the “Results” sections (but not the table of included studies). If the review included both men and women, data should be extracted by sex, that is, extract sex-disaggregated data and contact authors for sex-disaggregated data and sex of participants if not reported.*

- - Yes, review met criteria
    - *The data extraction form included sections to extract data by sex; or*
    - *Data were extracted by sex; or*
    - *The review planned to extract data by sex but there was no data to extract due to there were no included studies.*
  - No, review did not meet criteria

*No included studies but there was no plan to extract data by sex*

*Comment: No included studies*

- - - The review planned to describe the sex of the samples
    - Not reported if the review planned to describe the sex of the samples
    - The review did not plan to report the sex of the samples
  - Item was not applicable to the review

*This is the case only one sex or gender was eligible.*

- - - Sex focused review
  - Unable to determine
    - Empty review + No information about the data extraction template

**4.3. Did the review extract data on sex of withdrawals and dropouts?**

*Read all the review from “Methods”, normally “Methods”, “Results”, and “Table of characteristics of included studies”. Were Data on withdrawals and dropouts were reported disaggregated by sex. (SAGER)*

- - Yes, review met criteria

*The data extraction form included sections to extract data by sex for withdrawals and dropouts. The review presented withdrawals and dropouts by sex*

- - No, review did not meet criteria

*If there is no information in the review and we do not have access to the data extraction form we will assume “No”. No included studies but there was no plan to extract data by sex*

- - Item was not applicable to the review
    - Sex focused review
  - Unable to determine
    - Empty review + No information about the data extraction template

**4.4. In cases where sex/gender is used as a proxy for other measures (i.e., weight), is there an explanation for this approach?**

- - Yes, review met criteria
  - No, review did not meet criteria
  - Item was not applicable to the review

*Example: cases where sex/gender is NOT used as a proxy for other measures*

- - - Sex focused review
  - Unable to determine

**4.5. Were any subgroup analyses completed?**

*This section refers to subgroup analyses done on any factor, sex or not. Judge only subgroup analysis completed. Therefore, do not answer “yes” if subgroup analysis were planned but not done.*

- - Yes, review met criteria

*Subgroup analyses were completed.*

*No need to quote or summarise them.*

- - No, review did not meet criteria
    - Added: No subgroup analysis was planned
    - Added: Subgroup analyses were planned, but not completed
  - Item was not applicable to the review
    - Sex focused review
  - Unable to determine

**4.6. Were subgroup analyses by sex completed?**

- - Yes, review met criteria
    - Subgroup analyses by sex were completed.
  - No, review did not meet criteria
    - Added: No subgroup analysis was planned
    - Added: At least one subgroup analysis was planned but sex was not considered

*No need to quote or summarise them*

- - - Added: Subgroup analysis by sex was planned but not completed
    - Subgroup analysis by sex not done & not known what were the factors planned
  - Item was not applicable to the review
    - Sex focused review
  - Unable to determine

**5. Review section: Results and analysis**

*For questions 5.1 to 5.4: If no studies were included in the review choose*

*“Item was not applicable to review”*

*Add: Comment: No included studies*

**5.1. Do results distinguish between findings for males/females?**

*Data should be routinely presented disaggregated by sex and gender (SAGER). Do not consider the information presented in the “Included studies” section. For example: The proportion of females ranged between 20.7% and 100% in the six trials that reported this information (Ishibashi 2009; Ishida 2001; Morimoto 2002; Saadeddin 2005; Stone 2010; Vuorisalo 1998).”*

- - Yes, review met criteria
  - No, review did not meet criteria
    - Added: Review did NOT extract data by sex
    - Added: Review extracted data by sex, results didn’t distinguish between findings for males/females, and this was JUSTIFIED
    - Added: Review extracted data by sex, results didn’t distinguish between findings for males/females, and this was NOT JUSTIFIED
  - Item was not applicable to the review
    - Empty review
    - Sex focused review
  - Unable to determine

**5.2. Does the review report conclusions (of effectiveness, efficacy, safety) that are different for men and women?**

*Look for information in the results section. If there are no results reported in the review answer “Item was not applicable to review” and explain this as a comment.*

- - Yes, review met criteria
  - No, review did not meet criteria

*If 5.1 was “No, review did not meet criteria”, 5.2 “No, review did not meet criteria” should be no*

- - Item was not applicable to the review
    - Empty review
    - Sex focused review
  - Unable to determine

**5.3. If adverse effects are reported, is information sex disaggregated?**

*If there are no results reported in the review, answer “Item was not applicable to review” and explain this as a comment.*

- - Yes, review met criteria
  - No, review did not meet criteria

*Example: adverse effects were planned to be measured in the review, but they finally were not reported because there were no included studies measuring this outcome.*

*Example: adverse effects not planned to be measured in the review.*

- - Item was not applicable to the review

*Example: adverse effects were planned to be measured in the review but they finally were not reported because there were no included studies measuring this outcome.*

*Example: adverse effects not planned to be measured in the review*

- - - Empty review
    - Adverse effects not planned in the review
    - Adverse effects planned in the review but not reported as no included studies reported adverse effects
    - Sex focused review
  - Unable to determine

**5.4. Does review note that subgroup analyses by sex could not be done?**

*If sub-group analysis by sex and/or gender could not be completed, did the review explain why?*

*Example: insufficient data in primary studies*

- - Yes, review met criteria
    - Due to insufficient data in primary studies (in a review that is not empty)
    - Due to empty review
  - No, review did not meet criteria

*Example: the review was not empty and sub-group analysis by sex and/or gender were not planned. Add as comment: Subgroup analysis by sex was not planned.*

- - Item was not applicable to the review

*Sub-group analysis by sex and/or gender were not planned. 4.6 = NO + “No Subgroup analysis planned”. 4.6 = NO + “Subgroup analysis were planned but sex was not considered”*

- - - 4.6. Were subgroup analyses by sex completed?= NO + “No Subgroup analysis planned"
    - 4.6. Were subgroup analyses by sex completed?= NO + “Subgroup analysis were planned but sex was not considered"
    - 4.6. Were subgroup analyses by sex completed?= YES
    - Sex focused review
  - Unable to determine

**6. Review section: Discussion and conclusion**

**6.1. Does the review report that primary studies analysed or failed to analyse results by sex?**

- - Yes, review met criteria
  - No, review did not meet criteria
  - Item was not applicable to the review
    - Empty review
    - Sex focused review
  - Unable to determine

**6.2. Does the review address sex/gender implications for clinical practice?**

- - Yes, review met criteria

*Answer yes if a sex and gender analysis was not conducted, and the rationale was given.*

- - No, review did not meet criteria
  - Item was not applicable to the review
    - Sex focused review
  - Unable to determine

**6.3. Does the review address sex/gender implications for policy and regulation?**

- - Yes, review met criteria
  - No, review did not meet criteria
  - Item was not applicable to the review
    - Sex focused review
  - Unable to determine

**6.4. Does the review address sex/gender implications for research?**

- - Yes, review met criteria
  - No, review did not meet criteria
  - Item was not applicable to the review
    - Sex focused review
  - Unable to determine

**7. Review section: Table of included studies**

*We answered these questions considering if the review attempted to report information on sex AND gender in the samples recruited in the studies. Therefore, the review should attempt to report the following information:*

1. *For sex:*
   1. *Number of both female and male participants in included studies.*
   2. *Report if data are not available*
2. *For gender:*
   1. *Number of both men and women in included studies.*
   2. *Report if data are not available*

*We focused on the information that the review tried to report in the table of included studies. For example, if the review tried to report on sex/gender but the review reported that this information was not available in the study, we answered “Yes, review met criteria”.*

**7.1. Does the description of included studies give detailed information on sex/gender of the study samples?**

- - Yes, review met criteria
    - 7.1.1. and 7.1.2. Yes
  - No, review did not meet criteria (at least 7.1.1. or 7.1.2. are NO)

*No description of the samples included, only the inclusion-exclusion criteria were reported/. Add as comment: Comment: No description of the samples included, only the inclusion-exclusion criteria were reported.*

- - Item was not applicable to the review
    - 7.1.1. and 7.1.2. Not applicable
      - Empty review
      - Sex focused review
  - Unable to determine
    - - At least 7.1.1 or 7.1.2. Unable to determine without any of them being NO

**7.1.1. Detailed information on SEX of the study samples**

- - Yes, review met criteria
    - There was a correct definition for sex
    - For all the studies, the review reported the heading “Sex” and what was found (for example, “information not available”)
    - Terms used were correct (for sex: male and female)
  - No, review did not meet criteria

*The review did NOT provide information for sex. No description of the samples included, only the inclusion-exclusion criteria were reported. Add as comment: Comment: No description of the samples included, only the inclusion-exclusion criteria were reported*

- - - The review did NOT provide information for sex for ANY study
    - The review did NOT report the Heading “Sex” and what was found (for example, “information not available”) for ALL the studies

*For example, three tables report on sex but the remainder four do not report any information on sex*

- - - Only the inclusion-exclusion criteria were reported
    - Only if sex was balanced at baseline was reported

*Example: "Study and control groups were well matched in terms of age, sex and number of episodes of tonsillitis prior to surgery"*

- - Item was not applicable to the review

*Comment: No included studies*

- - - Empty review
    - Sex focused review
  - Unable to determine

*Only if the review reported the Heading “Sex” and what was found (for example, “information not available”) for all the studies.*

- - - Sex heading and composition for all the studies BUT NO definitions for sex provided

*Example: Sex: 92 males. And no definition for sex. Therefore, it is not clear if this refer to sex or gender.*

- - - Sex terms reported for all the included used but not clarified if they refer to sex

*Example: the table reports “88 males and 78 females enrolled in the study.”*

- - - Terms used incorrectly (men or women for sex)

*Numbers reported but we do not know if they refer to sex or gender. Add comment: Comment: Not reported if the numbers reported refer to sex or gender. Example: the table reports “88 males and 78 females enrolled in the study.” There is mention to sex/gender but the terminology was unclear or not correct. Example: “Sex: 92 men.”*

**7.1.2. Detailed information on GENDER of the study samples**

- - Yes, review met criteria
    - There was a correct definition for gender
    - For all the studies, the review reported the Heading “Gender” and what was found (for example, “information not available”)
    - Terms used were correct (for gender: men and women)
  - No, review did not meet criteria

*The review did NOT provide information for gender. No description of the samples included, only the inclusion-exclusion criteria were reported. Add as comment: Comment: No description of the samples included, only the inclusion-exclusion criteria were reported.*

- - - The review did NOT provide information for gender for ANY study
    - The review did NOT report the Heading “gender” and what was found (for example, “information not available”) for ALL the studies.

*For example, three tables report on sex but the remainder four do not report any information on sex.*

- - - Only the inclusion-exclusion criteria were reported
    - Only if gender was balanced at baseline was reported

*Example: "Study and control groups were well matched in terms of age, gender and number of episodes of tonsillitis prior to surgery".*

- - Item was not applicable to the review

*Only if the review reported the Heading “Gender” and what was found (for example, “information not available”) for all the studies.*

- - - Empty review
    - Sex focused review
  - Unable to determine
    - Gender heading and composition for all the studies BUT NO definitions for gender provided

*Example: gender: 92 men. And no definition for gender. Therefore, it is not clear if this refer to sex or gender.*

- - - Terms used incorrectly (male or female for gender)

*Numbers reported, but we do not know if this refers to sex or gender*

*Example: the table reports “88 males and 78 females enrolled in the study.” There is mention to sex/gender but the terminology was unclear or not correct. Example: “gender: 92 male.” For example, 3 tables report on gender but the remainder 4 do not report any information on gender.*

- - - Gender terms reported for all the studies but not clarified if they refer to gender

*Example: the table reports “88 men and 78 women enrolled in the study.”*

1. Doull M, Runnels VE, Tudiver S, Boscoe M: **Appraising the evidence: applying sex- and gender-based analysis (SGBA) to Cochrane systematic reviews on cardiovascular diseases.** *J Womens Health (Larchmt)* 2010, **19:**997-1003 [↑](#footnote-ref-1)
