# Supplementary material for: Consideration of sex and gender in Cochrane reviews of interventions for preventing healthcare-associated infections: a methodology study
Source: BMC Health Serv Res. 2019 Mar 15;19:169. doi: 10.1186/s12913-019-4001-9 (PMC6419810; doi:10.1186/s12913-019-4001-9)
Supplement: Supplementary file 5 — List of included reviews. (DOCX 21 kb) [file 12913_2019_4001_MOESM5_ESM.docx]

**Additional file 5: List of included reviews**

1. Alkhawaja S, Martin C, Butler Ronald J, Gwadry-Sridhar F: **Post-pyloric versus gastric tube feeding for preventing pneumonia and improving nutritional outcomes in critically ill adults**. *Cochrane Database of Systematic Reviews* 2015(8).

2. Andersen Betina R, Kallehave Finn L, Andersen Henning K: **Antibiotics versus placebo for prevention of postoperative infection after appendicectomy**. *Cochrane Database of Systematic Reviews* 2005(3).

3. Arnold Sandra R, Straus Sharon E: **Interventions to improve antibiotic prescribing practices in ambulatory care**. *Cochrane Database of Systematic Reviews* 2005(4).

4. Arora Ramandeep S, Roberts R, Eden Tim O, Pizer B: **Interventions other than anticoagulants and systemic antibiotics for prevention of central venous catheter-related infections in children with cancer**. *Cochrane Database of Systematic Reviews* 2010(12).

5. Arrowsmith Vickie A, Taylor R: **Removal of nail polish and finger rings to prevent surgical infection**. *Cochrane Database of Systematic Reviews* 2014(8).

6. Barajas-Nava Leticia A, López-Alcalde J, Roqué i Figuls M, Solà I, Bonfill Cosp X: **Antibiotic prophylaxis for preventing burn wound infection**. *Cochrane Database of Systematic Reviews* 2013(6).

7. Bo L, Li J, Tao T, Bai Y, Ye X, Hotchkiss Richard S, Kollef Marin H, Crooks Neil H, Deng X: **Probiotics for preventing ventilator-associated pneumonia**. *Cochrane Database of Systematic Reviews* 2014(10).

8. Bradford Natalie K, Edwards Rachel M, Chan Raymond J: **Heparin versus 0.9% sodium chloride intermittent flushing for the prevention of occlusion in long term central venous catheters in infants and children**. *Cochrane Database of Systematic Reviews* 2015(11).

9. Brand M, Bizos D, O'Farrell P, Jr.: **Antibiotic prophylaxis for patients undergoing elective endoscopic retrograde cholangiopancreatography**. *Cochrane Database of Systematic Reviews* 2010(10).

10. Brand M, Grieve A: **Prophylactic antibiotics for penetrating abdominal trauma**. *Cochrane Database of Systematic Reviews* 2013(11).

11. Brass P, Hellmich M, Kolodziej L, Schick G, Smith Andrew F: **Ultrasound guidance versus anatomical landmarks for subclavian or femoral vein catheterization**. *Cochrane Database of Systematic Reviews* 2015(1).

12. Bravo Zuñiga Jessica I, Loza Munárriz C, López-Alcalde J: **Isolation as a strategy for controlling the transmission of hepatitis C virus (HCV) infection in haemodialysis units**. *Cochrane Database of Systematic Reviews* 2016(8).

13. Brignardello-Petersen R, Carrasco-Labra A, Araya I, Yanine N, Cordova Jara L, Villanueva J: **Antibiotic prophylaxis for preventing infectious complications in orthognathic surgery**. *Cochrane Database of Systematic Reviews* 2015(1).

14. Cooper Fergus P, Alexander Cameron E, Sinha S, Omar Muhammad I: **Policies for replacing long-term indwelling urinary catheters in adults**. *Cochrane Database of Systematic Reviews* 2016(7).

15. D'Amico R, Pifferi S, Torri V, Brazzi L, Parmelli E, Liberati A: **Antibiotic prophylaxis to reduce respiratory tract infections and mortality in adults receiving intensive care**. *Cochrane Database of Systematic Reviews* 2009(4).

16. Dhiwakar M, Clement WA, Supriya M, McKerrow W: **Antibiotics to reduce post-tonsillectomy morbidity**. *Cochrane Database of Systematic Reviews* 2012(12).

17. Dumville Jo C, Gray Trish A, Walter Catherine J, Sharp Catherine A, Page T, Macefield R, Blencowe N, Milne Thomas K, Reeves Barnaby C, Blazeby J: **Dressings for the prevention of surgical site infection**. *Cochrane Database of Systematic Reviews* 2016(12).

18. Dumville Jo C, McFarlane E, Edwards P, Lipp A, Holmes A, Liu Z: **Preoperative skin antiseptics for preventing surgical wound infections after clean surgery**. *Cochrane Database of Systematic Reviews* 2015(4).

19. Ejemot-Nwadiaro Regina I, Ehiri John E, Arikpo D, Meremikwu Martin M, Critchley Julia A: **Hand washing promotion for preventing diarrhoea**. *Cochrane Database of Systematic Reviews* 2015(9).

20. Fernandez R, Griffiths R: **Water for wound cleansing**. *Cochrane Database of Systematic Reviews* 2012(2).

21. Flodgren G, Conterno Lucieni O, Mayhew A, Omar O, Pereira Cresio R, Shepperd S: **Interventions to improve professional adherence to guidelines for prevention of device-related infections**. *Cochrane Database of Systematic Reviews* 2013(3).

22. Flodgren G, Gonçalves-Bradley Daniela C, Pomey M-P: **External inspection of compliance with standards for improved healthcare outcomes**. *Cochrane Database of Systematic Reviews* 2016(12).

23. Foon R, Toozs-Hobson P, Latthe P: **Prophylactic antibiotics to reduce the risk of urinary tract infections after urodynamic studies**. *Cochrane Database of Systematic Reviews* 2012(10).

24. Gavin Nicole C, Webster J, Chan Raymond J, Rickard Claire M: **Frequency of dressing changes for central venous access devices on catheter-related infections**. *Cochrane Database of Systematic Reviews* 2016(2).

25. Ge X, Cavallazzi R, Li C, Pan Shu M, Wang Ying W, Wang F-L: **Central venous access sites for the prevention of venous thrombosis, stenosis and infection**. *Cochrane Database of Systematic Reviews* 2012(3).

26. Gillespie William J, Walenkamp Geert H: **Antibiotic prophylaxis for surgery for proximal femoral and other closed long bone fractures**. *Cochrane Database of Systematic Reviews* 2010(3).

27. Glenny A-M, Oliver R, Roberts Graham J, Hooper L, Worthington Helen V: **Antibiotics for the prophylaxis of bacterial endocarditis in dentistry**. *Cochrane Database of Systematic Reviews* 2013(10).

28. Gosselin Richard A, Roberts I, Gillespie William J: **Antibiotics for preventing infection in open limb fractures**. *Cochrane Database of Systematic Reviews* 2004(1).

29. Gould Dinah J, Moralejo D, Drey N, Chudleigh Jane H: **Interventions to improve hand hygiene compliance in patient care**. *Cochrane Database of Systematic Reviews* 2010(9).

30. Griffiths R, Fernandez R: **Strategies for the removal of short-term indwelling urethral catheters in adults**. *Cochrane Database of Systematic Reviews* 2007(2).

31. Gurusamy Kurinchi S, Koti R, Toon Clare D, Wilson P, Davidson Brian R: **Antibiotic therapy for the treatment of methicillin-resistant Staphylococcus aureus (MRSA) in non surgical wounds**. *Cochrane Database of Systematic Reviews* 2013(11).

32. Gurusamy Kurinchi S, Koti R, Wilson P, Davidson Brian R: **Antibiotic prophylaxis for the prevention of methicillin-resistant Staphylococcus aureus (MRSA) related complications in surgical patients**. *Cochrane Database of Systematic Reviews* 2013(8).

33. Gurusamy Kurinchi S, Naik P, Davidson Brian R: **Methods of decreasing infection to improve outcomes after liver resections**. *Cochrane Database of Systematic Reviews* 2011(11).

34. Gyte Gillian M, Dou L, Vazquez Juan C: **Different classes of antibiotics given to women routinely for preventing infection at caesarean section**. *Cochrane Database of Systematic Reviews* 2014(11).

35. Haas David M, Morgan S, Contreras K: **Vaginal preparation with antiseptic solution before cesarean section for preventing postoperative infections**. *Cochrane Database of Systematic Reviews* 2014(12).

36. Hadiati Diah R, Hakimi M, Nurdiati Detty S, Ota E: **Skin preparation for preventing infection following caesarean section**. *Cochrane Database of Systematic Reviews* 2014(9).

37. Heal Clare F, Banks Jennifer L, Lepper Phoebe D, Kontopantelis E, van Driel Mieke L: **Topical antibiotics for preventing surgical site infection in wounds healing by primary intention**. *Cochrane Database of Systematic Reviews* 2016(11).

38. Hsu Charlie CT, Kwan Gigi N, Evans-Barns H, Rophael John A, van Driel Mieke L: **Venous cutdown versus the Seldinger technique for placement of totally implantable venous access ports**. *Cochrane Database of Systematic Reviews* 2016(8).

39. Hua F, Xie H, Worthington Helen V, Furness S, Zhang Q, Li C: **Oral hygiene care for critically ill patients to prevent ventilator-associated pneumonia**. *Cochrane Database of Systematic Reviews* 2016(10).

40. Hughes C, Tunney M, Bradley Marie C: **Infection control strategies for preventing the transmission of meticillin-resistant Staphylococcus aureus (MRSA) in nursing homes for older people**. *Cochrane Database of Systematic Reviews* 2013(11).

41. Ireland Claire J, Chapman Timothy M, Mathew Suneeth F, Herbison GP, Zacharias M: **Continuous positive airway pressure (CPAP) during the postoperative period for prevention of postoperative morbidity and mortality following major abdominal surgery**. *Cochrane Database of Systematic Reviews* 2014(8).

42. Jamison J, Maguire S, McCann J: **Catheter policies for management of long term voiding problems in adults with neurogenic bladder disorders**. *Cochrane Database of Systematic Reviews* 2013(11).

43. Jefferson T, Del Mar Chris B, Dooley L, Ferroni E, Al-Ansary Lubna A, Bawazeer Ghada A, van Driel Mieke L, Nair S, Jones Mark A, Thorning S *et al*: **Physical interventions to interrupt or reduce the spread of respiratory viruses**. *Cochrane Database of Systematic Reviews* 2011(7).

44. Jones Daniel J, Bunn F, Bell-Syer Sophie V: **Prophylactic antibiotics to prevent surgical site infection after breast cancer surgery**. *Cochrane Database of Systematic Reviews* 2014(3).

45. Kao Lillian S, Meeks D, Moyer Virginia A, Lally Kevin P: **Peri-operative glycaemic control regimens for preventing surgical site infections in adults**. *Cochrane Database of Systematic Reviews* 2009(3).

46. Kelly M, Gillies D, Todd David A, Lockwood C: **Heated humidification versus heat and moisture exchangers for ventilated adults and children**. *Cochrane Database of Systematic Reviews* 2010(4).

47. Kidd Emily A, Stewart F, Kassis Nadine C, Hom E, Omar Muhammad I: **Urethral (indwelling or intermittent) or suprapubic routes for short-term catheterisation in hospitalised adults**. *Cochrane Database of Systematic Reviews* 2015(12).

48. Lai Nai M, Chaiyakunapruk N, Lai Nai A, O'Riordan E, Pau Wilson Shu C, Saint S: **Catheter impregnation, coating or bonding for reducing central venous catheter-related infections in adults**. *Cochrane Database of Systematic Reviews* 2016(3).

49. Lai Nai M, Lai Nai A, O'Riordan E, Chaiyakunapruk N, Taylor Jacqueline E, Tan K: **Skin antisepsis for reducing central venous catheter-related infections**. *Cochrane Database of Systematic Reviews* 2016(7).

50. Lam Thomas B, Omar Muhammad I, Fisher E, Gillies K, MacLennan S: **Types of indwelling urethral catheters for short-term catheterisation in hospitalised adults**. *Cochrane Database of Systematic Reviews* 2014(9).

51. Lethaby A, Temple J, Santy-Tomlinson J: **Pin site care for preventing infections associated with external bone fixators and pins**. *Cochrane Database of Systematic Reviews* 2013(12).

52. Liabsuetrakul T, Choobun T, Peeyananjarassri K, Islam QM: **Antibiotic prophylaxis for operative vaginal delivery**. *Cochrane Database of Systematic Reviews* 2014(10).

53. Lipp A, Lusardi G: **Systemic antimicrobial prophylaxis for percutaneous endoscopic gastrostomy**. *Cochrane Database of Systematic Reviews* 2013(11).

54. Lo David K, Hurley Matthew N, Muhlebach Marianne S, Smyth Alan R: **Interventions for the eradication of meticillin-resistant Staphylococcus aureus (MRSA) in people with cystic fibrosis**. *Cochrane Database of Systematic Reviews* 2015(2).

55. Lodi G, Figini L, Sardella A, Carrassi A, Del Fabbro M, Furness S: **Antibiotics to prevent complications following tooth extractions**. *Cochrane Database of Systematic Reviews* 2012(11).

56. Loeb Mark B, Main C, Eady A, Walkers-Dilks C: **Antimicrobial drugs for treating methicillin-resistant Staphylococcus aureus colonization**. *Cochrane Database of Systematic Reviews* 2003(4).

57. López-Alcalde J, Mateos-Mazón M, Guevara M, Conterno Lucieni O, Solà I, Cabir Nunes S, Bonfill Cosp X: **Gloves, gowns and masks for reducing the transmission of meticillin-resistant Staphylococcus aureus (MRSA) in the hospital setting**. *Cochrane Database of Systematic Reviews* 2015(7).

58. López-Briz E, Ruiz Garcia V, Cabello Juan B, Bort-Marti S, Carbonell Sanchis R, Burls A: **Heparin versus 0.9% sodium chloride intermittent flushing for prevention of occlusion in central venous catheters in adults**. *Cochrane Database of Systematic Reviews* 2014(10).

59. Low N, Mueller M, Van Vliet Huib A, Kapp N: **Perioperative antibiotics to prevent infection after first-trimester abortion**. *Cochrane Database of Systematic Reviews* 2012(3).

60. Lusardi G, Lipp A, Shaw C: **Antibiotic prophylaxis for short-term catheter bladder drainage in adults**. *Cochrane Database of Systematic Reviews* 2013(7).

61. Mackeen AD, Packard Roger E, Ota E, Berghella V, Baxter Jason K: **Timing of intravenous prophylactic antibiotics for preventing postpartum infectious morbidity in women undergoing cesarean delivery**. *Cochrane Database of Systematic Reviews* 2014(12).

62. Madrid E, Urrútia G, Roqué i Figuls M, Pardo-Hernandez H, Campos Juan M, Paniagua P, Maestre L, Alonso-Coello P: **Active body surface warming systems for preventing complications caused by inadvertent perioperative hypothermia in adults**. *Cochrane Database of Systematic Reviews* 2016(4).

63. Marsh N, Webster J, Mihala G, Rickard Claire M: **Devices and dressings to secure peripheral venous catheters to prevent complications**. *Cochrane Database of Systematic Reviews* 2015(6).

64. Masters B, Aarabi S, Sidhwa F, Wood F: **High-carbohydrate, high-protein, low-fat versus low-carbohydrate, high-protein, high-fat enteral feeds for burns**. *Cochrane Database of Systematic Reviews* 2012(1).

65. May W, Gülmezoglu AM, Ba-Thike K: **Antibiotics for incomplete abortion**. *Cochrane Database of Systematic Reviews* 2007(4).

66. McCann M, Moore Zena E: **Interventions for preventing infectious complications in haemodialysis patients with central venous catheters**. *Cochrane Database of Systematic Reviews* 2010(1).

67. Nabhan Ashraf F, Allam Nahed E, Hamed Abdel-Aziz Salama M: **Routes of administration of antibiotic prophylaxis for preventing infection after caesarean section**. *Cochrane Database of Systematic Reviews* 2016(6).

68. Nelson Richard L, Gladman E, Barbateskovic M: **Antimicrobial prophylaxis for colorectal surgery**. *Cochrane Database of Systematic Reviews* 2014(5).

69. Niël-Weise Barbara S, van den Broek Peterhans J, da Silva Edina M, Silva Laercio A: **Urinary catheter policies for long-term bladder drainage**. *Cochrane Database of Systematic Reviews* 2012(8).

70. Norman G, Dumville Jo C, Mohapatra Devi P, Owens Gemma L, Crosbie Emma J: **Antibiotics and antiseptics for surgical wounds healing by secondary intention**. *Cochrane Database of Systematic Reviews* 2016(3).

71. Norman G, Dumville Jo C, Moore Zena E, Tanner J, Christie J, Goto S: **Antibiotics and antiseptics for pressure ulcers**. *Cochrane Database of Systematic Reviews* 2016(4).

72. Phipps S, Lim Yik N, McClinton S, Barry C, Rane A, N'Dow James M: **Short term urinary catheter policies following urogenital surgery in adults**. *Cochrane Database of Systematic Reviews* 2006(2).

73. Prayle Andrew P, Hurley Matthew N, Smyth Alan R: **Percutaneous lines for delivering intravenous antibiotics in people with cystic fibrosis**. *Cochrane Database of Systematic Reviews* 2010(11).

74. Ratilal Bernardo O, Costa J, Sampaio C: **Antibiotic prophylaxis for surgical introduction of intracranial ventricular shunts**. *Cochrane Database of Systematic Reviews* 2006(3).

75. Ray A, Ray S: **Antibiotics prior to amniotomy for reducing infectious morbidity in mother and infant**. *Cochrane Database of Systematic Reviews* 2014(10).

76. Robertson-Malt S, Malt Greg N, Farquhar V, Greer W: **Heparin versus normal saline for patency of arterial lines**. *Cochrane Database of Systematic Reviews* 2014(5).

77. Sajid Muhammad S, Hutson Kristian H, Rapisarda Ignazio F, Bonomi R: **Fibrin glue instillation under skin flaps to prevent seroma-related morbidity following breast and axillary surgery**. *Cochrane Database of Systematic Reviews* 2013(5).

78. Sanabria A, Dominguez Luis C, Valdivieso E, Gomez G: **Antibiotic prophylaxis for patients undergoing elective laparoscopic cholecystectomy**. *Cochrane Database of Systematic Reviews* 2010(12).

79. Sanchez-Manuel Francisco J, Lozano-García J, Seco-Gil Juan L: **Antibiotic prophylaxis for hernia repair**. *Cochrane Database of Systematic Reviews* 2012(2).

80. Shah Prakeshkumar S, Shah N: **Heparin-bonded catheters for prolonging the patency of central venous catheters in children**. *Cochrane Database of Systematic Reviews* 2014(2).

81. Smaill Fiona M, Grivell Rosalie M: **Antibiotic prophylaxis versus no prophylaxis for preventing infection after cesarean section**. *Cochrane Database of Systematic Reviews* 2014(10).

82. Stewart A, Eyers Paul S, Earnshaw Jonothan J: **Prevention of infection in arterial reconstruction**. *Cochrane Database of Systematic Reviews* 2006(3).

83. Storm-Versloot Marja N, Vos Cornelis G, Ubbink Dirk T, Vermeulen H: **Topical silver for preventing wound infection**. *Cochrane Database of Systematic Reviews* 2010(3).

84. Strippoli Giovanni F, Tong A, Johnson David W, Schena Francesco P, Craig Jonathan C: **Antimicrobial agents for preventing peritonitis in peritoneal dialysis patients**. *Cochrane Database of Systematic Reviews* 2004(4).

85. Strippoli Giovanni F, Tong A, Johnson David W, Schena Francesco P, Craig Jonathan C: **Catheter type, placement and insertion techniques for preventing peritonitis in peritoneal dialysis patients**. *Cochrane Database of Systematic Reviews* 2004(4).

86. Subirana M, Solà I, Benito S: **Closed tracheal suction systems versus open tracheal suction systems for mechanically ventilated adult patients**. *Cochrane Database of Systematic Reviews* 2007(4).

87. Syed Mohammed I, Suller S, Browning George G, Akeroyd Michael A: **Interventions for the prevention of postoperative ear discharge after insertion of ventilation tubes (grommets) in children**. *Cochrane Database of Systematic Reviews* 2013(4).

88. Tanner J, Dumville Jo C, Norman G, Fortnam M: **Surgical hand antisepsis to reduce surgical site infection**. *Cochrane Database of Systematic Reviews* 2016(1).

89. Tanner J, Norrie P, Melen K: **Preoperative hair removal to reduce surgical site infection**. *Cochrane Database of Systematic Reviews* 2011(11).

90. Tanner J, Parkinson H: **Double gloving to reduce surgical cross-infection**. *Cochrane Database of Systematic Reviews* 2006(3).

91. Thomas Roger E, Jefferson T, Lasserson Toby J: **Influenza vaccination for healthcare workers who care for people aged 60 or older living in long-term care institutions**. *Cochrane Database of Systematic Reviews* 2016(6).

92. Tokmaji G, Vermeulen H, Müller Marcella C, Kwakman Paulus H, Schultz Marcus J, Zaat Sebastian A: **Silver-coated endotracheal tubes for prevention of ventilator-associated pneumonia in critically ill patients**. *Cochrane Database of Systematic Reviews* 2015(8).

93. Toon Clare D, Lusuku C, Ramamoorthy R, Davidson Brian R, Gurusamy Kurinchi S: **Early versus delayed dressing removal after primary closure of clean and clean-contaminated surgical wounds**. *Cochrane Database of Systematic Reviews* 2015(9).

94. Toon Clare D, Sinha S, Davidson Brian R, Gurusamy Kurinchi S: **Early versus delayed post-operative bathing or showering to prevent wound complications**. *Cochrane Database of Systematic Reviews* 2015(7).

95. Torres Maria F, Porfirio Gustavo J, Carvalho Alan P, Riera R: **Non-invasive positive pressure ventilation for prevention of complications after pulmonary resection in lung cancer patients**. *Cochrane Database of Systematic Reviews* 2015(9).

96. Ullman Amanda J, Cooke Marie L, Gillies D, Marsh Nicole M, Daud A, McGrail Matthew R, O'Riordan E, Rickard Claire M: **Optimal timing for intravascular administration set replacement**. *Cochrane Database of Systematic Reviews* 2013(9).

97. Ullman Amanda J, Cooke Marie L, Mitchell M, Lin F, New K, Long Debbie A, Mihala G, Rickard Claire M: **Dressings and securement devices for central venous catheters (CVC)**. *Cochrane Database of Systematic Reviews* 2015(9).

98. van de Wetering Marianne D, van Woensel Job B, Lawrie Theresa A: **Prophylactic antibiotics for preventing Gram positive infections associated with long-term central venous catheters in oncology patients**. *Cochrane Database of Systematic Reviews* 2013(11).

99. van Rijen M, Bonten M, Wenzel R, Kluytmans J: **Mupirocin ointment for preventing Staphylococcus aureus infections in nasal carriers**. *Cochrane Database of Systematic Reviews* 2008(4).

100. Vermeulen H, Ubbink Dirk T, Goossens A, de Vos R, Legemate Dink A, Westerbos Stijn J: **Dressings and topical agents for surgical wounds healing by secondary intention**. *Cochrane Database of Systematic Reviews* 2004(1).

101. Verschuur Hendrik P, de Wever W, van Benthem Peter P: **Antibiotic prophylaxis in clean and clean-contaminated ear surgery**. *Cochrane Database of Systematic Reviews* 2004(3).

102. Villatoro E, Mulla M, Larvin M: **Antibiotic therapy for prophylaxis against infection of pancreatic necrosis in acute pancreatitis**. *Cochrane Database of Systematic Reviews* 2010(5).

103. Vincent M, Edwards P: **Disposable surgical face masks for preventing surgical wound infection in clean surgery**. *Cochrane Database of Systematic Reviews* 2016(4).

104. Wang L, Li X, Yang Z, Tang X, Yuan Q, Deng L, Sun X: **Semi-recumbent position versus supine position for the prevention of ventilator-associated pneumonia in adults requiring mechanical ventilation**. *Cochrane Database of Systematic Reviews* 2016(1).

105. Wang Y, Ivany Jessica N, Perkovic V, Gallagher Martin P, Woodward M, Jardine Meg J: **Anticoagulants and antiplatelet agents for preventing central venous haemodialysis catheter malfunction in patients with end-stage kidney disease**. *Cochrane Database of Systematic Reviews* 2016(4).

106. Webster J, Alghamdi A: **Use of plastic adhesive drapes during surgery for preventing surgical site infection**. *Cochrane Database of Systematic Reviews* 2015(4).

107. Webster J, Bell-Syer Sally E, Foxlee R: **Skin preparation with alcohol versus alcohol followed by any antiseptic for preventing bacteraemia or contamination of blood for transfusion**. *Cochrane Database of Systematic Reviews* 2015(2).

108. Webster J, Osborne S: **Preoperative bathing or showering with skin antiseptics to prevent surgical site infection**. *Cochrane Database of Systematic Reviews* 2015(2).

109. Webster J, Scuffham P, Stankiewicz M, Chaboyer Wendy P: **Negative pressure wound therapy for skin grafts and surgical wounds healing by primary intention**. *Cochrane Database of Systematic Reviews* 2014(10).

110. Westendorp Willeke F, Vermeij J-D, Vermeij F, Den Hertog Heleen M, Dippel Diederik W, van de Beek D, Nederkoorn Paul J: **Antibiotic therapy for preventing infections in patients with acute stroke**. *Cochrane Database of Systematic Reviews* 2012(1).

111. Wetterslev J, Meyhoff Christian S, Jørgensen Lars N, Gluud C, Lindschou J, Rasmussen Lars S: **The effects of high perioperative inspiratory oxygen fraction for adult surgical patients**. *Cochrane Database of Systematic Reviews* 2015(6).

112. Wojcieszek Aleena M, Stock Owen M, Flenady V: **Antibiotics for prelabour rupture of membranes at or near term**. *Cochrane Database of Systematic Reviews* 2014(10).

113. Wood C, Phillips C: **Cyanoacrylate microbial sealants for skin preparation prior to surgery**. *Cochrane Database of Systematic Reviews* 2016(5).
