# Supplementary material for: Consideration of sex and gender in Cochrane reviews of interventions for preventing healthcare-associated infections: a methodology study
Source: BMC Health Serv Res. 2019 Mar 15;19:169. doi: 10.1186/s12913-019-4001-9 (PMC6419810; doi:10.1186/s12913-019-4001-9)
Supplement: Supplementary file 6 — Responses to the Sex and Gender Appraisal Tool for Systematic Reviews (SGAT-SR). (DOCX 96 kb) [file 12913_2019_4001_MOESM6_ESM.docx]

**Additional file 6: Responses to the Sex and Gender Appraisal Tool for Systematic Reviews (SGAT-SR)**

| **Item** | **Alkhawaja (2015)** |
| --- | --- |
| Background | **1.1 Are the terms sex and gender used in the background?** • No, review did not meet criteria **1.2 Are sex/gender identified as relevant or not to review question?** • No, review did not meet criteria **1.3. Does background discuss why sex/gender differences may be expected?** • No, review did not meet criteria |
| Objectives | **2.1. Are the terms sex, gender, male, or female used in objectives?** • No, review did not meet criteria |
| Criteria for inclusion/ exclusion | **3.1. Do the review’s inclusion-exclusion criteria consider sex-gender differences?** • No, review did not meet criteria **3.2. Was there justification or explanation for the exclusion of some groups?** • No, review did not meet criteria |
| Methods | **4.1. Does the review examine whether outcome measures are different for males and females?** • No, review did not meet criteria **4.2. Did the review extract data by sex?** • No, review did not meet criteria **4.3. Did the review extract data on sex of withdrawals and dropouts?** • No, review did not meet criteria **4.4. In cases where sex/gender is used as a proxy for other measures (i.e., weight), is there an explanation for this approach?** • Item was not applicable to the review **4.5. Were any subgroup analyses completed?** • Yes, review met criteria *Quote: "Subgroup analysis: Participants were mechanically ventilated in six trials (Acosta- Escribano 2010; Davies 2002; Davies 2012; Hsu 2009; Kortbeek 1999; White 2009). Authors for the remaining three trials (Day 2001; Montecalvo 1992;Montejo 2002) did not mention whether participants were mechanically ventilated; this prevented us from performing a subgroup analysis for participants who were not on mechanical ventilation. Only one study provided no clear definition for pneumonia (Day 2001). Results of this subgroup analysis were not modified much by removal of this study (RR 0.66, 95%CI 0.52 to 0.85; I² = 0%)."* **4.6. Were subgroup analyses by sex completed?** • No, review did not meet criteria |
| Results and Analysis | **5.1. Do results distinguish between findings for males/females?** • No, review did not meet criteria **5.2. Does the review report conclusions (of effectiveness, efficacy, safety) that are different for men and women?**  • No, review did not meet criteria **5.3. If adverse effects are reported, is information sex disaggregated?** • No, review did not meet criteria *Quote: "Complications related to tube insertion (epistaxis, pneumothoraces, gastrointestinal bleeding)". Quote: "Complications related to tube maintenance (need for tube replacement, tube occlusion)".*  **5.4. Does review note that subgroup analyses by sex could not be done?** • No, review did not meet criteria *Comment: Subgroup analysis by sex was not planned.* |
| Discussion and conclusion | **6.1. Does the review report that primary studies analysed or failed to analyse results by sex?** • No, review did not meet criteria **6.2. Does the review address sex/gender implications for clinical practice?** • No, review did not meet criteria **6.3. Does the review address sex/gender implications for policy and regulation?** • No, review did not meet criteria **6.4. Does the review address sex/gender implications for research?** • No, review did not meet criteria |
| Table of included studies | **7.1. Does the description of included studies give detailed information on sex/gender of the study samples?** • No, review did not meet criteria (At least 7.1.1. or 7.1.2. are NO) **7.1.1. Detailed information on SEX of the study samples** • No, review did not meet criteria **7.1.2. Detailed information on GENDER of the study samples** • No, review did not meet criteria *Quote: "Male: 86%”.* |
|  |  |
| **Item** | **Andersen (2005)** |
| Background | **1.1 Are the terms sex and gender used in the background?** • Yes, review met criteria *Quote: “The actual incidence of acute appendicitis varies, the overall lifetime risk for acute appendicitis is 6-20% (Blewett 1995; Addiss 1990). Addiss reported 8.6% for males and 6.7% for females, compared to an overall life time risk for appendectomy of 12% (males) and 23.1% (females) in US.”* **1.2 Are sex/gender identified as relevant or not to review question?** • Unable to determine **1.3. Does background discuss why sex/gender differences may be expected?** • No, review did not meet criteria *Quote: "The actual incidence of acute appendicitis varies, the overall lifetime risk for acute appendicitis is 6-20% (Blewett 1995; Addiss 1990). Addiss reported 8.6% for males and 6.7% for females, compared to an overall life time risk for appendectomy of 12% (males) and 23.1% (females) in US.” Comment: The review describes the sex differences in the incidence of acute appendicitis and appendectomy, but it does not explain why this occurs.* |
| Objectives | **2.1. Are the terms sex, gender, male, or female used in objectives?** • No, review did not meet criteria *Quote: "A systematic search for relevant literature from controlled clinical trials was performed to find evidence relating to the use of antibiotics in patients undergoing appendectomy on the suspicion of appendicitis. Relevant data were extracted from these reports of clinical trials and analysis performed which reflects the clinical decision-making process. Given the fact, that patients are operated upon suspicion of appendicitis, we wished to explore whether postoperative infection and prolonged stay in hospital could be prevented if the patients were given antimicrobial therapy prior to, under, or after the surgery." (on objectives)* |
| Criteria for inclusion/ exclusion | **3.1. Do the review’s inclusion-exclusion criteria consider sex-gender differences?** • No, review did not meet criteria *Quote: "No restrictions to age or gender" (in Types of participants). Comment: The review did not justify this* **3.2. Was there justification or explanation for the exclusion of some groups?** • No, review did not meet criteria *Comment: sex-gender were not considered as inclusion-exclusion criteria.* |
| Methods | **4.1. Does the review examine whether outcome measures are different for males and females?** • No, review did not meet criteria *Quote: "No attempt was made to elucidate age- or gender related correlations." (in Description of studies)* **4.2. Did the review extract data by sex?** • No, review did not meet criteria *Quote: "No attempt was made to elucidate age- or gender related correlations."* **4.3. Did the review extract data on sex of withdrawals and dropouts?** • No, review did not meet criteria *Quote: "Wrote to author to specify subgrouping on drop-outs." (in Table included studies). Comment: The reviewers asked for drop-outs, but they appear not to look for sex-gender disaggregated information.* **4.4. In cases where sex/gender is used as a proxy for other measures (i.e., weight), is there an explanation for this approach?** • Item was not applicable to the review **4.5. Were any subgroup analyses completed?** • Yes, review met criteria *Quote: "The analyses are stratified to include subgroups of patients with simple appendicitis and complicated appendicitis." (in Background) Quote: "In this review we analysed patients with a normal appendix and patients with perforated appendix." (in outcomes) Quote: Subgroups: "patients with a normal appendix" and "patients with perforated appendix."*  **4.6. Were subgroup analyses by sex completed?** • No, review did not meet criteria *Quote: "No attempt was made to elucidate age- or gender related correlations." (in Description of studies)* |
| Results and Analysis | **5.1. Do results distinguish between findings for males/females?** • No, review did not meet criteria **5.2. Does the review report conclusions (of effectiveness, efficacy, safety) that are different for men and women?**  • No, review did not meet criteria *Quote: "No attempt was made to elucidate age- or gender related correlations." (in Description of studies)* **5.3. If adverse effects are reported, is information sex disaggregated?** • Item was not applicable to the review *Comment: No adverse effects were reported.* **5.4. Does review note that subgroup analyses by sex could not be done?** • No, review did not meet criteria *Comment: Subgroup analysis by sex was not planned.* |
| Discussion and conclusion | **6.1. Does the review report that primary studies analysed or failed to analyse results by sex?** • No, review did not meet criteria **6.2. Does the review address sex/gender implications for clinical practice?** • No, review did not meet criteria *Quote: "Implications for practice: It seems reasonable to conclude that antibiotic prophylaxis is effective in the prevention of post-operative complications in appendectomied patients, whether the administration is given pre- , per- or post-operatively, and should be considered for routine use in emergency appendicectomy. The overall strategy (type of antibiotics and/or preferred time of administration) needs to be evaluated in another systematic review. Our results indicate that single doses have the same impact as multiple doses. In order to reduce cost, toxicity and the risk of developing bacterial resistance, it is desirable to establish the shortest, effective prophylaxis for post-operative complications, and from the meta-analysis it seem that single doses have the same impact as multiple doses. The general assumption is that it is better to treat patients with complicated appendicitis with antibiotics than with placebo, in contrast to patients with simple appendicitis. Yet other studies have questioned the use of antibiotics at all in appendectomised patients."* **6.3. Does the review address sex/gender implications for policy and regulation?** • No, review did not meet criteria **6.4. Does the review address sex/gender implications for research?** • No, review did not meet criteria |
| Table of included studies | **7.1. Does the description of included studies give detailed information on sex/gender of the study samples?** • No, review did not meet criteria (At least 7.1.1. or 7.1.2. are NO) **7.1.1. Detailed information on SEX of the study samples** • No, review did not meet criteria **7.1.2. Detailed information on GENDER of the study samples** • No, review did not meet criteria |
|  |  |
| **Item** | **Arnold (2005)** |
| Background | **1.1 Are the terms sex and gender used in the background?** • No, review did not meet criteria **1.2 Are sex/gender identified as relevant or not to review question?** • No, review did not meet criteria **1.3. Does background discuss why sex/gender differences may be expected?** • No, review did not meet criteria |
| Objectives | **2.1. Are the terms sex, gender, male, or female used in objectives?** • No, review did not meet criteria *Quote: “The main objective of this study was to systematically review the literature to find trials to enable an estimate of the effectiveness of interventions targeting professionals, when given alone or in combination, in improving antibiotic prescribing by healthcare providers; in the outpatient setting with both adults and children. Prescribing behaviours included: (a) the decision to prescribe an antibiotic for a specific condition; (b) the type of antibiotic prescribed; (c) the dose and duration of antibiotic therapy. The secondary objective of this systematic review was to estimate the effect of any of the interventions on patient outcomes that were related to antibiotic use, including: (a) colonization or infection with antibiotic-resistant organisms (b) adverse drug reactions (allergic and non-allergic) and antibiotic associated diarrhoea (c) adverse events related to non-use of antibiotics, use of narrower spectrum antibiotics or a reduced duration of antibiotic therapy.”* |
| Criteria for inclusion/ exclusion | **3.1. Do the review’s inclusion-exclusion criteria consider sex-gender differences?** • No, review did not meet criteria **3.2. Was there justification or explanation for the exclusion of some groups?** • No, review did not meet criteria |
| Methods | **4.1. Does the review examine whether outcome measures are different for males and females?** • No, review did not meet criteria **4.2. Did the review extract data by sex?** • No, review did not meet criteria **4.3. Did the review extract data on sex of withdrawals and dropouts?** • No, review did not meet criteria **4.4. In cases where sex/gender is used as a proxy for other measures (i.e., weight), is there an explanation for this approach?** • Item was not applicable to the review **4.5. Were any subgroup analyses completed?** • No, review did not meet criteria **4.6. Were subgroup analyses by sex completed?** • No, review did not meet criteria |
| Results and Analysis | **5.1. Do results distinguish between findings for males/females?** • No, review did not meet criteria **5.2. Does the review report conclusions (of effectiveness, efficacy, safety) that are different for men and women?**  • No, review did not meet criteria **5.3. If adverse effects are reported, is information sex disaggregated?** • Item was not applicable to the review **5.4. Does review note that subgroup analyses by sex could not be done?** • Item was not applicable to the review |
| Discussion and conclusion | **6.1. Does the review report that primary studies analysed or failed to analyse results by sex?** • No, review did not meet criteria **6.2. Does the review address sex/gender implications for clinical practice?** • No, review did not meet criteria **6.3. Does the review address sex/gender implications for policy and regulation?** • No, review did not meet criteria **6.4. Does the review address sex/gender implications for research?** • No, review did not meet criteria |
| Table of included studies | **7.1. Does the description of included studies give detailed information on sex/gender of the study samples?** • No, review did not meet criteria (At least 7.1.1. or 7.1.2. are NO) **7.1.1. Detailed information on SEX of the study samples** • No, review did not meet criteria **7.1.2. Detailed information on GENDER of the study samples** • No, review did not meet criteria |
|  |  |
| **Item** | **Arora (2010)** |
| Background | **1.1 Are the terms sex and gender used in the background?** • No, review did not meet criteria **1.2 Are sex/gender identified as relevant or not to review question?** • No, review did not meet criteria **1.3. Does background discuss why sex/gender differences may be expected?** • No, review did not meet criteria |
| Objectives | **2.1. Are the terms sex, gender, male, or female used in objectives?** • No, review did not meet criteria *Quote: "OBJECTIVES: The primary aim was to find which interventions (other than anticoagulants, systemic antibiotics and antibiotic lock techniques) were effective in preventing CVC-related infections in children with cancer. Further objectives were to examine the effectiveness of each intervention (for details see the section ’Types of interventions’) across the following subgroups: 1. those with implanted versus external long-term tunnelled catheters; 2. those with haematological versus non-haematological malignancies; and 3. those children with cancer who have undergone haematopoietic stem cell transplant (HSCT) versus those who have not."* |
| Criteria for inclusion/ exclusion | **3.1. Do the review’s inclusion-exclusion criteria consider sex-gender differences?** • No, review did not meet criteria *Quote: "All children (less than 18 years of age) [...]"* **3.2. Was there justification or explanation for the exclusion of some groups?** • No, review did not meet criteria |
| Methods | **4.1. Does the review examine whether outcome measures are different for males and females?** • No, review did not meet criteria **4.2. Did the review extract data by sex?** • No, review did not meet criteria **4.3. Did the review extract data on sex of withdrawals and dropouts?** • No, review did not meet criteria **4.4. In cases where sex/gender is used as a proxy for other measures (i.e., weight), is there an explanation for this approach?** • Item was not applicable to the review **4.5. Were any subgroup analyses completed?** • No, review did not meet criteria **4.6. Were subgroup analyses by sex completed?** • No, review did not meet criteria |
| Results and Analysis | **5.1. Do results distinguish between findings for males/females?** • No, review did not meet criteria **5.2. Does the review report conclusions (of effectiveness, efficacy, safety) that are different for men and women?**  • No, review did not meet criteria **5.3. If adverse effects are reported, is information sex disaggregated?** • Item was not applicable to the review **5.4. Does review note that subgroup analyses by sex could not be done?** • Item was not applicable to the review |
| Discussion and conclusion | **6.1. Does the review report that primary studies analysed or failed to analyse results by sex?** • No, review did not meet criteria **6.2. Does the review address sex/gender implications for clinical practice?** • No, review did not meet criteria **6.3. Does the review address sex/gender implications for policy and regulation?** • No, review did not meet criteria **6.4. Does the review address sex/gender implications for research?** • No, review did not meet criteria |
| Table of included studies | **7.1. Does the description of included studies give detailed information on sex/gender of the study samples?** • No, review did not meet criteria (At least 7.1.1. or 7.1.2. are NO) **7.1.1. Detailed information on SEX of the study samples** • No, review did not meet criteria **7.1.2. Detailed information on GENDER of the study samples** • No, review did not meet criteria |
|  |  |
| **Item** | **Arrowsmith (2014)** |
| Background | **1.1 Are the terms sex and gender used in the background?** • No, review did not meet criteria **1.2 Are sex/gender identified as relevant or not to review question?** • No, review did not meet criteria **1.3. Does background discuss why sex/gender differences may be expected?** • No, review did not meet criteria |
| Objectives | **2.1. Are the terms sex, gender, male, or female used in objectives?** • No, review did not meet criteria *Quote: "OBJECTIVES: To assess the effect of the presence or absence of rings and nail polish on the hands of the surgical team on postoperative wound infection rates.”* |
| Criteria for inclusion/ exclusion | **3.1. Do the review’s inclusion-exclusion criteria consider sex-gender differences?** • No, review did not meet criteria *Quote: "Types of participants: Those members of the theatre team who scrub for invasive operative procedures: surgeons, anaesthetists, surgeon’s assistants, scrub nurses, operating department practitioners, and scrubbed observers, in any country.”* **3.2. Was there justification or explanation for the exclusion of some groups?** • No, review did not meet criteria *Quote: "Types of participants: Those members of the theatre team who scrub for invasive operative procedures: surgeons, anaesthetists, surgeon’s assistants, scrub nurses, operating department practitioners, and scrubbed observers, in any country.”* |
| Methods | **4.1. Does the review examine whether outcome measures are different for males and females?** • No, review did not meet criteria **4.2. Did the review extract data by sex?** • No, review did not meet criteria **4.3. Did the review extract data on sex of withdrawals and dropouts?** • No, review did not meet criteria **4.4. In cases where sex/gender is used as a proxy for other measures (i.e., weight), is there an explanation for this approach?** • Item was not applicable to the review **4.5. Were any subgroup analyses completed?** • No, review did not meet criteria **4.6. Were subgroup analyses by sex completed?** • No, review did not meet criteria |
| Results and Analysis | **5.1. Do results distinguish between findings for males/females?** • No, review did not meet criteria **5.2. Does the review report conclusions (of effectiveness, efficacy, safety) that are different for men and women?**  • No, review did not meet criteria **5.3. If adverse effects are reported, is information sex disaggregated?** • Item was not applicable to the review **5.4. Does review note that subgroup analyses by sex could not be done?** • Item was not applicable to the review |
| Discussion and conclusion | **6.1. Does the review report that primary studies analysed or failed to analyse results by sex?** • No, review did not meet criteria **6.2. Does the review address sex/gender implications for clinical practice?** • No, review did not meet criteria **6.3. Does the review address sex/gender implications for policy and regulation?** • No, review did not meet criteria **6.4. Does the review address sex/gender implications for research?** • No, review did not meet criteria |
| Table of included studies | **7.1. Does the description of included studies give detailed information on sex/gender of the study samples?** • No, review did not meet criteria (At least 7.1.1. or 7.1.2. are NO) **7.1.1. Detailed information on SEX of the study samples** • No, review did not meet criteria **7.1.2. Detailed information on GENDER of the study samples** • No, review did not meet criteria |
|  |  |
| **Item** | **Barajas-Nava (2013)** |
| Background | **1.1 Are the terms sex and gender used in the background?** • No, review did not meet criteria **1.2 Are sex/gender identified as relevant or not to review question?** • No, review did not meet criteria **1.3. Does background discuss why sex/gender differences may be expected?** • No, review did not meet criteria |
| Objectives | **2.1. Are the terms sex, gender, male, or female used in objectives?** • No, review did not meet criteria *Quote: "To assess the effects of antibiotic prophylaxis on rates of burn wound infection.”* |
| Criteria for inclusion/ exclusion | **3.1. Do the review’s inclusion-exclusion criteria consider sex-gender differences?** • No, review did not meet criteria *Quote: "People of any age or gender [...]"* **3.2. Was there justification or explanation for the exclusion of some groups?** • No, review did not meet criteria *Quote: "People of any age or gender [...]"* |
| Methods | **4.1. Does the review examine whether outcome measures are different for males and females?** • No, review did not meet criteria **4.2. Did the review extract data by sex?** • No, review did not meet criteria **4.3. Did the review extract data on sex of withdrawals and dropouts?** • No, review did not meet criteria **4.4. In cases where sex/gender is used as a proxy for other measures (i.e., weight), is there an explanation for this approach?** • Item was not applicable to the review **4.5. Were any subgroup analyses completed?** • No, review did not meet criteria **4.6. Were subgroup analyses by sex completed?** • No, review did not meet criteria |
| Results and Analysis | **5.1. Do results distinguish between findings for males/females?** • No, review did not meet criteria **5.2. Does the review report conclusions (of effectiveness, efficacy, safety) that are different for men and women?**  • No, review did not meet criteria **5.3. If adverse effects are reported, is information sex disaggregated?** • No, review did not meet criteria **5.4. Does review note that subgroup analyses by sex could not be done?** • Item was not applicable to the review |
| Discussion and conclusion | **6.1. Does the review report that primary studies analysed or failed to analyse results by sex?** • No, review did not meet criteria **6.2. Does the review address sex/gender implications for clinical practice?** • No, review did not meet criteria **6.3. Does the review address sex/gender implications for policy and regulation?** • No, review did not meet criteria **6.4. Does the review address sex/gender implications for research?** • No, review did not meet criteria |
| Table of included studies | **7.1. Does the description of included studies give detailed information on sex/gender of the study samples?** • No, review did not meet criteria (At least 7.1.1. or 7.1.2. are NO) **7.1.1. Detailed information on SEX of the study samples** • No, review did not meet criteria *Comment: no information on sex* **7.1.2. Detailed information on GENDER of the study samples** • Unable to determine |
|  |  |
| **Item** | **Bo (2014)** |
| Background | **1.1 Are the terms sex and gender used in the background?** • No, review did not meet criteria **1.2 Are sex/gender identified as relevant or not to review question?** • No, review did not meet criteria **1.3. Does background discuss why sex/gender differences may be expected?** • No, review did not meet criteria |
| Objectives | **2.1. Are the terms sex, gender, male, or female used in objectives?** • No, review did not meet criteria |
| Criteria for inclusion/ exclusion | **3.1. Do the review’s inclusion-exclusion criteria consider sex-gender differences?** • No, review did not meet criteria *Quote: "Types of participants: Adult ICU patients (≥ 18 years of age) receiving mechanical ventilation with a reported incidence of VAP."* **3.2. Was there justification or explanation for the exclusion of some groups?** • No, review did not meet criteria |
| Methods | **4.1. Does the review examine whether outcome measures are different for males and females?** • No, review did not meet criteria **4.2. Did the review extract data by sex?** • No, review did not meet criteria *Quote: "We extracted the following information from each study: author, year of publication, language, their institutions, source of funding, participants (age range, gender, socioeconomic status, inclusion and exclusion criteria), methodological design [...]" Comments: This is a description of the study samples, not data extraction by sex.* **4.3. Did the review extract data on sex of withdrawals and dropouts?** • No, review did not meet criteria **4.4. In cases where sex/gender is used as a proxy for other measures (i.e., weight), is there an explanation for this approach?** • Item was not applicable to the review **4.5. Were any subgroup analyses completed?** • Yes, review met criteria *Quote: "Subgroup analysis and investigation of heterogeneity We performed subgroup analysis based on different placebo control groups and different probiotic doses or duration of therapy. We interpreted the results of subgroup analyses cautiously."* **4.6. Were subgroup analyses by sex completed?** • No, review did not meet criteria |
| Results and Analysis | **5.1. Do results distinguish between findings for males/females?** • No, review did not meet criteria **5.2. Does the review report conclusions (of effectiveness, efficacy, safety) that are different for men and women?**  • No, review did not meet criteria **5.3. If adverse effects are reported, is information sex disaggregated?** • No, review did not meet criteria **5.4. Does review note that subgroup analyses by sex could not be done?** • Item was not applicable to the review |
| Discussion and conclusion | **6.1. Does the review report that primary studies analysed or failed to analyse results by sex?** • No, review did not meet criteria **6.2. Does the review address sex/gender implications for clinical practice?** • No, review did not meet criteria **6.3. Does the review address sex/gender implications for policy and regulation?** • No, review did not meet criteria **6.4. Does the review address sex/gender implications for research?** • No, review did not meet criteria |
| Table of included studies | **7.1. Does the description of included studies give detailed information on sex/gender of the study samples?** • No, review did not meet criteria (At least 7.1.1. or 7.1.2. are NO) **7.1.1. Detailed information on SEX of the study samples** • Unable to determine **7.1.2. Detailed information on GENDER of the study samples** • No, review did not meet criteria *Quote: "Sex (MF)". Comment: no information on gender.* |
|  |  |
| **Item** | **Bradford (2015)** |
| Background | **1.1 Are the terms sex and gender used in the background?** • No, review did not meet criteria **1.2 Are sex/gender identified as relevant or not to review question?** • No, review did not meet criteria **1.3. Does background discuss why sex/gender differences may be expected?** • No, review did not meet criteria |
| Objectives | **2.1. Are the terms sex, gender, male, or female used in objectives?** • No, review did not meet criteria *Quote: "OBJECTIVES: To assess the clinical effects (benefits and harms) of intermittent flushing of heparin versus normal saline to prevent occlusion in long term central venous catheters in infants and children".* |
| Criteria for inclusion/ exclusion | **3.1. Do the review’s inclusion-exclusion criteria consider sex-gender differences?** • No, review did not meet criteria *Quote: "The study population of interest comprised infants and children aged 0 to 18 years of age [...]"* **3.2. Was there justification or explanation for the exclusion of some groups?** • No, review did not meet criteria |
| Methods | **4.1. Does the review examine whether outcome measures are different for males and females?** • No, review did not meet criteria **4.2. Did the review extract data by sex?** • No, review did not meet criteria **4.3. Did the review extract data on sex of withdrawals and dropouts?** • No, review did not meet criteria **4.4. In cases where sex/gender is used as a proxy for other measures (i.e., weight), is there an explanation for this approach?** • Item was not applicable to the review **4.5. Were any subgroup analyses completed?** • No, review did not meet criteria **4.6. Were subgroup analyses by sex completed?** • No, review did not meet criteria |
| Results and Analysis | **5.1. Do results distinguish between findings for males/females?** • No, review did not meet criteria **5.2. Does the review report conclusions (of effectiveness, efficacy, safety) that are different for men and women?**  • No, review did not meet criteria **5.3. If adverse effects are reported, is information sex disaggregated?** • No, review did not meet criteria **5.4. Does review note that subgroup analyses by sex could not be done?** • Item was not applicable to the review |
| Discussion and conclusion | **6.1. Does the review report that primary studies analysed or failed to analyse results by sex?** • No, review did not meet criteria **6.2. Does the review address sex/gender implications for clinical practice?** • No, review did not meet criteria **6.3. Does the review address sex/gender implications for policy and regulation?** • No, review did not meet criteria **6.4. Does the review address sex/gender implications for research?** • No, review did not meet criteria |
| Table of included studies | **7.1. Does the description of included studies give detailed information on sex/gender of the study samples?** • No, review did not meet criteria (At least 7.1.1. or 7.1.2. are NO) **7.1.1. Detailed information on SEX of the study samples** • No, review did not meet criteria **7.1.2. Detailed information on GENDER of the study samples** • Unable to determine |
|  |  |
| **Item** | **Brand (2010)** |
| Background | **1.1 Are the terms sex and gender used in the background?** • No, review did not meet criteria **1.2 Are sex/gender identified as relevant or not to review question?** • No, review did not meet criteria **1.3. Does background discuss why sex/gender differences may be expected?** • No, review did not meet criteria |
| Objectives | **2.1. Are the terms sex, gender, male, or female used in objectives?** • No, review did not meet criteria *Quote: "OBJECTIVES: To assess the benefits and harms of antibiotic prophylaxis before elective ERCP."* |
| Criteria for inclusion/ exclusion | **3.1. Do the review’s inclusion-exclusion criteria consider sex-gender differences?** • No, review did not meet criteria *Quote: "Types of participants: Patients that underwent elective ERCP that were not on antibiotics, without evidence of acute or chronic cholecystitis, cholangitis, or severe acute pancreatitis before the procedure."* **3.2. Was there justification or explanation for the exclusion of some groups?** • No, review did not meet criteria |
| Methods | **4.1. Does the review examine whether outcome measures are different for males and females?** • No, review did not meet criteria **4.2. Did the review extract data by sex?** • No, review did not meet criteria **4.3. Did the review extract data on sex of withdrawals and dropouts?** • No, review did not meet criteria **4.4. In cases where sex/gender is used as a proxy for other measures (i.e., weight), is there an explanation for this approach?** • Item was not applicable to the review **4.5. Were any subgroup analyses completed?** • Yes, review met criteria *Quote: "Subgroups that were considered included diagnostic compared to therapeutic ERCP, biliary compared to pancreatic indications for ERCP, different antibiotics, and trials with low bias risk compared to trials with high bias risk (one or more than one of the components inadequate or unclear)." Quote: "When we considered the subgroup of patients that underwent a successful first ERCP relieving the biliary obstruction, we were only able to include information from three studies. There were 13 patients with cholangitis out of 309 patients; 6 out of 147 in the prophylaxis group and 7 out of 162 patients in the control group. This gave a RR of 0.98, 95% CI 0.35 to 2.69, P = 0.96, I2 0% (Analysis 2.1)."* **4.6. Were subgroup analyses by sex completed?** • No, review did not meet criteria |
| Results and Analysis | **5.1. Do results distinguish between findings for males/females?** • No, review did not meet criteria **5.2. Does the review report conclusions (of effectiveness, efficacy, safety) that are different for men and women?**  • No, review did not meet criteria **5.3. If adverse effects are reported, is information sex disaggregated?** • No, review did not meet criteria **5.4. Does review note that subgroup analyses by sex could not be done?** • Item was not applicable to the review |
| Discussion and conclusion | **6.1. Does the review report that primary studies analysed or failed to analyse results by sex?** • No, review did not meet criteria **6.2. Does the review address sex/gender implications for clinical practice?** • No, review did not meet criteria **6.3. Does the review address sex/gender implications for policy and regulation?** • No, review did not meet criteria **6.4. Does the review address sex/gender implications for research?** • No, review did not meet criteria |
| Table of included studies | **7.1. Does the description of included studies give detailed information on sex/gender of the study samples?** • No, review did not meet criteria (At least 7.1.1. or 7.1.2. are NO) **7.1.1. Detailed information on SEX of the study samples** • Unable to determine **7.1.2. Detailed information on GENDER of the study samples** • No, review did not meet criteria |
|  |  |
| **Item** | **Brand (2013)** |
| Background | **1.1 Are the terms sex and gender used in the background?** • No, review did not meet criteria **1.2 Are sex/gender identified as relevant or not to review question?** • No, review did not meet criteria **1.3. Does background discuss why sex/gender differences may be expected?** • No, review did not meet criteria |
| Objectives | **2.1. Are the terms sex, gender, male, or female used in objectives?** • No, review did not meet criteria *Quote: "OBJECTIVES: To assess whether there was a reduction in the incidence of infective complications following the administration of prophylactic antibiotics in penetrating abdominal trauma (wounds that enter the peritoneal cavity)."* |
| Criteria for inclusion/ exclusion | **3.1. Do the review’s inclusion-exclusion criteria consider sex-gender differences?** • No, review did not meet criteria *Quote: "Types of participants: Patients who had an isolated penetrating abdominal wound, were not on antibiotics, and had no evidence of intra-abdominal sepsis or any other focus of infection."* **3.2. Was there justification or explanation for the exclusion of some groups?** • No, review did not meet criteria |
| Methods | **4.1. Does the review examine whether outcome measures are different for males and females?** • No, review did not meet criteria **4.2. Did the review extract data by sex?** • No, review did not meet criteria **4.3. Did the review extract data on sex of withdrawals and dropouts?** • No, review did not meet criteria **4.4. In cases where sex/gender is used as a proxy for other measures (i.e., weight), is there an explanation for this approach?** • Item was not applicable to the review **4.5. Were any subgroup analyses completed?** • No, review did not meet criteria **4.6. Were subgroup analyses by sex completed?** • No, review did not meet criteria |
| Results and Analysis | **5.1. Do results distinguish between findings for males/females?** • Item was not applicable to the review **5.2. Does the review report conclusions (of effectiveness, efficacy, safety) that are different for men and women?**  • Item was not applicable to the review **5.3. If adverse effects are reported, is information sex disaggregated?** • Item was not applicable to the review **5.4. Does review note that subgroup analyses by sex could not be done?** • Item was not applicable to the review |
| Discussion and conclusion | **6.1. Does the review report that primary studies analysed or failed to analyse results by sex?** • Item was not applicable to the review **6.2. Does the review address sex/gender implications for clinical practice?** • No, review did not meet criteria **6.3. Does the review address sex/gender implications for policy and regulation?** • No, review did not meet criteria **6.4. Does the review address sex/gender implications for research?** • No, review did not meet criteria |
| Table of included studies | **7.1. Does the description of included studies give detailed information on sex/gender of the study samples?** • Item was not applicable to the review **7.1.1. Detailed information on SEX of the study samples** • Item was not applicable to the review **7.1.2. Detailed information on GENDER of the study samples** • Item was not applicable to the review |
|  |  |
| **Item** | **Brass (2015)** |
| Background | **1.1 Are the terms sex and gender used in the background?** • No, review did not meet criteria **1.2 Are sex/gender identified as relevant or not to review question?** • No, review did not meet criteria **1.3. Does background discuss why sex/gender differences may be expected?** • No, review did not meet criteria |
| Objectives | **2.1. Are the terms sex, gender, male, or female used in objectives?** • No, review did not meet criteria |
| Criteria for inclusion/ exclusion | **3.1. Do the review’s inclusion-exclusion criteria consider sex-gender differences?** • No, review did not meet criteria *Quote: "Types of participants: [...] We applied no restrictions with respect to specific population characteristics (such as age, gender, race or presence of a particular condition, for example, risk factors), [...]"* **3.2. Was there justification or explanation for the exclusion of some groups?** • No, review did not meet criteria |
| Methods | **4.1. Does the review examine whether outcome measures are different for males and females?** • No, review did not meet criteria **4.2. Did the review extract data by sex?** • No, review did not meet criteria **4.3. Did the review extract data on sex of withdrawals and dropouts?** • No, review did not meet criteria **4.4. In cases where sex/gender is used as a proxy for other measures (i.e., weight), is there an explanation for this approach?** • Item was not applicable to the review **4.5. Were any subgroup analyses completed?** • No, review did not meet criteria **4.6. Were subgroup analyses by sex completed?** • No, review did not meet criteria |
| Results and Analysis | **5.1. Do results distinguish between findings for males/females?** • No, review did not meet criteria **5.2. Does the review report conclusions (of effectiveness, efficacy, safety) that are different for men and women?**  • No, review did not meet criteria **5.3. If adverse effects are reported, is information sex disaggregated?** • No, review did not meet criteria **5.4. Does review note that subgroup analyses by sex could not be done?** • Item was not applicable to the review |
| Discussion and conclusion | **6.1. Does the review report that primary studies analysed or failed to analyse results by sex?** • No, review did not meet criteria **6.2. Does the review address sex/gender implications for clinical practice?** • No, review did not meet criteria **6.3. Does the review address sex/gender implications for policy and regulation?** • No, review did not meet criteria **6.4. Does the review address sex/gender implications for research?** • No, review did not meet criteria |
| Table of included studies | **7.1. Does the description of included studies give detailed information on sex/gender of the study samples?** • No, review did not meet criteria (At least 7.1.1. or 7.1.2. are NO) **7.1.1. Detailed information on SEX of the study samples** • No, review did not meet criteria **7.1.2. Detailed information on GENDER of the study samples** • No, review did not meet criteria |
|  |  |
| **Item** | **Bravo (2016)** |
| Background | **1.1 Are the terms sex and gender used in the background?** • No, review did not meet criteria **1.2 Are sex/gender identified as relevant or not to review question?** • No, review did not meet criteria **1.3. Does background discuss why sex/gender differences may be expected?** • No, review did not meet criteria |
| Objectives | **2.1. Are the terms sex, gender, male, or female used in objectives?** • No, review did not meet criteria |
| Criteria for inclusion/ exclusion | **3.1. Do the review’s inclusion-exclusion criteria consider sex-gender differences?** • No, review did not meet criteria **3.2. Was there justification or explanation for the exclusion of some groups?** • No, review did not meet criteria |
| Methods | **4.1. Does the review examine whether outcome measures are different for males and females?** • No, review did not meet criteria **4.2. Did the review extract data by sex?** • No, review did not meet criteria **4.3. Did the review extract data on sex of withdrawals and dropouts?** • No, review did not meet criteria **4.4. In cases where sex/gender is used as a proxy for other measures (i.e., weight), is there an explanation for this approach?** • Item was not applicable to the review **4.5. Were any subgroup analyses completed?** • No, review did not meet criteria **4.6. Were subgroup analyses by sex completed?** • No, review did not meet criteria |
| Results and Analysis | **5.1. Do results distinguish between findings for males/females?** • No, review did not meet criteria **5.2. Does the review report conclusions (of effectiveness, efficacy, safety) that are different for men and women?**  • No, review did not meet criteria **5.3. If adverse effects are reported, is information sex disaggregated?** • Item was not applicable to the review **5.4. Does review note that subgroup analyses by sex could not be done?** • Item was not applicable to the review |
| Discussion and conclusion | **6.1. Does the review report that primary studies analysed or failed to analyse results by sex?** • No, review did not meet criteria **6.2. Does the review address sex/gender implications for clinical practice?** • No, review did not meet criteria **6.3. Does the review address sex/gender implications for policy and regulation?** • No, review did not meet criteria **6.4. Does the review address sex/gender implications for research?** • No, review did not meet criteria |
| Table of included studies | **7.1. Does the description of included studies give detailed information on sex/gender of the study samples?** • No, review did not meet criteria (At least 7.1.1. or 7.1.2. are NO) **7.1.1. Detailed information on SEX of the study samples** • Unable to determine **7.1.2. Detailed information on GENDER of the study samples** • No, review did not meet criteria |
|  |  |
| **Item** | **Brignardello-Petersen (2015)** |
| Background | **1.1 Are the terms sex and gender used in the background?** • Yes, review met criteria *Quote: "Female patients accounted for 56.2% of those undergoing the procedure (Venugoplan 2012)".* **1.2 Are sex/gender identified as relevant or not to review question?** • Unable to determine **1.3. Does background discuss why sex/gender differences may be expected?** • No, review did not meet criteria *Quote: "Female patients accounted for 56.2% of those undergoing the procedure (Venugoplan 2012)".* |
| Objectives | **2.1. Are the terms sex, gender, male, or female used in objectives?** • No, review did not meet criteria *Quote: "To assess the effects of antibiotic prophylaxis for preventing SSI in people undergoing OS."* |
| Criteria for inclusion/ exclusion | **3.1. Do the review’s inclusion-exclusion criteria consider sex-gender differences?** • No, review did not meet criteria *Quote: "Types of participants: People of any age undergoing OS in any setting."* **3.2. Was there justification or explanation for the exclusion of some groups?** • No, review did not meet criteria |
| Methods | **4.1. Does the review examine whether outcome measures are different for males and females?** • No, review did not meet criteria **4.2. Did the review extract data by sex?** • No, review did not meet criteria **4.3. Did the review extract data on sex of withdrawals and dropouts?** • No, review did not meet criteria **4.4. In cases where sex/gender is used as a proxy for other measures (i.e., weight), is there an explanation for this approach?** • Item was not applicable to the review **4.5. Were any subgroup analyses completed?** • No, review did not meet criteria **4.6. Were subgroup analyses by sex completed?** • No, review did not meet criteria |
| Results and Analysis | **5.1. Do results distinguish between findings for males/females?** • No, review did not meet criteria **5.2. Does the review report conclusions (of effectiveness, efficacy, safety) that are different for men and women?**  • No, review did not meet criteria **5.3. If adverse effects are reported, is information sex disaggregated?** • No, review did not meet criteria **5.4. Does review note that subgroup analyses by sex could not be done?** • Item was not applicable to the review |
| Discussion and conclusion | **6.1. Does the review report that primary studies analysed or failed to analyse results by sex?** • No, review did not meet criteria **6.2. Does the review address sex/gender implications for clinical practice?** • No, review did not meet criteria **6.3. Does the review address sex/gender implications for policy and regulation?** • No, review did not meet criteria **6.4. Does the review address sex/gender implications for research?** • No, review did not meet criteria |
| Table of included studies | **7.1. Does the description of included studies give detailed information on sex/gender of the study samples?** • No, review did not meet criteria (At least 7.1.1. or 7.1.2. are NO) **7.1.1. Detailed information on SEX of the study samples** • Unable to determine **7.1.2. Detailed information on GENDER of the study samples** • No, review did not meet criteria |
|  |  |
| **Item** | **Cooper (2016)** |
| Background | **1.1 Are the terms sex and gender used in the background?** • Yes, review met criteria *Quote: "A study by Sørbye and colleagues found that, in Italy, 35.9% of male and 27.4% of female elderly home care clients used either an indwelling, intermittent or condom catheter. However, rates of use of these catheters were lower in men in Finland (2.9%) and in women in the Czech Republic (0.6%) (Sørbye 2009)."* **1.2 Are sex/gender identified as relevant or not to review question?** • Unable to determine *Quote: "[..] a study by Sørbye and colleagues found that, in Italy, 35.9% of male and 27.4% of female elderly home care clients used either an indwelling, intermittent or condom catheter. However, rates of use of these catheters were lower in men in Finland (2.9%) and in women in the Czech Republic (0.6%) (Sørbye 2009)." Comment: Not clear if the review identified sex/gender as relevant to the review question.* **1.3. Does background discuss why sex/gender differences may be expected?** • No, review did not meet criteria *Quote: "[..] a study by Sørbye and colleagues found that, in Italy, 35.9% of male and 27.4% of female elderly home care clients used either an indwelling, intermittent or condom catheter. However, rates of use of these catheters were lower in men in Finland (2.9%) and in women in the Czech Republic (0.6%) (Sørbye 2009)." Comment: no explicit explanation about sex/gender differences.* |
| Objectives | **2.1. Are the terms sex, gender, male, or female used in objectives?** • No, review did not meet criteria *Quote: "OBJECTIVES: To determine the effectiveness of different policies for replacing long-term indwelling urinary catheters in adults"* |
| Criteria for inclusion/ exclusion | **3.1. Do the review’s inclusion-exclusion criteria consider sex-gender differences?** • No, review did not meet criteria *Quote: "Types of participants: Adults (over the age of 18 years) with long-term (> 14 days) indwelling urinary or suprapubic catheters that are anticipated to require replacement."* **3.2. Was there justification or explanation for the exclusion of some groups?** • No, review did not meet criteria |
| Methods | **4.1. Does the review examine whether outcome measures are different for males and females?** • No, review did not meet criteria **4.2. Did the review extract data by sex?** • No, review did not meet criteria **4.3. Did the review extract data on sex of withdrawals and dropouts?** • No, review did not meet criteria **4.4. In cases where sex/gender is used as a proxy for other measures (i.e., weight), is there an explanation for this approach?** • Item was not applicable to the review **4.5. Were any subgroup analyses completed?** • No, review did not meet criteria **4.6. Were subgroup analyses by sex completed?** • No, review did not meet criteria |
| Results and Analysis | **5.1. Do results distinguish between findings for males/females?** • No, review did not meet criteria **5.2. Does the review report conclusions (of effectiveness, efficacy, safety) that are different for men and women?**  • No, review did not meet criteria **5.3. If adverse effects are reported, is information sex disaggregated?** • No, review did not meet criteria **5.4. Does review note that subgroup analyses by sex could not be done?** • Yes, review met criteria |
| Discussion and conclusion | **6.1. Does the review report that primary studies analysed or failed to analyse results by sex?** • No, review did not meet criteria **6.2. Does the review address sex/gender implications for clinical practice?** • No, review did not meet criteria **6.3. Does the review address sex/gender implications for policy and regulation?** • No, review did not meet criteria **6.4. Does the review address sex/gender implications for research?** • Yes, review met criteria *Quote: "Implications for research: "[...] Sub-group analysis would give valuable data as to whether certain policies are more effective in sub-groups such as females or younger participants. We did not identify any long-term follow-up data. It is paramount that future trials report long-term follow-up data as this is also valuable evidence."* |
| Table of included studies | **7.1. Does the description of included studies give detailed information on sex/gender of the study samples?** • No, review did not meet criteria (At least 7.1.1. or 7.1.2. are NO) **7.1.1. Detailed information on SEX of the study samples** • No, review did not meet criteria **7.1.2. Detailed information on GENDER of the study samples** • No, review did not meet criteria |
|  |  |
| **Item** | **D'Amico (2009)** |
| Background | **1.1 Are the terms sex and gender used in the background?** • No, review did not meet criteria **1.2 Are sex/gender identified as relevant or not to review question?** • No, review did not meet criteria **1.3. Does background discuss why sex/gender differences may be expected?** • No, review did not meet criteria |
| Objectives | **2.1. Are the terms sex, gender, male, or female used in objectives?** • No, review did not meet criteria *Quote: "OBJECTIVES: To determine whether antibiotic prophylaxis reduces RTIs and overall mortality in adult patients treated in ICUs. Specifically, the main question left unanswered by existing randomised controlled trials (RCTs) and previous meta-analyses was whether different forms of antibiotic prophylaxis (that is, topical antimicrobials or a combination of topical and systemic drugs) are effective in reducing overall mortality."* |
| Criteria for inclusion/ exclusion | **3.1. Do the review’s inclusion-exclusion criteria consider sex-gender differences?** • No, review did not meet criteria *Quote: "Types of participants: Adult patients admitted to an ICU. Studies based on specific preselected types of patients (that is, patients undergoing elective oesophageal resection, cardiac or gastric surgery, liver transplant or suffering from acute liver failure) were excluded because these patients need co-interventions that may interact with the main treatment. Studies where the majority of patients (> 50%) did not undergo mechanical ventilation for more than 48 hours were also excluded. The characteristics of excluded studies are reported in the ’Characteristics of excluded studies’ table."* **3.2. Was there justification or explanation for the exclusion of some groups?** • No, review did not meet criteria |
| Methods | **4.1. Does the review examine whether outcome measures are different for males and females?** • No, review did not meet criteria **4.2. Did the review extract data by sex?** • No, review did not meet criteria **4.3. Did the review extract data on sex of withdrawals and dropouts?** • No, review did not meet criteria **4.4. In cases where sex/gender is used as a proxy for other measures (i.e., weight), is there an explanation for this approach?** • Item was not applicable to the review **4.5. Were any subgroup analyses completed?** • Yes, review met criteria *Quote: "Subgroup analysis and investigation of heterogeneity: Two pre-specified subgroup analyses based on quality criteria were carried out within the two main groups of RCTs specified above: • quality of randomisation procedures; and • blinding of patients and doctors to allocated treatment."* **4.6. Were subgroup analyses by sex completed?** • No, review did not meet criteria |
| Results and Analysis | **5.1. Do results distinguish between findings for males/females?** • No, review did not meet criteria **5.2. Does the review report conclusions (of effectiveness, efficacy, safety) that are different for men and women?**  • No, review did not meet criteria **5.3. If adverse effects are reported, is information sex disaggregated?** • Item was not applicable to the review **5.4. Does review note that subgroup analyses by sex could not be done?** • Item was not applicable to the review |
| Discussion and conclusion | **6.1. Does the review report that primary studies analysed or failed to analyse results by sex?** • No, review did not meet criteria **6.2. Does the review address sex/gender implications for clinical practice?** • No, review did not meet criteria **6.3. Does the review address sex/gender implications for policy and regulation?** • No, review did not meet criteria **6.4. Does the review address sex/gender implications for research?** • No, review did not meet criteria |
| Table of included studies | **7.1. Does the description of included studies give detailed information on sex/gender of the study samples?** • No, review did not meet criteria (At least 7.1.1. or 7.1.2. are NO) **7.1.1. Detailed information on SEX of the study samples** • No, review did not meet criteria **7.1.2. Detailed information on GENDER of the study samples** • No, review did not meet criteria |
|  |  |
| **Item** | **Dhiwakar (2012)** |
| Background | **1.1 Are the terms sex and gender used in the background?** • No, review did not meet criteria **1.2 Are sex/gender identified as relevant or not to review question?** • No, review did not meet criteria **1.3. Does background discuss why sex/gender differences may be expected?** • No, review did not meet criteria |
| Objectives | **2.1. Are the terms sex, gender, male, or female used in objectives?** • No, review did not meet criteria *Quote: "OBJECTIVES: To determine whether perioperative antibiotics reduce pain, associated morbidity and complications following tonsillectomy."* |
| Criteria for inclusion/ exclusion | **3.1. Do the review’s inclusion-exclusion criteria consider sex-gender differences?** • No, review did not meet criteria **3.2. Was there justification or explanation for the exclusion of some groups?** • No, review did not meet criteria |
| Methods | **4.1. Does the review examine whether outcome measures are different for males and females?** • No, review did not meet criteria **4.2. Did the review extract data by sex?** • No, review did not meet criteria *Quote: "Data extraction: Two authors independently extracted data (MD and AC) and separately entered these into a specific, pre-designed pro forma. One author (MD) then entered data into RevMan (RevMan 2011) for analysis."* **4.3. Did the review extract data on sex of withdrawals and dropouts?** • No, review did not meet criteria **4.4. In cases where sex/gender is used as a proxy for other measures (i.e., weight), is there an explanation for this approach?** • Item was not applicable to the review **4.5. Were any subgroup analyses completed?** • No, review did not meet criteria **4.6. Were subgroup analyses by sex completed?** • No, review did not meet criteria |
| Results and Analysis | **5.1. Do results distinguish between findings for males/females?** • No, review did not meet criteria **5.2. Does the review report conclusions (of effectiveness, efficacy, safety) that are different for men and women?**  • No, review did not meet criteria **5.3. If adverse effects are reported, is information sex disaggregated?** • No, review did not meet criteria **5.4. Does review note that subgroup analyses by sex could not be done?** • Item was not applicable to the review |
| Discussion and conclusion | **6.1. Does the review report that primary studies analysed or failed to analyse results by sex?** • No, review did not meet criteria **6.2. Does the review address sex/gender implications for clinical practice?** • No, review did not meet criteria **6.3. Does the review address sex/gender implications for policy and regulation?** • No, review did not meet criteria **6.4. Does the review address sex/gender implications for research?** • No, review did not meet criteria |
| Table of included studies | **7.1. Does the description of included studies give detailed information on sex/gender of the study samples?** • No, review did not meet criteria (At least 7.1.1. or 7.1.2. are NO) **7.1.1. Detailed information on SEX of the study samples** • No, review did not meet criteria *Quote: "Study and control groups were well matched in terms of age, sex and number of episodes of tonsillitis prior to surgery." Comment: Only reports comparability of study groups regarding sex.* **7.1.2. Detailed information on GENDER of the study samples** • No, review did not meet criteria |
|  |  |
| **Item** | **Dumville (2015)** |
| Background | **1.1 Are the terms sex and gender used in the background?** • No, review did not meet criteria **1.2 Are sex/gender identified as relevant or not to review question?** • No, review did not meet criteria **1.3. Does background discuss why sex/gender differences may be expected?** • No, review did not meet criteria |
| Objectives | **2.1. Are the terms sex, gender, male, or female used in objectives?** • No, review did not meet criteria *Quote: "OBJECTIVES: To determine whether preoperative skin antisepsis immediately prior to incision prevents SSI and to determine the comparative effectiveness of alternative antiseptics."* |
| Criteria for inclusion/ exclusion | **3.1. Do the review’s inclusion-exclusion criteria consider sex-gender differences?** • No, review did not meet criteria *Quote: "Types of participants: People of any age undergoing clean surgery. For the purposes of this review the CDC definition of a clean surgical wound was applied (Mangram 1999). Settings were not limited to a specific clinical area as clean surgery can take place in a variety of environments."* **3.2. Was there justification or explanation for the exclusion of some groups?** • No, review did not meet criteria |
| Methods | **4.1. Does the review examine whether outcome measures are different for males and females?** • No, review did not meet criteria **4.2. Did the review extract data by sex?** • No, review did not meet criteria **4.3. Did the review extract data on sex of withdrawals and dropouts?** • No, review did not meet criteria **4.4. In cases where sex/gender is used as a proxy for other measures (i.e., weight), is there an explanation for this approach?** • Item was not applicable to the review **4.5. Were any subgroup analyses completed?** • No, review did not meet criteria **4.6. Were subgroup analyses by sex completed?** • No, review did not meet criteria |
| Results and Analysis | **5.1. Do results distinguish between findings for males/females?** • No, review did not meet criteria **5.2. Does the review report conclusions (of effectiveness, efficacy, safety) that are different for men and women?**  • No, review did not meet criteria **5.3. If adverse effects are reported, is information sex disaggregated?** • Item was not applicable to the review **5.4. Does review note that subgroup analyses by sex could not be done?** • Item was not applicable to the review |
| Discussion and conclusion | **6.1. Does the review report that primary studies analysed or failed to analyse results by sex?** • No, review did not meet criteria **6.2. Does the review address sex/gender implications for clinical practice?** • No, review did not meet criteria **6.3. Does the review address sex/gender implications for policy and regulation?** • No, review did not meet criteria **6.4. Does the review address sex/gender implications for research?** • No, review did not meet criteria |
| Table of included studies | **7.1. Does the description of included studies give detailed information on sex/gender of the study samples?** • No, review did not meet criteria (At least 7.1.1. or 7.1.2. are NO) **7.1.1. Detailed information on SEX of the study samples** • No, review did not meet criteria **7.1.2. Detailed information on GENDER of the study samples** • No, review did not meet criteria |
|  |  |
| **Item** | **Dumville (2016)** |
| Background | **1.1 Are the terms sex and gender used in the background?** • No, review did not meet criteria **1.2 Are sex/gender identified as relevant or not to review question?** • No, review did not meet criteria **1.3. Does background discuss why sex/gender differences may be expected?** • No, review did not meet criteria |
| Objectives | **2.1. Are the terms sex, gender, male, or female used in objectives?** • No, review did not meet criteria *Quote: "To assess the effects of wound dressings compared with no wound dressings, and the effects of alternative wound dressings, in preventing SSIs in surgical wounds healing by primary intention."* |
| Criteria for inclusion/ exclusion | **3.1. Do the review’s inclusion-exclusion criteria consider sex-gender differences?** • No, review did not meet criteria *Quote: "Types of participants: Studies involving adults or children (aged two years and over) who had undergone surgical procedures where healing of the surgical wound was planned by primary intention. Wounds of any contamination level (clean, clean contaminated, contaminated and dirty) were eligible for inclusion. We excluded procedures involving graft sites, and wounds of the mouth and eye. Participants were required to have dressings applied in the operating theatre, immediately after closure of the skin. We excluded studies where participants had infected wounds at the start of the study."* **3.2. Was there justification or explanation for the exclusion of some groups?** • No, review did not meet criteria |
| Methods | **4.1. Does the review examine whether outcome measures are different for males and females?** • No, review did not meet criteria **4.2. Did the review extract data by sex?** • No, review did not meet criteria **4.3. Did the review extract data on sex of withdrawals and dropouts?** • No, review did not meet criteria **4.4. In cases where sex/gender is used as a proxy for other measures (i.e., weight), is there an explanation for this approach?** • Item was not applicable to the review **4.5. Were any subgroup analyses completed?** • No, review did not meet criteria **4.6. Were subgroup analyses by sex completed?** • No, review did not meet criteria |
| Results and Analysis | **5.1. Do results distinguish between findings for males/females?** • No, review did not meet criteria **5.2. Does the review report conclusions (of effectiveness, efficacy, safety) that are different for men and women?**  • No, review did not meet criteria **5.3. If adverse effects are reported, is information sex disaggregated?** • Item was not applicable to the review **5.4. Does review note that subgroup analyses by sex could not be done?** • Item was not applicable to the review |
| Discussion and conclusion | **6.1. Does the review report that primary studies analysed or failed to analyse results by sex?** • No, review did not meet criteria **6.2. Does the review address sex/gender implications for clinical practice?** • No, review did not meet criteria **6.3. Does the review address sex/gender implications for policy and regulation?** • No, review did not meet criteria **6.4. Does the review address sex/gender implications for research?** • No, review did not meet criteria |
| Table of included studies | **7.1. Does the description of included studies give detailed information on sex/gender of the study samples?** • No, review did not meet criteria (At least 7.1.1. or 7.1.2. are NO) **7.1.1. Detailed information on SEX of the study samples** • No, review did not meet criteria **7.1.2. Detailed information on GENDER of the study samples** • No, review did not meet criteria |
|  |  |
| **Item** | **Ejemot-Nwadiaro (2015)** |
| Background | **1.1 Are the terms sex and gender used in the background?** • No, review did not meet criteria **1.2 Are sex/gender identified as relevant or not to review question?** • No, review did not meet criteria **1.3. Does background discuss why sex/gender differences may be expected?** • No, review did not meet criteria |
| Objectives | **2.1. Are the terms sex, gender, male, or female used in objectives?** • No, review did not meet criteria *Quote: "To assess the effects of hand washing promotion interventions on diarrhoeal episodes in children and adults."* |
| Criteria for inclusion/ exclusion | **3.1. Do the review’s inclusion-exclusion criteria consider sex-gender differences?** • No, review did not meet criteria *Quote: "Types of participants: Individuals (adults and children) in day-care centres or schools, patients in hospitals, communities, or households."* **3.2. Was there justification or explanation for the exclusion of some groups?** • No, review did not meet criteria |
| Methods | **4.1. Does the review examine whether outcome measures are different for males and females?** • No, review did not meet criteria **4.2. Did the review extract data by sex?** • No, review did not meet criteria **4.3. Did the review extract data on sex of withdrawals and dropouts?** • No, review did not meet criteria **4.4. In cases where sex/gender is used as a proxy for other measures (i.e., weight), is there an explanation for this approach?** • Item was not applicable to the review **4.5. Were any subgroup analyses completed?** • Yes, review met criteria *Quote: "We planned to explore the possible causes of heterogeneity if we detected any using subgroup analysis. The subgroups used were: trial setting, provision of hand washing material (soap) as part of intervention, type of promotional activity employed), and quality characteristics (whether outcome assessors were blinded)." Comment: Subgroup analysis done.*  **4.6. Were subgroup analyses by sex completed?** • No, review did not meet criteria |
| Results and Analysis | **5.1. Do results distinguish between findings for males/females?** • No, review did not meet criteria **5.2. Does the review report conclusions (of effectiveness, efficacy, safety) that are different for men and women?**  • No, review did not meet criteria **5.3. If adverse effects are reported, is information sex disaggregated?** • Item was not applicable to the review **5.4. Does review note that subgroup analyses by sex could not be done?** • Item was not applicable to the review |
| Discussion and conclusion | **6.1. Does the review report that primary studies analysed or failed to analyse results by sex?** • No, review did not meet criteria **6.2. Does the review address sex/gender implications for clinical practice?** • No, review did not meet criteria **6.3. Does the review address sex/gender implications for policy and regulation?** • No, review did not meet criteria **6.4. Does the review address sex/gender implications for research?** • No, review did not meet criteria |
| Table of included studies | **7.1. Does the description of included studies give detailed information on sex/gender of the study samples?** • No, review did not meet criteria (At least 7.1.1. or 7.1.2. are NO) **7.1.1. Detailed information on SEX of the study samples** • No, review did not meet criteria **7.1.2. Detailed information on GENDER of the study samples** • No, review did not meet criteria |
|  |  |
| **Item** | **Fernandez (2012)** |
| Background | **1.1 Are the terms sex and gender used in the background?** • No, review did not meet criteria **1.2 Are sex/gender identified as relevant or not to review question?** • No, review did not meet criteria **1.3. Does background discuss why sex/gender differences may be expected?** • No, review did not meet criteria |
| Objectives | **2.1. Are the terms sex, gender, male, or female used in objectives?** • No, review did not meet criteria *Quote: "The objective of this review was to compare the effects of water (tap or cool, boiled or distilled) and saline for wound cleansing. The review will address the following questions. What are the comparative effects on rates of healing and infection in acute and chronic wounds, of the following cleansing solutions: • tap water compared with no cleansing; • tap water compared with sterile normal saline; • water (distilled and/or cooled boiled water) compared with sterile normal saline; • tap water compared with cooled boiled tap water; • tap water compared with any other solution."* |
| Criteria for inclusion/ exclusion | **3.1. Do the review’s inclusion-exclusion criteria consider sex-gender differences?** • No, review did not meet criteria *Quote: "Types of participants: Trials involving people of all ages with a wound of any aetiology, in any setting (hospital, community, nursing homes, general practice, wound clinics). For the purpose of the review a wound was defined as a break in the skin. We excluded trials if they compared solutions for dental procedures or for patients with burns."* **3.2. Was there justification or explanation for the exclusion of some groups?** • No, review did not meet criteria |
| Methods | **4.1. Does the review examine whether outcome measures are different for males and females?** • No, review did not meet criteria **4.2. Did the review extract data by sex?** • No, review did not meet criteria **4.3. Did the review extract data on sex of withdrawals and dropouts?** • No, review did not meet criteria **4.4. In cases where sex/gender is used as a proxy for other measures (i.e., weight), is there an explanation for this approach?** • Item was not applicable to the review **4.5. Were any subgroup analyses completed?** • No, review did not meet criteria **4.6. Were subgroup analyses by sex completed?** • No, review did not meet criteria |
| Results and Analysis | **5.1. Do results distinguish between findings for males/females?** • No, review did not meet criteria **5.2. Does the review report conclusions (of effectiveness, efficacy, safety) that are different for men and women?**  • No, review did not meet criteria **5.3. If adverse effects are reported, is information sex disaggregated?** • Item was not applicable to the review **5.4. Does review note that subgroup analyses by sex could not be done?** • Item was not applicable to the review |
| Discussion and conclusion | **6.1. Does the review report that primary studies analysed or failed to analyse results by sex?** • No, review did not meet criteria **6.2. Does the review address sex/gender implications for clinical practice?** • No, review did not meet criteria **6.3. Does the review address sex/gender implications for policy and regulation?** • No, review did not meet criteria **6.4. Does the review address sex/gender implications for research?** • No, review did not meet criteria |
| Table of included studies | **7.1. Does the description of included studies give detailed information on sex/gender of the study samples?** • No, review did not meet criteria (At least 7.1.1. or 7.1.2. are NO) **7.1.1. Detailed information on SEX of the study samples** • No, review did not meet criteria **7.1.2. Detailed information on GENDER of the study samples** • No, review did not meet criteria *Quote: "Both groups comparable for age however comparability for gender not stated". Comment: only for one study it was reported comparability of study groups was not known.* |
|  |  |
| **Item** | **Flodgren (2013)** |
| Background | **1.1 Are the terms sex and gender used in the background?** • Yes, review met criteria *Quote: "Specific risk factors for catheter-associated urinary tract infections (CAUTIs) are: female sex, [...]"* **1.2 Are sex/gender identified as relevant or not to review question?** • Yes, review met criteria *Quote: "Specific risk factors for catheter-associated urinary tract infections (CAUTIs) are: female sex, […]"* **1.3. Does background discuss why sex/gender differences may be expected?** • No, review did not meet criteria *Quote: "Specific risk factors for catheter-associated urinary tract infections (CAUTIs) are: female sex, […]"* |
| Objectives | **2.1. Are the terms sex, gender, male, or female used in objectives?** • No, review did not meet criteria *Quote: "To assess the effectiveness of different interventions, alone or in combination, which target healthcare professionals or healthcare organisations to improve professional adherence to infection control guidelines on device-related infection rates and measures of adherence. The specific objectives are to determine the effectiveness of interventions targeting health professionals or the organisation of healthcare in order to: 1. avoid the use of invasive medical devices; 2. reduce the duration of invasive medical device use; and 3. improve the adoption of adequate procedures for insertion, and maintenance of invasive medical devices, and thereby the prevention of device-related infections."* |
| Criteria for inclusion/ exclusion | **3.1. Do the review’s inclusion-exclusion criteria consider sex-gender differences?** • No, review did not meet criteria *Quote: "Types of participants: Healthcare professionals involved with the insertion or the maintenance of invasive devices, or both."* **3.2. Was there justification or explanation for the exclusion of some groups?** • No, review did not meet criteria |
| Methods | **4.1. Does the review examine whether outcome measures are different for males and females?** • No, review did not meet criteria **4.2. Did the review extract data by sex?** • No, review did not meet criteria **4.3. Did the review extract data on sex of withdrawals and dropouts?** • No, review did not meet criteria **4.4. In cases where sex/gender is used as a proxy for other measures (i.e., weight), is there an explanation for this approach?** • Item was not applicable to the review **4.5. Were any subgroup analyses completed?** • No, review did not meet criteria **4.6. Were subgroup analyses by sex completed?** • No, review did not meet criteria |
| Results and Analysis | **5.1. Do results distinguish between findings for males/females?** • No, review did not meet criteria **5.2. Does the review report conclusions (of effectiveness, efficacy, safety) that are different for men and women?**  • No, review did not meet criteria **5.3. If adverse effects are reported, is information sex disaggregated?** • Item was not applicable to the review **5.4. Does review note that subgroup analyses by sex could not be done?** • Item was not applicable to the review |
| Discussion and conclusion | **6.1. Does the review report that primary studies analysed or failed to analyse results by sex?** • No, review did not meet criteria **6.2. Does the review address sex/gender implications for clinical practice?** • No, review did not meet criteria **6.3. Does the review address sex/gender implications for policy and regulation?** • No, review did not meet criteria **6.4. Does the review address sex/gender implications for research?** • No, review did not meet criteria |
| Table of included studies | **7.1. Does the description of included studies give detailed information on sex/gender of the study samples?** • No, review did not meet criteria (At least 7.1.1. or 7.1.2. are NO) **7.1.1. Detailed information on SEX of the study samples** • No, review did not meet criteria **7.1.2. Detailed information on GENDER of the study samples** • Unable to determine |
|  |  |
| **Item** | **Flodgren (2016)** |
| Background | **1.1 Are the terms sex and gender used in the background?** • No, review did not meet criteria **1.2 Are sex/gender identified as relevant or not to review question?** • No, review did not meet criteria **1.3. Does background discuss why sex/gender differences may be expected?** • No, review did not meet criteria |
| Objectives | **2.1. Are the terms sex, gender, male, or female used in objectives?** • No, review did not meet criteria |
| Criteria for inclusion/ exclusion | **3.1. Do the review’s inclusion-exclusion criteria consider sex-gender differences?** • No, review did not meet criteria **3.2. Was there justification or explanation for the exclusion of some groups?** • No, review did not meet criteria |
| Methods | **4.1. Does the review examine whether outcome measures are different for males and females?** • No, review did not meet criteria **4.2. Did the review extract data by sex?** • No, review did not meet criteria **4.3. Did the review extract data on sex of withdrawals and dropouts?** • No, review did not meet criteria **4.4. In cases where sex/gender is used as a proxy for other measures (i.e., weight), is there an explanation for this approach?** • Item was not applicable to the review **4.5. Were any subgroup analyses completed?** • No, review did not meet criteria **4.6. Were subgroup analyses by sex completed?** • No, review did not meet criteria |
| Results and Analysis | **5.1. Do results distinguish between findings for males/females?** • No, review did not meet criteria **5.2. Does the review report conclusions (of effectiveness, efficacy, safety) that are different for men and women?**  • No, review did not meet criteria **5.3. If adverse effects are reported, is information sex disaggregated?** • Item was not applicable to the review **5.4. Does review note that subgroup analyses by sex could not be done?** • Item was not applicable to the review |
| Discussion and conclusion | **6.1. Does the review report that primary studies analysed or failed to analyse results by sex?** • No, review did not meet criteria **6.2. Does the review address sex/gender implications for clinical practice?** • No, review did not meet criteria **6.3. Does the review address sex/gender implications for policy and regulation?** • No, review did not meet criteria **6.4. Does the review address sex/gender implications for research?** • No, review did not meet criteria |
| Table of included studies | **7.1. Does the description of included studies give detailed information on sex/gender of the study samples?** • No, review did not meet criteria (At least 7.1.1. or 7.1.2. are NO) **7.1.1. Detailed information on SEX of the study samples** • No, review did not meet criteria **7.1.2. Detailed information on GENDER of the study samples** • No, review did not meet criteria |
|  |  |
| **Item** | **Foon (2012)** |
| Background | **1.1 Are the terms sex and gender used in the background?** • Yes, review met criteria *Quote: "Urodynamic studies are used to detect dysfunction of the lower urinary tract in men, women or children with urinary symptoms such as frequency, urgency, incontinence, voiding difficulties etc".* **1.2 Are sex/gender identified as relevant or not to review question?** • No, review did not meet criteria *Quote: "Urodynamic studies are used to detect dysfunction of the lower urinary tract in men, women or children with urinary symptoms such as frequency, urgency, incontinence, voiding difficulties etc".* **1.3. Does background discuss why sex/gender differences may be expected?** • No, review did not meet criteria *Quote: "Urodynamic studies are used to detect dysfunction of the lower urinary tract in men, women or children with urinary symptoms such as frequency, urgency, incontinence, voiding difficulties etc".* |
| Objectives | **2.1. Are the terms sex, gender, male, or female used in objectives?** • No, review did not meet criteria *Quote: "The rationale for the review is that there is uncertainty as to whether prophylactic antibiotics are effective in preventing UTI and a systematic review of randomized controlled trials (RCTs) is needed to address this question. It is unclear whether antibiotics should be given to everybody or should be confined to at risk groups and what the best drug is if antibiotics are to be prescribed for prophylaxis of UTI. The objective of this systematic review was to assess the effectiveness and safety of administering prophylactic antibiotics in reducing the risk of UTI after urodynamic studies. The following comparisons were planned. 1. Antibiotics versus placebo or no antibiotics. 2. One antibiotic versus another. 3. One dose of antibiotics versus another dose. 4. One duration of antibiotic use versus another duration. 5. One route of administration of antibiotics versus another. 6. Antibiotics versus other treatments (e.g. increased fluid intake, cranberry juice, urinary antiseptics etc)."* |
| Criteria for inclusion/ exclusion | **3.1. Do the review’s inclusion-exclusion criteria consider sex-gender differences?** • No, review did not meet criteria *Quote: "Types of participants: Adult males, females or children undergoing urodynamic studies irrespective of whether they had spinal cord injury or a suprapubic catheter."* **3.2. Was there justification or explanation for the exclusion of some groups?** • No, review did not meet criteria |
| Methods | **4.1. Does the review examine whether outcome measures are different for males and females?** • No, review did not meet criteria **4.2. Did the review extract data by sex?** • No, review did not meet criteria **4.3. Did the review extract data on sex of withdrawals and dropouts?** • No, review did not meet criteria **4.4. In cases where sex/gender is used as a proxy for other measures (i.e., weight), is there an explanation for this approach?** • Item was not applicable to the review **4.5. Were any subgroup analyses completed?** • No, review did not meet criteria **4.6. Were subgroup analyses by sex completed?** • No, review did not meet criteria |
| Results and Analysis | **5.1. Do results distinguish between findings for males/females?** • No, review did not meet criteria **5.2. Does the review report conclusions (of effectiveness, efficacy, safety) that are different for men and women?**  • No, review did not meet criteria **5.3. If adverse effects are reported, is information sex disaggregated?** • No, review did not meet criteria **5.4. Does review note that subgroup analyses by sex could not be done?** • Yes, review met criteria |
| Discussion and conclusion | **6.1. Does the review report that primary studies analysed or failed to analyse results by sex?** • No, review did not meet criteria **6.2. Does the review address sex/gender implications for clinical practice?** • No, review did not meet criteria **6.3. Does the review address sex/gender implications for policy and regulation?** • No, review did not meet criteria **6.4. Does the review address sex/gender implications for research?** • No, review did not meet criteria |
| Table of included studies | **7.1. Does the description of included studies give detailed information on sex/gender of the study samples?** • No, review did not meet criteria (At least 7.1.1. or 7.1.2. are NO) **7.1.1. Detailed information on SEX of the study samples** • No, review did not meet criteria **7.1.2. Detailed information on GENDER of the study samples** • No, review did not meet criteria |
|  |  |
| **Item** | **Gavin (2016)** |
| Background | **1.1 Are the terms sex and gender used in the background?** • No, review did not meet criteria **1.2 Are sex/gender identified as relevant or not to review question?** • No, review did not meet criteria **1.3. Does background discuss why sex/gender differences may be expected?** • No, review did not meet criteria |
| Objectives | **2.1. Are the terms sex, gender, male, or female used in objectives?** • No, review did not meet criteria |
| Criteria for inclusion/ exclusion | **3.1. Do the review’s inclusion-exclusion criteria consider sex-gender differences?** • No, review did not meet criteria **3.2. Was there justification or explanation for the exclusion of some groups?** • No, review did not meet criteria |
| Methods | **4.1. Does the review examine whether outcome measures are different for males and females?** • No, review did not meet criteria **4.2. Did the review extract data by sex?** • No, review did not meet criteria **4.3. Did the review extract data on sex of withdrawals and dropouts?** • No, review did not meet criteria **4.4. In cases where sex/gender is used as a proxy for other measures (i.e., weight), is there an explanation for this approach?** • Item was not applicable to the review **4.5. Were any subgroup analyses completed?** • No, review did not meet criteria **4.6. Were subgroup analyses by sex completed?** • No, review did not meet criteria |
| Results and Analysis | **5.1. Do results distinguish between findings for males/females?** • No, review did not meet criteria **5.2. Does the review report conclusions (of effectiveness, efficacy, safety) that are different for men and women?**  • No, review did not meet criteria **5.3. If adverse effects are reported, is information sex disaggregated?** • No, review did not meet criteria **5.4. Does review note that subgroup analyses by sex could not be done?** • Item was not applicable to the review |
| Discussion and conclusion | **6.1. Does the review report that primary studies analysed or failed to analyse results by sex?** • No, review did not meet criteria **6.2. Does the review address sex/gender implications for clinical practice?** • No, review did not meet criteria **6.3. Does the review address sex/gender implications for policy and regulation?** • No, review did not meet criteria **6.4. Does the review address sex/gender implications for research?** • No, review did not meet criteria |
| Table of included studies | **7.1. Does the description of included studies give detailed information on sex/gender of the study samples?** • No, review did not meet criteria (At least 7.1.1. or 7.1.2. are NO) **7.1.1. Detailed information on SEX of the study samples** • No, review did not meet criteria **7.1.2. Detailed information on GENDER of the study samples** • Unable to determine |
|  |  |
| **Item** | **Ge (2012)** |
| Background | **1.1 Are the terms sex and gender used in the background?** • No, review did not meet criteria **1.2 Are sex/gender identified as relevant or not to review question?** • No, review did not meet criteria **1.3. Does background discuss why sex/gender differences may be expected?** • No, review did not meet criteria |
| Objectives | **2.1. Are the terms sex, gender, male, or female used in objectives?** • No, review did not meet criteria *Quote: "1. Our primary objective was to establish whether the jugular, subclavian or femoral CVA routes resulted in a lower incidence of venous thrombosis, venous stenosis or infections related to CVA devices in adult patients. 2. Our secondary objective was to assess whether the jugular, subclavian or femoral CVA routes influenced the incidence of catheter-related mechanical complications in adult patients; and the reasons why patients left the studies early."* |
| Criteria for inclusion/ exclusion | **3.1. Do the review’s inclusion-exclusion criteria consider sex-gender differences?** • No, review did not meet criteria *Quote: "Types of participants: 1. We included adults over 16 years of age with any disease process requiring proposed intravenous therapy via the central venous route, irrespective of sex or severity of illness. 2. We excluded studies in children (aged less than 16 years) because CVC-related complications in paediatric patients have been closely linked to age, body size and age-related immune status, and the optimal site of insertion also depended on factors such as the paediatric patient’s age and the need for sedation and analgesia during the insertion procedure (De Jonge 2005)."* **3.2. Was there justification or explanation for the exclusion of some groups?** • No, review did not meet criteria |
| Methods | **4.1. Does the review examine whether outcome measures are different for males and females?** • No, review did not meet criteria **4.2. Did the review extract data by sex?** • No, review did not meet criteria **4.3. Did the review extract data on sex of withdrawals and dropouts?** • No, review did not meet criteria **4.4. In cases where sex/gender is used as a proxy for other measures (i.e., weight), is there an explanation for this approach?** • Item was not applicable to the review **4.5. Were any subgroup analyses completed?** • Yes, review met criteria *Quote: "1. Subgroup analysis: If data were available, we considered subgroup analysis on the basis of following. 1.1 Different duration of catheter placement: short term (< one month), long term (> one month) defined according to the FDA (FDA 1995). 1.2 Different usage of catheter (to administer medications or fluids, obtain blood tests, obtain cardiovascular measurements, or haemodialysis). 1.3 Influence of disease process, influence of vessels on either the right or left side. 1.4 Patients receiving anticoagulation agents (warfarin, low molecular weight heparin or conventional heparin)." Quote: "[...] but an a priori subgroup analysis for the effect of BMI on catheter colonization demonstrated a significant difference".* **4.6. Were subgroup analyses by sex completed?** • No, review did not meet criteria |
| Results and Analysis | **5.1. Do results distinguish between findings for males/females?** • No, review did not meet criteria **5.2. Does the review report conclusions (of effectiveness, efficacy, safety) that are different for men and women?**  • No, review did not meet criteria **5.3. If adverse effects are reported, is information sex disaggregated?** • No, review did not meet criteria **5.4. Does review note that subgroup analyses by sex could not be done?** • Item was not applicable to the review |
| Discussion and conclusion | **6.1. Does the review report that primary studies analysed or failed to analyse results by sex?** • No, review did not meet criteria **6.2. Does the review address sex/gender implications for clinical practice?** • No, review did not meet criteria **6.3. Does the review address sex/gender implications for policy and regulation?** • No, review did not meet criteria **6.4. Does the review address sex/gender implications for research?** • No, review did not meet criteria |
| Table of included studies | **7.1. Does the description of included studies give detailed information on sex/gender of the study samples?** • No, review did not meet criteria (At least 7.1.1. or 7.1.2. are NO) **7.1.1. Detailed information on SEX of the study samples** • Unable to determine **7.1.2. Detailed information on GENDER of the study samples** • No, review did not meet criteria |
|  |  |
| **Item** | **Gillespie (2010)** |
| Background | **1.1 Are the terms sex and gender used in the background?** • No, review did not meet criteria **1.2 Are sex/gender identified as relevant or not to review question?** • No, review did not meet criteria **1.3. Does background discuss why sex/gender differences may be expected?** • No, review did not meet criteria |
| Objectives | **2.1. Are the terms sex, gender, male, or female used in objectives?** • No, review did not meet criteria |
| Criteria for inclusion/ exclusion | **3.1. Do the review’s inclusion-exclusion criteria consider sex-gender differences?** • No, review did not meet criteria *Quote: "Types of participants: Any person undergoing surgery for internal fixation or replacement arthroplasty as treatment for a closed fracture of the proximal femur, or any other long bone."* **3.2. Was there justification or explanation for the exclusion of some groups?** • No, review did not meet criteria |
| Methods | **4.1. Does the review examine whether outcome measures are different for males and females?** • No, review did not meet criteria **4.2. Did the review extract data by sex?** • No, review did not meet criteria **4.3. Did the review extract data on sex of withdrawals and dropouts?** • No, review did not meet criteria **4.4. In cases where sex/gender is used as a proxy for other measures (i.e., weight), is there an explanation for this approach?** • Item was not applicable to the review **4.5. Were any subgroup analyses completed?** • Yes, review met criteria *Quote: "For the main outcome of interest, deep surgical site infection, the data were presented by subgroups to investigate any possible heterogeneity associated with participant context."* **4.6. Were subgroup analyses by sex completed?** • No, review did not meet criteria |
| Results and Analysis | **5.1. Do results distinguish between findings for males/females?** • No, review did not meet criteria **5.2. Does the review report conclusions (of effectiveness, efficacy, safety) that are different for men and women?**  • No, review did not meet criteria **5.3. If adverse effects are reported, is information sex disaggregated?** • No, review did not meet criteria **5.4. Does review note that subgroup analyses by sex could not be done?** • Item was not applicable to the review |
| Discussion and conclusion | **6.1. Does the review report that primary studies analysed or failed to analyse results by sex?** • No, review did not meet criteria **6.2. Does the review address sex/gender implications for clinical practice?** • No, review did not meet criteria **6.3. Does the review address sex/gender implications for policy and regulation?** • No, review did not meet criteria **6.4. Does the review address sex/gender implications for research?** • No, review did not meet criteria |
| Table of included studies | **7.1. Does the description of included studies give detailed information on sex/gender of the study samples?** • No, review did not meet criteria (At least 7.1.1. or 7.1.2. are NO) **7.1.1. Detailed information on SEX of the study samples** • No, review did not meet criteria **7.1.2. Detailed information on GENDER of the study samples** • Unable to determine |
|  |  |
| **Item** | **Glenny (2013)** |
| Background | **1.1 Are the terms sex and gender used in the background?** • No, review did not meet criteria **1.2 Are sex/gender identified as relevant or not to review question?** • No, review did not meet criteria **1.3. Does background discuss why sex/gender differences may be expected?** • No, review did not meet criteria |
| Objectives | **2.1. Are the terms sex, gender, male, or female used in objectives?** • No, review did not meet criteria |
| Criteria for inclusion/ exclusion | **3.1. Do the review’s inclusion-exclusion criteria consider sex-gender differences?** • No, review did not meet criteria **3.2. Was there justification or explanation for the exclusion of some groups?** • No, review did not meet criteria |
| Methods | **4.1. Does the review examine whether outcome measures are different for males and females?** • No, review did not meet criteria **4.2. Did the review extract data by sex?** • No, review did not meet criteria **4.3. Did the review extract data on sex of withdrawals and dropouts?** • No, review did not meet criteria **4.4. In cases where sex/gender is used as a proxy for other measures (i.e., weight), is there an explanation for this approach?** • Item was not applicable to the review **4.5. Were any subgroup analyses completed?** • No, review did not meet criteria *Quote: "Also, if appropriate, subgrouping would be used to explore the effects of different underlying causes of at risk and high risk status for endocarditis, and of different invasive dental techniques."* **4.6. Were subgroup analyses by sex completed?** • No, review did not meet criteria |
| Results and Analysis | **5.1. Do results distinguish between findings for males/females?** • No, review did not meet criteria **5.2. Does the review report conclusions (of effectiveness, efficacy, safety) that are different for men and women?**  • No, review did not meet criteria **5.3. If adverse effects are reported, is information sex disaggregated?** • Item was not applicable to the review **5.4. Does review note that subgroup analyses by sex could not be done?** • Item was not applicable to the review |
| Discussion and conclusion | **6.1. Does the review report that primary studies analysed or failed to analyse results by sex?** • No, review did not meet criteria *Quote: "Selection of appropriate controls is probably the most challenging aspect; ideally, as in Van der Meer’s study, they would have had dental treatment in a predefined time and be matched very closely for sex, age and type of cardiac risk factor." Comment: This statements is on the baseline characteristics, not on the analysis by sex.*  **6.2. Does the review address sex/gender implications for clinical practice?** • No, review did not meet criteria **6.3. Does the review address sex/gender implications for policy and regulation?** • No, review did not meet criteria **6.4. Does the review address sex/gender implications for research?** • No, review did not meet criteria |
| Table of included studies | **7.1. Does the description of included studies give detailed information on sex/gender of the study samples?** • No, review did not meet criteria (At least 7.1.1. or 7.1.2. are NO) **7.1.1. Detailed information on SEX of the study samples** • No, review did not meet criteria **7.1.2. Detailed information on GENDER of the study samples** • No, review did not meet criteria |
|  |  |
| **Item** | **Gosselin (2004)** |
| Background | **1.1 Are the terms sex and gender used in the background?** • No, review did not meet criteria **1.2 Are sex/gender identified as relevant or not to review question?** • No, review did not meet criteria **1.3. Does background discuss why sex/gender differences may be expected?** • No, review did not meet criteria |
| Objectives | **2.1. Are the terms sex, gender, male, or female used in objectives?** • No, review did not meet criteria |
| Criteria for inclusion/ exclusion | **3.1. Do the review’s inclusion-exclusion criteria consider sex-gender differences?** • No, review did not meet criteria **3.2. Was there justification or explanation for the exclusion of some groups?** • No, review did not meet criteria |
| Methods | **4.1. Does the review examine whether outcome measures are different for males and females?** • No, review did not meet criteria **4.2. Did the review extract data by sex?** • No, review did not meet criteria **4.3. Did the review extract data on sex of withdrawals and dropouts?** • No, review did not meet criteria **4.4. In cases where sex/gender is used as a proxy for other measures (i.e., weight), is there an explanation for this approach?** • Item was not applicable to the review **4.5. Were any subgroup analyses completed?** • Yes, review met criteria *Quote: "We planned three subgroup analyses exploring whether the effect differed between placebo-controlled and no-placebo studies, whether the effect of antimicrobials differed depending on the location of the fracture (specifically comparing use in phalangeal fractures in the hand with use in fractures of major limb bones), and whether the timing of antibiotic administration was a critical factor." Quote: "In the subgroup analysis, the lower risk of bias group had an absolute risk of wound infection of 0.13 (22169) for the controls and 0.04 (9201) for those receiving antibiotics (risk difference - 0.09 (95% CI -0.03 to -0.15), while the no-placebo group had an absolute risk of 0.11 (31292) for the controls and 0.05 (24 444) for those receiving antibiotics (risk difference -0.05 (95%CI -0.01 to -0.10). The difference between these subgroups was not statistically significant."* **4.6. Were subgroup analyses by sex completed?** • No, review did not meet criteria |
| Results and Analysis | **5.1. Do results distinguish between findings for males/females?** • No, review did not meet criteria **5.2. Does the review report conclusions (of effectiveness, efficacy, safety) that are different for men and women?**  • No, review did not meet criteria **5.3. If adverse effects are reported, is information sex disaggregated?** • Item was not applicable to the review **5.4. Does review note that subgroup analyses by sex could not be done?** • Item was not applicable to the review |
| Discussion and conclusion | **6.1. Does the review report that primary studies analysed or failed to analyse results by sex?** • No, review did not meet criteria **6.2. Does the review address sex/gender implications for clinical practice?** • No, review did not meet criteria **6.3. Does the review address sex/gender implications for policy and regulation?** • No, review did not meet criteria **6.4. Does the review address sex/gender implications for research?** • No, review did not meet criteria |
| Table of included studies | **7.1. Does the description of included studies give detailed information on sex/gender of the study samples?** • No, review did not meet criteria (At least 7.1.1. or 7.1.2. are NO) **7.1.1. Detailed information on SEX of the study samples** • No, review did not meet criteria **7.1.2. Detailed information on GENDER of the study samples** • No, review did not meet criteria |
|  |  |
| **Item** | **Gould (2010)** |
| Background | **1.1 Are the terms sex and gender used in the background?** • No, review did not meet criteria **1.2 Are sex/gender identified as relevant or not to review question?** • No, review did not meet criteria **1.3. Does background discuss why sex/gender differences may be expected?** • No, review did not meet criteria |
| Objectives | **2.1. Are the terms sex, gender, male, or female used in objectives?** • No, review did not meet criteria |
| Criteria for inclusion/ exclusion | **3.1. Do the review’s inclusion-exclusion criteria consider sex-gender differences?** • No, review did not meet criteria **3.2. Was there justification or explanation for the exclusion of some groups?** • No, review did not meet criteria |
| Methods | **4.1. Does the review examine whether outcome measures are different for males and females?** • No, review did not meet criteria **4.2. Did the review extract data by sex?** • No, review did not meet criteria **4.3. Did the review extract data on sex of withdrawals and dropouts?** • No, review did not meet criteria **4.4. In cases where sex/gender is used as a proxy for other measures (i.e., weight), is there an explanation for this approach?** • Item was not applicable to the review **4.5. Were any subgroup analyses completed?** • No, review did not meet criteria **4.6. Were subgroup analyses by sex completed?** • No, review did not meet criteria |
| Results and Analysis | **5.1. Do results distinguish between findings for males/females?** • No, review did not meet criteria **5.2. Does the review report conclusions (of effectiveness, efficacy, safety) that are different for men and women?**  • No, review did not meet criteria **5.3. If adverse effects are reported, is information sex disaggregated?** • Item was not applicable to the review **5.4. Does review note that subgroup analyses by sex could not be done?** • Item was not applicable to the review |
| Discussion and conclusion | **6.1. Does the review report that primary studies analysed or failed to analyse results by sex?** • No, review did not meet criteria **6.2. Does the review address sex/gender implications for clinical practice?** • No, review did not meet criteria **6.3. Does the review address sex/gender implications for policy and regulation?** • No, review did not meet criteria **6.4. Does the review address sex/gender implications for research?** • No, review did not meet criteria |
| Table of included studies | **7.1. Does the description of included studies give detailed information on sex/gender of the study samples?** • No, review did not meet criteria (At least 7.1.1. or 7.1.2. are NO) **7.1.1. Detailed information on SEX of the study samples** • No, review did not meet criteria **7.1.2. Detailed information on GENDER of the study samples** • No, review did not meet criteria |
|  |  |
| **Item** | **Griffiths (2007)** |
| Background | **1.1 Are the terms sex and gender used in the background?** • No, review did not meet criteria **1.2 Are sex/gender identified as relevant or not to review question?** • No, review did not meet criteria **1.3. Does background discuss why sex/gender differences may be expected?** • No, review did not meet criteria |
| Objectives | **2.1. Are the terms sex, gender, male, or female used in objectives?** • No, review did not meet criteria |
| Criteria for inclusion/ exclusion | **3.1. Do the review’s inclusion-exclusion criteria consider sex-gender differences?** • No, review did not meet criteria *Quote: "Types of participants: People of all ages having a short-term indwelling urethral catheter, in any setting (hospital, community, nursing home) were included in the review. For the purpose of this review a short-term indwelling catheter was defined as a catheter inserted for a period of 1 to 14 days (Dunn 2000). Participants with congenital abnormalities of the genitourinary system were excluded from the review."* **3.2. Was there justification or explanation for the exclusion of some groups?** • No, review did not meet criteria |
| Methods | **4.1. Does the review examine whether outcome measures are different for males and females?** • No, review did not meet criteria **4.2. Did the review extract data by sex?** • No, review did not meet criteria **4.3. Did the review extract data on sex of withdrawals and dropouts?** • No, review did not meet criteria **4.4. In cases where sex/gender is used as a proxy for other measures (i.e., weight), is there an explanation for this approach?** • Item was not applicable to the review **4.5. Were any subgroup analyses completed?** • Yes, review met criteria *Quote: "Planned subgroup analyses were undertaken to consider differences between the sexes and reasons for catheterisations. In addition, subgroup sensitivity analysis by different catheter sizes was planned but could not be undertaken due to the absence of data to allow this analysis."* **4.6. Were subgroup analyses by sex completed?** • Yes, review met criteria *Quote: "Planned subgroup analyses were undertaken to consider differences between the sexes and reasons for catheterisations." Comment: see Analysis 2.5. Comparison 2 SHORT VERSUS LONGER DURATION OF CATHETER USE, Outcome 5 Urinary Tract Infection (by gender). Comment: see Analysis 4.7. Comparison 4 CLAMPING VERSUS FREE DRAINAGE, Outcome 7 Incidence of postoperative dysfunction (by gender).* |
| Results and Analysis | **5.1. Do results distinguish between findings for males/females?** • No, review did not meet criteria **5.2. Does the review report conclusions (of effectiveness, efficacy, safety) that are different for men and women?**  • No, review did not meet criteria **5.3. If adverse effects are reported, is information sex disaggregated?** • No, review did not meet criteria **5.4. Does review note that subgroup analyses by sex could not be done?** • Item was not applicable to the review |
| Discussion and conclusion | **6.1. Does the review report that primary studies analysed or failed to analyse results by sex?** • No, review did not meet criteria **6.2. Does the review address sex/gender implications for clinical practice?** • No, review did not meet criteria **6.3. Does the review address sex/gender implications for policy and regulation?** • No, review did not meet criteria **6.4. Does the review address sex/gender implications for research?** • No, review did not meet criteria |
| Table of included studies | **7.1. Does the description of included studies give detailed information on sex/gender of the study samples?** • No, review did not meet criteria (At least 7.1.1. or 7.1.2. are NO) **7.1.1. Detailed information on SEX of the study samples** • No, review did not meet criteria *Quote: "101 female patients". Comment: information only for some studies.*  **7.1.2. Detailed information on GENDER of the study samples** • No, review did not meet criteria *Quote: "106 women". Comment: information only for some studies.* |
|  |  |
| **Item** | **Gurusamy (2011)** |
| Background | **1.1 Are the terms sex and gender used in the background?** • No, review did not meet criteria **1.2 Are sex/gender identified as relevant or not to review question?** • No, review did not meet criteria **1.3. Does background discuss why sex/gender differences may be expected?** • No, review did not meet criteria |
| Objectives | **2.1. Are the terms sex, gender, male, or female used in objectives?** • No, review did not meet criteria |
| Criteria for inclusion/ exclusion | **3.1. Do the review’s inclusion-exclusion criteria consider sex-gender differences?** • No, review did not meet criteria **3.2. Was there justification or explanation for the exclusion of some groups?** • No, review did not meet criteria |
| Methods | **4.1. Does the review examine whether outcome measures are different for males and females?** • No, review did not meet criteria **4.2. Did the review extract data by sex?** • No, review did not meet criteria **4.3. Did the review extract data on sex of withdrawals and dropouts?** • No, review did not meet criteria **4.4. In cases where sex/gender is used as a proxy for other measures (i.e., weight), is there an explanation for this approach?** • Item was not applicable to the review **4.5. Were any subgroup analyses completed?** • No, review did not meet criteria **4.6. Were subgroup analyses by sex completed?** • No, review did not meet criteria |
| Results and Analysis | **5.1. Do results distinguish between findings for males/females?** • No, review did not meet criteria **5.2. Does the review report conclusions (of effectiveness, efficacy, safety) that are different for men and women?**  • No, review did not meet criteria **5.3. If adverse effects are reported, is information sex disaggregated?** • No, review did not meet criteria **5.4. Does review note that subgroup analyses by sex could not be done?** • Item was not applicable to the review |
| Discussion and conclusion | **6.1. Does the review report that primary studies analysed or failed to analyse results by sex?** • No, review did not meet criteria **6.2. Does the review address sex/gender implications for clinical practice?** • No, review did not meet criteria **6.3. Does the review address sex/gender implications for policy and regulation?** • No, review did not meet criteria **6.4. Does the review address sex/gender implications for research?** • No, review did not meet criteria |
| Table of included studies | **7.1. Does the description of included studies give detailed information on sex/gender of the study samples?** • No, review did not meet criteria (At least 7.1.1. or 7.1.2. are NO) **7.1.1. Detailed information on SEX of the study samples** • Unable to determine **7.1.2. Detailed information on GENDER of the study samples** • No, review did not meet criteria |
|  |  |
| **Item** | **Gurusamy (2013)** |
| Background | **1.1 Are the terms sex and gender used in the background?** • No, review did not meet criteria **1.2 Are sex/gender identified as relevant or not to review question?** • No, review did not meet criteria **1.3. Does background discuss why sex/gender differences may be expected?** • No, review did not meet criteria |
| Objectives | **2.1. Are the terms sex, gender, male, or female used in objectives?** • No, review did not meet criteria *Quote: "OBJECTIVES: To compare the benefits (such as decreased mortality and improved quality of life) and harms (such as adverse events related to antibiotic use) of all antibiotic treatments in people with non-surgical wounds with established colonisation or infection caused by MRSA."* |
| Criteria for inclusion/ exclusion | **3.1. Do the review’s inclusion-exclusion criteria consider sex-gender differences?** • No, review did not meet criteria *Quote: "Any person (irrespective of age) [...]"* **3.2. Was there justification or explanation for the exclusion of some groups?** • No, review did not meet criteria |
| Methods | **4.1. Does the review examine whether outcome measures are different for males and females?** • No, review did not meet criteria **4.2. Did the review extract data by sex?** • No, review did not meet criteria **4.3. Did the review extract data on sex of withdrawals and dropouts?** • No, review did not meet criteria **4.4. In cases where sex/gender is used as a proxy for other measures (i.e., weight), is there an explanation for this approach?** • Item was not applicable to the review **4.5. Were any subgroup analyses completed?** • No, review did not meet criteria **4.6. Were subgroup analyses by sex completed?** • No, review did not meet criteria |
| Results and Analysis | **5.1. Do results distinguish between findings for males/females?** • No, review did not meet criteria **5.2. Does the review report conclusions (of effectiveness, efficacy, safety) that are different for men and women?**  • No, review did not meet criteria **5.3. If adverse effects are reported, is information sex disaggregated?** • No, review did not meet criteria **5.4. Does review note that subgroup analyses by sex could not be done?** • Item was not applicable to the review *Comment: Subgroup analysis by sex was not planned.* |
| Discussion and conclusion | **6.1. Does the review report that primary studies analysed or failed to analyse results by sex?** • No, review did not meet criteria **6.2. Does the review address sex/gender implications for clinical practice?** • No, review did not meet criteria **6.3. Does the review address sex/gender implications for policy and regulation?** • No, review did not meet criteria **6.4. Does the review address sex/gender implications for research?** • No, review did not meet criteria |
| Table of included studies | **7.1. Does the description of included studies give detailed information on sex/gender of the study samples?** • No, review did not meet criteria (At least 7.1.1. or 7.1.2. are NO) **7.1.1. Detailed information on SEX of the study samples** • Unable to determine **7.1.2. Detailed information on GENDER of the study samples** • No, review did not meet criteria |
|  |  |
| **Item** | **Gurusamy (2013)** |
| Background | **1.1 Are the terms sex and gender used in the background?** • No, review did not meet criteria **1.2 Are sex/gender identified as relevant or not to review question?** • No, review did not meet criteria **1.3. Does background discuss why sex/gender differences may be expected?** • No, review did not meet criteria |
| Objectives | **2.1. Are the terms sex, gender, male, or female used in objectives?** • No, review did not meet criteria |
| Criteria for inclusion/ exclusion | **3.1. Do the review’s inclusion-exclusion criteria consider sex-gender differences?** • No, review did not meet criteria **3.2. Was there justification or explanation for the exclusion of some groups?** • No, review did not meet criteria |
| Methods | **4.1. Does the review examine whether outcome measures are different for males and females?** • No, review did not meet criteria **4.2. Did the review extract data by sex?** • No, review did not meet criteria **4.3. Did the review extract data on sex of withdrawals and dropouts?** • No, review did not meet criteria **4.4. In cases where sex/gender is used as a proxy for other measures (i.e., weight), is there an explanation for this approach?** • Item was not applicable to the review **4.5. Were any subgroup analyses completed?** • No, review did not meet criteria **4.6. Were subgroup analyses by sex completed?** • No, review did not meet criteria |
| Results and Analysis | **5.1. Do results distinguish between findings for males/females?** • No, review did not meet criteria **5.2. Does the review report conclusions (of effectiveness, efficacy, safety) that are different for men and women?**  • No, review did not meet criteria **5.3. If adverse effects are reported, is information sex disaggregated?** • No, review did not meet criteria **5.4. Does review note that subgroup analyses by sex could not be done?** • Item was not applicable to the review |
| Discussion and conclusion | **6.1. Does the review report that primary studies analysed or failed to analyse results by sex?** • No, review did not meet criteria **6.2. Does the review address sex/gender implications for clinical practice?** • No, review did not meet criteria **6.3. Does the review address sex/gender implications for policy and regulation?** • No, review did not meet criteria **6.4. Does the review address sex/gender implications for research?** • No, review did not meet criteria |
| Table of included studies | **7.1. Does the description of included studies give detailed information on sex/gender of the study samples?** • No, review did not meet criteria (At least 7.1.1. or 7.1.2. are NO) **7.1.1. Detailed information on SEX of the study samples** • Unable to determine **7.1.2. Detailed information on GENDER of the study samples** • No, review did not meet criteria |
|  |  |
| **Item** | **Gyte (2014)** |
| Background | **1.1 Are the terms sex and gender used in the background?** • Item was not applicable to the review **1.2 Are sex/gender identified as relevant or not to review question?** • Item was not applicable to the review **1.3. Does background discuss why sex/gender differences may be expected?** • Item was not applicable to the review |
| Objectives | **2.1. Are the terms sex, gender, male, or female used in objectives?** • Item was not applicable to the review |
| Criteria for inclusion/ exclusion | **3.1. Do the review’s inclusion-exclusion criteria consider sex-gender differences?** • Item was not applicable to the review **3.2. Was there justification or explanation for the exclusion of some groups?** • Item was not applicable to the review |
| Methods | **4.1. Does the review examine whether outcome measures are different for males and females?** • Item was not applicable to the review **4.2. Did the review extract data by sex?** • Item was not applicable to the review **4.3. Did the review extract data on sex of withdrawals and dropouts?** • Item was not applicable to the review **4.4. In cases where sex/gender is used as a proxy for other measures (i.e., weight), is there an explanation for this approach?** • Item was not applicable to the review **4.5. Were any subgroup analyses completed?** • Item was not applicable to the review **4.6. Were subgroup analyses by sex completed?** • Item was not applicable to the review |
| Results and Analysis | **5.1. Do results distinguish between findings for males/females?** • Item was not applicable to the review **5.2. Does the review report conclusions (of effectiveness, efficacy, safety) that are different for men and women?**  • Item was not applicable to the review **5.3. If adverse effects are reported, is information sex disaggregated?** • Item was not applicable to the review **5.4. Does review note that subgroup analyses by sex could not be done?** • Item was not applicable to the review |
| Discussion and conclusion | **6.1. Does the review report that primary studies analysed or failed to analyse results by sex?** • Item was not applicable to the review **6.2. Does the review address sex/gender implications for clinical practice?** • Item was not applicable to the review **6.3. Does the review address sex/gender implications for policy and regulation?** • Item was not applicable to the review **6.4. Does the review address sex/gender implications for research?** • Item was not applicable to the review |
| Table of included studies | **7.1. Does the description of included studies give detailed information on sex/gender of the study samples?** • Item was not applicable to the review **7.1.1. Detailed information on SEX of the study samples** • Item was not applicable to the review **7.1.2. Detailed information on GENDER of the study samples** • Item was not applicable to the review |
|  |  |
| **Item** | **Haas (2014)** |
| Background | **1.1 Are the terms sex and gender used in the background?** • Item was not applicable to the review **1.2 Are sex/gender identified as relevant or not to review question?** • Item was not applicable to the review **1.3. Does background discuss why sex/gender differences may be expected?** • Item was not applicable to the review |
| Objectives | **2.1. Are the terms sex, gender, male, or female used in objectives?** • Item was not applicable to the review |
| Criteria for inclusion/ exclusion | **3.1. Do the review’s inclusion-exclusion criteria consider sex-gender differences?** • Item was not applicable to the review **3.2. Was there justification or explanation for the exclusion of some groups?** • Item was not applicable to the review |
| Methods | **4.1. Does the review examine whether outcome measures are different for males and females?** • Item was not applicable to the review **4.2. Did the review extract data by sex?** • Item was not applicable to the review **4.3. Did the review extract data on sex of withdrawals and dropouts?** • Item was not applicable to the review **4.4. In cases where sex/gender is used as a proxy for other measures (i.e., weight), is there an explanation for this approach?** • Item was not applicable to the review **4.5. Were any subgroup analyses completed?** • Item was not applicable to the review **4.6. Were subgroup analyses by sex completed?** • Item was not applicable to the review |
| Results and Analysis | **5.1. Do results distinguish between findings for males/females?** • Item was not applicable to the review **5.2. Does the review report conclusions (of effectiveness, efficacy, safety) that are different for men and women?**  • Item was not applicable to the review **5.3. If adverse effects are reported, is information sex disaggregated?** • Item was not applicable to the review **5.4. Does review note that subgroup analyses by sex could not be done?** • Item was not applicable to the review |
| Discussion and conclusion | **6.1. Does the review report that primary studies analysed or failed to analyse results by sex?** • Item was not applicable to the review **6.2. Does the review address sex/gender implications for clinical practice?** • Item was not applicable to the review **6.3. Does the review address sex/gender implications for policy and regulation?** • Item was not applicable to the review **6.4. Does the review address sex/gender implications for research?** • Item was not applicable to the review |
| Table of included studies | **7.1. Does the description of included studies give detailed information on sex/gender of the study samples?** • Item was not applicable to the review **7.1.1. Detailed information on SEX of the study samples** • Item was not applicable to the review **7.1.2. Detailed information on GENDER of the study samples** • Item was not applicable to the review |
|  |  |
| **Item** | **Hadiati (2014)** |
| Background | **1.1 Are the terms sex and gender used in the background?** • Item was not applicable to the review **1.2 Are sex/gender identified as relevant or not to review question?** • Item was not applicable to the review **1.3. Does background discuss why sex/gender differences may be expected?** • Item was not applicable to the review |
| Objectives | **2.1. Are the terms sex, gender, male, or female used in objectives?** • Item was not applicable to the review |
| Criteria for inclusion/ exclusion | **3.1. Do the review’s inclusion-exclusion criteria consider sex-gender differences?** • Item was not applicable to the review **3.2. Was there justification or explanation for the exclusion of some groups?** • Item was not applicable to the review |
| Methods | **4.1. Does the review examine whether outcome measures are different for males and females?** • Item was not applicable to the review **4.2. Did the review extract data by sex?** • Item was not applicable to the review **4.3. Did the review extract data on sex of withdrawals and dropouts?** • Item was not applicable to the review **4.4. In cases where sex/gender is used as a proxy for other measures (i.e., weight), is there an explanation for this approach?** • Item was not applicable to the review **4.5. Were any subgroup analyses completed?** • Item was not applicable to the review **4.6. Were subgroup analyses by sex completed?** • Item was not applicable to the review |
| Results and Analysis | **5.1. Do results distinguish between findings for males/females?** • Item was not applicable to the review **5.2. Does the review report conclusions (of effectiveness, efficacy, safety) that are different for men and women?**  • Item was not applicable to the review **5.3. If adverse effects are reported, is information sex disaggregated?** • Item was not applicable to the review **5.4. Does review note that subgroup analyses by sex could not be done?** • Item was not applicable to the review |
| Discussion and conclusion | **6.1. Does the review report that primary studies analysed or failed to analyse results by sex?** • Item was not applicable to the review **6.2. Does the review address sex/gender implications for clinical practice?** • Item was not applicable to the review **6.3. Does the review address sex/gender implications for policy and regulation?** • Item was not applicable to the review **6.4. Does the review address sex/gender implications for research?** • Item was not applicable to the review |
| Table of included studies | **7.1. Does the description of included studies give detailed information on sex/gender of the study samples?** • Item was not applicable to the review **7.1.1. Detailed information on SEX of the study samples** • Item was not applicable to the review **7.1.2. Detailed information on GENDER of the study samples** • Item was not applicable to the review |
|  |  |
| **Item** | **Heal (2016)** |
| Background | **1.1 Are the terms sex and gender used in the background?** • No, review did not meet criteria **1.2 Are sex/gender identified as relevant or not to review question?** • No, review did not meet criteria **1.3. Does background discuss why sex/gender differences may be expected?** • No, review did not meet criteria |
| Objectives | **2.1. Are the terms sex, gender, male, or female used in objectives?** • No, review did not meet criteria *Quote: "The primary objective of this review was to determine whether the application of topical antibiotics to surgical wounds that are healing by primary intention reduces the incidence of SSI and whether it increases the incidence of adverse outcomes (allergic contact dermatitis, infections with patterns of antibiotic resistance and anaphylaxis)."* |
| Criteria for inclusion/ exclusion | **3.1. Do the review’s inclusion-exclusion criteria consider sex-gender differences?** • No, review did not meet criteria *Quote: "Types of participants We included: • people of any age, gender or country of origin who had undergone surgical procedures where healing of the surgical wound was planned by primary intention, i.e. where wounds had edges approximated with sutures, staples, clips or glue; [...]"* **3.2. Was there justification or explanation for the exclusion of some groups?** • No, review did not meet criteria |
| Methods | **4.1. Does the review examine whether outcome measures are different for males and females?** • No, review did not meet criteria **4.2. Did the review extract data by sex?** • No, review did not meet criteria **4.3. Did the review extract data on sex of withdrawals and dropouts?** • No, review did not meet criteria *Quote: "Subgroup analysis and investigation of heterogeneity Where there were sufficient trials of adequate size and it was possible to conduct subgroup analyses, we planned to conduct subgroup analyses for: • clean versus clean contaminated versus contaminated wounds; • dermatological versus general surgery; • class of antibiotic used; • single application versus multiple applications; and • no treatment control versus placebo ointment control".* **4.4. In cases where sex/gender is used as a proxy for other measures (i.e., weight), is there an explanation for this approach?** • Item was not applicable to the review **4.5. Were any subgroup analyses completed?** • No, review did not meet criteria **4.6. Were subgroup analyses by sex completed?** • No, review did not meet criteria |
| Results and Analysis | **5.1. Do results distinguish between findings for males/females?** • No, review did not meet criteria **5.2. Does the review report conclusions (of effectiveness, efficacy, safety) that are different for men and women?**  • No, review did not meet criteria **5.3. If adverse effects are reported, is information sex disaggregated?** • No, review did not meet criteria **5.4. Does review note that subgroup analyses by sex could not be done?** • Item was not applicable to the review |
| Discussion and conclusion | **6.1. Does the review report that primary studies analysed or failed to analyse results by sex?** • No, review did not meet criteria **6.2. Does the review address sex/gender implications for clinical practice?** • No, review did not meet criteria **6.3. Does the review address sex/gender implications for policy and regulation?** • No, review did not meet criteria **6.4. Does the review address sex/gender implications for research?** • No, review did not meet criteria |
| Table of included studies | **7.1. Does the description of included studies give detailed information on sex/gender of the study samples?** • No, review did not meet criteria (At least 7.1.1. or 7.1.2. are NO) **7.1.1. Detailed information on SEX of the study samples** • No, review did not meet criteria **7.1.2. Detailed information on GENDER of the study samples** • No, review did not meet criteria |
|  |  |
| **Item** | **Hsu (2016)** |
| Background | **1.1 Are the terms sex and gender used in the background?** • No, review did not meet criteria **1.2 Are sex/gender identified as relevant or not to review question?** • No, review did not meet criteria **1.3. Does background discuss why sex/gender differences may be expected?** • No, review did not meet criteria |
| Objectives | **2.1. Are the terms sex, gender, male, or female used in objectives?** • No, review did not meet criteria *Quote: "To compare the efficacy and safety of three commonly used techniques for implanting totally implantable venous access ports (TIVAPs): the venous cut down technique, the Seldinger technique, and the modified Seldinger technique. The review includes studies that use Doppler or real-time two-dimensional ultrasonography for locating the vein in the Seldinger technique."* |
| Criteria for inclusion/ exclusion | **3.1. Do the review’s inclusion-exclusion criteria consider sex-gender differences?** • No, review did not meet criteria **3.2. Was there justification or explanation for the exclusion of some groups?** • No, review did not meet criteria |
| Methods | **4.1. Does the review examine whether outcome measures are different for males and females?** • No, review did not meet criteria **4.2. Did the review extract data by sex?** • No, review did not meet criteria **4.3. Did the review extract data on sex of withdrawals and dropouts?** • No, review did not meet criteria **4.4. In cases where sex/gender is used as a proxy for other measures (i.e., weight), is there an explanation for this approach?** • Item was not applicable to the review **4.5. Were any subgroup analyses completed?** • No, review did not meet criteria **4.6. Were subgroup analyses by sex completed?** • No, review did not meet criteria |
| Results and Analysis | **5.1. Do results distinguish between findings for males/females?** • No, review did not meet criteria **5.2. Does the review report conclusions (of effectiveness, efficacy, safety) that are different for men and women?**  • No, review did not meet criteria **5.3. If adverse effects are reported, is information sex disaggregated?** • No, review did not meet criteria **5.4. Does review note that subgroup analyses by sex could not be done?** • Item was not applicable to the review |
| Discussion and conclusion | **6.1. Does the review report that primary studies analysed or failed to analyse results by sex?** • No, review did not meet criteria **6.2. Does the review address sex/gender implications for clinical practice?** • No, review did not meet criteria **6.3. Does the review address sex/gender implications for policy and regulation?** • No, review did not meet criteria **6.4. Does the review address sex/gender implications for research?** • No, review did not meet criteria |
| Table of included studies | **7.1. Does the description of included studies give detailed information on sex/gender of the study samples?** • No, review did not meet criteria (At least 7.1.1. or 7.1.2. are NO) **7.1.1. Detailed information on SEX of the study samples** • No, review did not meet criteria **7.1.2. Detailed information on GENDER of the study samples** • Unable to determine |
|  |  |
| **Item** | **Hua (2016)** |
| Background | **1.1 Are the terms sex and gender used in the background?** • No, review did not meet criteria **1.2 Are sex/gender identified as relevant or not to review question?** • No, review did not meet criteria **1.3. Does background discuss why sex/gender differences may be expected?** • No, review did not meet criteria |
| Objectives | **2.1. Are the terms sex, gender, male, or female used in objectives?** • No, review did not meet criteria *Quote: "To assess the effects of oral hygiene care on incidence of ventilator associated pneumonia in critically ill patients receiving mechanical ventilation in hospital intensive care units (ICUs)."* |
| Criteria for inclusion/ exclusion | **3.1. Do the review’s inclusion-exclusion criteria consider sex-gender differences?** • No, review did not meet criteria *Quote: "Critically ill patients in hospital settings receiving mechanical ventilation for a minimum of 48 hours, without ventilator-associated pneumonia or respiratory infection at baseline. We included trials where only some of the participants were receiving mechanical ventilation if the outcome of ventilator-associated pneumonia was reported, and data were available for those who had been treated with mechanical ventilation for a minimum of 48 hours and then developed nosocomial pneumonia. We included trials where participants were undergoing a surgical procedure that involved mechanical ventilation (e.g. cardiac surgery) only if the oral hygiene care was given during the period of mechanical ventilation that had a minimum duration of 48 hours. We excluded trials where patients received a single preoperative dose of antibacterial rinse or gargle, and received mechanical ventilation only for the duration of the surgery, with no further mechanical ventilation and oral hygiene care during the postoperative period."* **3.2. Was there justification or explanation for the exclusion of some groups?** • No, review did not meet criteria |
| Methods | **4.1. Does the review examine whether outcome measures are different for males and females?** • No, review did not meet criteria **4.2. Did the review extract data by sex?** • No, review did not meet criteria **4.3. Did the review extract data on sex of withdrawals and dropouts?** • No, review did not meet criteria **4.4. In cases where sex/gender is used as a proxy for other measures (i.e., weight), is there an explanation for this approach?** • Item was not applicable to the review **4.5. Were any subgroup analyses completed?** • Yes, review met criteria *Quote: "We proposed one subgroup analysis a priori. We decided to undertake a subgroup analysis according to whether participants’ teeth were cleaned or not, as we hypothesised that antiseptics would be less effective if tooth brushing was not used to disrupt dental plaque biofilm." Comment: See, for example, "The meta-analysis showed a reduction in VAP in the chlorhexidine group (RR 0.71, 95% CI 0.53 to 0.94, P = 0.02, I2 = 28%) (Analysis 1.1, Subgroup 1.1.1)."* **4.6. Were subgroup analyses by sex completed?** • No, review did not meet criteria |
| Results and Analysis | **5.1. Do results distinguish between findings for males/females?** • No, review did not meet criteria **5.2. Does the review report conclusions (of effectiveness, efficacy, safety) that are different for men and women?**  • No, review did not meet criteria **5.3. If adverse effects are reported, is information sex disaggregated?** • No, review did not meet criteria **5.4. Does review note that subgroup analyses by sex could not be done?** • Item was not applicable to the review |
| Discussion and conclusion | **6.1. Does the review report that primary studies analysed or failed to analyse results by sex?** • No, review did not meet criteria **6.2. Does the review address sex/gender implications for clinical practice?** • No, review did not meet criteria **6.3. Does the review address sex/gender implications for policy and regulation?** • No, review did not meet criteria **6.4. Does the review address sex/gender implications for research?** • No, review did not meet criteria |
| Table of included studies | **7.1. Does the description of included studies give detailed information on sex/gender of the study samples?** • No, review did not meet criteria (At least 7.1.1. or 7.1.2. are NO) **7.1.1. Detailed information on SEX of the study samples** • No, review did not meet criteria **7.1.2. Detailed information on GENDER of the study samples** • No, review did not meet criteria |
|  |  |
| **Item** | **Hughes (2013)** |
| Background | **1.1 Are the terms sex and gender used in the background?** • Yes, review met criteria *Quote: "In the period 2008-12, MRSA death rates increased with age and were higher for males than for females (Health Stats 2013)".* **1.2 Are sex/gender identified as relevant or not to review question?** • Unable to determine **1.3. Does background discuss why sex/gender differences may be expected?** • No, review did not meet criteria *Quote: "MRSA death rates increased with age and were higher for males than for females (Health Stats 2013). Comment: The review provides data about sex/gender differences but it does not explain why.* |
| Objectives | **2.1. Are the terms sex, gender, male, or female used in objectives?** • No, review did not meet criteria *Quote: "OBJECTIVES: The objective of this review is to determine the effects of infection control strategies for preventing the transmission of MRSA in nursing homes for older people.”* |
| Criteria for inclusion/ exclusion | **3.1. Do the review’s inclusion-exclusion criteria consider sex-gender differences?** • No, review did not meet criteria *Quote: "Types of participants 1. Residents over the age of 65 years, living in nursing homes: this age was chosen as it is the conventional cut-off point for the categorisation of those considered to be ’older’. We accepted trials for inclusion if the majority of participants were over the age of 65 years or the mean age was more than 65 years. 2. Staff of nursing homes: we included all staff that work in nursing homes and may be a potential source of transmission of MRSA. 3. Nursing homes: in the UK context, nursing homes were defined as facilities in which qualified nursing care is available 24 hours a day. This excluded residential homes where qualified nursing care is not provided. In the US context, nursing homes may be termed skilled nursing facilities or long-term care facilities. Other descriptions such as aged-care facilities were considered if they appeared to meet the definitions used in the UK or USA. If there is any ambiguity with the description of the institution, clarification will be sought from the authors of the relevant papers. Including these three types of participants will allow us to include studies that have considered interventions at the level of the residents, staff and at nursing home level".* **3.2. Was there justification or explanation for the exclusion of some groups?** • No, review did not meet criteria |
| Methods | **4.1. Does the review examine whether outcome measures are different for males and females?** • No, review did not meet criteria **4.2. Did the review extract data by sex?** • No, review did not meet criteria **4.3. Did the review extract data on sex of withdrawals and dropouts?** • No, review did not meet criteria **4.4. In cases where sex/gender is used as a proxy for other measures (i.e., weight), is there an explanation for this approach?** • Item was not applicable to the review **4.5. Were any subgroup analyses completed?** • No, review did not meet criteria **4.6. Were subgroup analyses by sex completed?** • No, review did not meet criteria |
| Results and Analysis | **5.1. Do results distinguish between findings for males/females?** • No, review did not meet criteria **5.2. Does the review report conclusions (of effectiveness, efficacy, safety) that are different for men and women?**  • No, review did not meet criteria **5.3. If adverse effects are reported, is information sex disaggregated?** • Item was not applicable to the review *Comment: adverse effects not planned to be measured in the review.*  **5.4. Does review note that subgroup analyses by sex could not be done?** • Item was not applicable to the review *Comment: Subgroup analysis by sex was not planned.* |
| Discussion and conclusion | **6.1. Does the review report that primary studies analysed or failed to analyse results by sex?** • No, review did not meet criteria **6.2. Does the review address sex/gender implications for clinical practice?** • No, review did not meet criteria **6.3. Does the review address sex/gender implications for policy and regulation?** • No, review did not meet criteria **6.4. Does the review address sex/gender implications for research?** • No, review did not meet criteria |
| Table of included studies | **7.1. Does the description of included studies give detailed information on sex/gender of the study samples?** • No, review did not meet criteria (At least 7.1.1. or 7.1.2. are NO) **7.1.1. Detailed information on SEX of the study samples** • No, review did not meet criteria **7.1.2. Detailed information on GENDER of the study samples** • No, review did not meet criteria |
|  |  |
| **Item** | **Ireland (2014)** |
| Background | **1.1 Are the terms sex and gender used in the background?** • No, review did not meet criteria **1.2 Are sex/gender identified as relevant or not to review question?** • No, review did not meet criteria **1.3. Does background discuss why sex/gender differences may be expected?** • No, review did not meet criteria |
| Objectives | **2.1. Are the terms sex, gender, male, or female used in objectives?** • No, review did not meet criteria *Quote: "To determine whether any difference can be found in the rate of mortality and adverse events following major abdominal surgery in patients treated postoperatively with CPAP versus standard care, which may include traditional oxygen delivery systems, physiotherapy and incentive spirometry."* |
| Criteria for inclusion/ exclusion | **3.1. Do the review’s inclusion-exclusion criteria consider sex-gender differences?** • No, review did not meet criteria *Quote: "We included all adults (adults as defined by individual studies) of both sexes who underwent elective or emergency major abdominal surgery. We did not exclude patients with co-morbidities such as obesity, respiratory disease and a history of smoking. We excluded patients who received bilevel positive airway pressure (BiPAP) and those treated with CPAP perioperatively, because the review was confined to postoperative use of CPAP."* **3.2. Was there justification or explanation for the exclusion of some groups?** • No, review did not meet criteria |
| Methods | **4.1. Does the review examine whether outcome measures are different for males and females?** • No, review did not meet criteria **4.2. Did the review extract data by sex?** • No, review did not meet criteria **4.3. Did the review extract data on sex of withdrawals and dropouts?** • No, review did not meet criteria **4.4. In cases where sex/gender is used as a proxy for other measures (i.e., weight), is there an explanation for this approach?** • Item was not applicable to the review **4.5. Were any subgroup analyses completed?** • No, review did not meet criteria **4.6. Were subgroup analyses by sex completed?** • No, review did not meet criteria |
| Results and Analysis | **5.1. Do results distinguish between findings for males/females?** • No, review did not meet criteria **5.2. Does the review report conclusions (of effectiveness, efficacy, safety) that are different for men and women?**  • No, review did not meet criteria **5.3. If adverse effects are reported, is information sex disaggregated?** • No, review did not meet criteria **5.4. Does review note that subgroup analyses by sex could not be done?** • Item was not applicable to the review |
| Discussion and conclusion | **6.1. Does the review report that primary studies analysed or failed to analyse results by sex?** • No, review did not meet criteria **6.2. Does the review address sex/gender implications for clinical practice?** • No, review did not meet criteria **6.3. Does the review address sex/gender implications for policy and regulation?** • No, review did not meet criteria **6.4. Does the review address sex/gender implications for research?** • No, review did not meet criteria |
| Table of included studies | **7.1. Does the description of included studies give detailed information on sex/gender of the study samples?** • No, review did not meet criteria (At least 7.1.1. or 7.1.2. are NO) **7.1.1. Detailed information on SEX of the study samples** • Unable to determine **7.1.2. Detailed information on GENDER of the study samples** • No, review did not meet criteria |
|  |  |
| **Item** | **Jamison (2013)** |
| Background | **1.1 Are the terms sex and gender used in the background?** • Yes, review met criteria *Quote: "Neurogenic bladder comprises any dysfunctional condition of the urinary bladder caused by a spinal cord injury (SCI) or other lesion or disease of the central nervous system (CNS). [..] SCI primarily affects young males, whereas young women are more likely to develop multiple sclerosis (female: male sex ratio 1: 4 and 2.5: 1, respectively)." Quote: "Management options include intermittent urethral catheterisation, indwelling urethral or suprapubic catheterisation, timed voiding, use of an external catheter (for men), augmentation cystoplasty and urinary diversion".*  **1.2 Are sex/gender identified as relevant or not to review question?** • No, review did not meet criteria *Quote: "Neurogenic bladder comprises any dysfunctional condition of the urinary bladder caused by a spinal cord injury (SCI) or other lesion or disease of the central nervous system (CNS). [..] SCI primarily affects young males, whereas young women are more likely to develop multiple sclerosis (female: male sex ratio 1: 4 and 2.5: 1, respectively)." Quote: "Management options include intermittent urethral catheterisation, indwelling urethral or suprapubic catheterisation, timed voiding, use of an external catheter (for men), augmentation cystoplasty and urinary diversion" Comment: there is no explicit mention to the relevance for men or women.*  **1.3. Does background discuss why sex/gender differences may be expected?** • No, review did not meet criteria *Quote: "Neurogenic bladder comprises any dysfunctional condition of the urinary bladder caused by a spinal cord injury (SCI) or other lesion or disease of the central nervous system (CNS). [..] SCI primarily affects young males, whereas young women are more likely to develop multiple sclerosis (female: male sex ratio 1: 4 and 2.5: 1, respectively)." Quote: "Management options include intermittent urethral catheterisation, indwelling urethral or suprapubic catheterisation, timed voiding, use of an external catheter (for men), augmentation cystoplasty and urinary diversion".* |
| Objectives | **2.1. Are the terms sex, gender, male, or female used in objectives?** • No, review did not meet criteria |
| Criteria for inclusion/ exclusion | **3.1. Do the review’s inclusion-exclusion criteria consider sex-gender differences?** • No, review did not meet criteria *Quote: "Adults in hospitals, nursing homes and the community with neurogenic bladder persisting after three months for whom catheters are an option for long-term management, whether or not they have had surgery or another invasive procedure."* **3.2. Was there justification or explanation for the exclusion of some groups?** • No, review did not meet criteria |
| Methods | **4.1. Does the review examine whether outcome measures are different for males and females?** • No, review did not meet criteria **4.2. Did the review extract data by sex?** • No, review did not meet criteria **4.3. Did the review extract data on sex of withdrawals and dropouts?** • No, review did not meet criteria *Comment: The review does not provide any information on this.*  **4.4. In cases where sex/gender is used as a proxy for other measures (i.e., weight), is there an explanation for this approach?** • Item was not applicable to the review **4.5. Were any subgroup analyses completed?** • No, review did not meet criteria **4.6. Were subgroup analyses by sex completed?** • No, review did not meet criteria |
| Results and Analysis | **5.1. Do results distinguish between findings for males/females?** • Item was not applicable to the review **5.2. Does the review report conclusions (of effectiveness, efficacy, safety) that are different for men and women?**  • Item was not applicable to the review **5.3. If adverse effects are reported, is information sex disaggregated?** • Item was not applicable to the review **5.4. Does review note that subgroup analyses by sex could not be done?** • Yes, review met criteria |
| Discussion and conclusion | **6.1. Does the review report that primary studies analysed or failed to analyse results by sex?** • Item was not applicable to the review **6.2. Does the review address sex/gender implications for clinical practice?** • No, review did not meet criteria **6.3. Does the review address sex/gender implications for policy and regulation?** • No, review did not meet criteria **6.4. Does the review address sex/gender implications for research?** • No, review did not meet criteria |
| Table of included studies | **7.1. Does the description of included studies give detailed information on sex/gender of the study samples?** • Item was not applicable to the review **7.1.1. Detailed information on SEX of the study samples** • Item was not applicable to the review **7.1.2. Detailed information on GENDER of the study samples** • Item was not applicable to the review |
|  |  |
| **Item** | **Jefferson (2011)** |
| Background | **1.1 Are the terms sex and gender used in the background?** • No, review did not meet criteria **1.2 Are sex/gender identified as relevant or not to review question?** • No, review did not meet criteria **1.3. Does background discuss why sex/gender differences may be expected?** • No, review did not meet criteria |
| Objectives | **2.1. Are the terms sex, gender, male, or female used in objectives?** • No, review did not meet criteria *Quote: "To systematically review the evidence of effectiveness of physical interventions to interrupt or reduce the spread of acute respiratory viruses."* |
| Criteria for inclusion/ exclusion | **3.1. Do the review’s inclusion-exclusion criteria consider sex-gender differences?** • No, review did not meet criteria **3.2. Was there justification or explanation for the exclusion of some groups?** • No, review did not meet criteria |
| Methods | **4.1. Does the review examine whether outcome measures are different for males and females?** • No, review did not meet criteria **4.2. Did the review extract data by sex?** • No, review did not meet criteria **4.3. Did the review extract data on sex of withdrawals and dropouts?** • No, review did not meet criteria **4.4. In cases where sex/gender is used as a proxy for other measures (i.e., weight), is there an explanation for this approach?** • Item was not applicable to the review **4.5. Were any subgroup analyses completed?** • Yes, review met criteria *Quote: "Subgroup analysis and investigation of heterogeneity: An a priori subgroup analysis was planned for: 1. pandemic influenza outbreaks; 2. seasonal influenza; and 3. other epidemics (for example, SARS). We had sufficient data to carry out only the last."* **4.6. Were subgroup analyses by sex completed?** • No, review did not meet criteria |
| Results and Analysis | **5.1. Do results distinguish between findings for males/females?** • No, review did not meet criteria **5.2. Does the review report conclusions (of effectiveness, efficacy, safety) that are different for men and women?**  • No, review did not meet criteria **5.3. If adverse effects are reported, is information sex disaggregated?** • Item was not applicable to the review **5.4. Does review note that subgroup analyses by sex could not be done?** • Item was not applicable to the review |
| Discussion and conclusion | **6.1. Does the review report that primary studies analysed or failed to analyse results by sex?** • No, review did not meet criteria **6.2. Does the review address sex/gender implications for clinical practice?** • No, review did not meet criteria **6.3. Does the review address sex/gender implications for policy and regulation?** • No, review did not meet criteria **6.4. Does the review address sex/gender implications for research?** • No, review did not meet criteria |
| Table of included studies | **7.1. Does the description of included studies give detailed information on sex/gender of the study samples?** • No, review did not meet criteria (At least 7.1.1. or 7.1.2. are NO) **7.1.1. Detailed information on SEX of the study samples** • No, review did not meet criteria **7.1.2. Detailed information on GENDER of the study samples** • No, review did not meet criteria |
|  |  |
| **Item** | **Jones (2014)** |
| Background | **1.1 Are the terms sex and gender used in the background?** • Yes, review met criteria *Quote: "Breast cancer accounts for one in 10 of all new cancer cases diagnosed around the world each year (Bray 2004) and is the leading cause of cancer death in women (Pisani 1999)." Quote: "Whilst the risk of breast cancer for men is only 1%, treatment for men is very similar to that of women (Harris 2004)." Quote: "Some women have immediate breast reconstruction; however this group of patients has a higher risk of SSI (Spauwen 2000)." Quote: "A recent review (Pittet 2005) found that women who had been treated for breast cancer and who had immediate reconstruction had a SSI rate of between 0% and 53%, whilst non-cancer patients undergoing the same reconstructive surgery had an average rate of 2.5%."* **1.2 Are sex/gender identified as relevant or not to review question?** • Unable to determine **1.3. Does background discuss why sex/gender differences may be expected?** • No, review did not meet criteria *Quote: "Breast cancer accounts for one in 10 of all new cancer cases diagnosed around the world each year (Bray 2004) and is the leading cause of cancer death in women (Pisani 1999)." Quote: "Whilst the risk of breast cancer for men is only 1%, treatment for men is very similar to that of women (Harris 2004)." Quote: "Some women have immediate breast reconstruction; however this group of patients has a higher risk of SSI (Spauwen 2000)."* |
| Objectives | **2.1. Are the terms sex, gender, male, or female used in objectives?** • No, review did not meet criteria *Quote: "To determine the effects of prophylactic antibiotics on SSI after breast cancer surgery."* |
| Criteria for inclusion/ exclusion | **3.1. Do the review’s inclusion-exclusion criteria consider sex-gender differences?** • No, review did not meet criteria *Quote: "People with breast cancer undergoing breast surgery with or without immediate re-construction as part of their treatment. We included studies that involved mixed patient groups (i.e. cancer and non-cancer, other surgeries or breast implants not as part of cancer treatment) as long as it was possible to extract separate data for those undergoing surgery primarily to treat breast cancer."* **3.2. Was there justification or explanation for the exclusion of some groups?** • No, review did not meet criteria |
| Methods | **4.1. Does the review examine whether outcome measures are different for males and females?** • No, review did not meet criteria **4.2. Did the review extract data by sex?** • No, review did not meet criteria **4.3. Did the review extract data on sex of withdrawals and dropouts?** • No, review did not meet criteria **4.4. In cases where sex/gender is used as a proxy for other measures (i.e., weight), is there an explanation for this approach?** • Item was not applicable to the review **4.5. Were any subgroup analyses completed?** • Yes, review met criteria **4.6. Were subgroup analyses by sex completed?** • No, review did not meet criteria |
| Results and Analysis | **5.1. Do results distinguish between findings for males/females?** • No, review did not meet criteria **5.2. Does the review report conclusions (of effectiveness, efficacy, safety) that are different for men and women?**  • No, review did not meet criteria **5.3. If adverse effects are reported, is information sex disaggregated?** • No, review did not meet criteria **5.4. Does review note that subgroup analyses by sex could not be done?** • Item was not applicable to the review |
| Discussion and conclusion | **6.1. Does the review report that primary studies analysed or failed to analyse results by sex?** • No, review did not meet criteria **6.2. Does the review address sex/gender implications for clinical practice?** • No, review did not meet criteria **6.3. Does the review address sex/gender implications for policy and regulation?** • No, review did not meet criteria **6.4. Does the review address sex/gender implications for research?** • No, review did not meet criteria |
| Table of included studies | **7.1. Does the description of included studies give detailed information on sex/gender of the study samples?** • No, review did not meet criteria (At least 7.1.1. or 7.1.2. are NO) **7.1.1. Detailed information on SEX of the study samples** • No, review did not meet criteria **7.1.2. Detailed information on GENDER of the study samples** • No, review did not meet criteria |
|  |  |
| **Item** | **Kao (2009)** |
| Background | **1.1 Are the terms sex and gender used in the background?** • No, review did not meet criteria **1.2 Are sex/gender identified as relevant or not to review question?** • No, review did not meet criteria **1.3. Does background discuss why sex/gender differences may be expected?** • No, review did not meet criteria |
| Objectives | **2.1. Are the terms sex, gender, male, or female used in objectives?** • No, review did not meet criteria *Quote: "To summarize the evidence for the impact of strict glycaemic control in the peri-operative period on the incidence of surgical site infections, hypoglycaemia, level of glycaemic control, all-cause and infection-related mortality, and length of hospital stay and to investigate for differences of effect between different levels of glycaemic control."* |
| Criteria for inclusion/ exclusion | **3.1. Do the review’s inclusion-exclusion criteria consider sex-gender differences?** • No, review did not meet criteria **3.2. Was there justification or explanation for the exclusion of some groups?** • No, review did not meet criteria |
| Methods | **4.1. Does the review examine whether outcome measures are different for males and females?** • No, review did not meet criteria **4.2. Did the review extract data by sex?** • No, review did not meet criteria **4.3. Did the review extract data on sex of withdrawals and dropouts?** • No, review did not meet criteria **4.4. In cases where sex/gender is used as a proxy for other measures (i.e., weight), is there an explanation for this approach?** • Item was not applicable to the review **4.5. Were any subgroup analyses completed?** • Yes, review met criteria *Quote: "Subgroup analyses of people with and without diabetes yielded similar results".* **4.6. Were subgroup analyses by sex completed?** • No, review did not meet criteria |
| Results and Analysis | **5.1. Do results distinguish between findings for males/females?** • No, review did not meet criteria **5.2. Does the review report conclusions (of effectiveness, efficacy, safety) that are different for men and women?**  • No, review did not meet criteria **5.3. If adverse effects are reported, is information sex disaggregated?** • No, review did not meet criteria **5.4. Does review note that subgroup analyses by sex could not be done?** • Item was not applicable to the review |
| Discussion and conclusion | **6.1. Does the review report that primary studies analysed or failed to analyse results by sex?** • No, review did not meet criteria **6.2. Does the review address sex/gender implications for clinical practice?** • No, review did not meet criteria **6.3. Does the review address sex/gender implications for policy and regulation?** • No, review did not meet criteria **6.4. Does the review address sex/gender implications for research?** • No, review did not meet criteria |
| Table of included studies | **7.1. Does the description of included studies give detailed information on sex/gender of the study samples?** • No, review did not meet criteria (At least 7.1.1. or 7.1.2. are NO) **7.1.1. Detailed information on SEX of the study samples** • No, review did not meet criteria **7.1.2. Detailed information on GENDER of the study samples** • No, review did not meet criteria |
|  |  |
| **Item** | **Kelly (2010)** |
| Background | **1.1 Are the terms sex and gender used in the background?** • No, review did not meet criteria **1.2 Are sex/gender identified as relevant or not to review question?** • No, review did not meet criteria **1.3. Does background discuss why sex/gender differences may be expected?** • No, review did not meet criteria |
| Objectives | **2.1. Are the terms sex, gender, male, or female used in objectives?** • No, review did not meet criteria *Quote: "The objective of this review was to determine whether heated humidification (HH) or heat and moisture exchangers (HME) is more effective in preventing complications in mechanically ventilated people. Subgroup analyses were proposed based on the age of patients, the period of ventilation, and patients with an endotracheal tube compared to patients with a tracheostomy."* |
| Criteria for inclusion/ exclusion | **3.1. Do the review’s inclusion-exclusion criteria consider sex-gender differences?** • No, review did not meet criteria **3.2. Was there justification or explanation for the exclusion of some groups?** • No, review did not meet criteria |
| Methods | **4.1. Does the review examine whether outcome measures are different for males and females?** • No, review did not meet criteria **4.2. Did the review extract data by sex?** • No, review did not meet criteria **4.3. Did the review extract data on sex of withdrawals and dropouts?** • No, review did not meet criteria **4.4. In cases where sex/gender is used as a proxy for other measures (i.e., weight), is there an explanation for this approach?** • Item was not applicable to the review **4.5. Were any subgroup analyses completed?** • Yes, review met criteria **4.6. Were subgroup analyses by sex completed?** • No, review did not meet criteria |
| Results and Analysis | **5.1. Do results distinguish between findings for males/females?** • No, review did not meet criteria **5.2. Does the review report conclusions (of effectiveness, efficacy, safety) that are different for men and women?**  • No, review did not meet criteria **5.3. If adverse effects are reported, is information sex disaggregated?** • No, review did not meet criteria **5.4. Does review note that subgroup analyses by sex could not be done?** • Item was not applicable to the review |
| Discussion and conclusion | **6.1. Does the review report that primary studies analysed or failed to analyse results by sex?** • No, review did not meet criteria **6.2. Does the review address sex/gender implications for clinical practice?** • No, review did not meet criteria **6.3. Does the review address sex/gender implications for policy and regulation?** • No, review did not meet criteria **6.4. Does the review address sex/gender implications for research?** • No, review did not meet criteria |
| Table of included studies | **7.1. Does the description of included studies give detailed information on sex/gender of the study samples?** • No, review did not meet criteria (At least 7.1.1. or 7.1.2. are NO) **7.1.1. Detailed information on SEX of the study samples** • No, review did not meet criteria **7.1.2. Detailed information on GENDER of the study samples** • No, review did not meet criteria |
|  |  |
| **Item** | **Kidd (2015)** |
| Background | **1.1 Are the terms sex and gender used in the background?** • No, review did not meet criteria **1.2 Are sex/gender identified as relevant or not to review question?** • No, review did not meet criteria **1.3. Does background discuss why sex/gender differences may be expected?** • No, review did not meet criteria |
| Objectives | **2.1. Are the terms sex, gender, male, or female used in objectives?** • No, review did not meet criteria *Quote: "To determine the advantages and disadvantages of alternative routes of short-term bladder catheterisation in adults in terms of infection, adverse events, replacement, duration of use, participant satisfaction and cost effectiveness. For the purpose of this review, we define ’short-term’ as intended duration of catheterisation for 14 days or less."* |
| Criteria for inclusion/ exclusion | **3.1. Do the review’s inclusion-exclusion criteria consider sex-gender differences?** • No, review did not meet criteria *Quote: "We included studies of adults requiring short-term urethral catheterisation in hospital for any reason such as urine monitoring, investigations, acute retention problems, and after surgery. These included those suffering from acute illness, urinary retention, perioperative, postoperative, during labour, and during or following surgery."* **3.2. Was there justification or explanation for the exclusion of some groups?** • No, review did not meet criteria |
| Methods | **4.1. Does the review examine whether outcome measures are different for males and females?** • No, review did not meet criteria **4.2. Did the review extract data by sex?** • No, review did not meet criteria **4.3. Did the review extract data on sex of withdrawals and dropouts?** • No, review did not meet criteria **4.4. In cases where sex/gender is used as a proxy for other measures (i.e., weight), is there an explanation for this approach?** • Item was not applicable to the review **4.5. Were any subgroup analyses completed?** • Yes, review met criteria *Quote: "We performed subgroup analyses comparing (a) men versus women, (b) participants undergoing urogenital surgery versus other surgery, and (c) two trials which used antibiotic prophylaxis. In both subgroups there was also no statistical difference in symptomatic UTI (Analysis 1.1.2, 3, 4, 5, 6, 7). We also performed a sensitivity analysis by excluding Barry 1992 PE, as it did not have a definition for symptomatic UTI. There was no difference in the pooled effect when we excluded this trial, so it remains in the meta-analysis."* **4.6. Were subgroup analyses by sex completed?** • Yes, review met criteria *Quote: "We performed subgroup analyses comparing (a) men versus women, (b) participants undergoing urogenital surgery versus other surgery, and (c) two trials which used antibiotic prophylaxis. In both subgroups there was also no statistical difference in symptomatic UTI (Analysis 1.1.2, 3, 4, 5, 6, 7). We also performed a sensitivity analysis by excluding Barry 1992 PE, as it did not have a definition for symptomatic UTI. There was no difference in the pooled effect when we excluded this trial, so it remains in the meta-analysis."* |
| Results and Analysis | **5.1. Do results distinguish between findings for males/females?** • No, review did not meet criteria **5.2. Does the review report conclusions (of effectiveness, efficacy, safety) that are different for men and women?**  • No, review did not meet criteria *Quote: "Six trials enrolled only men (Ahmed 1993; Hammarsten 1992; Katz 1992; Korkes 2008; Prasad 2014; Ratnaval 1996)."* **5.3. If adverse effects are reported, is information sex disaggregated?** • No, review did not meet criteria **5.4. Does review note that subgroup analyses by sex could not be done?** • Item was not applicable to the review |
| Discussion and conclusion | **6.1. Does the review report that primary studies analysed or failed to analyse results by sex?** • No, review did not meet criteria **6.2. Does the review address sex/gender implications for clinical practice?** • No, review did not meet criteria **6.3. Does the review address sex/gender implications for policy and regulation?** • No, review did not meet criteria **6.4. Does the review address sex/gender implications for research?** • Yes, review met criteria  *Quote: "Future trials comparing suprapubic and intermittent urethral catheterisation for short-term use in hospitalised men should be conducted [...]"* |
| Table of included studies | **7.1. Does the description of included studies give detailed information on sex/gender of the study samples?** • No, review did not meet criteria (At least 7.1.1. or 7.1.2. are NO) **7.1.1. Detailed information on SEX of the study samples** • No, review did not meet criteria **7.1.2. Detailed information on GENDER of the study samples** • No, review did not meet criteria |
|  |  |
| **Item** | **Lai (2016)** |
| Background | **1.1 Are the terms sex and gender used in the background?** • No, review did not meet criteria **1.2 Are sex/gender identified as relevant or not to review question?** • No, review did not meet criteria **1.3. Does background discuss why sex/gender differences may be expected?** • No, review did not meet criteria |
| Objectives | **2.1. Are the terms sex, gender, male, or female used in objectives?** • No, review did not meet criteria *Quote: "Our main objective was to assess the effectiveness of antimicrobial impregnation, coating or bonding on CVCs in reducing clinically diagnosed sepsis, catheter-related blood stream infection (CRBSI), all-cause mortality, catheter colonization and other catheter-related infections in adult participants who required central venous catheterization, along with their safety and cost effectiveness where data were available. We undertook the following comparisons: 1) catheters with antimicrobial modifications in the form of antimicrobial impregnation, coating or bonding, against catheters without antimicrobial modifications and 2) catheters with one type of antimicrobial impregnation against catheters with another type of antimicrobial impregnation. We planned to analyse the comparison of catheters with any type of antimicrobial impregnation against catheters with other antimicrobial modifications, e.g. antiseptic dressings, hubs, tunnelling, needleless connectors or antiseptic lock solutions, but did not find any relevant studies. Additionally, we planned to conduct subgroup analyses based on the length of catheter use, settings or levels of care (e.g. intensive care unit, standard ward and oncology unit), baseline risks, definition of sepsis, presence or absence of co-interventions and cost-effectiveness in different currencies."* |
| Criteria for inclusion/ exclusion | **3.1. Do the review’s inclusion-exclusion criteria consider sex-gender differences?** • No, review did not meet criteria **3.2. Was there justification or explanation for the exclusion of some groups?** • No, review did not meet criteria |
| Methods | **4.1. Does the review examine whether outcome measures are different for males and females?** • No, review did not meet criteria **4.2. Did the review extract data by sex?** • No, review did not meet criteria **4.3. Did the review extract data on sex of withdrawals and dropouts?** • No, review did not meet criteria **4.4. In cases where sex/gender is used as a proxy for other measures (i.e., weight), is there an explanation for this approach?** • Item was not applicable to the review **4.5. Were any subgroup analyses completed?** • Yes, review met criteria **4.6. Were subgroup analyses by sex completed?** • No, review did not meet criteria |
| Results and Analysis | **5.1. Do results distinguish between findings for males/females?** • No, review did not meet criteria **5.2. Does the review report conclusions (of effectiveness, efficacy, safety) that are different for men and women?**  • No, review did not meet criteria **5.3. If adverse effects are reported, is information sex disaggregated?** • No, review did not meet criteria **5.4. Does review note that subgroup analyses by sex could not be done?** • Item was not applicable to the review |
| Discussion and conclusion | **6.1. Does the review report that primary studies analysed or failed to analyse results by sex?** • No, review did not meet criteria **6.2. Does the review address sex/gender implications for clinical practice?** • No, review did not meet criteria **6.3. Does the review address sex/gender implications for policy and regulation?** • No, review did not meet criteria **6.4. Does the review address sex/gender implications for research?** • No, review did not meet criteria |
| Table of included studies | **7.1. Does the description of included studies give detailed information on sex/gender of the study samples?** • No, review did not meet criteria (At least 7.1.1. or 7.1.2. are NO) **7.1.1. Detailed information on SEX of the study samples** • No, review did not meet criteria **7.1.2. Detailed information on GENDER of the study samples** • No, review did not meet criteria |
|  |  |
| **Item** | **Lai (2016)** |
| Background | **1.1 Are the terms sex and gender used in the background?** • No, review did not meet criteria **1.2 Are sex/gender identified as relevant or not to review question?** • No, review did not meet criteria **1.3. Does background discuss why sex/gender differences may be expected?** • No, review did not meet criteria |
| Objectives | **2.1. Are the terms sex, gender, male, or female used in objectives?** • No, review did not meet criteria |
| Criteria for inclusion/ exclusion | **3.1. Do the review’s inclusion-exclusion criteria consider sex-gender differences?** • No, review did not meet criteria *Quote: "We included studies involving adults and children cared for in a hospital setting (in adult or paediatric wards or ICUs) with any underlying illness and a CVC inserted for any reason during the study period. Studies that enrolled a patient more than once were acceptable provided that the enrolment took place in separate hospital admissions. We excluded studies conducted in neonatal settings, for example in a neonatal intensive care unit (NICU), as the types of catheters used, the insertion site and techniques, the possible complications as well as the risk factors for sepsis are different compared with those in older children and adults (Trieschmann 2007)."* **3.2. Was there justification or explanation for the exclusion of some groups?** • No, review did not meet criteria |
| Methods | **4.1. Does the review examine whether outcome measures are different for males and females?** • No, review did not meet criteria **4.2. Did the review extract data by sex?** • No, review did not meet criteria **4.3. Did the review extract data on sex of withdrawals and dropouts?** • No, review did not meet criteria **4.4. In cases where sex/gender is used as a proxy for other measures (i.e., weight), is there an explanation for this approach?** • Item was not applicable to the review **4.5. Were any subgroup analyses completed?** • Yes, review met criteria **4.6. Were subgroup analyses by sex completed?** • No, review did not meet criteria |
| Results and Analysis | **5.1. Do results distinguish between findings for males/females?** • No, review did not meet criteria **5.2. Does the review report conclusions (of effectiveness, efficacy, safety) that are different for men and women?**  • No, review did not meet criteria **5.3. If adverse effects are reported, is information sex disaggregated?** • No, review did not meet criteria **5.4. Does review note that subgroup analyses by sex could not be done?** • Item was not applicable to the review |
| Discussion and conclusion | **6.1. Does the review report that primary studies analysed or failed to analyse results by sex?** • No, review did not meet criteria **6.2. Does the review address sex/gender implications for clinical practice?** • No, review did not meet criteria **6.3. Does the review address sex/gender implications for policy and regulation?** • No, review did not meet criteria **6.4. Does the review address sex/gender implications for research?** • No, review did not meet criteria |
| Table of included studies | **7.1. Does the description of included studies give detailed information on sex/gender of the study samples?** • No, review did not meet criteria (At least 7.1.1. or 7.1.2. are NO) **7.1.1. Detailed information on SEX of the study samples** • Unable to determine **7.1.2. Detailed information on GENDER of the study samples** • No, review did not meet criteria |
|  |  |
| **Item** | **Lam (2014)** |
| Background | **1.1 Are the terms sex and gender used in the background?** • Yes, review met criteria *Quote: "Other factors that increase the risk of infection include female gender, older age, impaired immunity, severity of illness (Stamm 1998), and care process factors, such as lack of antibiotic use, longer duration of catheterisation, catheter insertion or maintenance by poorly trained personnel, and deviation from catheter care protocols (CDC 2009)".* **1.2 Are sex/gender identified as relevant or not to review question?** • Unable to determine **1.3. Does background discuss why sex/gender differences may be expected?** • No, review did not meet criteria *Quote: "Other factors that increase the risk of infection include female gender, older age, impaired immunity, severity of illness (Stamm 1998), and care process factors, such as lack of antibiotic use, longer duration of catheterisation, catheter insertion or maintenance by poorly trained personnel, and deviation from catheter care protocols (CDC 2009)".* |
| Objectives | **2.1. Are the terms sex, gender, male, or female used in objectives?** • No, review did not meet criteria *Quote: "The primary objective of this review was to compare the effectiveness of different types of indwelling urethral catheters in reducing the risk of UTI and to assess their impact on other outcomes in adults who require short-term urethral catheterisation in hospitals."* |
| Criteria for inclusion/ exclusion | **3.1. Do the review’s inclusion-exclusion criteria consider sex-gender differences?** • No, review did not meet criteria *Quote: "Hospitalised adults (patients admitted to an adult hospital) with an indwelling urethral catheter of short-term duration (less than or equal to 14 days duration, or other temporary short-term use as defined by the trialists)."* **3.2. Was there justification or explanation for the exclusion of some groups?** • No, review did not meet criteria |
| Methods | **4.1. Does the review examine whether outcome measures are different for males and females?** • No, review did not meet criteria **4.2. Did the review extract data by sex?** • No, review did not meet criteria **4.3. Did the review extract data on sex of withdrawals and dropouts?** • No, review did not meet criteria **4.4. In cases where sex/gender is used as a proxy for other measures (i.e., weight), is there an explanation for this approach?** • Item was not applicable to the review **4.5. Were any subgroup analyses completed?** • Yes, review met criteria *Quote: "Subgroup analysis was possible in one trial (Riley 1995). For bacteriuria reported separately in women and men, there was a reduction of risk of almost one-third with the silver oxide catheter for women (RR 0.63, 95% CI 0.45 to 0.89, Analysis 1.12.2), while for men there was not enough evidence to suggest whether or not there was a difference in risk with the standard catheter (RR 1.62, 95% CI 0.91 to 2.88, Analysis 1.12.3)."* **4.6. Were subgroup analyses by sex completed?** • Yes, review met criteria *Quote: "Subgroup analysis was possible in one trial (Riley 1995). For bacteriuria reported separately in women and men, there was a reduction of risk of almost one-third with the silver oxide catheter for women (RR 0.63, 95% CI 0.45 to 0.89, Analysis 1.12.2), while for men there was not enough evidence to suggest whether or not there was a difference in risk with the standard catheter (RR 1.62, 95% CI 0.91 to 2.88, Analysis 1.12.3)."* |
| Results and Analysis | **5.1. Do results distinguish between findings for males/females?** • Unable to determine *Quote: "Further analysis of women and men separately who received systemic antibiotics suggested that women were protected from bacteriuria with silver oxide catheters (RR 0.50, 95% CI 0.31 to 0.79, Analysis 1.12.5), but there was not enough evidence either way for men (RR 1.02, 95% CI 0.49 to 2.13, Analysis 1.12.6)." Comment: for some outcomes results for men and women were reported separately.* **5.2. Does the review report conclusions (of effectiveness, efficacy, safety) that are different for men and women?**  • Unable to determine *Quote: "Further analysis of women and men separately who received systemic antibiotics suggested that women were protected from bacteriuria with silver oxide catheters (RR 0.50, 95% CI 0.31 to 0.79, Analysis 1.12.5), but there was not enough evidence either way for men (RR 1.02, 95% CI 0.49 to 2.13, Analysis 1.12.6)." Comment: for some outcomes results for men and women were reported separately.* **5.3. If adverse effects are reported, is information sex disaggregated?** • No, review did not meet criteria **5.4. Does review note that subgroup analyses by sex could not be done?** • Item was not applicable to the review |
| Discussion and conclusion | **6.1. Does the review report that primary studies analysed or failed to analyse results by sex?** • No, review did not meet criteria **6.2. Does the review address sex/gender implications for clinical practice?** • Yes, review met criteria **6.3. Does the review address sex/gender implications for policy and regulation?** • No, review did not meet criteria **6.4. Does the review address sex/gender implications for research?** • No, review did not meet criteria |
| Table of included studies | **7.1. Does the description of included studies give detailed information on sex/gender of the study samples?** • No, review did not meet criteria (At least 7.1.1. or 7.1.2. are NO) **7.1.1. Detailed information on SEX of the study samples** • No, review did not meet criteria **7.1.2. Detailed information on GENDER of the study samples** • No, review did not meet criteria |
|  |  |
| **Item** | **Lethaby (2013)** |
| Background | **1.1 Are the terms sex and gender used in the background?** • No, review did not meet criteria **1.2 Are sex/gender identified as relevant or not to review question?** • No, review did not meet criteria **1.3. Does background discuss why sex/gender differences may be expected?** • No, review did not meet criteria |
| Objectives | **2.1. Are the terms sex, gender, male, or female used in objectives?** • No, review did not meet criteria *Quote: "To assess the evidence for the effects of cleansing, massage and dressing techniques for pin sites on postoperative infection."* |
| Criteria for inclusion/ exclusion | **3.1. Do the review’s inclusion-exclusion criteria consider sex-gender differences?** • No, review did not meet criteria *Quote: "Adults and children with pins inserted for either external fixators or skeletal traction. Studies of treatment regimens that set out to manage established infections were not included. Studies of people of any age and any care setting were included."* **3.2. Was there justification or explanation for the exclusion of some groups?** • No, review did not meet criteria |
| Methods | **4.1. Does the review examine whether outcome measures are different for males and females?** • No, review did not meet criteria **4.2. Did the review extract data by sex?** • No, review did not meet criteria **4.3. Did the review extract data on sex of withdrawals and dropouts?** • No, review did not meet criteria **4.4. In cases where sex/gender is used as a proxy for other measures (i.e., weight), is there an explanation for this approach?** • Item was not applicable to the review **4.5. Were any subgroup analyses completed?** • No, review did not meet criteria **4.6. Were subgroup analyses by sex completed?** • No, review did not meet criteria |
| Results and Analysis | **5.1. Do results distinguish between findings for males/females?** • No, review did not meet criteria **5.2. Does the review report conclusions (of effectiveness, efficacy, safety) that are different for men and women?**  • No, review did not meet criteria **5.3. If adverse effects are reported, is information sex disaggregated?** • Item was not applicable to the review **5.4. Does review note that subgroup analyses by sex could not be done?** • Unable to determine *Quote: "A priori, it was planned to undertake sensitivity analyses and subgroup analyses to compare results from the pooled data according to differences in the quality of the trials, and differences in participants, interventions and outcomes". Comment: Not clear if sex was planned for subgroup analysis.* |
| Discussion and conclusion | **6.1. Does the review report that primary studies analysed or failed to analyse results by sex?** • No, review did not meet criteria **6.2. Does the review address sex/gender implications for clinical practice?** • No, review did not meet criteria **6.3. Does the review address sex/gender implications for policy and regulation?** • No, review did not meet criteria **6.4. Does the review address sex/gender implications for research?** • No, review did not meet criteria |
| Table of included studies | **7.1. Does the description of included studies give detailed information on sex/gender of the study samples?** • No, review did not meet criteria (At least 7.1.1. or 7.1.2. are NO) **7.1.1. Detailed information on SEX of the study samples** • No, review did not meet criteria **7.1.2. Detailed information on GENDER of the study samples** • No, review did not meet criteria |
|  |  |
| **Item** | **Liabsuetrakul (2014)** |
| Background | **1.1 Are the terms sex and gender used in the background?** • Item was not applicable to the review **1.2 Are sex/gender identified as relevant or not to review question?** • Item was not applicable to the review **1.3. Does background discuss why sex/gender differences may be expected?** • Item was not applicable to the review |
| Objectives | **2.1. Are the terms sex, gender, male, or female used in objectives?** • Item was not applicable to the review |
| Criteria for inclusion/ exclusion | **3.1. Do the review’s inclusion-exclusion criteria consider sex-gender differences?** • Item was not applicable to the review **3.2. Was there justification or explanation for the exclusion of some groups?** • Item was not applicable to the review |
| Methods | **4.1. Does the review examine whether outcome measures are different for males and females?** • Item was not applicable to the review **4.2. Did the review extract data by sex?** • Item was not applicable to the review **4.3. Did the review extract data on sex of withdrawals and dropouts?** • Item was not applicable to the review **4.4. In cases where sex/gender is used as a proxy for other measures (i.e., weight), is there an explanation for this approach?** • Item was not applicable to the review **4.5. Were any subgroup analyses completed?** • Item was not applicable to the review **4.6. Were subgroup analyses by sex completed?** • Item was not applicable to the review |
| Results and Analysis | **5.1. Do results distinguish between findings for males/females?** • Item was not applicable to the review **5.2. Does the review report conclusions (of effectiveness, efficacy, safety) that are different for men and women?**  • Item was not applicable to the review **5.3. If adverse effects are reported, is information sex disaggregated?** • Item was not applicable to the review **5.4. Does review note that subgroup analyses by sex could not be done?** • Item was not applicable to the review |
| Discussion and conclusion | **6.1. Does the review report that primary studies analysed or failed to analyse results by sex?** • Item was not applicable to the review **6.2. Does the review address sex/gender implications for clinical practice?** • Item was not applicable to the review **6.3. Does the review address sex/gender implications for policy and regulation?** • Item was not applicable to the review **6.4. Does the review address sex/gender implications for research?** • Item was not applicable to the review |
| Table of included studies | **7.1. Does the description of included studies give detailed information on sex/gender of the study samples?** • Item was not applicable to the review **7.1.1. Detailed information on SEX of the study samples** • Item was not applicable to the review **7.1.2. Detailed information on GENDER of the study samples** • Item was not applicable to the review |
|  |  |
| **Item** | **Lipp (2013)** |
| Background | **1.1 Are the terms sex and gender used in the background?** • No, review did not meet criteria **1.2 Are sex/gender identified as relevant or not to review question?** • No, review did not meet criteria **1.3. Does background discuss why sex/gender differences may be expected?** • No, review did not meet criteria |
| Objectives | **2.1. Are the terms sex, gender, male, or female used in objectives?** • No, review did not meet criteria |
| Criteria for inclusion/ exclusion | **3.1. Do the review’s inclusion-exclusion criteria consider sex-gender differences?** • No, review did not meet criteria *Quote: "Studies in people of any age, gender or diagnosis, undergoing placement of a PEG tube (the placement of a feeding tube through the anterior abdominal wall of the stomach using an endoscopic technique). Studies in people undergoing replacement of PEG tubes were excluded, along with those undergoing percutaneous endoscopic jejunostomy (PEJ), or percutaneous endoscopic duodenostomy (PED)."* **3.2. Was there justification or explanation for the exclusion of some groups?** • No, review did not meet criteria |
| Methods | **4.1. Does the review examine whether outcome measures are different for males and females?** • No, review did not meet criteria **4.2. Did the review extract data by sex?** • No, review did not meet criteria **4.3. Did the review extract data on sex of withdrawals and dropouts?** • No, review did not meet criteria **4.4. In cases where sex/gender is used as a proxy for other measures (i.e., weight), is there an explanation for this approach?** • Item was not applicable to the review **4.5. Were any subgroup analyses completed?** • Yes, review met criteria *Quote: "The data allowed us to perform the following pre-planned subgroup analyses that assessed: • the impact of study validity on outcomes (i.e. adequate allocation concealment compared with inadequate allocation concealment); • the use of different antimicrobials (cephalosporins compared with penicillins); and, • commercial sponsorship of trials. We were unable to perform other pre-planned subgroup analyses to investigate specific patient groups (neonate, child, adult); different diagnostic groups (paediatric, head and neck cancer); or PEG tube placement techniques (push versus pull methods)."* **4.6. Were subgroup analyses by sex completed?** • No, review did not meet criteria |
| Results and Analysis | **5.1. Do results distinguish between findings for males/females?** • No, review did not meet criteria **5.2. Does the review report conclusions (of effectiveness, efficacy, safety) that are different for men and women?**  • No, review did not meet criteria **5.3. If adverse effects are reported, is information sex disaggregated?** • No, review did not meet criteria **5.4. Does review note that subgroup analyses by sex could not be done?** • Item was not applicable to the review |
| Discussion and conclusion | **6.1. Does the review report that primary studies analysed or failed to analyse results by sex?** • No, review did not meet criteria **6.2. Does the review address sex/gender implications for clinical practice?** • No, review did not meet criteria **6.3. Does the review address sex/gender implications for policy and regulation?** • No, review did not meet criteria **6.4. Does the review address sex/gender implications for research?** • No, review did not meet criteria |
| Table of included studies | **7.1. Does the description of included studies give detailed information on sex/gender of the study samples?** • No, review did not meet criteria (At least 7.1.1. or 7.1.2. are NO) **7.1.1. Detailed information on SEX of the study samples** • No, review did not meet criteria **7.1.2. Detailed information on GENDER of the study samples** • No, review did not meet criteria |
|  |  |
| **Item** | **Lo (2015)** |
| Background | **1.1 Are the terms sex and gender used in the background?** • No, review did not meet criteria **1.2 Are sex/gender identified as relevant or not to review question?** • No, review did not meet criteria **1.3. Does background discuss why sex/gender differences may be expected?** • No, review did not meet criteria |
| Objectives | **2.1. Are the terms sex, gender, male, or female used in objectives?** • No, review did not meet criteria *Quote: "Objectives: To evaluate the effectiveness of treatment regimens designed to eradicate MRSA and to determine whether the eradication of MRSA confers better clinical and microbiological outcomes for patients with CF. To ascertain whether attempts at eradicating MRSA can lead to increased acquisition of other resistant organisms (including P. aeruginosa) or increased adverse effects from drugs, or both.”* |
| Criteria for inclusion/ exclusion | **3.1. Do the review’s inclusion-exclusion criteria consider sex-gender differences?** • No, review did not meet criteria *Quote: “Children and adults diagnosed with CF clinically and by sweat or genetic testing with a confirmed positive microbiological isolate of MRSA on clinically relevant CF respiratory cultures (bronchoalveolar lavage (BAL), cough or oropharyngeal swab, spontaneous or induced sputum culture) specimen prior to enrolment into the trial. We included all disease severities. We did not include patients with nasal carriage of MRSA alone in this review."* **3.2. Was there justification or explanation for the exclusion of some groups?** • No, review did not meet criteria *Quote: “Children and adults diagnosed with CF clinically and by sweat or genetic testing with a confirmed positive microbiological isolate of MRSA on clinically relevant CF respiratory cultures (bronchoalveolar lavage (BAL), cough or oropharyngeal swab, spontaneous or induced sputum culture) specimen prior to enrolment into the trial. We included all disease severities. We did not include patients with nasal carriage of MRSA alone in this review."* |
| Methods | **4.1. Does the review examine whether outcome measures are different for males and females?** • No, review did not meet criteria **4.2. Did the review extract data by sex?** • No, review did not meet criteria **4.3. Did the review extract data on sex of withdrawals and dropouts?** • No, review did not meet criteria **4.4. In cases where sex/gender is used as a proxy for other measures (i.e., weight), is there an explanation for this approach?** • Item was not applicable to the review **4.5. Were any subgroup analyses completed?** • No, review did not meet criteria **4.6. Were subgroup analyses by sex completed?** • No, review did not meet criteria |
| Results and Analysis | **5.1. Do results distinguish between findings for males/females?** • Item was not applicable to the review *Comment: No included studies* **5.2. Does the review report conclusions (of effectiveness, efficacy, safety) that are different for men and women?**  • Item was not applicable to the review *Comment: No included studies* **5.3. If adverse effects are reported, is information sex disaggregated?** • Item was not applicable to the review *Comment: No included studies* **5.4. Does review note that subgroup analyses by sex could not be done?** • Item was not applicable to the review *Comment: No included studies* |
| Discussion and conclusion | **6.1. Does the review report that primary studies analysed or failed to analyse results by sex?** • Item was not applicable to the review *Comment: Empty review.* **6.2. Does the review address sex/gender implications for clinical practice?** • No, review did not meet criteria **6.3. Does the review address sex/gender implications for policy and regulation?** • No, review did not meet criteria **6.4. Does the review address sex/gender implications for research?** • No, review did not meet criteria |
| Table of included studies | **7.1. Does the description of included studies give detailed information on sex/gender of the study samples?** • Item was not applicable to the review *Comment: empty review.*  **7.1.1. Detailed information on SEX of the study samples** • Item was not applicable to the review *Comment: empty review.*  **7.1.2. Detailed information on GENDER of the study samples** • Item was not applicable to the review *Comment: empty review.* |
|  |  |
| **Item** | **Lodi (2012)** |
| Background | **1.1 Are the terms sex and gender used in the background?** • No, review did not meet criteria **1.2 Are sex/gender identified as relevant or not to review question?** • No, review did not meet criteria **1.3. Does background discuss why sex/gender differences may be expected?** • No, review did not meet criteria |
| Objectives | **2.1. Are the terms sex, gender, male, or female used in objectives?** • No, review did not meet criteria |
| Criteria for inclusion/ exclusion | **3.1. Do the review’s inclusion-exclusion criteria consider sex-gender differences?** • No, review did not meet criteria *Quote: "Anyone undergoing a tooth extraction, including extraction of impacted teeth."* **3.2. Was there justification or explanation for the exclusion of some groups?** • No, review did not meet criteria |
| Methods | **4.1. Does the review examine whether outcome measures are different for males and females?** • No, review did not meet criteria **4.2. Did the review extract data by sex?** • No, review did not meet criteria **4.3. Did the review extract data on sex of withdrawals and dropouts?** • No, review did not meet criteria **4.4. In cases where sex/gender is used as a proxy for other measures (i.e., weight), is there an explanation for this approach?** • Item was not applicable to the review **4.5. Were any subgroup analyses completed?** • Yes, review met criteria *Quote: "Whenever possible, subgroup analyses were undertaken based on time of administration (pre- or post-procedure) and the presence or absence of patients with systemic conditions (HIV, diabetes, etc)." Quote: "The results from the remaining 15 trials are described below in subgroups depending on the time(s) the antibiotics were administered (either pre-operatively, post-operatively or both pre- and post-operatively)."* **4.6. Were subgroup analyses by sex completed?** • No, review did not meet criteria |
| Results and Analysis | **5.1. Do results distinguish between findings for males/females?** • No, review did not meet criteria **5.2. Does the review report conclusions (of effectiveness, efficacy, safety) that are different for men and women?**  • No, review did not meet criteria **5.3. If adverse effects are reported, is information sex disaggregated?** • No, review did not meet criteria **5.4. Does review note that subgroup analyses by sex could not be done?** • Item was not applicable to the review |
| Discussion and conclusion | **6.1. Does the review report that primary studies analysed or failed to analyse results by sex?** • No, review did not meet criteria **6.2. Does the review address sex/gender implications for clinical practice?** • No, review did not meet criteria **6.3. Does the review address sex/gender implications for policy and regulation?** • No, review did not meet criteria **6.4. Does the review address sex/gender implications for research?** • No, review did not meet criteria |
| Table of included studies | **7.1. Does the description of included studies give detailed information on sex/gender of the study samples?** • No, review did not meet criteria (At least 7.1.1. or 7.1.2. are NO) **7.1.1. Detailed information on SEX of the study samples** • No, review did not meet criteria **7.1.2. Detailed information on GENDER of the study samples** • No, review did not meet criteria |
|  |  |
| **Item** | **Loeb (2003)** |
| Background | **1.1 Are the terms sex and gender used in the background?** • No, review did not meet criteria **1.2 Are sex/gender identified as relevant or not to review question?** • No, review did not meet criteria **1.3. Does background discuss why sex/gender differences may be expected?** • No, review did not meet criteria |
| Objectives | **2.1. Are the terms sex, gender, male, or female used in objectives?** • No, review did not meet criteria |
| Criteria for inclusion/ exclusion | **3.1. Do the review’s inclusion-exclusion criteria consider sex-gender differences?** • No, review did not meet criteria *Quote: "People colonized with MRSA either nasally or at extra-nasal sites (including perineum, wounds, axilla, groin, catheter exit sites). Excluded: healthcare workers".* **3.2. Was there justification or explanation for the exclusion of some groups?** • No, review did not meet criteria |
| Methods | **4.1. Does the review examine whether outcome measures are different for males and females?** • No, review did not meet criteria **4.2. Did the review extract data by sex?** • No, review did not meet criteria **4.3. Did the review extract data on sex of withdrawals and dropouts?** • No, review did not meet criteria **4.4. In cases where sex/gender is used as a proxy for other measures (i.e., weight), is there an explanation for this approach?** • Item was not applicable to the review **4.5. Were any subgroup analyses completed?** • No, review did not meet criteria **4.6. Were subgroup analyses by sex completed?** • No, review did not meet criteria |
| Results and Analysis | **5.1. Do results distinguish between findings for males/females?** • No, review did not meet criteria **5.2. Does the review report conclusions (of effectiveness, efficacy, safety) that are different for men and women?**  • No, review did not meet criteria **5.3. If adverse effects are reported, is information sex disaggregated?** • No, review did not meet criteria *Quote: "Adverse events: None were reported for placebo or treatment trials."* **5.4. Does review note that subgroup analyses by sex could not be done?** • Item was not applicable to the review *Comment: Subgroup analysis by sex was not planned.* |
| Discussion and conclusion | **6.1. Does the review report that primary studies analysed or failed to analyse results by sex?** • No, review did not meet criteria **6.2. Does the review address sex/gender implications for clinical practice?** • No, review did not meet criteria **6.3. Does the review address sex/gender implications for policy and regulation?** • No, review did not meet criteria **6.4. Does the review address sex/gender implications for research?** • No, review did not meet criteria |
| Table of included studies | **7.1. Does the description of included studies give detailed information on sex/gender of the study samples?** • No, review did not meet criteria (At least 7.1.1. or 7.1.2. are NO) **7.1.1. Detailed information on SEX of the study samples** • No, review did not meet criteria *Comment: No information on sex.* **7.1.2. Detailed information on GENDER of the study samples** • Unable to determine |
|  |  |
| **Item** | **López-Alcalde (2015)** |
| Background | **1.1 Are the terms sex and gender used in the background?** • No, review did not meet criteria **1.2 Are sex/gender identified as relevant or not to review question?** • No, review did not meet criteria **1.3. Does background discuss why sex/gender differences may be expected?** • No, review did not meet criteria |
| Objectives | **2.1. Are the terms sex, gender, male, or female used in objectives?** • No, review did not meet criteria |
| Criteria for inclusion/ exclusion | **3.1. Do the review’s inclusion-exclusion criteria consider sex-gender differences?** • No, review did not meet criteria **3.2. Was there justification or explanation for the exclusion of some groups?** • No, review did not meet criteria *Comment: The review did not exclude groups based on sex-gender”. Comment: But the review did not justify this.* |
| Methods | **4.1. Does the review examine whether outcome measures are different for males and females?** • No, review did not meet criteria *Comment: No included studies but there was no plan to examine whether outcomes measures were different by sex.* **4.2. Did the review extract data by sex?** • No, review did not meet criteria *Comment: No included studies, but there was no plan to extract data by sex.* **4.3. Did the review extract data on sex of withdrawals and dropouts?** • No, review did not meet criteria *Comment: No included studies, but there was no plan to extract data by sex.* **4.4. In cases where sex/gender is used as a proxy for other measures (i.e., weight), is there an explanation for this approach?** • Item was not applicable to the review **4.5. Were any subgroup analyses completed?** • No, review did not meet criteria **4.6. Were subgroup analyses by sex completed?** • No, review did not meet criteria |
| Results and Analysis | **5.1. Do results distinguish between findings for males/females?** • Item was not applicable to the review *Comment: No included studies* **5.2. Does the review report conclusions (of effectiveness, efficacy, safety) that are different for men and women?**  • Item was not applicable to the review *Comment: No included studies* **5.3. If adverse effects are reported, is information sex disaggregated?** • Item was not applicable to the review *Comment: No included studies* **5.4. Does review note that subgroup analyses by sex could not be done?** • Item was not applicable to the review *Comment: No included studies* |
| Discussion and conclusion | **6.1. Does the review report that primary studies analysed or failed to analyse results by sex?** • Item was not applicable to the review *Comment: Empty review.* **6.2. Does the review address sex/gender implications for clinical practice?** • No, review did not meet criteria **6.3. Does the review address sex/gender implications for policy and regulation?** • No, review did not meet criteria **6.4. Does the review address sex/gender implications for research?** • No, review did not meet criteria |
| Table of included studies | **7.1. Does the description of included studies give detailed information on sex/gender of the study samples?** • Item was not applicable to the review *Comment: No included studies so no tables of included studies* **7.1.1. Detailed information on SEX of the study samples** • Item was not applicable to the review *Comment: No included studies* **7.1.2. Detailed information on GENDER of the study samples** • Item was not applicable to the review *Comment: No included studies* |
|  |  |
| **Item** | **López-Briz (2014)** |
| Background | **1.1 Are the terms sex and gender used in the background?** • No, review did not meet criteria **1.2 Are sex/gender identified as relevant or not to review question?** • No, review did not meet criteria **1.3. Does background discuss why sex/gender differences may be expected?** • No, review did not meet criteria |
| Objectives | **2.1. Are the terms sex, gender, male, or female used in objectives?** • No, review did not meet criteria *Quote: "To assess the effectiveness of intermittent flushing with heparin versus 0.9% sodium chloride (normal saline) solution in adults with central venous catheters in terms of prevention of occlusion and overall benefits versus harms."* |
| Criteria for inclusion/ exclusion | **3.1. Do the review’s inclusion-exclusion criteria consider sex-gender differences?** • No, review did not meet criteria *Quote: "Adults 18 years of age or older with a CVC. Studies on infants and children were excluded from this review, as they are the topic of another Cochrane review (Bradford 2014)."*  **3.2. Was there justification or explanation for the exclusion of some groups?** • No, review did not meet criteria |
| Methods | **4.1. Does the review examine whether outcome measures are different for males and females?** • No, review did not meet criteria **4.2. Did the review extract data by sex?** • No, review did not meet criteria **4.3. Did the review extract data on sex of withdrawals and dropouts?** • No, review did not meet criteria **4.4. In cases where sex/gender is used as a proxy for other measures (i.e., weight), is there an explanation for this approach?** • Item was not applicable to the review **4.5. Were any subgroup analyses completed?** • No, review did not meet criteria **4.6. Were subgroup analyses by sex completed?** • No, review did not meet criteria |
| Results and Analysis | **5.1. Do results distinguish between findings for males/females?** • No, review did not meet criteria **5.2. Does the review report conclusions (of effectiveness, efficacy, safety) that are different for men and women?**  • No, review did not meet criteria **5.3. If adverse effects are reported, is information sex disaggregated?** • No, review did not meet criteria **5.4. Does review note that subgroup analyses by sex could not be done?** • Item was not applicable to the review |
| Discussion and conclusion | **6.1. Does the review report that primary studies analysed or failed to analyse results by sex?** • No, review did not meet criteria *Quote: "Risk factors for HIT include type of heparin (greater risk with unfractionated heparin), duration of exposure, patient setting and patient gender (1.5 to 2 times higher in women)."* **6.2. Does the review address sex/gender implications for clinical practice?** • No, review did not meet criteria **6.3. Does the review address sex/gender implications for policy and regulation?** • No, review did not meet criteria **6.4. Does the review address sex/gender implications for research?** • No, review did not meet criteria |
| Table of included studies | **7.1. Does the description of included studies give detailed information on sex/gender of the study samples?** • No, review did not meet criteria (At least 7.1.1. or 7.1.2. are NO) **7.1.1. Detailed information on SEX of the study samples** • No, review did not meet criteria **7.1.2. Detailed information on GENDER of the study samples** • No, review did not meet criteria |
|  |  |
| **Item** | **Low (2012)** |
| Background | **1.1 Are the terms sex and gender used in the background?** • Item was not applicable to the review **1.2 Are sex/gender identified as relevant or not to review question?** • Item was not applicable to the review **1.3. Does background discuss why sex/gender differences may be expected?** • Item was not applicable to the review |
| Objectives | **2.1. Are the terms sex, gender, male, or female used in objectives?** • Item was not applicable to the review |
| Criteria for inclusion/ exclusion | **3.1. Do the review’s inclusion-exclusion criteria consider sex-gender differences?** • Item was not applicable to the review **3.2. Was there justification or explanation for the exclusion of some groups?** • Item was not applicable to the review |
| Methods | **4.1. Does the review examine whether outcome measures are different for males and females?** • Item was not applicable to the review **4.2. Did the review extract data by sex?** • Item was not applicable to the review **4.3. Did the review extract data on sex of withdrawals and dropouts?** • Item was not applicable to the review **4.4. In cases where sex/gender is used as a proxy for other measures (i.e., weight), is there an explanation for this approach?** • Item was not applicable to the review **4.5. Were any subgroup analyses completed?** • Item was not applicable to the review **4.6. Were subgroup analyses by sex completed?** • Item was not applicable to the review |
| Results and Analysis | **5.1. Do results distinguish between findings for males/females?** • Item was not applicable to the review **5.2. Does the review report conclusions (of effectiveness, efficacy, safety) that are different for men and women?**  • Item was not applicable to the review **5.3. If adverse effects are reported, is information sex disaggregated?** • Item was not applicable to the review **5.4. Does review note that subgroup analyses by sex could not be done?** • Item was not applicable to the review |
| Discussion and conclusion | **6.1. Does the review report that primary studies analysed or failed to analyse results by sex?** • Item was not applicable to the review **6.2. Does the review address sex/gender implications for clinical practice?** • Item was not applicable to the review **6.3. Does the review address sex/gender implications for policy and regulation?** • Item was not applicable to the review **6.4. Does the review address sex/gender implications for research?** • Item was not applicable to the review |
| Table of included studies | **7.1. Does the description of included studies give detailed information on sex/gender of the study samples?** • Item was not applicable to the review **7.1.1. Detailed information on SEX of the study samples** • Item was not applicable to the review **7.1.2. Detailed information on GENDER of the study samples** • Item was not applicable to the review |
|  |  |
| **Item** | **Lusardi (2013)** |
| Background | **1.1 Are the terms sex and gender used in the background?** • No, review did not meet criteria **1.2 Are sex/gender identified as relevant or not to review question?** • No, review did not meet criteria **1.3. Does background discuss why sex/gender differences may be expected?** • No, review did not meet criteria |
| Objectives | **2.1. Are the terms sex, gender, male, or female used in objectives?** • No, review did not meet criteria *Quote: "To determine if one type of antibiotic prophylaxis is better than another or none in terms of prevention of urinary tract infections, complications, quality of life and cost-effectiveness for short-term catheterised adults."* |
| Criteria for inclusion/ exclusion | **3.1. Do the review’s inclusion-exclusion criteria consider sex-gender differences?** • No, review did not meet criteria *Quote: "All adults requiring short-term urinary urethral and supra-pubic catheterisation (up to and including 14 days) in hospital for urine monitoring, investigations, acute retention problems, and acute incontinence problems. These include those suffering from general medical problems, acute illness, urinary retention and following surgery (excluding urodynamics (Foon 2012) and transurethral surgical procedures (Alsaywid 2012))."* **3.2. Was there justification or explanation for the exclusion of some groups?** • No, review did not meet criteria |
| Methods | **4.1. Does the review examine whether outcome measures are different for males and females?** • No, review did not meet criteria **4.2. Did the review extract data by sex?** • No, review did not meet criteria **4.3. Did the review extract data on sex of withdrawals and dropouts?** • No, review did not meet criteria **4.4. In cases where sex/gender is used as a proxy for other measures (i.e., weight), is there an explanation for this approach?** • Item was not applicable to the review **4.5. Were any subgroup analyses completed?** • No, review did not meet criteria **4.6. Were subgroup analyses by sex completed?** • No, review did not meet criteria |
| Results and Analysis | **5.1. Do results distinguish between findings for males/females?** • No, review did not meet criteria **5.2. Does the review report conclusions (of effectiveness, efficacy, safety) that are different for men and women?**  • No, review did not meet criteria **5.3. If adverse effects are reported, is information sex disaggregated?** • No, review did not meet criteria **5.4. Does review note that subgroup analyses by sex could not be done?** • Yes, review met criteria |
| Discussion and conclusion | **6.1. Does the review report that primary studies analysed or failed to analyse results by sex?** • No, review did not meet criteria **6.2. Does the review address sex/gender implications for clinical practice?** • No, review did not meet criteria **6.3. Does the review address sex/gender implications for policy and regulation?** • No, review did not meet criteria **6.4. Does the review address sex/gender implications for research?** • No, review did not meet criteria |
| Table of included studies | **7.1. Does the description of included studies give detailed information on sex/gender of the study samples?** • No, review did not meet criteria (At least 7.1.1. or 7.1.2. are NO) **7.1.1. Detailed information on SEX of the study samples** • No, review did not meet criteria **7.1.2. Detailed information on GENDER of the study samples** • No, review did not meet criteria |
|  |  |
| **Item** | **Mackeen (2014)** |
| Background | **1.1 Are the terms sex and gender used in the background?** • Item was not applicable to the review **1.2 Are sex/gender identified as relevant or not to review question?** • Item was not applicable to the review **1.3. Does background discuss why sex/gender differences may be expected?** • Item was not applicable to the review |
| Objectives | **2.1. Are the terms sex, gender, male, or female used in objectives?** • Item was not applicable to the review |
| Criteria for inclusion/ exclusion | **3.1. Do the review’s inclusion-exclusion criteria consider sex-gender differences?** • Item was not applicable to the review **3.2. Was there justification or explanation for the exclusion of some groups?** • Item was not applicable to the review |
| Methods | **4.1. Does the review examine whether outcome measures are different for males and females?** • Item was not applicable to the review **4.2. Did the review extract data by sex?** • Item was not applicable to the review **4.3. Did the review extract data on sex of withdrawals and dropouts?** • Item was not applicable to the review **4.4. In cases where sex/gender is used as a proxy for other measures (i.e., weight), is there an explanation for this approach?** • Item was not applicable to the review **4.5. Were any subgroup analyses completed?** • Item was not applicable to the review **4.6. Were subgroup analyses by sex completed?** • Item was not applicable to the review |
| Results and Analysis | **5.1. Do results distinguish between findings for males/females?** • Item was not applicable to the review **5.2. Does the review report conclusions (of effectiveness, efficacy, safety) that are different for men and women?**  • Item was not applicable to the review **5.3. If adverse effects are reported, is information sex disaggregated?** • Item was not applicable to the review **5.4. Does review note that subgroup analyses by sex could not be done?** • Item was not applicable to the review |
| Discussion and conclusion | **6.1. Does the review report that primary studies analysed or failed to analyse results by sex?** • Item was not applicable to the review **6.2. Does the review address sex/gender implications for clinical practice?** • No, review did not meet criteria • Item was not applicable to the review **6.3. Does the review address sex/gender implications for policy and regulation?** • Item was not applicable to the review **6.4. Does the review address sex/gender implications for research?** • Item was not applicable to the review |
| Table of included studies | **7.1. Does the description of included studies give detailed information on sex/gender of the study samples?** • Item was not applicable to the review **7.1.1. Detailed information on SEX of the study samples** • Item was not applicable to the review **7.1.2. Detailed information on GENDER of the study samples** • Item was not applicable to the review |
|  |  |
| **Item** | **Madrid (2016)** |
| Background | **1.1 Are the terms sex and gender used in the background?** • No, review did not meet criteria **1.2 Are sex/gender identified as relevant or not to review question?** • No, review did not meet criteria **1.3. Does background discuss why sex/gender differences may be expected?** • No, review did not meet criteria |
| Objectives | **2.1. Are the terms sex, gender, male, or female used in objectives?** • No, review did not meet criteria |
| Criteria for inclusion/ exclusion | **3.1. Do the review’s inclusion-exclusion criteria consider sex-gender differences?** • No, review did not meet criteria *Quote: "We only included adults undergoing a scheduled surgery (including ambulatory surgery), except surgery using intended hypothermia (such as off-pump surgery and certain neurosurgical interventions)."* **3.2. Was there justification or explanation for the exclusion of some groups?** • No, review did not meet criteria |
| Methods | **4.1. Does the review examine whether outcome measures are different for males and females?** • No, review did not meet criteria **4.2. Did the review extract data by sex?** • No, review did not meet criteria **4.3. Did the review extract data on sex of withdrawals and dropouts?** • No, review did not meet criteria **4.4. In cases where sex/gender is used as a proxy for other measures (i.e., weight), is there an explanation for this approach?** • Item was not applicable to the review **4.5. Were any subgroup analyses completed?** • Yes, review met criteria *Quote: "We conducted subgroup analyses for the comparison ABSW versus control for all outcomes. We applied a test for subgroup differences based on the I² value. We have analysed the following subgroups: 1. Type of anaesthesia (general or combined anaesthesia versus exclusively regional anaesthesia). 2. Timing of application of the intervention (preoperatively, intraoperatively, or both preoperatively and intraoperatively). We did not run the other planned subgroup analyses, based on type of surgery and use of premedications."* **4.6. Were subgroup analyses by sex completed?** • No, review did not meet criteria |
| Results and Analysis | **5.1. Do results distinguish between findings for males/females?** • No, review did not meet criteria **5.2. Does the review report conclusions (of effectiveness, efficacy, safety) that are different for men and women?**  • No, review did not meet criteria **5.3. If adverse effects are reported, is information sex disaggregated?** • No, review did not meet criteria **5.4. Does review note that subgroup analyses by sex could not be done?** • Item was not applicable to the review |
| Discussion and conclusion | **6.1. Does the review report that primary studies analysed or failed to analyse results by sex?** • No, review did not meet criteria **6.2. Does the review address sex/gender implications for clinical practice?** • No, review did not meet criteria **6.3. Does the review address sex/gender implications for policy and regulation?** • No, review did not meet criteria **6.4. Does the review address sex/gender implications for research?** • No, review did not meet criteria |
| Table of included studies | **7.1. Does the description of included studies give detailed information on sex/gender of the study samples?** • No, review did not meet criteria (At least 7.1.1. or 7.1.2. are NO) **7.1.1. Detailed information on SEX of the study samples** • No, review did not meet criteria **7.1.2. Detailed information on GENDER of the study samples** • Unable to determine |
|  |  |
| **Item** | **Marsh (2015)** |
| Background | **1.1 Are the terms sex and gender used in the background?** • No, review did not meet criteria **1.2 Are sex/gender identified as relevant or not to review question?** • No, review did not meet criteria **1.3. Does background discuss why sex/gender differences may be expected?** • No, review did not meet criteria |
| Objectives | **2.1. Are the terms sex, gender, male, or female used in objectives?** • No, review did not meet criteria *Quote: "To assess the effects of PVC dressings and securement devices on the incidence of PVC failure."* |
| Criteria for inclusion/ exclusion | **3.1. Do the review’s inclusion-exclusion criteria consider sex-gender differences?** • No, review did not meet criteria **3.2. Was there justification or explanation for the exclusion of some groups?** • No, review did not meet criteria |
| Methods | **4.1. Does the review examine whether outcome measures are different for males and females?** • No, review did not meet criteria **4.2. Did the review extract data by sex?** • No, review did not meet criteria **4.3. Did the review extract data on sex of withdrawals and dropouts?** • No, review did not meet criteria **4.4. In cases where sex/gender is used as a proxy for other measures (i.e., weight), is there an explanation for this approach?** • Item was not applicable to the review **4.5. Were any subgroup analyses completed?** • No, review did not meet criteria **4.6. Were subgroup analyses by sex completed?** • No, review did not meet criteria |
| Results and Analysis | **5.1. Do results distinguish between findings for males/females?** • No, review did not meet criteria **5.2. Does the review report conclusions (of effectiveness, efficacy, safety) that are different for men and women?**  • No, review did not meet criteria **5.3. If adverse effects are reported, is information sex disaggregated?** • No, review did not meet criteria **5.4. Does review note that subgroup analyses by sex could not be done?** • Item was not applicable to the review |
| Discussion and conclusion | **6.1. Does the review report that primary studies analysed or failed to analyse results by sex?** • No, review did not meet criteria **6.2. Does the review address sex/gender implications for clinical practice?** • No, review did not meet criteria **6.3. Does the review address sex/gender implications for policy and regulation?** • No, review did not meet criteria **6.4. Does the review address sex/gender implications for research?** • No, review did not meet criteria |
| Table of included studies | **7.1. Does the description of included studies give detailed information on sex/gender of the study samples?** • No, review did not meet criteria (At least 7.1.1. or 7.1.2. are NO) **7.1.1. Detailed information on SEX of the study samples** • Unable to determine **7.1.2. Detailed information on GENDER of the study samples** • No, review did not meet criteria |
|  |  |
| **Item** | **Masters (2012)** |
| Background | **1.1 Are the terms sex and gender used in the background?** • No, review did not meet criteria **1.2 Are sex/gender identified as relevant or not to review question?** • No, review did not meet criteria **1.3. Does background discuss why sex/gender differences may be expected?** • No, review did not meet criteria |
| Objectives | **2.1. Are the terms sex, gender, male, or female used in objectives?** • No, review did not meet criteria *Quote: "To examine the evidence of improved clinical outcomes in burn patients treated with high-carbohydrate enteral feeds compared with those treated with high-fat enteral feeds."* |
| Criteria for inclusion/ exclusion | **3.1. Do the review’s inclusion-exclusion criteria consider sex-gender differences?** • No, review did not meet criteria *Quote: "Patients must have burns to at least 10% of their total body surface area (TBSA) and have received enteral nutrition. Trials involving patients with any agent of burn injury (thermal, chemical, or electrical) and of any age were eligible. No criteria were set for participant characteristics (age, gender, etc.)."* **3.2. Was there justification or explanation for the exclusion of some groups?** • No, review did not meet criteria |
| Methods | **4.1. Does the review examine whether outcome measures are different for males and females?** • No, review did not meet criteria **4.2. Did the review extract data by sex?** • No, review did not meet criteria **4.3. Did the review extract data on sex of withdrawals and dropouts?** • No, review did not meet criteria **4.4. In cases where sex/gender is used as a proxy for other measures (i.e., weight), is there an explanation for this approach?** • Item was not applicable to the review **4.5. Were any subgroup analyses completed?** • No, review did not meet criteria **4.6. Were subgroup analyses by sex completed?** • No, review did not meet criteria |
| Results and Analysis | **5.1. Do results distinguish between findings for males/females?** • No, review did not meet criteria **5.2. Does the review report conclusions (of effectiveness, efficacy, safety) that are different for men and women?**  • No, review did not meet criteria **5.3. If adverse effects are reported, is information sex disaggregated?** • Item was not applicable to the review **5.4. Does review note that subgroup analyses by sex could not be done?** • Item was not applicable to the review |
| Discussion and conclusion | **6.1. Does the review report that primary studies analysed or failed to analyse results by sex?** • No, review did not meet criteria **6.2. Does the review address sex/gender implications for clinical practice?** • No, review did not meet criteria **6.3. Does the review address sex/gender implications for policy and regulation?** • No, review did not meet criteria **6.4. Does the review address sex/gender implications for research?** • No, review did not meet criteria |
| Table of included studies | **7.1. Does the description of included studies give detailed information on sex/gender of the study samples?** • No, review did not meet criteria (At least 7.1.1. or 7.1.2. are NO) **7.1.1. Detailed information on SEX of the study samples** • No, review did not meet criteria **7.1.2. Detailed information on GENDER of the study samples** • No, review did not meet criteria |
|  |  |
| **Item** | **May (2007)** |
| Background | **1.1 Are the terms sex and gender used in the background?** • Item was not applicable to the review **1.2 Are sex/gender identified as relevant or not to review question?** • Item was not applicable to the review **1.3. Does background discuss why sex/gender differences may be expected?** • Item was not applicable to the review |
| Objectives | **2.1. Are the terms sex, gender, male, or female used in objectives?** • Item was not applicable to the review |
| Criteria for inclusion/ exclusion | **3.1. Do the review’s inclusion-exclusion criteria consider sex-gender differences?** • Item was not applicable to the review **3.2. Was there justification or explanation for the exclusion of some groups?** • Item was not applicable to the review |
| Methods | **4.1. Does the review examine whether outcome measures are different for males and females?** • Item was not applicable to the review **4.2. Did the review extract data by sex?** • Item was not applicable to the review **4.3. Did the review extract data on sex of withdrawals and dropouts?** • Item was not applicable to the review **4.4. In cases where sex/gender is used as a proxy for other measures (i.e., weight), is there an explanation for this approach?** • Item was not applicable to the review **4.5. Were any subgroup analyses completed?** • Item was not applicable to the review **4.6. Were subgroup analyses by sex completed?** • Item was not applicable to the review |
| Results and Analysis | **5.1. Do results distinguish between findings for males/females?** • Item was not applicable to the review **5.2. Does the review report conclusions (of effectiveness, efficacy, safety) that are different for men and women?**  • Item was not applicable to the review **5.3. If adverse effects are reported, is information sex disaggregated?** • Item was not applicable to the review **5.4. Does review note that subgroup analyses by sex could not be done?** • Item was not applicable to the review |
| Discussion and conclusion | **6.1. Does the review report that primary studies analysed or failed to analyse results by sex?** • Item was not applicable to the review **6.2. Does the review address sex/gender implications for clinical practice?** • Item was not applicable to the review **6.3. Does the review address sex/gender implications for policy and regulation?** • Item was not applicable to the review **6.4. Does the review address sex/gender implications for research?** • Item was not applicable to the review |
| Table of included studies | **7.1. Does the description of included studies give detailed information on sex/gender of the study samples?** • Item was not applicable to the review **7.1.1. Detailed information on SEX of the study samples** • Item was not applicable to the review **7.1.2. Detailed information on GENDER of the study samples** • Item was not applicable to the review |
|  |  |
| **Item** | **McCann (2010)** |
| Background | **1.1 Are the terms sex and gender used in the background?** • No, review did not meet criteria **1.2 Are sex/gender identified as relevant or not to review question?** • No, review did not meet criteria **1.3. Does background discuss why sex/gender differences may be expected?** • No, review did not meet criteria |
| Objectives | **2.1. Are the terms sex, gender, male, or female used in objectives?** • No, review did not meet criteria |
| Criteria for inclusion/ exclusion | **3.1. Do the review’s inclusion-exclusion criteria consider sex-gender differences?** • No, review did not meet criteria **3.2. Was there justification or explanation for the exclusion of some groups?** • No, review did not meet criteria |
| Methods | **4.1. Does the review examine whether outcome measures are different for males and females?** • No, review did not meet criteria **4.2. Did the review extract data by sex?** • No, review did not meet criteria **4.3. Did the review extract data on sex of withdrawals and dropouts?** • No, review did not meet criteria **4.4. In cases where sex/gender is used as a proxy for other measures (i.e., weight), is there an explanation for this approach?** • Item was not applicable to the review **4.5. Were any subgroup analyses completed?** • Yes, review met criteria **4.6. Were subgroup analyses by sex completed?** • No, review did not meet criteria |
| Results and Analysis | **5.1. Do results distinguish between findings for males/females?** • No, review did not meet criteria **5.2. Does the review report conclusions (of effectiveness, efficacy, safety) that are different for men and women?**  • No, review did not meet criteria **5.3. If adverse effects are reported, is information sex disaggregated?** • No, review did not meet criteria **5.4. Does review note that subgroup analyses by sex could not be done?** • Item was not applicable to the review |
| Discussion and conclusion | **6.1. Does the review report that primary studies analysed or failed to analyse results by sex?** • No, review did not meet criteria **6.2. Does the review address sex/gender implications for clinical practice?** • No, review did not meet criteria **6.3. Does the review address sex/gender implications for policy and regulation?** • No, review did not meet criteria **6.4. Does the review address sex/gender implications for research?** • No, review did not meet criteria |
| Table of included studies | **7.1. Does the description of included studies give detailed information on sex/gender of the study samples?** • No, review did not meet criteria (At least 7.1.1. or 7.1.2. are NO) **7.1.1. Detailed information on SEX of the study samples** • No, review did not meet criteria **7.1.2. Detailed information on GENDER of the study samples** • No, review did not meet criteria |
|  |  |
| **Item** | **Nabhan (2016)** |
| Background | **1.1 Are the terms sex and gender used in the background?** • Item was not applicable to the review **1.2 Are sex/gender identified as relevant or not to review question?** • Item was not applicable to the review **1.3. Does background discuss why sex/gender differences may be expected?** • Item was not applicable to the review |
| Objectives | **2.1. Are the terms sex, gender, male, or female used in objectives?** • Item was not applicable to the review |
| Criteria for inclusion/ exclusion | **3.1. Do the review’s inclusion-exclusion criteria consider sex-gender differences?** • Item was not applicable to the review **3.2. Was there justification or explanation for the exclusion of some groups?** • Item was not applicable to the review |
| Methods | **4.1. Does the review examine whether outcome measures are different for males and females?** • Item was not applicable to the review **4.2. Did the review extract data by sex?** • Item was not applicable to the review **4.3. Did the review extract data on sex of withdrawals and dropouts?** • Item was not applicable to the review **4.4. In cases where sex/gender is used as a proxy for other measures (i.e., weight), is there an explanation for this approach?** • Item was not applicable to the review **4.5. Were any subgroup analyses completed?** • Item was not applicable to the review **4.6. Were subgroup analyses by sex completed?** • Item was not applicable to the review |
| Results and Analysis | **5.1. Do results distinguish between findings for males/females?** • Item was not applicable to the review **5.2. Does the review report conclusions (of effectiveness, efficacy, safety) that are different for men and women?**  • Item was not applicable to the review **5.3. If adverse effects are reported, is information sex disaggregated?** • Item was not applicable to the review **5.4. Does review note that subgroup analyses by sex could not be done?** • Item was not applicable to the review |
| Discussion and conclusion | **6.1. Does the review report that primary studies analysed or failed to analyse results by sex?** • Item was not applicable to the review **6.2. Does the review address sex/gender implications for clinical practice?** • Item was not applicable to the review **6.3. Does the review address sex/gender implications for policy and regulation?** • Item was not applicable to the review **6.4. Does the review address sex/gender implications for research?** • Item was not applicable to the review |
| Table of included studies | **7.1. Does the description of included studies give detailed information on sex/gender of the study samples?** • Item was not applicable to the review **7.1.1. Detailed information on SEX of the study samples** • Item was not applicable to the review **7.1.2. Detailed information on GENDER of the study samples** • Item was not applicable to the review |
|  |  |
| **Item** | **Nelson (2014)** |
| Background | **1.1 Are the terms sex and gender used in the background?** • No, review did not meet criteria **1.2 Are sex/gender identified as relevant or not to review question?** • No, review did not meet criteria **1.3. Does background discuss why sex/gender differences may be expected?** • No, review did not meet criteria |
| Objectives | **2.1. Are the terms sex, gender, male, or female used in objectives?** • No, review did not meet criteria |
| Criteria for inclusion/ exclusion | **3.1. Do the review’s inclusion-exclusion criteria consider sex-gender differences?** • No, review did not meet criteria *Quote: "Patients (adults and children) undergoing either elective or emergency colorectal surgery. No trial was found that included children."* **3.2. Was there justification or explanation for the exclusion of some groups?** • No, review did not meet criteria |
| Methods | **4.1. Does the review examine whether outcome measures are different for males and females?** • No, review did not meet criteria **4.2. Did the review extract data by sex?** • No, review did not meet criteria **4.3. Did the review extract data on sex of withdrawals and dropouts?** • No, review did not meet criteria **4.4. In cases where sex/gender is used as a proxy for other measures (i.e., weight), is there an explanation for this approach?** • Item was not applicable to the review **4.5. Were any subgroup analyses completed?** • No, review did not meet criteria **4.6. Were subgroup analyses by sex completed?** • No, review did not meet criteria |
| Results and Analysis | **5.1. Do results distinguish between findings for males/females?** • No, review did not meet criteria **5.2. Does the review report conclusions (of effectiveness, efficacy, safety) that are different for men and women?**  • No, review did not meet criteria **5.3. If adverse effects are reported, is information sex disaggregated?** • Item was not applicable to the review **5.4. Does review note that subgroup analyses by sex could not be done?** • Item was not applicable to the review |
| Discussion and conclusion | **6.1. Does the review report that primary studies analysed or failed to analyse results by sex?** • No, review did not meet criteria **6.2. Does the review address sex/gender implications for clinical practice?** • No, review did not meet criteria **6.3. Does the review address sex/gender implications for policy and regulation?** • No, review did not meet criteria **6.4. Does the review address sex/gender implications for research?** • No, review did not meet criteria |
| Table of included studies | **7.1. Does the description of included studies give detailed information on sex/gender of the study samples?** • No, review did not meet criteria (At least 7.1.1. or 7.1.2. are NO) **7.1.1. Detailed information on SEX of the study samples** • No, review did not meet criteria **7.1.2. Detailed information on GENDER of the study samples** • No, review did not meet criteria |
|  |  |
| **Item** | **Niël-Weise (2012)** |
| Background | **1.1 Are the terms sex and gender used in the background?** • No, review did not meet criteria **1.2 Are sex/gender identified as relevant or not to review question?** • No, review did not meet criteria **1.3. Does background discuss why sex/gender differences may be expected?** • No, review did not meet criteria |
| Objectives | **2.1. Are the terms sex, gender, male, or female used in objectives?** • No, review did not meet criteria |
| Criteria for inclusion/ exclusion | **3.1. Do the review’s inclusion-exclusion criteria consider sex-gender differences?** • No, review did not meet criteria *Quote: "All patients requiring long-term catheterisation for urinary incontinence or retention that cannot be managed by another method. This could include people suffering from stress, urge and mixed incontinence, dementia, prostatic hypertrophy unsuitable for other management, stroke, neurological problems, spinal cord injury and spina bifida. They may receive this care at home, in residential homes or in hospital. In this review, long term is defined as more than 14 days."* **3.2. Was there justification or explanation for the exclusion of some groups?** • No, review did not meet criteria |
| Methods | **4.1. Does the review examine whether outcome measures are different for males and females?** • No, review did not meet criteria **4.2. Did the review extract data by sex?** • No, review did not meet criteria **4.3. Did the review extract data on sex of withdrawals and dropouts?** • No, review did not meet criteria **4.4. In cases where sex/gender is used as a proxy for other measures (i.e., weight), is there an explanation for this approach?** • Item was not applicable to the review **4.5. Were any subgroup analyses completed?** • No, review did not meet criteria **4.6. Were subgroup analyses by sex completed?** • No, review did not meet criteria |
| Results and Analysis | **5.1. Do results distinguish between findings for males/females?** • No, review did not meet criteria **5.2. Does the review report conclusions (of effectiveness, efficacy, safety) that are different for men and women?**  • No, review did not meet criteria **5.3. If adverse effects are reported, is information sex disaggregated?** • No, review did not meet criteria **5.4. Does review note that subgroup analyses by sex could not be done?** • Yes, review met criteria |
| Discussion and conclusion | **6.1. Does the review report that primary studies analysed or failed to analyse results by sex?** • No, review did not meet criteria **6.2. Does the review address sex/gender implications for clinical practice?** • No, review did not meet criteria **6.3. Does the review address sex/gender implications for policy and regulation?** • No, review did not meet criteria **6.4. Does the review address sex/gender implications for research?** • No, review did not meet criteria |
| Table of included studies | **7.1. Does the description of included studies give detailed information on sex/gender of the study samples?** • No, review did not meet criteria (At least 7.1.1. or 7.1.2. are NO) **7.1.1. Detailed information on SEX of the study samples** • No, review did not meet criteria **7.1.2. Detailed information on GENDER of the study samples** • No, review did not meet criteria |
|  |  |
| **Item** | **Norman (2016)** |
| Background | **1.1 Are the terms sex and gender used in the background?** • No, review did not meet criteria **1.2 Are sex/gender identified as relevant or not to review question?** • No, review did not meet criteria **1.3. Does background discuss why sex/gender differences may be expected?** • No, review did not meet criteria |
| Objectives | **2.1. Are the terms sex, gender, male, or female used in objectives?** • No, review did not meet criteria |
| Criteria for inclusion/ exclusion | **3.1. Do the review’s inclusion-exclusion criteria consider sex-gender differences?** • No, review did not meet criteria **3.2. Was there justification or explanation for the exclusion of some groups?** • No, review did not meet criteria |
| Methods | **4.1. Does the review examine whether outcome measures are different for males and females?** • No, review did not meet criteria **4.2. Did the review extract data by sex?** • No, review did not meet criteria **4.3. Did the review extract data on sex of withdrawals and dropouts?** • No, review did not meet criteria **4.4. In cases where sex/gender is used as a proxy for other measures (i.e., weight), is there an explanation for this approach?** • Item was not applicable to the review **4.5. Were any subgroup analyses completed?** • No, review did not meet criteria **4.6. Were subgroup analyses by sex completed?** • No, review did not meet criteria |
| Results and Analysis | **5.1. Do results distinguish between findings for males/females?** • No, review did not meet criteria **5.2. Does the review report conclusions (of effectiveness, efficacy, safety) that are different for men and women?**  • No, review did not meet criteria **5.3. If adverse effects are reported, is information sex disaggregated?** • Item was not applicable to the review **5.4. Does review note that subgroup analyses by sex could not be done?** • Item was not applicable to the review |
| Discussion and conclusion | **6.1. Does the review report that primary studies analysed or failed to analyse results by sex?** • No, review did not meet criteria **6.2. Does the review address sex/gender implications for clinical practice?** • No, review did not meet criteria **6.3. Does the review address sex/gender implications for policy and regulation?** • No, review did not meet criteria **6.4. Does the review address sex/gender implications for research?** • No, review did not meet criteria |
| Table of included studies | **7.1. Does the description of included studies give detailed information on sex/gender of the study samples?** • No, review did not meet criteria (At least 7.1.1. or 7.1.2. are NO) **7.1.1. Detailed information on SEX of the study samples** • No, review did not meet criteria **7.1.2. Detailed information on GENDER of the study samples** • No, review did not meet criteria |
|  |  |
| **Item** | **Norman (2016)** |
| Background | **1.1 Are the terms sex and gender used in the background?** • No, review did not meet criteria **1.2 Are sex/gender identified as relevant or not to review question?** • No, review did not meet criteria **1.3. Does background discuss why sex/gender differences may be expected?** • No, review did not meet criteria |
| Objectives | **2.1. Are the terms sex, gender, male, or female used in objectives?** • No, review did not meet criteria |
| Criteria for inclusion/ exclusion | **3.1. Do the review’s inclusion-exclusion criteria consider sex-gender differences?** • No, review did not meet criteria **3.2. Was there justification or explanation for the exclusion of some groups?** • No, review did not meet criteria |
| Methods | **4.1. Does the review examine whether outcome measures are different for males and females?** • No, review did not meet criteria **4.2. Did the review extract data by sex?** • No, review did not meet criteria **4.3. Did the review extract data on sex of withdrawals and dropouts?** • No, review did not meet criteria **4.4. In cases where sex/gender is used as a proxy for other measures (i.e., weight), is there an explanation for this approach?** • Item was not applicable to the review **4.5. Were any subgroup analyses completed?** • No, review did not meet criteria **4.6. Were subgroup analyses by sex completed?** • No, review did not meet criteria |
| Results and Analysis | **5.1. Do results distinguish between findings for males/females?** • No, review did not meet criteria **5.2. Does the review report conclusions (of effectiveness, efficacy, safety) that are different for men and women?**  • No, review did not meet criteria **5.3. If adverse effects are reported, is information sex disaggregated?** • No, review did not meet criteria **5.4. Does review note that subgroup analyses by sex could not be done?** • Item was not applicable to the review |
| Discussion and conclusion | **6.1. Does the review report that primary studies analysed or failed to analyse results by sex?** • No, review did not meet criteria **6.2. Does the review address sex/gender implications for clinical practice?** • No, review did not meet criteria **6.3. Does the review address sex/gender implications for policy and regulation?** • No, review did not meet criteria **6.4. Does the review address sex/gender implications for research?** • No, review did not meet criteria |
| Table of included studies | **7.1. Does the description of included studies give detailed information on sex/gender of the study samples?** • No, review did not meet criteria (At least 7.1.1. or 7.1.2. are NO) *Comment: Sometimes gender used, other sex, and the terminology was never explained.* **7.1.1. Detailed information on SEX of the study samples** • No, review did not meet criteria **7.1.2. Detailed information on GENDER of the study samples** • No, review did not meet criteria |
|  |  |
| **Item** | **Phipps (2006)** |
| Background | **1.1 Are the terms sex and gender used in the background?** • No, review did not meet criteria **1.2 Are sex/gender identified as relevant or not to review question?** • No, review did not meet criteria **1.3. Does background discuss why sex/gender differences may be expected?** • No, review did not meet criteria |
| Objectives | **2.1. Are the terms sex, gender, male, or female used in objectives?** • No, review did not meet criteria |
| Criteria for inclusion/ exclusion | **3.1. Do the review’s inclusion-exclusion criteria consider sex-gender differences?** • No, review did not meet criteria *Quote: "All adults undergoing urogenital surgery. No exclusions based on sex."* **3.2. Was there justification or explanation for the exclusion of some groups?** • No, review did not meet criteria |
| Methods | **4.1. Does the review examine whether outcome measures are different for males and females?** • No, review did not meet criteria **4.2. Did the review extract data by sex?** • No, review did not meet criteria **4.3. Did the review extract data on sex of withdrawals and dropouts?** • No, review did not meet criteria **4.4. In cases where sex/gender is used as a proxy for other measures (i.e., weight), is there an explanation for this approach?** • Item was not applicable to the review **4.5. Were any subgroup analyses completed?** • No, review did not meet criteria **4.6. Were subgroup analyses by sex completed?** • No, review did not meet criteria |
| Results and Analysis | **5.1. Do results distinguish between findings for males/females?** • No, review did not meet criteria **5.2. Does the review report conclusions (of effectiveness, efficacy, safety) that are different for men and women?**  • No, review did not meet criteria **5.3. If adverse effects are reported, is information sex disaggregated?** • No, review did not meet criteria **5.4. Does review note that subgroup analyses by sex could not be done?** • Yes, review met criteria |
| Discussion and conclusion | **6.1. Does the review report that primary studies analysed or failed to analyse results by sex?** • No, review did not meet criteria *Quote: "When comparing urethral versus suprapubic catheters, more women required re-catheterisation following surgery in the urethral catheter group based on the two trials with data (Comparison 02.08). The reasons were not stated but were assumed to be related to failure to void or incontinence."*  **6.2. Does the review address sex/gender implications for clinical practice?** • No, review did not meet criteria **6.3. Does the review address sex/gender implications for policy and regulation?** • No, review did not meet criteria **6.4. Does the review address sex/gender implications for research?** • No, review did not meet criteria |
| Table of included studies | **7.1. Does the description of included studies give detailed information on sex/gender of the study samples?** • No, review did not meet criteria (At least 7.1.1. or 7.1.2. are NO) **7.1.1. Detailed information on SEX of the study samples** • No, review did not meet criteria **7.1.2. Detailed information on GENDER of the study samples** • Unable to determine |
|  |  |
| **Item** | **Prayle (2010)** |
| Background | **1.1 Are the terms sex and gender used in the background?** • No, review did not meet criteria **1.2 Are sex/gender identified as relevant or not to review question?** • No, review did not meet criteria **1.3. Does background discuss why sex/gender differences may be expected?** • No, review did not meet criteria |
| Objectives | **2.1. Are the terms sex, gender, male, or female used in objectives?** • No, review did not meet criteria |
| Criteria for inclusion/ exclusion | **3.1. Do the review’s inclusion-exclusion criteria consider sex-gender differences?** • No, review did not meet criteria **3.2. Was there justification or explanation for the exclusion of some groups?** • No, review did not meet criteria |
| Methods | **4.1. Does the review examine whether outcome measures are different for males and females?** • No, review did not meet criteria **4.2. Did the review extract data by sex?** • No, review did not meet criteria **4.3. Did the review extract data on sex of withdrawals and dropouts?** • No, review did not meet criteria **4.4. In cases where sex/gender is used as a proxy for other measures (i.e., weight), is there an explanation for this approach?** • Item was not applicable to the review **4.5. Were any subgroup analyses completed?** • No, review did not meet criteria **4.6. Were subgroup analyses by sex completed?** • No, review did not meet criteria |
| Results and Analysis | **5.1. Do results distinguish between findings for males/females?** • No, review did not meet criteria **5.2. Does the review report conclusions (of effectiveness, efficacy, safety) that are different for men and women?**  • No, review did not meet criteria **5.3. If adverse effects are reported, is information sex disaggregated?** • No, review did not meet criteria **5.4. Does review note that subgroup analyses by sex could not be done?** • Item was not applicable to the review |
| Discussion and conclusion | **6.1. Does the review report that primary studies analysed or failed to analyse results by sex?** • No, review did not meet criteria **6.2. Does the review address sex/gender implications for clinical practice?** • No, review did not meet criteria **6.3. Does the review address sex/gender implications for policy and regulation?** • No, review did not meet criteria **6.4. Does the review address sex/gender implications for research?** • No, review did not meet criteria |
| Table of included studies | **7.1. Does the description of included studies give detailed information on sex/gender of the study samples?** • No, review did not meet criteria (At least 7.1.1. or 7.1.2. are NO) **7.1.1. Detailed information on SEX of the study samples** • No, review did not meet criteria **7.1.2. Detailed information on GENDER of the study samples** • No, review did not meet criteria |
|  |  |
| **Item** | **Ratilal (2006)** |
| Background | **1.1 Are the terms sex and gender used in the background?** • No, review did not meet criteria **1.2 Are sex/gender identified as relevant or not to review question?** • No, review did not meet criteria **1.3. Does background discuss why sex/gender differences may be expected?** • No, review did not meet criteria |
| Objectives | **2.1. Are the terms sex, gender, male, or female used in objectives?** • No, review did not meet criteria |
| Criteria for inclusion/ exclusion | **3.1. Do the review’s inclusion-exclusion criteria consider sex-gender differences?** • No, review did not meet criteria **3.2. Was there justification or explanation for the exclusion of some groups?** • No, review did not meet criteria |
| Methods | **4.1. Does the review examine whether outcome measures are different for males and females?** • No, review did not meet criteria **4.2. Did the review extract data by sex?** • No, review did not meet criteria **4.3. Did the review extract data on sex of withdrawals and dropouts?** • No, review did not meet criteria **4.4. In cases where sex/gender is used as a proxy for other measures (i.e., weight), is there an explanation for this approach?** • Item was not applicable to the review **4.5. Were any subgroup analyses completed?** • Yes, review met criteria **4.6. Were subgroup analyses by sex completed?** • No, review did not meet criteria |
| Results and Analysis | **5.1. Do results distinguish between findings for males/females?** • No, review did not meet criteria **5.2. Does the review report conclusions (of effectiveness, efficacy, safety) that are different for men and women?**  • No, review did not meet criteria **5.3. If adverse effects are reported, is information sex disaggregated?** • No, review did not meet criteria **5.4. Does review note that subgroup analyses by sex could not be done?** • Item was not applicable to the review |
| Discussion and conclusion | **6.1. Does the review report that primary studies analysed or failed to analyse results by sex?** • No, review did not meet criteria **6.2. Does the review address sex/gender implications for clinical practice?** • No, review did not meet criteria **6.3. Does the review address sex/gender implications for policy and regulation?** • No, review did not meet criteria **6.4. Does the review address sex/gender implications for research?** • No, review did not meet criteria |
| Table of included studies | **7.1. Does the description of included studies give detailed information on sex/gender of the study samples?** • No, review did not meet criteria (At least 7.1.1. or 7.1.2. are NO) **7.1.1. Detailed information on SEX of the study samples** • No, review did not meet criteria **7.1.2. Detailed information on GENDER of the study samples** • No, review did not meet criteria |
|  |  |
| **Item** | **Ray (2014)** |
| Background | **1.1 Are the terms sex and gender used in the background?** • Item was not applicable to the review **1.2 Are sex/gender identified as relevant or not to review question?** • Item was not applicable to the review **1.3. Does background discuss why sex/gender differences may be expected?** • Item was not applicable to the review |
| Objectives | **2.1. Are the terms sex, gender, male, or female used in objectives?** • Item was not applicable to the review |
| Criteria for inclusion/ exclusion | **3.1. Do the review’s inclusion-exclusion criteria consider sex-gender differences?** • Item was not applicable to the review **3.2. Was there justification or explanation for the exclusion of some groups?** • Item was not applicable to the review |
| Methods | **4.1. Does the review examine whether outcome measures are different for males and females?** • Item was not applicable to the review **4.2. Did the review extract data by sex?** • Item was not applicable to the review **4.3. Did the review extract data on sex of withdrawals and dropouts?** • Item was not applicable to the review **4.4. In cases where sex/gender is used as a proxy for other measures (i.e., weight), is there an explanation for this approach?** • Item was not applicable to the review **4.5. Were any subgroup analyses completed?** • Item was not applicable to the review **4.6. Were subgroup analyses by sex completed?** • Item was not applicable to the review |
| Results and Analysis | **5.1. Do results distinguish between findings for males/females?** • Item was not applicable to the review **5.2. Does the review report conclusions (of effectiveness, efficacy, safety) that are different for men and women?**  • Item was not applicable to the review **5.3. If adverse effects are reported, is information sex disaggregated?** • Item was not applicable to the review **5.4. Does review note that subgroup analyses by sex could not be done?** • Item was not applicable to the review |
| Discussion and conclusion | **6.1. Does the review report that primary studies analysed or failed to analyse results by sex?** • Item was not applicable to the review **6.2. Does the review address sex/gender implications for clinical practice?** • No, review did not meet criteria • Item was not applicable to the review **6.3. Does the review address sex/gender implications for policy and regulation?** • Item was not applicable to the review **6.4. Does the review address sex/gender implications for research?** • Item was not applicable to the review |
| Table of included studies | **7.1. Does the description of included studies give detailed information on sex/gender of the study samples?** • Item was not applicable to the review **7.1.1. Detailed information on SEX of the study samples** • Item was not applicable to the review **7.1.2. Detailed information on GENDER of the study samples** • Item was not applicable to the review |
|  |  |
| **Item** | **Robertson-Malt (2014)** |
| Background | **1.1 Are the terms sex and gender used in the background?** • No, review did not meet criteria **1.2 Are sex/gender identified as relevant or not to review question?** • No, review did not meet criteria **1.3. Does background discuss why sex/gender differences may be expected?** • No, review did not meet criteria |
| Objectives | **2.1. Are the terms sex, gender, male, or female used in objectives?** • No, review did not meet criteria |
| Criteria for inclusion/ exclusion | **3.1. Do the review’s inclusion-exclusion criteria consider sex-gender differences?** • No, review did not meet criteria **3.2. Was there justification or explanation for the exclusion of some groups?** • No, review did not meet criteria |
| Methods | **4.1. Does the review examine whether outcome measures are different for males and females?** • No, review did not meet criteria **4.2. Did the review extract data by sex?** • No, review did not meet criteria **4.3. Did the review extract data on sex of withdrawals and dropouts?** • No, review did not meet criteria **4.4. In cases where sex/gender is used as a proxy for other measures (i.e., weight), is there an explanation for this approach?** • Item was not applicable to the review **4.5. Were any subgroup analyses completed?** • No, review did not meet criteria **4.6. Were subgroup analyses by sex completed?** • No, review did not meet criteria |
| Results and Analysis | **5.1. Do results distinguish between findings for males/females?** • No, review did not meet criteria **5.2. Does the review report conclusions (of effectiveness, efficacy, safety) that are different for men and women?**  • No, review did not meet criteria **5.3. If adverse effects are reported, is information sex disaggregated?** • No, review did not meet criteria **5.4. Does review note that subgroup analyses by sex could not be done?** • Item was not applicable to the review |
| Discussion and conclusion | **6.1. Does the review report that primary studies analysed or failed to analyse results by sex?** • No, review did not meet criteria **6.2. Does the review address sex/gender implications for clinical practice?** • No, review did not meet criteria **6.3. Does the review address sex/gender implications for policy and regulation?** • No, review did not meet criteria **6.4. Does the review address sex/gender implications for research?** • No, review did not meet criteria |
| Table of included studies | **7.1. Does the description of included studies give detailed information on sex/gender of the study samples?** • No, review did not meet criteria (At least 7.1.1. or 7.1.2. are NO) **7.1.1. Detailed information on SEX of the study samples** • No, review did not meet criteria **7.1.2. Detailed information on GENDER of the study samples** • No, review did not meet criteria |
|  |  |
| **Item** | **Sajid (2013)** |
| Background | **1.1 Are the terms sex and gender used in the background?** • Yes, review met criteria *Quote: "Breast cancer remains the second most common cancer in women with a reported mortality reaching 460,000 deaths worldwide in 2008 (WHO 2011)."* **1.2 Are sex/gender identified as relevant or not to review question?** • Unable to determine **1.3. Does background discuss why sex/gender differences may be expected?** • No, review did not meet criteria *Quote: "Breast cancer remains the second most common cancer in women with a reported mortality reaching 460,000 deaths worldwide in 2008 (WHO 2011)."* |
| Objectives | **2.1. Are the terms sex, gender, male, or female used in objectives?** • No, review did not meet criteria *Quote: "1. To determine whether the application of FG following breast cancer surgery reduces the incidence of seroma formation. 2. To determine the effect of FG on the total drain volume, mean volume of seroma aspirate, frequency of wound infection or complications and the length of hospital stay."* |
| Criteria for inclusion/ exclusion | **3.1. Do the review’s inclusion-exclusion criteria consider sex-gender differences?** • No, review did not meet criteria *Quote: "We considered the inclusion of trials published in all languages regardless of the number of participants, their age and gender". Quote: "We included trials recruiting people with breast cancer undergoing simple mastectomy, MRM, breast-conserving surgery, oncoplastic breast surgery, lumpectomy, quadrantectomy, axillary sentinel node biopsy, axillary sampling, axillary dissection of any level, and immediate partial or total breast reconstruction."*  **3.2. Was there justification or explanation for the exclusion of some groups?** • No, review did not meet criteria |
| Methods | **4.1. Does the review examine whether outcome measures are different for males and females?** • No, review did not meet criteria **4.2. Did the review extract data by sex?** • No, review did not meet criteria **4.3. Did the review extract data on sex of withdrawals and dropouts?** • No, review did not meet criteria **4.4. In cases where sex/gender is used as a proxy for other measures (i.e., weight), is there an explanation for this approach?** • Item was not applicable to the review **4.5. Were any subgroup analyses completed?** • Yes, review met criteria *Quote: "Based on the duration of follow-up there were insufficient data to perform subgroup analyses. We performed the subgroup analysis on trials in breast surgery and breast plus axillary surgery to find out if there was any difference depending upon the site of surgery for breast cancer."* **4.6. Were subgroup analyses by sex completed?** • No, review did not meet criteria |
| Results and Analysis | **5.1. Do results distinguish between findings for males/females?** • No, review did not meet criteria **5.2. Does the review report conclusions (of effectiveness, efficacy, safety) that are different for men and women?**  • No, review did not meet criteria **5.3. If adverse effects are reported, is information sex disaggregated?** • No, review did not meet criteria **5.4. Does review note that subgroup analyses by sex could not be done?** • Item was not applicable to the review |
| Discussion and conclusion | **6.1. Does the review report that primary studies analysed or failed to analyse results by sex?** • No, review did not meet criteria **6.2. Does the review address sex/gender implications for clinical practice?** • No, review did not meet criteria **6.3. Does the review address sex/gender implications for policy and regulation?** • No, review did not meet criteria **6.4. Does the review address sex/gender implications for research?** • No, review did not meet criteria |
| Table of included studies | **7.1. Does the description of included studies give detailed information on sex/gender of the study samples?** • No, review did not meet criteria (At least 7.1.1. or 7.1.2. are NO) **7.1.1. Detailed information on SEX of the study samples** • No, review did not meet criteria **7.1.2. Detailed information on GENDER of the study samples** • No, review did not meet criteria |
|  |  |
| **Item** | **Sanabria (2010)** |
| Background | **1.1 Are the terms sex and gender used in the background?** • No, review did not meet criteria **1.2 Are sex/gender identified as relevant or not to review question?** • No, review did not meet criteria **1.3. Does background discuss why sex/gender differences may be expected?** • No, review did not meet criteria |
| Objectives | **2.1. Are the terms sex, gender, male, or female used in objectives?** • No, review did not meet criteria *Quote: "To assess the beneficial or harmful effects of antibiotic prophylaxis in patients undergoing elective laparoscopic cholecystectomy."* |
| Criteria for inclusion/ exclusion | **3.1. Do the review’s inclusion-exclusion criteria consider sex-gender differences?** • No, review did not meet criteria *Quote: "Adult patients (more than 17 years old) undergoing laparoscopic cholecystectomy with preoperative clinical diagnosis of cholelithiasis without acute cholecystitis or other benign non-acute inflammatory disease of the gallbladder. Jaundiced patients were excluded."* **3.2. Was there justification or explanation for the exclusion of some groups?** • No, review did not meet criteria |
| Methods | **4.1. Does the review examine whether outcome measures are different for males and females?** • No, review did not meet criteria **4.2. Did the review extract data by sex?** • No, review did not meet criteria **4.3. Did the review extract data on sex of withdrawals and dropouts?** • No, review did not meet criteria **4.4. In cases where sex/gender is used as a proxy for other measures (i.e., weight), is there an explanation for this approach?** • Item was not applicable to the review **4.5. Were any subgroup analyses completed?** • No, review did not meet criteria **4.6. Were subgroup analyses by sex completed?** • No, review did not meet criteria |
| Results and Analysis | **5.1. Do results distinguish between findings for males/females?** • No, review did not meet criteria **5.2. Does the review report conclusions (of effectiveness, efficacy, safety) that are different for men and women?**  • No, review did not meet criteria **5.3. If adverse effects are reported, is information sex disaggregated?** • Item was not applicable to the review **5.4. Does review note that subgroup analyses by sex could not be done?** • Item was not applicable to the review |
| Discussion and conclusion | **6.1. Does the review report that primary studies analysed or failed to analyse results by sex?** • No, review did not meet criteria **6.2. Does the review address sex/gender implications for clinical practice?** • No, review did not meet criteria **6.3. Does the review address sex/gender implications for policy and regulation?** • No, review did not meet criteria **6.4. Does the review address sex/gender implications for research?** • No, review did not meet criteria |
| Table of included studies | **7.1. Does the description of included studies give detailed information on sex/gender of the study samples?** • No, review did not meet criteria (At least 7.1.1. or 7.1.2. are NO) **7.1.1. Detailed information on SEX of the study samples** • Unable to determine **7.1.2. Detailed information on GENDER of the study samples** • No, review did not meet criteria |
|  |  |
| **Item** | **Sanchez-Manuel (2012)** |
| Background | **1.1 Are the terms sex and gender used in the background?** • No, review did not meet criteria **1.2 Are sex/gender identified as relevant or not to review question?** • No, review did not meet criteria **1.3. Does background discuss why sex/gender differences may be expected?** • No, review did not meet criteria |
| Objectives | **2.1. Are the terms sex, gender, male, or female used in objectives?** • No, review did not meet criteria |
| Criteria for inclusion/ exclusion | **3.1. Do the review’s inclusion-exclusion criteria consider sex-gender differences?** • No, review did not meet criteria **3.2. Was there justification or explanation for the exclusion of some groups?** • No, review did not meet criteria |
| Methods | **4.1. Does the review examine whether outcome measures are different for males and females?** • No, review did not meet criteria **4.2. Did the review extract data by sex?** • No, review did not meet criteria **4.3. Did the review extract data on sex of withdrawals and dropouts?** • No, review did not meet criteria **4.4. In cases where sex/gender is used as a proxy for other measures (i.e., weight), is there an explanation for this approach?** • Item was not applicable to the review **4.5. Were any subgroup analyses completed?** • Yes, review met criteria **4.6. Were subgroup analyses by sex completed?** • No, review did not meet criteria |
| Results and Analysis | **5.1. Do results distinguish between findings for males/females?** • No, review did not meet criteria **5.2. Does the review report conclusions (of effectiveness, efficacy, safety) that are different for men and women?**  • No, review did not meet criteria **5.3. If adverse effects are reported, is information sex disaggregated?** • Item was not applicable to the review **5.4. Does review note that subgroup analyses by sex could not be done?** • Item was not applicable to the review |
| Discussion and conclusion | **6.1. Does the review report that primary studies analysed or failed to analyse results by sex?** • No, review did not meet criteria **6.2. Does the review address sex/gender implications for clinical practice?** • No, review did not meet criteria **6.3. Does the review address sex/gender implications for policy and regulation?** • No, review did not meet criteria **6.4. Does the review address sex/gender implications for research?** • No, review did not meet criteria |
| Table of included studies | **7.1. Does the description of included studies give detailed information on sex/gender of the study samples?** • No, review did not meet criteria (At least 7.1.1. or 7.1.2. are NO) **7.1.1. Detailed information on SEX of the study samples** • No, review did not meet criteria **7.1.2. Detailed information on GENDER of the study samples** • No, review did not meet criteria |
|  |  |
| **Item** | **Shah (2014)** |
| Background | **1.1 Are the terms sex and gender used in the background?** • No, review did not meet criteria **1.2 Are sex/gender identified as relevant or not to review question?** • No, review did not meet criteria **1.3. Does background discuss why sex/gender differences may be expected?** • No, review did not meet criteria |
| Objectives | **2.1. Are the terms sex, gender, male, or female used in objectives?** • No, review did not meet criteria |
| Criteria for inclusion/ exclusion | **3.1. Do the review’s inclusion-exclusion criteria consider sex-gender differences?** • No, review did not meet criteria **3.2. Was there justification or explanation for the exclusion of some groups?** • No, review did not meet criteria |
| Methods | **4.1. Does the review examine whether outcome measures are different for males and females?** • No, review did not meet criteria **4.2. Did the review extract data by sex?** • No, review did not meet criteria **4.3. Did the review extract data on sex of withdrawals and dropouts?** • No, review did not meet criteria **4.4. In cases where sex/gender is used as a proxy for other measures (i.e., weight), is there an explanation for this approach?** • Item was not applicable to the review **4.5. Were any subgroup analyses completed?** • No, review did not meet criteria **4.6. Were subgroup analyses by sex completed?** • No, review did not meet criteria |
| Results and Analysis | **5.1. Do results distinguish between findings for males/females?** • No, review did not meet criteria **5.2. Does the review report conclusions (of effectiveness, efficacy, safety) that are different for men and women?**  • No, review did not meet criteria **5.3. If adverse effects are reported, is information sex disaggregated?** • No, review did not meet criteria **5.4. Does review note that subgroup analyses by sex could not be done?** • Item was not applicable to the review |
| Discussion and conclusion | **6.1. Does the review report that primary studies analysed or failed to analyse results by sex?** • No, review did not meet criteria **6.2. Does the review address sex/gender implications for clinical practice?** • No, review did not meet criteria **6.3. Does the review address sex/gender implications for policy and regulation?** • No, review did not meet criteria **6.4. Does the review address sex/gender implications for research?** • No, review did not meet criteria |
| Table of included studies | **7.1. Does the description of included studies give detailed information on sex/gender of the study samples?** • No, review did not meet criteria (At least 7.1.1. or 7.1.2. are NO) **7.1.1. Detailed information on SEX of the study samples** • Unable to determine **7.1.2. Detailed information on GENDER of the study samples** • No, review did not meet criteria |
|  |  |
| **Item** | **Smaill (2014)** |
| Background | **1.1 Are the terms sex and gender used in the background?** • Item was not applicable to the review **1.2 Are sex/gender identified as relevant or not to review question?** • Item was not applicable to the review **1.3. Does background discuss why sex/gender differences may be expected?** • Item was not applicable to the review |
| Objectives | **2.1. Are the terms sex, gender, male, or female used in objectives?** • Item was not applicable to the review |
| Criteria for inclusion/ exclusion | **3.1. Do the review’s inclusion-exclusion criteria consider sex-gender differences?** • Item was not applicable to the review **3.2. Was there justification or explanation for the exclusion of some groups?** • Item was not applicable to the review |
| Methods | **4.1. Does the review examine whether outcome measures are different for males and females?** • Item was not applicable to the review **4.2. Did the review extract data by sex?** • Item was not applicable to the review **4.3. Did the review extract data on sex of withdrawals and dropouts?** • Item was not applicable to the review **4.4. In cases where sex/gender is used as a proxy for other measures (i.e., weight), is there an explanation for this approach?** • Item was not applicable to the review **4.5. Were any subgroup analyses completed?** • Item was not applicable to the review **4.6. Were subgroup analyses by sex completed?** • Item was not applicable to the review |
| Results and Analysis | **5.1. Do results distinguish between findings for males/females?** • Item was not applicable to the review **5.2. Does the review report conclusions (of effectiveness, efficacy, safety) that are different for men and women?**  • Item was not applicable to the review **5.3. If adverse effects are reported, is information sex disaggregated?** • Item was not applicable to the review **5.4. Does review note that subgroup analyses by sex could not be done?** • Item was not applicable to the review |
| Discussion and conclusion | **6.1. Does the review report that primary studies analysed or failed to analyse results by sex?** • Item was not applicable to the review **6.2. Does the review address sex/gender implications for clinical practice?** • Item was not applicable to the review **6.3. Does the review address sex/gender implications for policy and regulation?** • Item was not applicable to the review **6.4. Does the review address sex/gender implications for research?** • Item was not applicable to the review |
| Table of included studies | **7.1. Does the description of included studies give detailed information on sex/gender of the study samples?** • Item was not applicable to the review **7.1.1. Detailed information on SEX of the study samples** • Item was not applicable to the review **7.1.2. Detailed information on GENDER of the study samples** • Item was not applicable to the review |
|  |  |
| **Item** | **Stewart (2006)** |
| Background | **1.1 Are the terms sex and gender used in the background?** • No, review did not meet criteria **1.2 Are sex/gender identified as relevant or not to review question?** • No, review did not meet criteria **1.3. Does background discuss why sex/gender differences may be expected?** • No, review did not meet criteria |
| Objectives | **2.1. Are the terms sex, gender, male, or female used in objectives?** • No, review did not meet criteria |
| Criteria for inclusion/ exclusion | **3.1. Do the review’s inclusion-exclusion criteria consider sex-gender differences?** • No, review did not meet criteria **3.2. Was there justification or explanation for the exclusion of some groups?** • No, review did not meet criteria |
| Methods | **4.1. Does the review examine whether outcome measures are different for males and females?** • No, review did not meet criteria **4.2. Did the review extract data by sex?** • No, review did not meet criteria **4.3. Did the review extract data on sex of withdrawals and dropouts?** • No, review did not meet criteria **4.4. In cases where sex/gender is used as a proxy for other measures (i.e., weight), is there an explanation for this approach?** • Item was not applicable to the review **4.5. Were any subgroup analyses completed?** • No, review did not meet criteria **4.6. Were subgroup analyses by sex completed?** • No, review did not meet criteria |
| Results and Analysis | **5.1. Do results distinguish between findings for males/females?** • No, review did not meet criteria **5.2. Does the review report conclusions (of effectiveness, efficacy, safety) that are different for men and women?**  • No, review did not meet criteria **5.3. If adverse effects are reported, is information sex disaggregated?** • Item was not applicable to the review **5.4. Does review note that subgroup analyses by sex could not be done?** • Item was not applicable to the review |
| Discussion and conclusion | **6.1. Does the review report that primary studies analysed or failed to analyse results by sex?** • No, review did not meet criteria **6.2. Does the review address sex/gender implications for clinical practice?** • No, review did not meet criteria **6.3. Does the review address sex/gender implications for policy and regulation?** • No, review did not meet criteria **6.4. Does the review address sex/gender implications for research?** • No, review did not meet criteria |
| Table of included studies | **7.1. Does the description of included studies give detailed information on sex/gender of the study samples?** • No, review did not meet criteria (At least 7.1.1. or 7.1.2. are NO) **7.1.1. Detailed information on SEX of the study samples** • Unable to determine **7.1.2. Detailed information on GENDER of the study samples** • No, review did not meet criteria |
|  |  |
| **Item** | **Storm-Versloot (2010)** |
| Background | **1.1 Are the terms sex and gender used in the background?** • No, review did not meet criteria **1.2 Are sex/gender identified as relevant or not to review question?** • No, review did not meet criteria **1.3. Does background discuss why sex/gender differences may be expected?** • No, review did not meet criteria |
| Objectives | **2.1. Are the terms sex, gender, male, or female used in objectives?** • No, review did not meet criteria *Quote: "To summarise the evidence for the effects of silver-containing dressings and topical agents compared with non-silver dressings and topical agents in terms of preventing of wound infections and or promoting wound healing."* |
| Criteria for inclusion/ exclusion | **3.1. Do the review’s inclusion-exclusion criteria consider sex-gender differences?** • No, review did not meet criteria *Quote: "Men and women aged 18 years and over with any type of wound (not diagnosed as infected at baseline) in any care setting."* **3.2. Was there justification or explanation for the exclusion of some groups?** • No, review did not meet criteria |
| Methods | **4.1. Does the review examine whether outcome measures are different for males and females?** • No, review did not meet criteria **4.2. Did the review extract data by sex?** • No, review did not meet criteria **4.3. Did the review extract data on sex of withdrawals and dropouts?** • No, review did not meet criteria **4.4. In cases where sex/gender is used as a proxy for other measures (i.e., weight), is there an explanation for this approach?** • Item was not applicable to the review **4.5. Were any subgroup analyses completed?** • Yes, review met criteria *Quote: "We conducted pre-specified subgroup analyses for different wound types: burns, acute (e.g. surgical), chronic (e.g. ulcers) and mixed wound types".* **4.6. Were subgroup analyses by sex completed?** • No, review did not meet criteria |
| Results and Analysis | **5.1. Do results distinguish between findings for males/females?** • No, review did not meet criteria **5.2. Does the review report conclusions (of effectiveness, efficacy, safety) that are different for men and women?**  • No, review did not meet criteria **5.3. If adverse effects are reported, is information sex disaggregated?** • No, review did not meet criteria **5.4. Does review note that subgroup analyses by sex could not be done?** • Item was not applicable to the review |
| Discussion and conclusion | **6.1. Does the review report that primary studies analysed or failed to analyse results by sex?** • No, review did not meet criteria **6.2. Does the review address sex/gender implications for clinical practice?** • No, review did not meet criteria **6.3. Does the review address sex/gender implications for policy and regulation?** • No, review did not meet criteria **6.4. Does the review address sex/gender implications for research?** • No, review did not meet criteria |
| Table of included studies | **7.1. Does the description of included studies give detailed information on sex/gender of the study samples?** • No, review did not meet criteria (At least 7.1.1. or 7.1.2. are NO) **7.1.1. Detailed information on SEX of the study samples** • No, review did not meet criteria **7.1.2. Detailed information on GENDER of the study samples** • No, review did not meet criteria |
|  |  |
| **Item** | **Strippoli (2004)** |
| Background | **1.1 Are the terms sex and gender used in the background?** • No, review did not meet criteria **1.2 Are sex/gender identified as relevant or not to review question?** • No, review did not meet criteria **1.3. Does background discuss why sex/gender differences may be expected?** • No, review did not meet criteria |
| Objectives | **2.1. Are the terms sex, gender, male, or female used in objectives?** • No, review did not meet criteria |
| Criteria for inclusion/ exclusion | **3.1. Do the review’s inclusion-exclusion criteria consider sex-gender differences?** • No, review did not meet criteria *Quote: "Adult and paediatric patients undergoing PD treatment."* **3.2. Was there justification or explanation for the exclusion of some groups?** • No, review did not meet criteria |
| Methods | **4.1. Does the review examine whether outcome measures are different for males and females?** • No, review did not meet criteria **4.2. Did the review extract data by sex?** • No, review did not meet criteria **4.3. Did the review extract data on sex of withdrawals and dropouts?** • No, review did not meet criteria **4.4. In cases where sex/gender is used as a proxy for other measures (i.e., weight), is there an explanation for this approach?** • Item was not applicable to the review **4.5. Were any subgroup analyses completed?** • No, review did not meet criteria **4.6. Were subgroup analyses by sex completed?** • No, review did not meet criteria |
| Results and Analysis | **5.1. Do results distinguish between findings for males/females?** • No, review did not meet criteria **5.2. Does the review report conclusions (of effectiveness, efficacy, safety) that are different for men and women?**  • No, review did not meet criteria **5.3. If adverse effects are reported, is information sex disaggregated?** • Item was not applicable to the review **5.4. Does review note that subgroup analyses by sex could not be done?** • Item was not applicable to the review |
| Discussion and conclusion | **6.1. Does the review report that primary studies analysed or failed to analyse results by sex?** • No, review did not meet criteria **6.2. Does the review address sex/gender implications for clinical practice?** • No, review did not meet criteria **6.3. Does the review address sex/gender implications for policy and regulation?** • No, review did not meet criteria **6.4. Does the review address sex/gender implications for research?** • No, review did not meet criteria |
| Table of included studies | **7.1. Does the description of included studies give detailed information on sex/gender of the study samples?** • No, review did not meet criteria (At least 7.1.1. or 7.1.2. are NO) **7.1.1. Detailed information on SEX of the study samples** • No, review did not meet criteria **7.1.2. Detailed information on GENDER of the study samples** • No, review did not meet criteria |
|  |  |
| **Item** | **Strippoli (2004)** |
| Background | **1.1 Are the terms sex and gender used in the background?** • No, review did not meet criteria **1.2 Are sex/gender identified as relevant or not to review question?** • No, review did not meet criteria **1.3. Does background discuss why sex/gender differences may be expected?** • No, review did not meet criteria |
| Objectives | **2.1. Are the terms sex, gender, male, or female used in objectives?** • No, review did not meet criteria *Quote: "To evaluate the benefits and harms of antimicrobial strategies used to prevent peritonitis in PD patients".* |
| Criteria for inclusion/ exclusion | **3.1. Do the review’s inclusion-exclusion criteria consider sex-gender differences?** • No, review did not meet criteria *Quote: "We included adults and children with ESKD who were undergoing PD treatment."* **3.2. Was there justification or explanation for the exclusion of some groups?** • No, review did not meet criteria |
| Methods | **4.1. Does the review examine whether outcome measures are different for males and females?** • No, review did not meet criteria **4.2. Did the review extract data by sex?** • No, review did not meet criteria **4.3. Did the review extract data on sex of withdrawals and dropouts?** • No, review did not meet criteria **4.4. In cases where sex/gender is used as a proxy for other measures (i.e., weight), is there an explanation for this approach?** • Item was not applicable to the review **4.5. Were any subgroup analyses completed?** • No, review did not meet criteria **4.6. Were subgroup analyses by sex completed?** • No, review did not meet criteria |
| Results and Analysis | **5.1. Do results distinguish between findings for males/females?** • No, review did not meet criteria **5.2. Does the review report conclusions (of effectiveness, efficacy, safety) that are different for men and women?**  • No, review did not meet criteria **5.3. If adverse effects are reported, is information sex disaggregated?** • No, review did not meet criteria **5.4. Does review note that subgroup analyses by sex could not be done?** • Item was not applicable to the review |
| Discussion and conclusion | **6.1. Does the review report that primary studies analysed or failed to analyse results by sex?** • No, review did not meet criteria **6.2. Does the review address sex/gender implications for clinical practice?** • No, review did not meet criteria **6.3. Does the review address sex/gender implications for policy and regulation?** • No, review did not meet criteria **6.4. Does the review address sex/gender implications for research?** • No, review did not meet criteria |
| Table of included studies | **7.1. Does the description of included studies give detailed information on sex/gender of the study samples?** • No, review did not meet criteria (At least 7.1.1. or 7.1.2. are NO) **7.1.1. Detailed information on SEX of the study samples** • Unable to determine **7.1.2. Detailed information on GENDER of the study samples** • No, review did not meet criteria |
|  |  |
| **Item** | **Subirana (2007)** |
| Background | **1.1 Are the terms sex and gender used in the background?** • No, review did not meet criteria **1.2 Are sex/gender identified as relevant or not to review question?** • No, review did not meet criteria **1.3. Does background discuss why sex/gender differences may be expected?** • No, review did not meet criteria |
| Objectives | **2.1. Are the terms sex, gender, male, or female used in objectives?** • No, review did not meet criteria |
| Criteria for inclusion/ exclusion | **3.1. Do the review’s inclusion-exclusion criteria consider sex-gender differences?** • No, review did not meet criteria **3.2. Was there justification or explanation for the exclusion of some groups?** • No, review did not meet criteria |
| Methods | **4.1. Does the review examine whether outcome measures are different for males and females?** • No, review did not meet criteria **4.2. Did the review extract data by sex?** • No, review did not meet criteria **4.3. Did the review extract data on sex of withdrawals and dropouts?** • No, review did not meet criteria **4.4. In cases where sex/gender is used as a proxy for other measures (i.e., weight), is there an explanation for this approach?** • Item was not applicable to the review **4.5. Were any subgroup analyses completed?** • No, review did not meet criteria **4.6. Were subgroup analyses by sex completed?** • No, review did not meet criteria |
| Results and Analysis | **5.1. Do results distinguish between findings for males/females?** • No, review did not meet criteria **5.2. Does the review report conclusions (of effectiveness, efficacy, safety) that are different for men and women?**  • No, review did not meet criteria **5.3. If adverse effects are reported, is information sex disaggregated?** • Item was not applicable to the review **5.4. Does review note that subgroup analyses by sex could not be done?** • Item was not applicable to the review |
| Discussion and conclusion | **6.1. Does the review report that primary studies analysed or failed to analyse results by sex?** • No, review did not meet criteria **6.2. Does the review address sex/gender implications for clinical practice?** • No, review did not meet criteria **6.3. Does the review address sex/gender implications for policy and regulation?** • No, review did not meet criteria **6.4. Does the review address sex/gender implications for research?** • No, review did not meet criteria |
| Table of included studies | **7.1. Does the description of included studies give detailed information on sex/gender of the study samples?** • No, review did not meet criteria (At least 7.1.1. or 7.1.2. are NO) **7.1.1. Detailed information on SEX of the study samples** • Unable to determine **7.1.2. Detailed information on GENDER of the study samples** • No, review did not meet criteria |
|  |  |
| **Item** | **Syed (2013)** |
| Background | **1.1 Are the terms sex and gender used in the background?** • No, review did not meet criteria **1.2 Are sex/gender identified as relevant or not to review question?** • No, review did not meet criteria **1.3. Does background discuss why sex/gender differences may be expected?** • No, review did not meet criteria |
| Objectives | **2.1. Are the terms sex, gender, male, or female used in objectives?** • No, review did not meet criteria |
| Criteria for inclusion/ exclusion | **3.1. Do the review’s inclusion-exclusion criteria consider sex-gender differences?** • No, review did not meet criteria *Quote: "Children between the ages of three months and 17 years who had a grommet inserted for otitis media with effusion and/or recurrent acute otitis media".* **3.2. Was there justification or explanation for the exclusion of some groups?** • No, review did not meet criteria |
| Methods | **4.1. Does the review examine whether outcome measures are different for males and females?** • No, review did not meet criteria **4.2. Did the review extract data by sex?** • No, review did not meet criteria **4.3. Did the review extract data on sex of withdrawals and dropouts?** • No, review did not meet criteria **4.4. In cases where sex/gender is used as a proxy for other measures (i.e., weight), is there an explanation for this approach?** • Item was not applicable to the review **4.5. Were any subgroup analyses completed?** • Yes, review met criteria *Quote: "Where relevant we conducted separate subgroup analyses for those trials considered to be at high or low risk of bias. We did not find enough trials to be able to conduct subgroup analyses for age, sex, indication of ventilation tube insertion or type of effusion at time of surgery."* **4.6. Were subgroup analyses by sex completed?** • No, review did not meet criteria |
| Results and Analysis | **5.1. Do results distinguish between findings for males/females?** • No, review did not meet criteria **5.2. Does the review report conclusions (of effectiveness, efficacy, safety) that are different for men and women?**  • No, review did not meet criteria **5.3. If adverse effects are reported, is information sex disaggregated?** • No, review did not meet criteria **5.4. Does review note that subgroup analyses by sex could not be done?** • Yes, review met criteria |
| Discussion and conclusion | **6.1. Does the review report that primary studies analysed or failed to analyse results by sex?** • No, review did not meet criteria **6.2. Does the review address sex/gender implications for clinical practice?** • No, review did not meet criteria **6.3. Does the review address sex/gender implications for policy and regulation?** • No, review did not meet criteria **6.4. Does the review address sex/gender implications for research?** • No, review did not meet criteria |
| Table of included studies | **7.1. Does the description of included studies give detailed information on sex/gender of the study samples?** • No, review did not meet criteria (At least 7.1.1. or 7.1.2. are NO) **7.1.1. Detailed information on SEX of the study samples** • Unable to determine **7.1.2. Detailed information on GENDER of the study samples** • No, review did not meet criteria |
|  |  |
| **Item** | **Tanner (2006)** |
| Background | **1.1 Are the terms sex and gender used in the background?** • No, review did not meet criteria **1.2 Are sex/gender identified as relevant or not to review question?** • No, review did not meet criteria **1.3. Does background discuss why sex/gender differences may be expected?** • No, review did not meet criteria |
| Objectives | **2.1. Are the terms sex, gender, male, or female used in objectives?** • No, review did not meet criteria |
| Criteria for inclusion/ exclusion | **3.1. Do the review’s inclusion-exclusion criteria consider sex-gender differences?** • No, review did not meet criteria *Quote: "All members of the surgical team practicing in a designated surgical theatre, in any surgical speciality, in any country. This includes first surgeon, second or assistant surgeon and scrub staff."* **3.2. Was there justification or explanation for the exclusion of some groups?** • No, review did not meet criteria |
| Methods | **4.1. Does the review examine whether outcome measures are different for males and females?** • No, review did not meet criteria **4.2. Did the review extract data by sex?** • No, review did not meet criteria **4.3. Did the review extract data on sex of withdrawals and dropouts?** • No, review did not meet criteria **4.4. In cases where sex/gender is used as a proxy for other measures (i.e., weight), is there an explanation for this approach?** • Item was not applicable to the review **4.5. Were any subgroup analyses completed?** • Yes, review met criteria **4.6. Were subgroup analyses by sex completed?** • No, review did not meet criteria |
| Results and Analysis | **5.1. Do results distinguish between findings for males/females?** • No, review did not meet criteria **5.2. Does the review report conclusions (of effectiveness, efficacy, safety) that are different for men and women?**  • No, review did not meet criteria **5.3. If adverse effects are reported, is information sex disaggregated?** • Item was not applicable to the review **5.4. Does review note that subgroup analyses by sex could not be done?** • No, review did not meet criteria |
| Discussion and conclusion | **6.1. Does the review report that primary studies analysed or failed to analyse results by sex?** • No, review did not meet criteria **6.2. Does the review address sex/gender implications for clinical practice?** • No, review did not meet criteria **6.3. Does the review address sex/gender implications for policy and regulation?** • No, review did not meet criteria **6.4. Does the review address sex/gender implications for research?** • No, review did not meet criteria |
| Table of included studies | **7.1. Does the description of included studies give detailed information on sex/gender of the study samples?** • No, review did not meet criteria (At least 7.1.1. or 7.1.2. are NO) **7.1.1. Detailed information on SEX of the study samples** • No, review did not meet criteria **7.1.2. Detailed information on GENDER of the study samples** • No, review did not meet criteria |
|  |  |
| **Item** | **Tanner (2011)** |
| Background | **1.1 Are the terms sex and gender used in the background?** • No, review did not meet criteria **1.2 Are sex/gender identified as relevant or not to review question?** • No, review did not meet criteria **1.3. Does background discuss why sex/gender differences may be expected?** • No, review did not meet criteria |
| Objectives | **2.1. Are the terms sex, gender, male, or female used in objectives?** • No, review did not meet criteria |
| Criteria for inclusion/ exclusion | **3.1. Do the review’s inclusion-exclusion criteria consider sex-gender differences?** • No, review did not meet criteria *Quote: "Adult patients undergoing surgery in a designated operating theatre. It was anticipated that, where appropriate, studies would be grouped and analysed by type of surgery anatomical site of surgery."* **3.2. Was there justification or explanation for the exclusion of some groups?** • No, review did not meet criteria |
| Methods | **4.1. Does the review examine whether outcome measures are different for males and females?** • No, review did not meet criteria **4.2. Did the review extract data by sex?** • No, review did not meet criteria **4.3. Did the review extract data on sex of withdrawals and dropouts?** • No, review did not meet criteria **4.4. In cases where sex/gender is used as a proxy for other measures (i.e., weight), is there an explanation for this approach?** • Item was not applicable to the review **4.5. Were any subgroup analyses completed?** • No, review did not meet criteria **4.6. Were subgroup analyses by sex completed?** • No, review did not meet criteria |
| Results and Analysis | **5.1. Do results distinguish between findings for males/females?** • No, review did not meet criteria **5.2. Does the review report conclusions (of effectiveness, efficacy, safety) that are different for men and women?**  • No, review did not meet criteria **5.3. If adverse effects are reported, is information sex disaggregated?** • Item was not applicable to the review **5.4. Does review note that subgroup analyses by sex could not be done?** • Item was not applicable to the review |
| Discussion and conclusion | **6.1. Does the review report that primary studies analysed or failed to analyse results by sex?** • No, review did not meet criteria **6.2. Does the review address sex/gender implications for clinical practice?** • No, review did not meet criteria **6.3. Does the review address sex/gender implications for policy and regulation?** • No, review did not meet criteria **6.4. Does the review address sex/gender implications for research?** • No, review did not meet criteria |
| Table of included studies | **7.1. Does the description of included studies give detailed information on sex/gender of the study samples?** • No, review did not meet criteria (At least 7.1.1. or 7.1.2. are NO) **7.1.1. Detailed information on SEX of the study samples** • No, review did not meet criteria **7.1.2. Detailed information on GENDER of the study samples** • No, review did not meet criteria |
|  |  |
| **Item** | **Tanner (2016)** |
| Background | **1.1 Are the terms sex and gender used in the background?** • No, review did not meet criteria **1.2 Are sex/gender identified as relevant or not to review question?** • No, review did not meet criteria **1.3. Does background discuss why sex/gender differences may be expected?** • No, review did not meet criteria |
| Objectives | **2.1. Are the terms sex, gender, male, or female used in objectives?** • No, review did not meet criteria |
| Criteria for inclusion/ exclusion | **3.1. Do the review’s inclusion-exclusion criteria consider sex-gender differences?** • No, review did not meet criteria *Quote: "All members of the scrub team or personnel working within the operating theatre or day case setting. The SSI outcome is measured in participants who have undergone surgery."* **3.2. Was there justification or explanation for the exclusion of some groups?** • No, review did not meet criteria |
| Methods | **4.1. Does the review examine whether outcome measures are different for males and females?** • No, review did not meet criteria **4.2. Did the review extract data by sex?** • No, review did not meet criteria **4.3. Did the review extract data on sex of withdrawals and dropouts?** • No, review did not meet criteria **4.4. In cases where sex/gender is used as a proxy for other measures (i.e., weight), is there an explanation for this approach?** • Item was not applicable to the review **4.5. Were any subgroup analyses completed?** • No, review did not meet criteria **4.6. Were subgroup analyses by sex completed?** • No, review did not meet criteria |
| Results and Analysis | **5.1. Do results distinguish between findings for males/females?** • No, review did not meet criteria **5.2. Does the review report conclusions (of effectiveness, efficacy, safety) that are different for men and women?**  • No, review did not meet criteria **5.3. If adverse effects are reported, is information sex disaggregated?** • Item was not applicable to the review **5.4. Does review note that subgroup analyses by sex could not be done?** • Item was not applicable to the review |
| Discussion and conclusion | **6.1. Does the review report that primary studies analysed or failed to analyse results by sex?** • No, review did not meet criteria **6.2. Does the review address sex/gender implications for clinical practice?** • No, review did not meet criteria **6.3. Does the review address sex/gender implications for policy and regulation?** • No, review did not meet criteria **6.4. Does the review address sex/gender implications for research?** • No, review did not meet criteria |
| Table of included studies | **7.1. Does the description of included studies give detailed information on sex/gender of the study samples?** • No, review did not meet criteria (At least 7.1.1. or 7.1.2. are NO) **7.1.1. Detailed information on SEX of the study samples** • No, review did not meet criteria **7.1.2. Detailed information on GENDER of the study samples** • No, review did not meet criteria |
|  |  |
| **Item** | **Thomas (2016)** |
| Background | **1.1 Are the terms sex and gender used in the background?** • No, review did not meet criteria **1.2 Are sex/gender identified as relevant or not to review question?** • No, review did not meet criteria **1.3. Does background discuss why sex/gender differences may be expected?** • No, review did not meet criteria |
| Objectives | **2.1. Are the terms sex, gender, male, or female used in objectives?** • No, review did not meet criteria *Quote: "To identify all randomised controlled trials (RCTs) and non-RCTs assessing the effects of vaccinating healthcare workers on the incidence of laboratory-proven influenza, pneumonia, death from pneumonia and admission to hospital for respiratory illness and death from all causes in those aged 60 years or older resident in long-term care institutions (LTCIs)."* |
| Criteria for inclusion/ exclusion | **3.1. Do the review’s inclusion-exclusion criteria consider sex-gender differences?** • No, review did not meet criteria *Quote: "Healthcare workers (nurses, doctors, nursing and medical students, other health professionals, cleaners, porters and volunteers who have regular contact with those aged 60 years or older) of all ages, caring for those aged 60 years or older in institutions such as nursing homes, LTCIs or hospital wards."* **3.2. Was there justification or explanation for the exclusion of some groups?** • No, review did not meet criteria |
| Methods | **4.1. Does the review examine whether outcome measures are different for males and females?** • No, review did not meet criteria **4.2. Did the review extract data by sex?** • No, review did not meet criteria **4.3. Did the review extract data on sex of withdrawals and dropouts?** • No, review did not meet criteria **4.4. In cases where sex/gender is used as a proxy for other measures (i.e., weight), is there an explanation for this approach?** • Item was not applicable to the review **4.5. Were any subgroup analyses completed?** • Yes, review met criteria *Comment: see, for example, Analysis 1.1.* **4.6. Were subgroup analyses by sex completed?** • No, review did not meet criteria |
| Results and Analysis | **5.1. Do results distinguish between findings for males/females?** • No, review did not meet criteria **5.2. Does the review report conclusions (of effectiveness, efficacy, safety) that are different for men and women?**  • No, review did not meet criteria **5.3. If adverse effects are reported, is information sex disaggregated?** • Item was not applicable to the review **5.4. Does review note that subgroup analyses by sex could not be done?** • Item was not applicable to the review |
| Discussion and conclusion | **6.1. Does the review report that primary studies analysed or failed to analyse results by sex?** • No, review did not meet criteria **6.2. Does the review address sex/gender implications for clinical practice?** • No, review did not meet criteria **6.3. Does the review address sex/gender implications for policy and regulation?** • No, review did not meet criteria **6.4. Does the review address sex/gender implications for research?** • No, review did not meet criteria |
| Table of included studies | **7.1. Does the description of included studies give detailed information on sex/gender of the study samples?** • No, review did not meet criteria (At least 7.1.1. or 7.1.2. are NO) **7.1.1. Detailed information on SEX of the study samples** • No, review did not meet criteria **7.1.2. Detailed information on GENDER of the study samples** • Unable to determine |
|  |  |
| **Item** | **Tokmaji (2015)** |
| Background | **1.1 Are the terms sex and gender used in the background?** • No, review did not meet criteria **1.2 Are sex/gender identified as relevant or not to review question?** • No, review did not meet criteria **1.3. Does background discuss why sex/gender differences may be expected?** • No, review did not meet criteria |
| Objectives | **2.1. Are the terms sex, gender, male, or female used in objectives?** • No, review did not meet criteria |
| Criteria for inclusion/ exclusion | **3.1. Do the review’s inclusion-exclusion criteria consider sex-gender differences?** • No, review did not meet criteria *Quote: "We included studies that investigated intubated and mechanically ventilated critically ill participants. Due to the variety of criteria for admission to adult ICUs, we used authors’ definitions of critically ill. We excluded studies investigating children under 16 years and participants who were re-intubated. Where possible, we only included participants who were intubated for 24 hours or longer."* **3.2. Was there justification or explanation for the exclusion of some groups?** • No, review did not meet criteria |
| Methods | **4.1. Does the review examine whether outcome measures are different for males and females?** • No, review did not meet criteria **4.2. Did the review extract data by sex?** • No, review did not meet criteria **4.3. Did the review extract data on sex of withdrawals and dropouts?** • No, review did not meet criteria **4.4. In cases where sex/gender is used as a proxy for other measures (i.e., weight), is there an explanation for this approach?** • Item was not applicable to the review **4.5. Were any subgroup analyses completed?** • No, review did not meet criteria **4.6. Were subgroup analyses by sex completed?** • No, review did not meet criteria |
| Results and Analysis | **5.1. Do results distinguish between findings for males/females?** • No, review did not meet criteria **5.2. Does the review report conclusions (of effectiveness, efficacy, safety) that are different for men and women?**  • No, review did not meet criteria **5.3. If adverse effects are reported, is information sex disaggregated?** • No, review did not meet criteria **5.4. Does review note that subgroup analyses by sex could not be done?** • Item was not applicable to the review |
| Discussion and conclusion | **6.1. Does the review report that primary studies analysed or failed to analyse results by sex?** • No, review did not meet criteria **6.2. Does the review address sex/gender implications for clinical practice?** • No, review did not meet criteria **6.3. Does the review address sex/gender implications for policy and regulation?** • No, review did not meet criteria **6.4. Does the review address sex/gender implications for research?** • No, review did not meet criteria |
| Table of included studies | **7.1. Does the description of included studies give detailed information on sex/gender of the study samples?** • No, review did not meet criteria (At least 7.1.1. or 7.1.2. are NO) **7.1.1. Detailed information on SEX of the study samples** • No, review did not meet criteria **7.1.2. Detailed information on GENDER of the study samples** • No, review did not meet criteria |
|  |  |
| **Item** | **Toon (2015)** |
| Background | **1.1 Are the terms sex and gender used in the background?** • No, review did not meet criteria **1.2 Are sex/gender identified as relevant or not to review question?** • No, review did not meet criteria **1.3. Does background discuss why sex/gender differences may be expected?** • No, review did not meet criteria |
| Objectives | **2.1. Are the terms sex, gender, male, or female used in objectives?** • No, review did not meet criteria *Quote: "To evaluate the benefits and risks of removing a dressing covering a closed surgical incision site within 48 hours (early dressing removal) or beyond 48 hours (delayed dressing removal) of surgery, on surgical site infection."* |
| Criteria for inclusion/ exclusion | **3.1. Do the review’s inclusion-exclusion criteria consider sex-gender differences?** • No, review did not meet criteria *Quote: "People, of any age and sex, undergoing a surgical procedure (major, minor or day-case procedure) who had their wound closed (primary wound closure), irrespective of the material and method used for the primary closure and the location of the wound."* **3.2. Was there justification or explanation for the exclusion of some groups?** • No, review did not meet criteria |
| Methods | **4.1. Does the review examine whether outcome measures are different for males and females?** • No, review did not meet criteria **4.2. Did the review extract data by sex?** • No, review did not meet criteria **4.3. Did the review extract data on sex of withdrawals and dropouts?** • No, review did not meet criteria **4.4. In cases where sex/gender is used as a proxy for other measures (i.e., weight), is there an explanation for this approach?** • Item was not applicable to the review **4.5. Were any subgroup analyses completed?** • No, review did not meet criteria **4.6. Were subgroup analyses by sex completed?** • No, review did not meet criteria |
| Results and Analysis | **5.1. Do results distinguish between findings for males/females?** • No, review did not meet criteria **5.2. Does the review report conclusions (of effectiveness, efficacy, safety) that are different for men and women?**  • No, review did not meet criteria **5.3. If adverse effects are reported, is information sex disaggregated?** • No, review did not meet criteria **5.4. Does review note that subgroup analyses by sex could not be done?** • Item was not applicable to the review |
| Discussion and conclusion | **6.1. Does the review report that primary studies analysed or failed to analyse results by sex?** • No, review did not meet criteria **6.2. Does the review address sex/gender implications for clinical practice?** • No, review did not meet criteria **6.3. Does the review address sex/gender implications for policy and regulation?** • No, review did not meet criteria **6.4. Does the review address sex/gender implications for research?** • No, review did not meet criteria |
| Table of included studies | **7.1. Does the description of included studies give detailed information on sex/gender of the study samples?** • No, review did not meet criteria (At least 7.1.1. or 7.1.2. are NO) **7.1.1. Detailed information on SEX of the study samples** • No, review did not meet criteria **7.1.2. Detailed information on GENDER of the study samples** • No, review did not meet criteria |
|  |  |
| **Item** | **Toon (2015)** |
| Background | **1.1 Are the terms sex and gender used in the background?** • No, review did not meet criteria **1.2 Are sex/gender identified as relevant or not to review question?** • No, review did not meet criteria **1.3. Does background discuss why sex/gender differences may be expected?** • No, review did not meet criteria |
| Objectives | **2.1. Are the terms sex, gender, male, or female used in objectives?** • No, review did not meet criteria *Quote: "To compare the benefits (such as potential improvements to quality of life) and harms (potentially increased wound-related morbidity) of early post-operative bathing or showering (i.e. within 48 hours after surgery) compared with delayed post-operative bathing or showering (i.e. no bathing or showering for over 48 hours after surgery) in patients with closed surgical wounds."* |
| Criteria for inclusion/ exclusion | **3.1. Do the review’s inclusion-exclusion criteria consider sex-gender differences?** • No, review did not meet criteria **3.2. Was there justification or explanation for the exclusion of some groups?** • No, review did not meet criteria |
| Methods | **4.1. Does the review examine whether outcome measures are different for males and females?** • No, review did not meet criteria **4.2. Did the review extract data by sex?** • No, review did not meet criteria **4.3. Did the review extract data on sex of withdrawals and dropouts?** • No, review did not meet criteria **4.4. In cases where sex/gender is used as a proxy for other measures (i.e., weight), is there an explanation for this approach?** • Item was not applicable to the review **4.5. Were any subgroup analyses completed?** • No, review did not meet criteria **4.6. Were subgroup analyses by sex completed?** • No, review did not meet criteria |
| Results and Analysis | **5.1. Do results distinguish between findings for males/females?** • No, review did not meet criteria **5.2. Does the review report conclusions (of effectiveness, efficacy, safety) that are different for men and women?**  • No, review did not meet criteria **5.3. If adverse effects are reported, is information sex disaggregated?** • Item was not applicable to the review **5.4. Does review note that subgroup analyses by sex could not be done?** • Item was not applicable to the review |
| Discussion and conclusion | **6.1. Does the review report that primary studies analysed or failed to analyse results by sex?** • No, review did not meet criteria **6.2. Does the review address sex/gender implications for clinical practice?** • No, review did not meet criteria **6.3. Does the review address sex/gender implications for policy and regulation?** • No, review did not meet criteria **6.4. Does the review address sex/gender implications for research?** • No, review did not meet criteria |
| Table of included studies | **7.1. Does the description of included studies give detailed information on sex/gender of the study samples?** • No, review did not meet criteria (At least 7.1.1. or 7.1.2. are NO) **7.1.1. Detailed information on SEX of the study samples** • No, review did not meet criteria **7.1.2. Detailed information on GENDER of the study samples** • No, review did not meet criteria |
|  |  |
| **Item** | **Torres (2015)** |
| Background | **1.1 Are the terms sex and gender used in the background?** • Yes, review met criteria *Quote: "It is one of the malignant tumours that causes the most deaths internationally, being the second most common cancer in men, after prostate cancer, and in women, after breast cancer."* **1.2 Are sex/gender identified as relevant or not to review question?** • Yes, review met criteria **1.3. Does background discuss why sex/gender differences may be expected?** • No, review did not meet criteria |
| Objectives | **2.1. Are the terms sex, gender, male, or female used in objectives?** • No, review did not meet criteria *Quote: "To assess the efficacy and safety of NIPPV for preventing complications in patients who underwent pulmonary resection for lung cancer."* |
| Criteria for inclusion/ exclusion | **3.1. Do the review’s inclusion-exclusion criteria consider sex-gender differences?** • No, review did not meet criteria *Quote: "Patients aged above 18 years of both genders, who underwent any type of lung resection (pneumectomy, lobectomy, segmentectomy) for lung cancer (small-cell lung carcinoma (SCLC) or non-small cell lung carcinoma (NSCLC))."* **3.2. Was there justification or explanation for the exclusion of some groups?** • No, review did not meet criteria |
| Methods | **4.1. Does the review examine whether outcome measures are different for males and females?** • No, review did not meet criteria **4.2. Did the review extract data by sex?** • No, review did not meet criteria **4.3. Did the review extract data on sex of withdrawals and dropouts?** • No, review did not meet criteria **4.4. In cases where sex/gender is used as a proxy for other measures (i.e., weight), is there an explanation for this approach?** • Item was not applicable to the review **4.5. Were any subgroup analyses completed?** • Yes, review met criteria *Quote: "We found no difference in the subgroup analysis considering ventilatory mode (bi-level or CPAP) and no difference between interventions after removing the quasi-randomised trial (Ludwig 2011)."* **4.6. Were subgroup analyses by sex completed?** • No, review did not meet criteria |
| Results and Analysis | **5.1. Do results distinguish between findings for males/females?** • No, review did not meet criteria **5.2. Does the review report conclusions (of effectiveness, efficacy, safety) that are different for men and women?**  • No, review did not meet criteria **5.3. If adverse effects are reported, is information sex disaggregated?** • Item was not applicable to the review **5.4. Does review note that subgroup analyses by sex could not be done?** • Item was not applicable to the review |
| Discussion and conclusion | **6.1. Does the review report that primary studies analysed or failed to analyse results by sex?** • No, review did not meet criteria **6.2. Does the review address sex/gender implications for clinical practice?** • No, review did not meet criteria **6.3. Does the review address sex/gender implications for policy and regulation?** • No, review did not meet criteria **6.4. Does the review address sex/gender implications for research?** • No, review did not meet criteria |
| Table of included studies | **7.1. Does the description of included studies give detailed information on sex/gender of the study samples?** • No, review did not meet criteria (At least 7.1.1. or 7.1.2. are NO) **7.1.1. Detailed information on SEX of the study samples** • No, review did not meet criteria **7.1.2. Detailed information on GENDER of the study samples** • No, review did not meet criteria |
|  |  |
| **Item** | **Ullman (2013)** |
| Background | **1.1 Are the terms sex and gender used in the background?** • No, review did not meet criteria **1.2 Are sex/gender identified as relevant or not to review question?** • No, review did not meet criteria **1.3. Does background discuss why sex/gender differences may be expected?** • No, review did not meet criteria |
| Objectives | **2.1. Are the terms sex, gender, male, or female used in objectives?** • No, review did not meet criteria *Quote: "The objective of this review was to identify any relationship between the frequency with which administration sets are replaced and rates of microbial colonization, infection and death."* |
| Criteria for inclusion/ exclusion | **3.1. Do the review’s inclusion-exclusion criteria consider sex-gender differences?** • No, review did not meet criteria **3.2. Was there justification or explanation for the exclusion of some groups?** • No, review did not meet criteria |
| Methods | **4.1. Does the review examine whether outcome measures are different for males and females?** • No, review did not meet criteria **4.2. Did the review extract data by sex?** • No, review did not meet criteria **4.3. Did the review extract data on sex of withdrawals and dropouts?** • No, review did not meet criteria **4.4. In cases where sex/gender is used as a proxy for other measures (i.e., weight), is there an explanation for this approach?** • Item was not applicable to the review **4.5. Were any subgroup analyses completed?** • Yes, review met criteria *Comment: See page 14.* **4.6. Were subgroup analyses by sex completed?** • No, review did not meet criteria |
| Results and Analysis | **5.1. Do results distinguish between findings for males/females?** • No, review did not meet criteria **5.2. Does the review report conclusions (of effectiveness, efficacy, safety) that are different for men and women?**  • No, review did not meet criteria **5.3. If adverse effects are reported, is information sex disaggregated?** • Item was not applicable to the review **5.4. Does review note that subgroup analyses by sex could not be done?** • Item was not applicable to the review |
| Discussion and conclusion | **6.1. Does the review report that primary studies analysed or failed to analyse results by sex?** • No, review did not meet criteria **6.2. Does the review address sex/gender implications for clinical practice?** • No, review did not meet criteria **6.3. Does the review address sex/gender implications for policy and regulation?** • No, review did not meet criteria **6.4. Does the review address sex/gender implications for research?** • No, review did not meet criteria |
| Table of included studies | **7.1. Does the description of included studies give detailed information on sex/gender of the study samples?** • No, review did not meet criteria (At least 7.1.1. or 7.1.2. are NO) **7.1.1. Detailed information on SEX of the study samples** • No, review did not meet criteria **7.1.2. Detailed information on GENDER of the study samples** • No, review did not meet criteria |
|  |  |
| **Item** | **Ullman (2015)** |
| Background | **1.1 Are the terms sex and gender used in the background?** • No, review did not meet criteria **1.2 Are sex/gender identified as relevant or not to review question?** • No, review did not meet criteria **1.3. Does background discuss why sex/gender differences may be expected?** • No, review did not meet criteria |
| Objectives | **2.1. Are the terms sex, gender, male, or female used in objectives?** • No, review did not meet criteria |
| Criteria for inclusion/ exclusion | **3.1. Do the review’s inclusion-exclusion criteria consider sex-gender differences?** • No, review did not meet criteria *Quote: "Any person of any age requiring a CVC in any healthcare or community setting. All CVCs were included, i.e. short- and long-term CVCs, tunnelled and non-tunnelled, port-a-caths, haemodialysis catheters, and peripherally-inserted central catheters (PICCs). For studies that included other types of vascular catheter, only data pertaining to CVCs were included."* **3.2. Was there justification or explanation for the exclusion of some groups?** • No, review did not meet criteria |
| Methods | **4.1. Does the review examine whether outcome measures are different for males and females?** • No, review did not meet criteria **4.2. Did the review extract data by sex?** • No, review did not meet criteria **4.3. Did the review extract data on sex of withdrawals and dropouts?** • No, review did not meet criteria **4.4. In cases where sex/gender is used as a proxy for other measures (i.e., weight), is there an explanation for this approach?** • Item was not applicable to the review **4.5. Were any subgroup analyses completed?** • No, review did not meet criteria **4.6. Were subgroup analyses by sex completed?** • No, review did not meet criteria |
| Results and Analysis | **5.1. Do results distinguish between findings for males/females?** • No, review did not meet criteria **5.2. Does the review report conclusions (of effectiveness, efficacy, safety) that are different for men and women?**  • No, review did not meet criteria **5.3. If adverse effects are reported, is information sex disaggregated?** • No, review did not meet criteria **5.4. Does review note that subgroup analyses by sex could not be done?** • Item was not applicable to the review |
| Discussion and conclusion | **6.1. Does the review report that primary studies analysed or failed to analyse results by sex?** • No, review did not meet criteria **6.2. Does the review address sex/gender implications for clinical practice?** • No, review did not meet criteria **6.3. Does the review address sex/gender implications for policy and regulation?** • No, review did not meet criteria **6.4. Does the review address sex/gender implications for research?** • No, review did not meet criteria |
| Table of included studies | **7.1. Does the description of included studies give detailed information on sex/gender of the study samples?** • No, review did not meet criteria (At least 7.1.1. or 7.1.2. are NO) **7.1.1. Detailed information on SEX of the study samples** • No, review did not meet criteria **7.1.2. Detailed information on GENDER of the study samples** • No, review did not meet criteria |
|  |  |
| **Item** | **van (2008)** |
| Background | **1.1 Are the terms sex and gender used in the background?** • No, review did not meet criteria **1.2 Are sex/gender identified as relevant or not to review question?** • No, review did not meet criteria **1.3. Does background discuss why sex/gender differences may be expected?** • No, review did not meet criteria |
| Objectives | **2.1. Are the terms sex, gender, male, or female used in objectives?** • No, review did not meet criteria *Quote: "To determine whether mupirocin nasal ointment reduces rates of S. aureus infection in patients who are nasal carriers of S. aureus."* |
| Criteria for inclusion/ exclusion | **3.1. Do the review’s inclusion-exclusion criteria consider sex-gender differences?** • No, review did not meet criteria *Quote: "Studies of nasal carriers (identified by microbiological culture) of S. aureus (both meticillin-resistant and meticillin-sensitive) that are using hospital services (either as inpatient or outpatient) were included. We included studies of patients from any population, gender and age."* **3.2. Was there justification or explanation for the exclusion of some groups?** • No, review did not meet criteria |
| Methods | **4.1. Does the review examine whether outcome measures are different for males and females?** • No, review did not meet criteria **4.2. Did the review extract data by sex?** • No, review did not meet criteria **4.3. Did the review extract data on sex of withdrawals and dropouts?** • No, review did not meet criteria **4.4. In cases where sex/gender is used as a proxy for other measures (i.e., weight), is there an explanation for this approach?** • Item was not applicable to the review **4.5. Were any subgroup analyses completed?** • Yes, review met criteria *Quote: "Subgroup analyses were planned where obvious differences existed between the included study groups; these differences might have existed in the following variables: age, healthcare setting (for example surgical compared with non-surgical), or length, timing, and dose of treatment. The performed subgroup analyses were based on healthcare setting, i.e. subgroup analyses were performed for surgical patients and for dialysis patients. No other subgroup analyses were performed because no obvious differences existed in age, or length, timing, and dose of treatment."* **4.6. Were subgroup analyses by sex completed?** • No, review did not meet criteria |
| Results and Analysis | **5.1. Do results distinguish between findings for males/females?** • No, review did not meet criteria **5.2. Does the review report conclusions (of effectiveness, efficacy, safety) that are different for men and women?**  • No, review did not meet criteria **5.3. If adverse effects are reported, is information sex disaggregated?** • No, review did not meet criteria **5.4. Does review note that subgroup analyses by sex could not be done?** • Item was not applicable to the review |
| Discussion and conclusion | **6.1. Does the review report that primary studies analysed or failed to analyse results by sex?** • No, review did not meet criteria **6.2. Does the review address sex/gender implications for clinical practice?** • No, review did not meet criteria **6.3. Does the review address sex/gender implications for policy and regulation?** • No, review did not meet criteria **6.4. Does the review address sex/gender implications for research?** • No, review did not meet criteria |
| Table of included studies | **7.1. Does the description of included studies give detailed information on sex/gender of the study samples?** • No, review did not meet criteria (At least 7.1.1. or 7.1.2. are NO) **7.1.1. Detailed information on SEX of the study samples** • No, review did not meet criteria **7.1.2. Detailed information on GENDER of the study samples** • No, review did not meet criteria |
|  |  |
| **Item** | **van (2013)** |
| Background | **1.1 Are the terms sex and gender used in the background?** • No, review did not meet criteria **1.2 Are sex/gender identified as relevant or not to review question?** • No, review did not meet criteria **1.3. Does background discuss why sex/gender differences may be expected?** • No, review did not meet criteria |
| Objectives | **2.1. Are the terms sex, gender, male, or female used in objectives?** • No, review did not meet criteria |
| Criteria for inclusion/ exclusion | **3.1. Do the review’s inclusion-exclusion criteria consider sex-gender differences?** • No, review did not meet criteria *Quote: "Adults and children with newly inserted long-term CVCs (TCVCs or TIDs) to facilitate chemotherapy."* **3.2. Was there justification or explanation for the exclusion of some groups?** • No, review did not meet criteria |
| Methods | **4.1. Does the review examine whether outcome measures are different for males and females?** • No, review did not meet criteria **4.2. Did the review extract data by sex?** • No, review did not meet criteria **4.3. Did the review extract data on sex of withdrawals and dropouts?** • No, review did not meet criteria **4.4. In cases where sex/gender is used as a proxy for other measures (i.e., weight), is there an explanation for this approach?** • Item was not applicable to the review **4.5. Were any subgroup analyses completed?** • Yes, review met criteria *Quote: "Where we identified substantial heterogeneity we investigated it using subgroup analyses and sensitivity analyses, where possible. Potential reasons for heterogeneity included types of participants (adults versus children), types of antibiotics (vancomycin versus others) and types of CVCs". Quote: "Differences were not significant for any of the antibiotic subgroups either".* **4.6. Were subgroup analyses by sex completed?** • No, review did not meet criteria |
| Results and Analysis | **5.1. Do results distinguish between findings for males/females?** • No, review did not meet criteria **5.2. Does the review report conclusions (of effectiveness, efficacy, safety) that are different for men and women?**  • No, review did not meet criteria **5.3. If adverse effects are reported, is information sex disaggregated?** • Item was not applicable to the review **5.4. Does review note that subgroup analyses by sex could not be done?** • Item was not applicable to the review |
| Discussion and conclusion | **6.1. Does the review report that primary studies analysed or failed to analyse results by sex?** • No, review did not meet criteria **6.2. Does the review address sex/gender implications for clinical practice?** • No, review did not meet criteria **6.3. Does the review address sex/gender implications for policy and regulation?** • No, review did not meet criteria **6.4. Does the review address sex/gender implications for research?** • No, review did not meet criteria |
| Table of included studies | **7.1. Does the description of included studies give detailed information on sex/gender of the study samples?** • No, review did not meet criteria (At least 7.1.1. or 7.1.2. are NO) **7.1.1. Detailed information on SEX of the study samples** • No, review did not meet criteria **7.1.2. Detailed information on GENDER of the study samples** • No, review did not meet criteria |
|  |  |
| **Item** | **Vermeulen (2004)** |
| Background | **1.1 Are the terms sex and gender used in the background?** • No, review did not meet criteria **1.2 Are sex/gender identified as relevant or not to review question?** • No, review did not meet criteria **1.3. Does background discuss why sex/gender differences may be expected?** • No, review did not meet criteria |
| Objectives | **2.1. Are the terms sex, gender, male, or female used in objectives?** • No, review did not meet criteria |
| Criteria for inclusion/ exclusion | **3.1. Do the review’s inclusion-exclusion criteria consider sex-gender differences?** • No, review did not meet criteria *Quote: "Men and women aged 18 years and over with surgical wounds healing by secondary intention".* **3.2. Was there justification or explanation for the exclusion of some groups?** • No, review did not meet criteria |
| Methods | **4.1. Does the review examine whether outcome measures are different for males and females?** • No, review did not meet criteria **4.2. Did the review extract data by sex?** • No, review did not meet criteria **4.3. Did the review extract data on sex of withdrawals and dropouts?** • No, review did not meet criteria **4.4. In cases where sex/gender is used as a proxy for other measures (i.e., weight), is there an explanation for this approach?** • Item was not applicable to the review **4.5. Were any subgroup analyses completed?** • No, review did not meet criteria **4.6. Were subgroup analyses by sex completed?** • No, review did not meet criteria |
| Results and Analysis | **5.1. Do results distinguish between findings for males/females?** • No, review did not meet criteria **5.2. Does the review report conclusions (of effectiveness, efficacy, safety) that are different for men and women?**  • No, review did not meet criteria **5.3. If adverse effects are reported, is information sex disaggregated?** • No, review did not meet criteria **5.4. Does review note that subgroup analyses by sex could not be done?** • Item was not applicable to the review |
| Discussion and conclusion | **6.1. Does the review report that primary studies analysed or failed to analyse results by sex?** • No, review did not meet criteria **6.2. Does the review address sex/gender implications for clinical practice?** • No, review did not meet criteria **6.3. Does the review address sex/gender implications for policy and regulation?** • No, review did not meet criteria **6.4. Does the review address sex/gender implications for research?** • No, review did not meet criteria |
| Table of included studies | **7.1. Does the description of included studies give detailed information on sex/gender of the study samples?** • No, review did not meet criteria (At least 7.1.1. or 7.1.2. are NO) **7.1.1. Detailed information on SEX of the study samples** • No, review did not meet criteria **7.1.2. Detailed information on GENDER of the study samples** • No, review did not meet criteria |
|  |  |
| **Item** | **Verschuur (2004)** |
| Background | **1.1 Are the terms sex and gender used in the background?** • No, review did not meet criteria **1.2 Are sex/gender identified as relevant or not to review question?** • No, review did not meet criteria **1.3. Does background discuss why sex/gender differences may be expected?** • No, review did not meet criteria |
| Objectives | **2.1. Are the terms sex, gender, male, or female used in objectives?** • No, review did not meet criteria |
| Criteria for inclusion/ exclusion | **3.1. Do the review’s inclusion-exclusion criteria consider sex-gender differences?** • No, review did not meet criteria *Quote: "Any patient undergoing clean or clean-contaminated types of ear surgery".* **3.2. Was there justification or explanation for the exclusion of some groups?** • No, review did not meet criteria |
| Methods | **4.1. Does the review examine whether outcome measures are different for males and females?** • No, review did not meet criteria **4.2. Did the review extract data by sex?** • No, review did not meet criteria **4.3. Did the review extract data on sex of withdrawals and dropouts?** • No, review did not meet criteria **4.4. In cases where sex/gender is used as a proxy for other measures (i.e., weight), is there an explanation for this approach?** • Item was not applicable to the review **4.5. Were any subgroup analyses completed?** • Yes, review met criteria *Quote: "Subgroup analyses for different ways of administrating the drug, e.g. systemic or local, was possible."* **4.6. Were subgroup analyses by sex completed?** • No, review did not meet criteria |
| Results and Analysis | **5.1. Do results distinguish between findings for males/females?** • No, review did not meet criteria **5.2. Does the review report conclusions (of effectiveness, efficacy, safety) that are different for men and women?**  • No, review did not meet criteria **5.3. If adverse effects are reported, is information sex disaggregated?** • No, review did not meet criteria **5.4. Does review note that subgroup analyses by sex could not be done?** • Item was not applicable to the review |
| Discussion and conclusion | **6.1. Does the review report that primary studies analysed or failed to analyse results by sex?** • No, review did not meet criteria **6.2. Does the review address sex/gender implications for clinical practice?** • No, review did not meet criteria **6.3. Does the review address sex/gender implications for policy and regulation?** • No, review did not meet criteria **6.4. Does the review address sex/gender implications for research?** • No, review did not meet criteria |
| Table of included studies | **7.1. Does the description of included studies give detailed information on sex/gender of the study samples?** • No, review did not meet criteria (At least 7.1.1. or 7.1.2. are NO) **7.1.1. Detailed information on SEX of the study samples** • No, review did not meet criteria **7.1.2. Detailed information on GENDER of the study samples** • No, review did not meet criteria |
|  |  |
| **Item** | **Villatoro (2010)** |
| Background | **1.1 Are the terms sex and gender used in the background?** • No, review did not meet criteria **1.2 Are sex/gender identified as relevant or not to review question?** • No, review did not meet criteria **1.3. Does background discuss why sex/gender differences may be expected?** • No, review did not meet criteria |
| Objectives | **2.1. Are the terms sex, gender, male, or female used in objectives?** • No, review did not meet criteria |
| Criteria for inclusion/ exclusion | **3.1. Do the review’s inclusion-exclusion criteria consider sex-gender differences?** • No, review did not meet criteria *Quote: "Patients with severe acute pancreatitis in whom pancreatic necrosis has been diagnosed by intravenous contrast enhanced CT according to internationally agreed criteria (Atlanta and Santorini criteria) (Bradley 1993; Dervenis 1999)."* **3.2. Was there justification or explanation for the exclusion of some groups?** • No, review did not meet criteria |
| Methods | **4.1. Does the review examine whether outcome measures are different for males and females?** • No, review did not meet criteria **4.2. Did the review extract data by sex?** • No, review did not meet criteria **4.3. Did the review extract data on sex of withdrawals and dropouts?** • No, review did not meet criteria **4.4. In cases where sex/gender is used as a proxy for other measures (i.e., weight), is there an explanation for this approach?** • Item was not applicable to the review **4.5. Were any subgroup analyses completed?** • Yes, review met criteria *Quote: "The data were analysed as comparisons between antibiotic and placebo with conservative management when available. The review protocol originally planned to categorise the data according to the following pre-specified subgroups: 1. the type of antibiotic regimen (i.e. beta-lactam, such as penicillin or cephalosporin based, or quinolone plus imidazole); 2. The time of commencement of therapy in relation to symptom onset and/or hospitalisation, and duration of therapy; 3. Aetiology of the attack (attributable to gallstones, alcohol, other identifiable causes, and idiopathic). Complete data was only found for the type of antibiotic regimen for the previous versions of this review, and therefore the other subgroup analyses have been omitted."* **4.6. Were subgroup analyses by sex completed?** • No, review did not meet criteria |
| Results and Analysis | **5.1. Do results distinguish between findings for males/females?** • No, review did not meet criteria **5.2. Does the review report conclusions (of effectiveness, efficacy, safety) that are different for men and women?**  • No, review did not meet criteria **5.3. If adverse effects are reported, is information sex disaggregated?** • No, review did not meet criteria **5.4. Does review note that subgroup analyses by sex could not be done?** • Item was not applicable to the review |
| Discussion and conclusion | **6.1. Does the review report that primary studies analysed or failed to analyse results by sex?** • No, review did not meet criteria **6.2. Does the review address sex/gender implications for clinical practice?** • No, review did not meet criteria **6.3. Does the review address sex/gender implications for policy and regulation?** • No, review did not meet criteria **6.4. Does the review address sex/gender implications for research?** • No, review did not meet criteria |
| Table of included studies | **7.1. Does the description of included studies give detailed information on sex/gender of the study samples?** • No, review did not meet criteria (At least 7.1.1. or 7.1.2. are NO) **7.1.1. Detailed information on SEX of the study samples** • No, review did not meet criteria **7.1.2. Detailed information on GENDER of the study samples** • No, review did not meet criteria |
|  |  |
| **Item** | **Vincent (2016)** |
| Background | **1.1 Are the terms sex and gender used in the background?** • No, review did not meet criteria **1.2 Are sex/gender identified as relevant or not to review question?** • No, review did not meet criteria **1.3. Does background discuss why sex/gender differences may be expected?** • No, review did not meet criteria |
| Objectives | **2.1. Are the terms sex, gender, male, or female used in objectives?** • No, review did not meet criteria *Quote: "To determine whether the wearing of disposable surgical face masks by the surgical team during clean surgery reduces postoperative surgical wound infection."* |
| Criteria for inclusion/ exclusion | **3.1. Do the review’s inclusion-exclusion criteria consider sex-gender differences?** • No, review did not meet criteria *Quote: "Adults and children undergoing clean surgery".* **3.2. Was there justification or explanation for the exclusion of some groups?** • No, review did not meet criteria |
| Methods | **4.1. Does the review examine whether outcome measures are different for males and females?** • No, review did not meet criteria **4.2. Did the review extract data by sex?** • No, review did not meet criteria **4.3. Did the review extract data on sex of withdrawals and dropouts?** • No, review did not meet criteria **4.4. In cases where sex/gender is used as a proxy for other measures (i.e., weight), is there an explanation for this approach?** • Item was not applicable to the review **4.5. Were any subgroup analyses completed?** • No, review did not meet criteria **4.6. Were subgroup analyses by sex completed?** • No, review did not meet criteria |
| Results and Analysis | **5.1. Do results distinguish between findings for males/females?** • No, review did not meet criteria **5.2. Does the review report conclusions (of effectiveness, efficacy, safety) that are different for men and women?**  • No, review did not meet criteria **5.3. If adverse effects are reported, is information sex disaggregated?** • Item was not applicable to the review **5.4. Does review note that subgroup analyses by sex could not be done?** • Item was not applicable to the review |
| Discussion and conclusion | **6.1. Does the review report that primary studies analysed or failed to analyse results by sex?** • No, review did not meet criteria **6.2. Does the review address sex/gender implications for clinical practice?** • No, review did not meet criteria **6.3. Does the review address sex/gender implications for policy and regulation?** • No, review did not meet criteria **6.4. Does the review address sex/gender implications for research?** • No, review did not meet criteria |
| Table of included studies | **7.1. Does the description of included studies give detailed information on sex/gender of the study samples?** • No, review did not meet criteria (At least 7.1.1. or 7.1.2. are NO) **7.1.1. Detailed information on SEX of the study samples** • No, review did not meet criteria **7.1.2. Detailed information on GENDER of the study samples** • No, review did not meet criteria |
|  |  |
| **Item** | **Wang (2016)** |
| Background | **1.1 Are the terms sex and gender used in the background?** • No, review did not meet criteria **1.2 Are sex/gender identified as relevant or not to review question?** • No, review did not meet criteria **1.3. Does background discuss why sex/gender differences may be expected?** • No, review did not meet criteria |
| Objectives | **2.1. Are the terms sex, gender, male, or female used in objectives?** • No, review did not meet criteria *Quote: "To assess the effectiveness and safety of semi-recumbent positioning versus supine positioning to prevent ventilator-associated pneumonia (VAP) in adults requiring mechanical ventilation."* |
| Criteria for inclusion/ exclusion | **3.1. Do the review’s inclusion-exclusion criteria consider sex-gender differences?** • No, review did not meet criteria *Quote: "We included adult patients (18 years or older) undergoing endotracheal intubation and mechanical ventilation. We excluded studies among which more than 15% of patients were ineligible for semi-recumbent positioning, e.g. abdominal surgery, obesity (body mass index (BMI) greater than 30 kgm2) (WHO 2000)."* **3.2. Was there justification or explanation for the exclusion of some groups?** • No, review did not meet criteria |
| Methods | **4.1. Does the review examine whether outcome measures are different for males and females?** • No, review did not meet criteria **4.2. Did the review extract data by sex?** • No, review did not meet criteria **4.3. Did the review extract data on sex of withdrawals and dropouts?** • No, review did not meet criteria **4.4. In cases where sex/gender is used as a proxy for other measures (i.e., weight), is there an explanation for this approach?** • Item was not applicable to the review **4.5. Were any subgroup analyses completed?** • Yes, review met criteria *Quote: "Pre-defined subgroup analysis only found a significant interaction between blinding of outcome assessors and body positioning for clinically suspected VAP."* **4.6. Were subgroup analyses by sex completed?** • No, review did not meet criteria |
| Results and Analysis | **5.1. Do results distinguish between findings for males/females?** • No, review did not meet criteria **5.2. Does the review report conclusions (of effectiveness, efficacy, safety) that are different for men and women?**  • No, review did not meet criteria **5.3. If adverse effects are reported, is information sex disaggregated?** • Item was not applicable to the review **5.4. Does review note that subgroup analyses by sex could not be done?** • Item was not applicable to the review |
| Discussion and conclusion | **6.1. Does the review report that primary studies analysed or failed to analyse results by sex?** • No, review did not meet criteria **6.2. Does the review address sex/gender implications for clinical practice?** • No, review did not meet criteria **6.3. Does the review address sex/gender implications for policy and regulation?** • No, review did not meet criteria **6.4. Does the review address sex/gender implications for research?** • No, review did not meet criteria |
| Table of included studies | **7.1. Does the description of included studies give detailed information on sex/gender of the study samples?** • No, review did not meet criteria (At least 7.1.1. or 7.1.2. are NO) **7.1.1. Detailed information on SEX of the study samples** • Unable to determine **7.1.2. Detailed information on GENDER of the study samples** • No, review did not meet criteria |
|  |  |
| **Item** | **Wang (2016)** |
| Background | **1.1 Are the terms sex and gender used in the background?** • Yes, review met criteria *Quote: "The incidence of catheter-related thrombosis varies by catheter location (Trerotola 2000), sex, systemic prothrombotic states, site of insertion (subclavian compared with internal jugular) (Trerotola 2000), previous catheter-related thrombosis and catheter malposition (Liangos 2006; Trerotola 2000)."* **1.2 Are sex/gender identified as relevant or not to review question?** • Unable to determine **1.3. Does background discuss why sex/gender differences may be expected?** • No, review did not meet criteria *Quote: "The incidence of catheter-related thrombosis varies by catheter location (Trerotola 2000), sex, systemic prothrombotic states, site of insertion (subclavian compared with internal jugular) (Trerotola 2000), previous catheter-related thrombosis and catheter malposition (Liangos 2006; Trerotola 2000)."* |
| Objectives | **2.1. Are the terms sex, gender, male, or female used in objectives?** • No, review did not meet criteria *Quote: "This review aimed to compare the prophylactic effect of different anticoagulant agents, preparations, doses and administration on the incidence of central venous haemodialysis catheter-related malfunction and sepsis in patients with ESKD"* |
| Criteria for inclusion/ exclusion | **3.1. Do the review’s inclusion-exclusion criteria consider sex-gender differences?** • No, review did not meet criteria *Quote: "Studies conducted in people with ESKD who require CVC for initiation or maintenance haemodialysis access were included. Studies enrolling patients who had been treated previously with anticoagulants for thrombotic events were included in the review."* **3.2. Was there justification or explanation for the exclusion of some groups?** • No, review did not meet criteria |
| Methods | **4.1. Does the review examine whether outcome measures are different for males and females?** • No, review did not meet criteria **4.2. Did the review extract data by sex?** • No, review did not meet criteria **4.3. Did the review extract data on sex of withdrawals and dropouts?** • No, review did not meet criteria **4.4. In cases where sex/gender is used as a proxy for other measures (i.e., weight), is there an explanation for this approach?** • Item was not applicable to the review **4.5. Were any subgroup analyses completed?** • Yes, review met criteria *Quote: "Planned a priori subgroup analyses were used to explore possible sources of heterogeneity. Heterogeneity in prevention of catheter malfunction in alternative anticoagulant locking solutions could be related to different class of interventions, i.e. citrate, rt-PA, LMWH and antibiotic locking solutions. Whether the use of a co-intervention or not can also cause heterogeneity of the results, which was also analysed."* **4.6. Were subgroup analyses by sex completed?** • No, review did not meet criteria |
| Results and Analysis | **5.1. Do results distinguish between findings for males/females?** • No, review did not meet criteria **5.2. Does the review report conclusions (of effectiveness, efficacy, safety) that are different for men and women?**  • No, review did not meet criteria **5.3. If adverse effects are reported, is information sex disaggregated?** • No, review did not meet criteria **5.4. Does review note that subgroup analyses by sex could not be done?** • Item was not applicable to the review |
| Discussion and conclusion | **6.1. Does the review report that primary studies analysed or failed to analyse results by sex?** • No, review did not meet criteria **6.2. Does the review address sex/gender implications for clinical practice?** • No, review did not meet criteria **6.3. Does the review address sex/gender implications for policy and regulation?** • No, review did not meet criteria **6.4. Does the review address sex/gender implications for research?** • No, review did not meet criteria |
| Table of included studies | **7.1. Does the description of included studies give detailed information on sex/gender of the study samples?** • No, review did not meet criteria (At least 7.1.1. or 7.1.2. are NO) **7.1.1. Detailed information on SEX of the study samples** • Unable to determine **7.1.2. Detailed information on GENDER of the study samples** • No, review did not meet criteria |
|  |  |
| **Item** | **Webster (2014)** |
| Background | **1.1 Are the terms sex and gender used in the background?** • No, review did not meet criteria **1.2 Are sex/gender identified as relevant or not to review question?** • No, review did not meet criteria **1.3. Does background discuss why sex/gender differences may be expected?** • No, review did not meet criteria |
| Objectives | **2.1. Are the terms sex, gender, male, or female used in objectives?** • No, review did not meet criteria |
| Criteria for inclusion/ exclusion | **3.1. Do the review’s inclusion-exclusion criteria consider sex-gender differences?** • No, review did not meet criteria **3.2. Was there justification or explanation for the exclusion of some groups?** • No, review did not meet criteria |
| Methods | **4.1. Does the review examine whether outcome measures are different for males and females?** • No, review did not meet criteria **4.2. Did the review extract data by sex?** • No, review did not meet criteria **4.3. Did the review extract data on sex of withdrawals and dropouts?** • No, review did not meet criteria **4.4. In cases where sex/gender is used as a proxy for other measures (i.e., weight), is there an explanation for this approach?** • Item was not applicable to the review **4.5. Were any subgroup analyses completed?** • Yes, review met criteria **4.6. Were subgroup analyses by sex completed?** • No, review did not meet criteria |
| Results and Analysis | **5.1. Do results distinguish between findings for males/females?** • No, review did not meet criteria **5.2. Does the review report conclusions (of effectiveness, efficacy, safety) that are different for men and women?**  • No, review did not meet criteria **5.3. If adverse effects are reported, is information sex disaggregated?** • No, review did not meet criteria **5.4. Does review note that subgroup analyses by sex could not be done?** • Item was not applicable to the review |
| Discussion and conclusion | **6.1. Does the review report that primary studies analysed or failed to analyse results by sex?** • No, review did not meet criteria **6.2. Does the review address sex/gender implications for clinical practice?** • No, review did not meet criteria **6.3. Does the review address sex/gender implications for policy and regulation?** • No, review did not meet criteria **6.4. Does the review address sex/gender implications for research?** • No, review did not meet criteria |
| Table of included studies | **7.1. Does the description of included studies give detailed information on sex/gender of the study samples?** • No, review did not meet criteria (At least 7.1.1. or 7.1.2. are NO) **7.1.1. Detailed information on SEX of the study samples** • No, review did not meet criteria **7.1.2. Detailed information on GENDER of the study samples** • No, review did not meet criteria |
|  |  |
| **Item** | **Webster (2015)** |
| Background | **1.1 Are the terms sex and gender used in the background?** • No, review did not meet criteria **1.2 Are sex/gender identified as relevant or not to review question?** • No, review did not meet criteria **1.3. Does background discuss why sex/gender differences may be expected?** • No, review did not meet criteria |
| Objectives | **2.1. Are the terms sex, gender, male, or female used in objectives?** • No, review did not meet criteria |
| Criteria for inclusion/ exclusion | **3.1. Do the review’s inclusion-exclusion criteria consider sex-gender differences?** • No, review did not meet criteria *Quote: "We considered for inclusion, trials recruiting people of any age or gender, undergoing any type of inpatient or outpatient surgery."* **3.2. Was there justification or explanation for the exclusion of some groups?** • No, review did not meet criteria |
| Methods | **4.1. Does the review examine whether outcome measures are different for males and females?** • No, review did not meet criteria **4.2. Did the review extract data by sex?** • No, review did not meet criteria **4.3. Did the review extract data on sex of withdrawals and dropouts?** • No, review did not meet criteria **4.4. In cases where sex/gender is used as a proxy for other measures (i.e., weight), is there an explanation for this approach?** • Item was not applicable to the review **4.5. Were any subgroup analyses completed?** • Yes, review met criteria *Quote: "We had planned the following four subgroup analyses. 1. Clean surgery compared with contaminated surgery. 2. Individual compared with cluster allocation. 3. Prophylactic antibiotic compared with no prophylaxis. 4. Hair clipping compared with shaving. The only subgroup analysis that was possible, based on available data, was of clean compared with contaminated surgery".* **4.6. Were subgroup analyses by sex completed?** • No, review did not meet criteria |
| Results and Analysis | **5.1. Do results distinguish between findings for males/females?** • No, review did not meet criteria **5.2. Does the review report conclusions (of effectiveness, efficacy, safety) that are different for men and women?**  • No, review did not meet criteria **5.3. If adverse effects are reported, is information sex disaggregated?** • Item was not applicable to the review **5.4. Does review note that subgroup analyses by sex could not be done?** • Item was not applicable to the review |
| Discussion and conclusion | **6.1. Does the review report that primary studies analysed or failed to analyse results by sex?** • No, review did not meet criteria **6.2. Does the review address sex/gender implications for clinical practice?** • No, review did not meet criteria **6.3. Does the review address sex/gender implications for policy and regulation?** • No, review did not meet criteria **6.4. Does the review address sex/gender implications for research?** • No, review did not meet criteria |
| Table of included studies | **7.1. Does the description of included studies give detailed information on sex/gender of the study samples?** • No, review did not meet criteria (At least 7.1.1. or 7.1.2. are NO) **7.1.1. Detailed information on SEX of the study samples** • No, review did not meet criteria **7.1.2. Detailed information on GENDER of the study samples** • No, review did not meet criteria |
|  |  |
| **Item** | **Webster (2015)** |
| Background | **1.1 Are the terms sex and gender used in the background?** • No, review did not meet criteria **1.2 Are sex/gender identified as relevant or not to review question?** • No, review did not meet criteria **1.3. Does background discuss why sex/gender differences may be expected?** • No, review did not meet criteria |
| Objectives | **2.1. Are the terms sex, gender, male, or female used in objectives?** • No, review did not meet criteria |
| Criteria for inclusion/ exclusion | **3.1. Do the review’s inclusion-exclusion criteria consider sex-gender differences?** • No, review did not meet criteria **3.2. Was there justification or explanation for the exclusion of some groups?** • No, review did not meet criteria |
| Methods | **4.1. Does the review examine whether outcome measures are different for males and females?** • No, review did not meet criteria **4.2. Did the review extract data by sex?** • No, review did not meet criteria **4.3. Did the review extract data on sex of withdrawals and dropouts?** • No, review did not meet criteria **4.4. In cases where sex/gender is used as a proxy for other measures (i.e., weight), is there an explanation for this approach?** • Item was not applicable to the review **4.5. Were any subgroup analyses completed?** • No, review did not meet criteria **4.6. Were subgroup analyses by sex completed?** • No, review did not meet criteria |
| Results and Analysis | **5.1. Do results distinguish between findings for males/females?** • Item was not applicable to the review **5.2. Does the review report conclusions (of effectiveness, efficacy, safety) that are different for men and women?**  • Item was not applicable to the review **5.3. If adverse effects are reported, is information sex disaggregated?** • Item was not applicable to the review **5.4. Does review note that subgroup analyses by sex could not be done?** • Item was not applicable to the review |
| Discussion and conclusion | **6.1. Does the review report that primary studies analysed or failed to analyse results by sex?** • Item was not applicable to the review **6.2. Does the review address sex/gender implications for clinical practice?** • No, review did not meet criteria **6.3. Does the review address sex/gender implications for policy and regulation?** • No, review did not meet criteria **6.4. Does the review address sex/gender implications for research?** • No, review did not meet criteria |
| Table of included studies | **7.1. Does the description of included studies give detailed information on sex/gender of the study samples?** • Item was not applicable to the review **7.1.1. Detailed information on SEX of the study samples** • Item was not applicable to the review **7.1.2. Detailed information on GENDER of the study samples** • Item was not applicable to the review |
|  |  |
| **Item** | **Webster (2015)** |
| Background | **1.1 Are the terms sex and gender used in the background?** • No, review did not meet criteria **1.2 Are sex/gender identified as relevant or not to review question?** • No, review did not meet criteria **1.3. Does background discuss why sex/gender differences may be expected?** • No, review did not meet criteria |
| Objectives | **2.1. Are the terms sex, gender, male, or female used in objectives?** • No, review did not meet criteria *Quote: "To review the evidence for preoperative bathing or showering with antiseptics for the prevention of surgical site infections."* |
| Criteria for inclusion/ exclusion | **3.1. Do the review’s inclusion-exclusion criteria consider sex-gender differences?** • No, review did not meet criteria *Quote: "Men, women and children undergoing any type of surgery in any setting."* **3.2. Was there justification or explanation for the exclusion of some groups?** • No, review did not meet criteria |
| Methods | **4.1. Does the review examine whether outcome measures are different for males and females?** • No, review did not meet criteria **4.2. Did the review extract data by sex?** • No, review did not meet criteria *Quote: "The following data were extracted [...] using a piloted data extraction sheet: type of study, study setting, number of participants, sex, mean age, predisposing risk factors, type of antiseptic solutions, use of prophylactic antibiotics, procedure and timing for full body wash, period of community follow-up, all primary and secondary outcome descriptions and outcome measures reported, including infection rates and study authors’ conclusions."* **4.3. Did the review extract data on sex of withdrawals and dropouts?** • No, review did not meet criteria **4.4. In cases where sex/gender is used as a proxy for other measures (i.e., weight), is there an explanation for this approach?** • Item was not applicable to the review **4.5. Were any subgroup analyses completed?** • No, review did not meet criteria **4.6. Were subgroup analyses by sex completed?** • No, review did not meet criteria |
| Results and Analysis | **5.1. Do results distinguish between findings for males/females?** • No, review did not meet criteria **5.2. Does the review report conclusions (of effectiveness, efficacy, safety) that are different for men and women?**  • No, review did not meet criteria **5.3. If adverse effects are reported, is information sex disaggregated?** • No, review did not meet criteria **5.4. Does review note that subgroup analyses by sex could not be done?** • Item was not applicable to the review |
| Discussion and conclusion | **6.1. Does the review report that primary studies analysed or failed to analyse results by sex?** • No, review did not meet criteria **6.2. Does the review address sex/gender implications for clinical practice?** • No, review did not meet criteria **6.3. Does the review address sex/gender implications for policy and regulation?** • No, review did not meet criteria **6.4. Does the review address sex/gender implications for research?** • No, review did not meet criteria |
| Table of included studies | **7.1. Does the description of included studies give detailed information on sex/gender of the study samples?** • No, review did not meet criteria (At least 7.1.1. or 7.1.2. are NO) **7.1.1. Detailed information on SEX of the study samples** • No, review did not meet criteria **7.1.2. Detailed information on GENDER of the study samples** • No, review did not meet criteria |
|  |  |
| **Item** | **Westendorp (2012)** |
| Background | **1.1 Are the terms sex and gender used in the background?** • No, review did not meet criteria **1.2 Are sex/gender identified as relevant or not to review question?** • No, review did not meet criteria **1.3. Does background discuss why sex/gender differences may be expected?** • No, review did not meet criteria |
| Objectives | **2.1. Are the terms sex, gender, male, or female used in objectives?** • No, review did not meet criteria |
| Criteria for inclusion/ exclusion | **3.1. Do the review’s inclusion-exclusion criteria consider sex-gender differences?** • No, review did not meet criteria *Quote: "All patients with acute ischaemic or haemorrhagic stroke, aged 18 years or older. We included trials that did not differentiate between ischaemic or haemorrhagic stroke by computed tomography (CT) or magnetic resonance imaging (MRI) prior to inclusion in the trial, on the basis that 75% to 90% of strokes are ischaemic in predominantly white populations."* **3.2. Was there justification or explanation for the exclusion of some groups?** • No, review did not meet criteria |
| Methods | **4.1. Does the review examine whether outcome measures are different for males and females?** • No, review did not meet criteria **4.2. Did the review extract data by sex?** • No, review did not meet criteria **4.3. Did the review extract data on sex of withdrawals and dropouts?** • No, review did not meet criteria **4.4. In cases where sex/gender is used as a proxy for other measures (i.e., weight), is there an explanation for this approach?** • Item was not applicable to the review **4.5. Were any subgroup analyses completed?** • No, review did not meet criteria **4.6. Were subgroup analyses by sex completed?** • No, review did not meet criteria |
| Results and Analysis | **5.1. Do results distinguish between findings for males/females?** • No, review did not meet criteria **5.2. Does the review report conclusions (of effectiveness, efficacy, safety) that are different for men and women?**  • No, review did not meet criteria **5.3. If adverse effects are reported, is information sex disaggregated?** • No, review did not meet criteria **5.4. Does review note that subgroup analyses by sex could not be done?** • Item was not applicable to the review |
| Discussion and conclusion | **6.1. Does the review report that primary studies analysed or failed to analyse results by sex?** • No, review did not meet criteria **6.2. Does the review address sex/gender implications for clinical practice?** • No, review did not meet criteria **6.3. Does the review address sex/gender implications for policy and regulation?** • No, review did not meet criteria **6.4. Does the review address sex/gender implications for research?** • No, review did not meet criteria |
| Table of included studies | **7.1. Does the description of included studies give detailed information on sex/gender of the study samples?** • No, review did not meet criteria (At least 7.1.1. or 7.1.2. are NO) **7.1.1. Detailed information on SEX of the study samples** • No, review did not meet criteria **7.1.2. Detailed information on GENDER of the study samples** • No, review did not meet criteria |
|  |  |
| **Item** | **Wetterslev (2015)** |
| Background | **1.1 Are the terms sex and gender used in the background?** • No, review did not meet criteria **1.2 Are sex/gender identified as relevant or not to review question?** • No, review did not meet criteria **1.3. Does background discuss why sex/gender differences may be expected?** • No, review did not meet criteria |
| Objectives | **2.1. Are the terms sex, gender, male, or female used in objectives?** • No, review did not meet criteria |
| Criteria for inclusion/ exclusion | **3.1. Do the review’s inclusion-exclusion criteria consider sex-gender differences?** • No, review did not meet criteria *Quote: "We included surgical patients 18 years of age or older who were undergoing elective or emergency surgery."* **3.2. Was there justification or explanation for the exclusion of some groups?** • No, review did not meet criteria |
| Methods | **4.1. Does the review examine whether outcome measures are different for males and females?** • No, review did not meet criteria **4.2. Did the review extract data by sex?** • No, review did not meet criteria **4.3. Did the review extract data on sex of withdrawals and dropouts?** • No, review did not meet criteria **4.4. In cases where sex/gender is used as a proxy for other measures (i.e., weight), is there an explanation for this approach?** • Item was not applicable to the review **4.5. Were any subgroup analyses completed?** • Yes, review met criteria **4.6. Were subgroup analyses by sex completed?** • No, review did not meet criteria |
| Results and Analysis | **5.1. Do results distinguish between findings for males/females?** • No, review did not meet criteria **5.2. Does the review report conclusions (of effectiveness, efficacy, safety) that are different for men and women?**  • No, review did not meet criteria **5.3. If adverse effects are reported, is information sex disaggregated?** • No, review did not meet criteria **5.4. Does review note that subgroup analyses by sex could not be done?** • Item was not applicable to the review |
| Discussion and conclusion | **6.1. Does the review report that primary studies analysed or failed to analyse results by sex?** • No, review did not meet criteria **6.2. Does the review address sex/gender implications for clinical practice?** • No, review did not meet criteria **6.3. Does the review address sex/gender implications for policy and regulation?** • No, review did not meet criteria **6.4. Does the review address sex/gender implications for research?** • No, review did not meet criteria |
| Table of included studies | **7.1. Does the description of included studies give detailed information on sex/gender of the study samples?** • No, review did not meet criteria (At least 7.1.1. or 7.1.2. are NO) **7.1.1. Detailed information on SEX of the study samples** • No, review did not meet criteria **7.1.2. Detailed information on GENDER of the study samples** • No, review did not meet criteria |
|  |  |
| **Item** | **Wojcieszek (2014)** |
| Background | **1.1 Are the terms sex and gender used in the background?** • Item was not applicable to the review **1.2 Are sex/gender identified as relevant or not to review question?** • Item was not applicable to the review **1.3. Does background discuss why sex/gender differences may be expected?** • Item was not applicable to the review |
| Objectives | **2.1. Are the terms sex, gender, male, or female used in objectives?** • Item was not applicable to the review |
| Criteria for inclusion/ exclusion | **3.1. Do the review’s inclusion-exclusion criteria consider sex-gender differences?** • Item was not applicable to the review **3.2. Was there justification or explanation for the exclusion of some groups?** • Item was not applicable to the review |
| Methods | **4.1. Does the review examine whether outcome measures are different for males and females?** • Item was not applicable to the review **4.2. Did the review extract data by sex?** • Item was not applicable to the review **4.3. Did the review extract data on sex of withdrawals and dropouts?** • Item was not applicable to the review **4.4. In cases where sex/gender is used as a proxy for other measures (i.e., weight), is there an explanation for this approach?** • Item was not applicable to the review **4.5. Were any subgroup analyses completed?** • Item was not applicable to the review **4.6. Were subgroup analyses by sex completed?** • Item was not applicable to the review |
| Results and Analysis | **5.1. Do results distinguish between findings for males/females?** • Item was not applicable to the review **5.2. Does the review report conclusions (of effectiveness, efficacy, safety) that are different for men and women?**  • Item was not applicable to the review **5.3. If adverse effects are reported, is information sex disaggregated?** • Item was not applicable to the review **5.4. Does review note that subgroup analyses by sex could not be done?** • Item was not applicable to the review |
| Discussion and conclusion | **6.1. Does the review report that primary studies analysed or failed to analyse results by sex?** • Item was not applicable to the review **6.2. Does the review address sex/gender implications for clinical practice?** • Item was not applicable to the review **6.3. Does the review address sex/gender implications for policy and regulation?** • Item was not applicable to the review **6.4. Does the review address sex/gender implications for research?** • Item was not applicable to the review |
| Table of included studies | **7.1. Does the description of included studies give detailed information on sex/gender of the study samples?** • Item was not applicable to the review **7.1.1. Detailed information on SEX of the study samples** • Item was not applicable to the review **7.1.2. Detailed information on GENDER of the study samples** • Item was not applicable to the review |
|  |  |
| **Item** | **Wood (2016)** |
| Background | **1.1 Are the terms sex and gender used in the background?** • No, review did not meet criteria **1.2 Are sex/gender identified as relevant or not to review question?** • No, review did not meet criteria **1.3. Does background discuss why sex/gender differences may be expected?** • No, review did not meet criteria |
| Objectives | **2.1. Are the terms sex, gender, male, or female used in objectives?** • No, review did not meet criteria |
| Criteria for inclusion/ exclusion | **3.1. Do the review’s inclusion-exclusion criteria consider sex-gender differences?** • No, review did not meet criteria *Quote: "Trials involving participants undergoing any type of clean surgery in an operating theatre."* **3.2. Was there justification or explanation for the exclusion of some groups?** • No, review did not meet criteria |
| Methods | **4.1. Does the review examine whether outcome measures are different for males and females?** • No, review did not meet criteria **4.2. Did the review extract data by sex?** • No, review did not meet criteria **4.3. Did the review extract data on sex of withdrawals and dropouts?** • No, review did not meet criteria **4.4. In cases where sex/gender is used as a proxy for other measures (i.e., weight), is there an explanation for this approach?** • Item was not applicable to the review **4.5. Were any subgroup analyses completed?** • No, review did not meet criteria **4.6. Were subgroup analyses by sex completed?** • No, review did not meet criteria |
| Results and Analysis | **5.1. Do results distinguish between findings for males/females?** • No, review did not meet criteria **5.2. Does the review report conclusions (of effectiveness, efficacy, safety) that are different for men and women?**  • No, review did not meet criteria **5.3. If adverse effects are reported, is information sex disaggregated?** • No, review did not meet criteria **5.4. Does review note that subgroup analyses by sex could not be done?** • Item was not applicable to the review |
| Discussion and conclusion | **6.1. Does the review report that primary studies analysed or failed to analyse results by sex?** • No, review did not meet criteria **6.2. Does the review address sex/gender implications for clinical practice?** • No, review did not meet criteria **6.3. Does the review address sex/gender implications for policy and regulation?** • No, review did not meet criteria **6.4. Does the review address sex/gender implications for research?** • No, review did not meet criteria |
| Table of included studies | **7.1. Does the description of included studies give detailed information on sex/gender of the study samples?** • No, review did not meet criteria (At least 7.1.1. or 7.1.2. are NO) **7.1.1. Detailed information on SEX of the study samples** • No, review did not meet criteria **7.1.2. Detailed information on GENDER of the study samples** • No, review did not meet criteria |
|  |  |
