# Supplementary material for: Consideration of sex and gender in Cochrane reviews of interventions for preventing healthcare-associated infections: a methodology study
Source: BMC Health Serv Res. 2019 Mar 15;19:169. doi: 10.1186/s12913-019-4001-9 (PMC6419810; doi:10.1186/s12913-019-4001-9)
Supplement: Supplementary file 7 — Sex and gender appraisal summary. (PDF 643 kb) [file 12913_2019_4001_MOESM7_ESM.pdf]

Additional file 7: Sex and gender appraisal summary

	1.1. Are the terms sex and gender used in the background?	1.2. Are sex/gender identified as relevant or not to review question?	1.3. Does background discuss why sex/gender differences may be expected?	2.1. Are the terms sex, gender, male, or female used in objectives?	3.1. Do the review's inclusion/exclusion criteria consider sex-gender differences?	3.2. Was there justification or explanation for the exclusion of some groups?	4.1. Does the review examine whether outcome measures are different for males and females?	4.2. Did the review extract data by sex?	4.3. Did the review extract data on sex of withdrawals and dropouts?	4.4. If sex/gender is used as a proxy for other measures (i.e., weight), is this explained?	4.5. Were any subgroup analyses completed?	4.6. Were subgroup analyses by sex completed?	5.1. Do results distinguish between findings for males/females?	5.2. Does the review report conclusions that are different for men and women?	5.3. If adverse effects are reported, is information sex disaggregated?	5.4. Does review note that subgroup analyses by sex could not be done?	6.1. Does the review report that primary studies analysed or failed to analyse results by sex?	6.2. Does the review address sex/gender implications for clinical practice?	6.3. Does the review address sex/gender implications for policy and regulation?	6.4. Does the review address sex/gender implications for research?	7.1. Does the description of included studies give detailed information on sex/gender of the study samples?
Alkhawaja 2015	-	-	-	-	-	-	-	-	-		+	-	-	-	-	-	-	-	-	-	-
Andersen 2005	+	?	-	-	-	-	-	-	-		+	-	-	-		-	-	-	-	-	-
Arnold 2005	-	-	-	-	-	-	-	-	-		-	-	-	-			-	-	-	-	-
Arora 2010	-	-	-	-	-	-	-	-	-		-	-	-	-			-	-	-	-	-
Arrowsmith 2014	-	-	-	-	-	-	-	-	-		-	-	-	-			-	-	-	-	-
Barajas-Nava 2013	-	-	-	-	-	-	-	-	-		-	-	-	-	-		-	-	-	-	-
Bo 2014	-	-	-	-	-	-	-	-	-		+	-	-	-	-		-	-	-	-	-
Bradford 2015	-	-	-	-	-	-	-	-	-		-	-	-	-	-		-	-	-	-	-
Brand 2010	-	-	-	-	-	-	-	-	-		+	-	-	-	-		-	-	-	-	-
Brand 2013	-	-	-	-	-	-	-	-	-		-	-						-	-	-	
Brass 2015	-	-	-	-	-	-	-	-	-		-	-	-	-	-		-	-	-	-	-
Bravo 2016	-	-	-	-	-	-	-	-	-		-	-	-	-			-	-	-	-	-
Brignardello-Petersen 2015	+	?	-	-	-	-	-	-	-		-	-	-	-	-		-	-	-	-	-
Cooper 2016	+	?	-	-	-	-	-	-	-		-	-	-	-	-	+	-	-	-	+	-
D'Amico 2009	-	-	-	-	-	-	-	-	-		+	-	-	-			-	-	-	-	-
Dhiwakar 2012	-	-	-	-	-	-	-	-	-		-	-	-	-	-		-	-	-	-	-
Dumville 2015	-	-	-	-	-	-	-	-	-		-	-	-	-			-	-	-	-	-
Dumville 2016	-	-	-	-	-	-	-	-	-		-	-	-	-			-	-	-	-	-
Ejemot-Nwadiaro 2015	-	-	-	-	-	-	-	-	-		+	-	-	-			-	-	-	-	-
Fernandez 2012	-	-	-	-	-	-	-	-	-		-	-	-	-			-	-	-	-	-
Flodgren 2013	+	+	-	-	-	-	-	-	-		-	-	-	-			-	-	-	-	-
Flodgren 2016	-	-	-	-	-	-	-	-	-		-	-	-	-			-	-	-	-	-
Foon 2012	+	-	-	-	-	-	-	-	-		-	-	-	-	-	+	-	-	-	-	-
Gavin 2016	-	-	-	-	-	-	-	-	-		-	-	-	-	-		-	-	-	-	-
Ge 2012	-	-	-	-	-	-	-	-	-		+	-	-	-	-		-	-	-	-	-
Gillespie 2010	-	-	-	-	-	-	-	-	-		+	-	-	-	-		-	-	-	-	-
Glenny 2013	-	-	-	-	-	-	-	-	-		-	-	-	-			-	-	-	-	-
Gosselin 2004	-	-	-	-	-	-	-	-	-		+	-	-	-			-	-	-	-	-
Gould 2010	-	-	-	-	-	-	-	-	-		-	-	-	-			-	-	-	-	-
Griffiths 2007	-	-	-	-	-	-	-	-	-		+	+	-	-	-		-	-	-	-	-
Gurusamy 2011	-	-	-	-	-	-	-	-	-		-	-	-	-	-		-	-	-	-	-
Gurusamy 2013a	-	-	-	-	-	-	-	-	-		-	-	-	-	-		-	-	-	-	-
Gurusamy 2013b	-	-	-	-	-	-	-	-	-		-	-	-	-	-		-	-	-	-	-
Gyte 2014																					
Haas 2014																					
Hadiati 2014																					
Heal 2016	-	-	-	-	-	-	-	-	-		-	-	-	-	-		-	-	-	-	-
Hsu 2016	-	-	-	-	-	-	-	-	-		-	-	-	-	-		-	-	-	-	-
Hua 2016	-	-	-	-	-	-	-	-	-		+	-	-	-	-		-	-	-	-	-
Hughes 2013	+	?	-	-	-	-	-	-	-		-	-	-	-			-	-	-	-	-
Ireland 2014	-	-	-	-	-	-	-	-	-		-	-	-	-	-		-	-	-	-	-
Jamison 2013	+	-	-	-	-	-	-	-	-		-	-				+		-	-	-	
Jefferson 2011	-	-	-	-	-	-	-	-	-		+	-	-	-			-	-	-	-	-
Jones 2014	+	?	-	-	-	-	-	-	-		+	-	-	-	-		-	-	-	-	-
Kao 2009	-	-	-	-	-	-	-	-	-		+	-	-	-	-		-	-	-	-	-
Kelly 2010	-	-	-	-	-	-	-	-	-		+	-	-	-	-		-	-	-	-	-
Kidd 2015	-	-	-	-	-	-	-	-	-		+	+	-	-	-		-	-	-	+	-
Lai 2016a	-	-	-	-	-	-	-	-	-		+	-	-	-	-		-	-	-	-	-
Lai 2016b	-	-	-	-	-	-	-	-	-		+	-	-	-	-		-	-	-	-	-
Lam 2014	+	?	-	-	-	-	-	-	-		+	+	?	?	-		-	+	-	-	-
Lethaby 2013	-	-	-	-	-	-	-	-	-		-	-	-	-		?	-	-	-	-	-
Liabsuetrakul 2014																					
Lipp 2013	-	-	-	-	-	-	-	-	-		+	-	-	-	-		-	-	-	-	-
Lo 2015	-	-	-	-	-	-	-	-	-		-	-						-	-	-	
Lodi 2012	-	-	-	-	-	-	-	-	-		+	-	-	-	-		-	-	-	-	-
Loeb 2003	-	-	-	-	-	-	-	-	-		-	-	-	-	-		-	-	-	-	-
López-Alcalde 2015	-	-	-	-	-	-	-	-	-		-	-						-	-	-	
Lopez-Briz 2014	-	-	-	-	-	-	-	-	-		-	-	-	-	-		-	-	-	-	-
Low 2012																					
Lusardi 2013	-	-	-	-	-	-	-	-	-		-	-	-	-	-	+	-	-	-	-	-
Mackeen 2014																					
Madrid 2016	-	-	-	-	-	-	-	-	-		+	-	-	-	-		-	-	-	-	-
Marsh 2015	-	-	-	-	-	-	-	-	-		-	-	-	-	-		-	-	-	-	-
Masters 2012	-	-	-	-	-	-	-	-	-		-	-	-	-			-	-	-	-	-
May 2007																					
McCann 2010	-	-	-	-	-	-	-	-	-		+	-	-	-	-		-	-	-	-	-
Nabhan 2016																					
Nelson 2014	-	-	-	-	-	-	-	-	-		-	-	-	-			-	-	-	-	-
Niel-Weise 2012	-	-	-	-	-	-	-	-	-		-	-	-	-	-	+	-	-	-	-	-
Norman 2016a	-	-	-	-	-	-	-	-	-		-	-	-	-	-		-	-	-	-	-
Norman 2016b	-	-	-	-	-	-	-	-	-		-	-	-	-			-	-	-	-	-
Phipps 2006	-	-	-	-	-	-	-	-	-		-	-	-	-	-	+	-	-	-	-	-
Prayle 2010	-	-	-	-	-	-	-	-	-		-	-	-	-	-		-	-	-	-	-
Ratilal 2006	-	-	-	-	-	-	-	-	-		+	-	-	-	-		-	-	-	-	-
Ray 2014																					
Robertson-Malt 2014	-	-	-	-	-	-	-	-	-		-	-	-	-	-		-	-	-	-	-
Sajid 2013	+	?	-	-	-	-	-	-	-		+	-	-	-	-		-	-	-	-	-
Sanabria 2010	-	-	-	-	-	-	-	-	-		-	-	-	-			-	-	-	-	-
Sanchez-Manuel 2012	-	-	-	-	-	-	-	-	-		+	-	-	-			-	-	-	-	-
Shah 2014	-	-	-	-	-	-	-	-	-		-	-	-	-	-		-	-	-	-	-
Smaill 2014																					
Stewart 2006	-	-	-	-	-	-	-	-	-		-	-	-	-			-	-	-	-	-
Storm-Versloot 2010	-	-	-	-	-	-	-	-	-		+	-	-	-	-		-	-	-	-	-
Strippoli 2004a	-	-	-	-	-	-	-	-	-		-	-	-	-	-		-	-	-	-	-
Strippoli 2004b	-	-	-	-	-	-	-	-	-		-	-	-	-			-	-	-	-	-
Subirana 2007	-	-	-	-	-	-	-	-	-		-	-	-	-			-	-	-	-	-
Syed 2013	-	-	-	-	-	-	-	-	-		+	-	-	-	-	+	-	-	-	-	-
Tanner 2006	-	-	-	-	-	-	-	-	-		+	-	-	-		-	-	-	-	-	-
Tanner 2011	-	-	-	-	-	-	-	-	-		-	-	-	-			-	-	-	-	-
Tanner 2016	-	-	-	-	-	-	-	-	-		-	-	-	-			-	-	-	-	-
Thomas 2016	-	-	-	-	-	-	-	-	-		+	-	-	-			-	-	-	-	-
Tokmaji 2015	-	-	-	-	-	-	-	-	-		-	-	-	-	-		-	-	-	-	-
Toon 2015	-	-	-	-	-	-	-	-	-		-	-	-	-	-		-	-	-	-	-
Toon 2015a	-	-	-	-	-	-	-	-	-		-	-	-	-	-		-	-	-	-	-
Torres 2015	+	+	-	-	-	-	-	-	-		+	-	-	-			-	-	-	-	-
Ullman 2013	-	-	-	-	-	-	-	-	-		+	-	-	-			-	-	-	-	-
Ullman 2015	-	-	-	-	-	-	-	-	-		-	-	-	-	-		-	-	-	-	-
van de Wetering 2013	-	-	-	-	-	-	-	-	-		+	-	-	-	-		-	-	-	-	-
van Rijen 2008	-	-	-	-	-	-	-	-	-		+	-	-	-			-	-	-	-	-
Vermeulen 2004	-	-	-	-	-	-	-	-	-		-	-	-	-	-		-	-	-	-	-
Verschuur 2004	-	-	-	-	-	-	-	-	-		+	-	-	-	-		-	-	-	-	-
Villatoro 2010	-	-	-	-	-	-	-	-	-		+	-	-	-	-		-	-	-	-	-
Vincent 2016	-	-	-	-	-	-	-	-	-		-	-	-	-			-	-	-	-	-
Wang 2016a	+	?	-	-	-	-	-	-	-		+	-	-	-	-		-	-	-	-	-
Wang 2016b	-	-	-	-	-	-	-	-	-		+	-	-	-			-	-	-	-	-
Webster 2014	-	-	-	-	-	-	-	-	-		+	-	-	-	-		-	-	-	-	-
Webster 2015a	-	-	-	-	-	-	-	-	-		-	-	-	-	-		-	-	-	-	-
Webster 2015b	-	-	-	-	-	-	-	-	-		-	-						-	-	-	
Webster 2015c	-	-	-	-	-	-	-	-	-		+	-	-	-			-	-	-	-	-
Westendorp 2012	-	-	-	-	-	-	-	-	-		-	-	-	-	-		-	-	-	-	-
Wetterslev 2015	-	-	-	-	-	-	-	-	-		+	-	-	-	-		-	-	-	-	-
Wojcieszek 2014																					
Wood 2016	-	-	-	-	-	-	-	-	-		-	-	-	-	-		-	-	-	-	-
